# Supplementary material for: Nickel/biimidazole-catalyzed electrochemical enantioselective reductive cross-coupling of aryl aziridines with aryl iodides
Source: Nat Commun. 2023 Apr 22;14:2322. doi: 10.1038/s41467-023-37965-0 (PMC10122672; doi:10.1038/s41467-023-37965-0)
Supplement: Supplementary file 1 — Supplementary Information [file 41467_2023_37965_MOESM1_ESM.pdf]

Enantioselective nickel-catalyzed electrochemical reductive cross-coupling of aryl aziridines with aryl iodides: enhanced reactivity of nickel by an electron-rich biimidazole ligand

Yun-Zhao Wang,[1,4] Zhen-Hua Wang,[1,4] Inbal L. Eshel,[2,4] Bing Sun,[1] Dong Liu,[1] Yu-Cheng Gu,[3] Anat Milo,[*,2] Tian-Sheng Mei[*,1]

[1]State Key Laboratory of Organometallic Chemistry, Shanghai Institute of Organic Chemistry, University of Chinese Academy of Sciences, CAS, Shanghai, China

[2]Department of Chemistry, Ben-Gurion University of the Negev, Beer-Sheva 841051, Israel

[3]Syngenta, Jealott's Hill International Research Centre, Berkshire RE42 6EY, U.K.

[4]Contributed equally to this work

*Correspondence: mei7900@sioc.ac.cn; anatmilo@bgu.ac.il

Table of Contents

1. Supplementary Notes 3

2. Supplementary Discussion 4

   2.1 Preparation of Aziridines and Ligands 4

   2.2. Synthetic Application and Characterization of Products 12

   2.3. Cyclic Voltammetry Studies 18

   2.4 Optimization Details 24

   2.5 Unsuccessful Substrates 30

   2.6 Large-Scale Synthesis and Mechanistic Studies 35

   2.7 Synthetic Procedures and Characterization of Products 38

3. Supplementary Figures 63

   3.1 Mathematical modeling 63

   3.2. X-Ray Crystal X-Ray Structures and Data 84

   3.3 ^{1}H NMR, ^{13}C NMR, ^{19}F NMR 88

   3.4 HPLC Spectra Data 132

4. References 177

1. Supplementary Notes

Commercially available materials were used without further purification. Column chromatography was performed using either 100–200 Mesh or 300–400 Mesh silica gel. Visualization of spots on the LC plate was accomplished with UV light (254 nm) and staining over I_2 chamber.

All commercial reagents were purchased from TCI, Sigma-Aldrich, Adamas-beta, J&K, Bidepharm, Leyan, 9-Ding chemistry, and Energy Chemical of the highest purity grade. They were used without further purification unless specified. Sodium iodide (99%) was purchased from TCI and was used without further purification. Nickel(II) chloride ethylene glycol dimethyl ether (≥97%) was purchased from Stream and was used as received. DMAc (99.8%, SuperDry) was purchased from J&K and was used without further purification. ^{1}H NMR and ^{13}C NMR spectra were recorded on Agilent AV 400, and Varian Inova 400 (400 MHz and 100 MHz, respectively). ^{19}F NMR spectra were recorded on Agilent AV 400, Varian Inova 400 (376 MHz) instrument. The peaks were internally referenced to TMS (0.00 ppm) or residual undeuterated solvent signal. The following abbreviations were used to explain multiplicities: s = singlet, d = doublet, t = triplet, q = quartet, m = multiplet, and br = broad. Infrared spectra were obtained on a Bio-Rad FTS-185 instrument High-resolution mass spectra were recorded at the Center for Mass Spectrometry, Shanghai Institute of Organic Chemistry. Analytical and spectral data of all those known compounds are exactly matching with the reported values. All air-and moisture-sensitive reactions were performed under an atmosphere of nitrogen-flamed dried glassware.

2. Supplementary Discussion

2.1 Preparation of Aziridines and Ligands

Preparation of Ligands

Synthesis of L7:

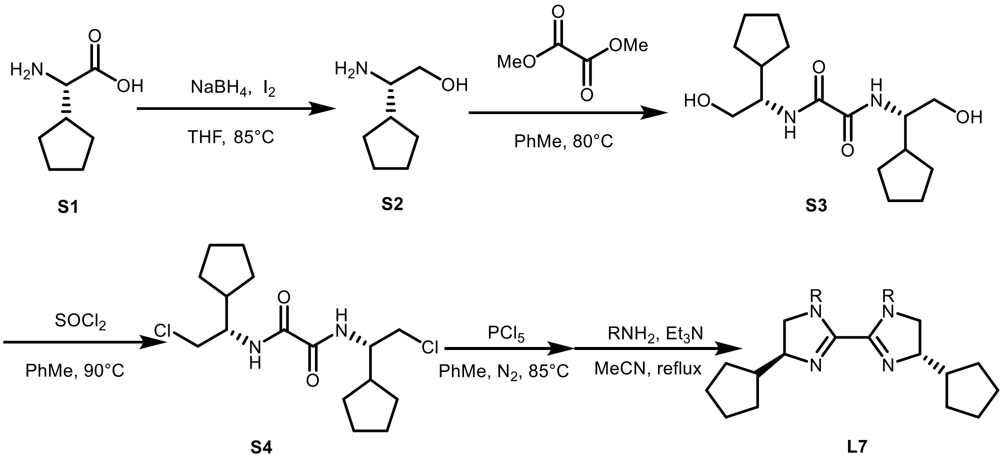

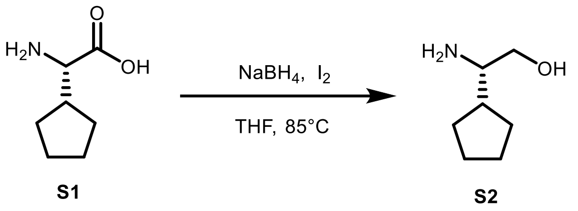

The biimidazole ligands were synthesized according to the previously reported method.[1-4]

(S)-2-amino-2-cyclopentylethan-1-ol (S2) The amino acid (35 mmol, 1 equiv., 5.0 g), and $NaBH_4$ (105 mmol, 3 equiv., 3.9 g), and 140 mL THF were added to a dry round-bottom flask and cooled to 0 °C. A solution of I_2 (35 mmol, 1 equiv., 8.8 g) in 70 mL THF was added dropwise, allowing the mixture to turn white after each drop. Following the addition, the mixture was heated to reflux for 15 hours. The reaction mixture was then cooled to 0 °C and quenched slowly with MeOH. The solvents were removed in vacuo, and the remaining solid was dissolved in 20% KOH and stirred at 50 °C for 1.5 hours. The mixture was cooled to room temperature, and extracted with EtOAc (3 x 100 mL), and the combined organic extracts were dried over $MgSO_4$ and filtered. The

solvent was removed in vacuo, and the white solid (**S2**, 4.5 g, 99.7% yield) was taken forward.

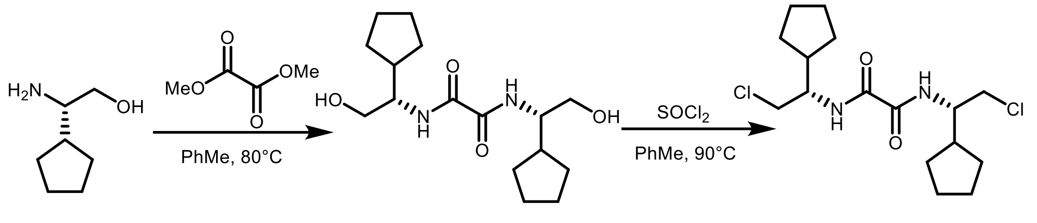

S2 **S3** **S4**

(S)-2-amino-2-cyclopentylethan-1-ol (2.0 equiv, 30 mmol, 3.87 g) and dimethyloxalate (1.0 equiv., 15 mmol, 1.77 g) were dissolved in toluene (350 mL) and heated to 80 °C. The reaction was allowed to stir at 80 °C for 12 h with the diamide precipitating out of the solution as a white solid. The reaction was cooled to room temperature and concentrated in a vacuum. The crude diol was dissolved in toluene (100 mL) and heated to 70 °C whereupon thionyl chloride (2.2 equiv., 33 mmol, 2.4 mL) was added. The reaction was stirred at 70 °C for 30 minutes then heated to 90 °C for 2 h. The reaction was cooled to room temperature and concentrated under reduced pressure to afford the dichloro-intermediate(**S4**) which was used without further purification.

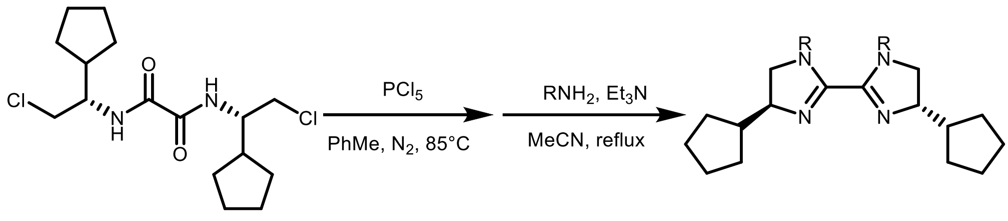

S4 **L7**

To an overdried three-necked flask was added the crude dichloro-intermediate (**S4**, 1.0 equiv., 6.8 mmol, 2.4 g) and phosphorus pentachloride (2.4 equiv., 16.2 mmol, 3.4 g) in 50 mL of toluene under N_2 atmosphere. The reaction was allowed to stir at 85 °C for 8 h before it was cooled to room temperature. Toluene was evaporated under reduced pressure and Et_3N (6 equiv., 41 mmol, 5.6 mL) and 3-*tert*-butylbenzenamine (6.0 equiv., 40.5 mmol, 6.0 g) in 100 mL of CH_3CN was added. The reaction was heated to reflux under N_2 until TLC showed complete consumption of the starting material. After cooling to room temperature, water was added and the aqueous layer was separated and extracted three times with DCM. The organic layer was dried with Na_2SO_4, filtered, and concentrated under reduced pressure. The residue was purified by flash column chromatography (PE:EA:TEA = 50:10:1) to give **L7** (1.8 g, 4.0 mmol) in 50% yield as

a white solid. All the biimidazole ligands in this research were synthesized by following Procedure.

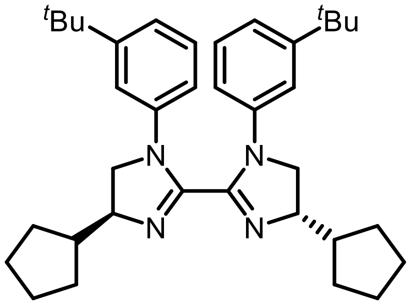

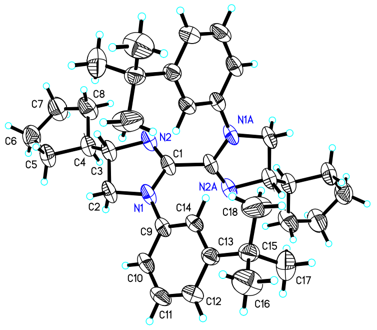

(4S,4'S)-1,1'-bis(3-(tert-butyl)phenyl)-4,4'-dicyclopentyl-4,4',5,5'-tetrahydro-1H,1'H-2,2'-biimidazole (L7): white solid. 50% yield. **^{1}H NMR (400 MHz, $CDCl_3$)** δ 7.05 – 6.88 (m, 2H), 6.72 (s, 1H), 6.46 (d, *J* = 7.6 Hz, 1H), 4.04 (q, *J* = 9.6 Hz, 1H), 3.78 (t, *J* = 9.6 Hz, 1H), 3.50 (t, *J* = 9.6 Hz, 1H), 2.20 – 2.06 (m, 1H), 2.06 – 1.90 (m, 1H), 1.76 (m, 1H), 1.61 (m, 5H), 1.22 (s, 9H). **^{13}C NMR (101 MHz, $CDCl_3$)** δ 153.6, 151.6, 139.7, 127.9, 120.4, 116.9, 116.7, 69.9, 55.8, 45.5, 34.6, 31.4, 30.2, 29.5, 25.4, 25.3. **HRMS** (ESI) calcd for $C_{36}H_{51}N_4$ $[M+H]^+$: 539.41122; found: 539.41082.

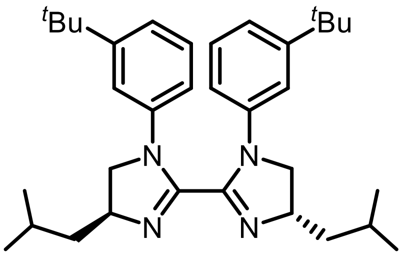

(4S,4'S)-1,1'-bis(3-(tert-butyl)phenyl)-4,4'-diisobutyl-4,4',5,5'-tetrahydro-1H,1'H-2,2'-biimidazole (L5): white solid. 19% yield. **^{1}H NMR (400 MHz, $CDCl_3$)** δ 7.03 – 6.94 (m, 2H), 6.76 (d, *J* = 1.6 Hz, 1H), 6.47 – 6.35 (m, 1H), 4.33 – 4.19 (m, 1H), 3.82 (t, *J* = 9.6 Hz, 1H), 3.41 (t, *J* = 9.2 Hz, 1H), 1.95 – 1.74 (m, 2H), 1.42 (m, 1H), 1.24 (s, 9H), 0.97 (d, *J* = 6.4 Hz, 3H), 0.95 (d, *J* = 6.4 Hz, 3H). **^{13}C NMR (101 MHz, $CDCl_3$)** δ 153.3, 151.7, 139.6, 128.0, 120.6, 116.9, 116.9, 63. 0, 57.2, 45.4, 34.7, 31.4, 25.2, 22.8, 22.7. **HRMS** (ESI) calcd for $C_{34}H_{51}N_4$ $[M+H]^+$: 515.41072; found: 515.41082.

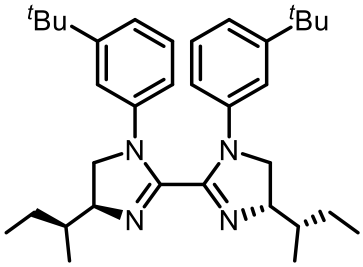

(4S,4'S)-4,4'-di((S)-sec-butyl)-1,1'-bis(3-(tert-butyl)phenyl)-4,4',5,5'-tetrahydro-1H,1'H-2,2'-biimidazole (L6): white solid. 60% yield. **^{1}H NMR (400 MHz, $CDCl_3$)** δ 7.04 – 6.90 (m, 2H), 6.69 (s, 1H), 6.54 – 6.41 (m, 1H), 4.08 (m, 1H), 3.69 (t, J = 9.6 Hz, 1H), 3.53 (m, 1H), 1.87 – 1.71 (m, 2H), 1.28 (m, 1H), 1.22 (s, 9H), 0.99 – 0.89 (m, 6H). **^{13}C NMR (101 MHz, $CDCl_3$)** δ 153.4, 151.6, 139.7, 127.8, 120.4, 116.9, 116.6, 69.6, 53.9, 39.4, 34.6, 31.4, 26.3, 14.9, 11.5. **HRMS** (ESI) calcd for $C_{34}H_{51}N_4$ $[M+H]^+$: 515.41023; found: 515.41082.

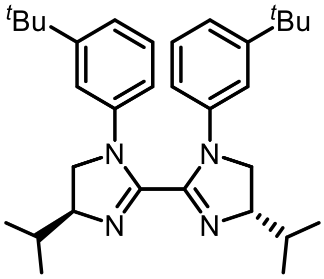

(4S,4'S)-1,1'-bis(3-(tert-butyl)phenyl)-4,4'-diisopropyl-4,4',5,5'-tetrahydro-1H,1'H-2,2'-biimidazole(L24): white solid. 17% yield. **^{1}H NMR (400 MHz, $CDCl_3$)** δ 7.04 – 6.88 (m, 2H), 6.70 (s, 1H), 6.47 (dd, J = 7.2, 1.6 Hz, 1H), 3.95 (m, 1H), 3.73 (t, J = 9.6 Hz, 1H), 3.53 (t, J = 9.6 Hz, 1H), 1.93 (m, 1H), 1.22 (s, 9H), 1.11 (d, J = 6.8 Hz, 3H), 0.96 (d, J = 6.8 Hz, 3H). **^{13}C NMR (101 MHz, $CDCl_3$)** δ 153.5, 151.6, 139.7, 127.9, 120.4, 116.9, 116.6, 71.2, 54.4, 34.6, 33.1, 31.4, 19.8, 18.8.

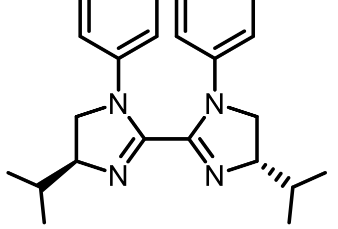

(4S,4'S)-4,4'-diisopropyl-1,1'-bis(3-(trifluoromethyl)phenyl)-4,4',5,5'-tetrahydro-1H,1'H-2,2'-biimidazole (L23) : white solid. 40% yield. **^{1}H NMR (400 MHz, $CDCl_3$)**

δ 7.13 (d, *J* = 5.2 Hz, 2H), 6.82 (s, 1H), 6.76 – 6.67 (m, 1H), 4.05 (m, 1H), 3.68 (t, *J* = 9.2 Hz, 1H), 3.58 (m, 1H), 1.99 (m, 1H), 1.17 (d, *J* = 6.8 Hz, 3H), 1.03 (d, *J* = 6.8 Hz, 3H). **^{13}C NMR (101 MHz, $CDCl_3$)** δ 152.3, 139.9, 131.1 (q, *J* = 32.2 Hz), 128.9, 123.7(q, *J* = 271.0 Hz), 121.5, 119.6(q, *J* = 3.8 Hz), 115.2(q, *J* = 3.7 Hz), 71.5, 53.7, 32.9, 19.7. **^{19}F NMR (376 MHz, $CDCl_3$)** δ -62.59. **HRMS** (ESI) calcd for $C_{26}H_{29}N_4F_6$ $[M+H]^+$: 511.22896; found: 511.22909.

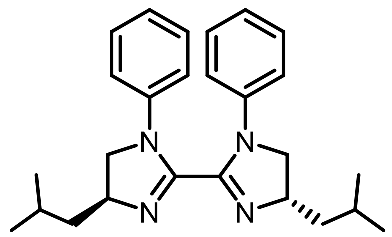

(4S,4'S)-4,4'-diisobutyl-1,1'-diphenyl-4,4',5,5'-tetrahydro-1H,1'H-2,2'-biimidazole (L27): white solid. 65% yield. **^{1}H NMR (400 MHz, $CDCl_3$)** δ 7.08 (t, *J* = 8.0 Hz, 2H), 6.92 (t, *J* = 7.2 Hz, 1H), 6.68 (d, *J* = 8.0 Hz, 2H), 4.33 – 4.19 (m, 1H), 3.81 (t, *J* = 9.2 Hz, 1H), 3.38 (t, *J* = 9.2 Hz, 1H), 1.90 (m, 1H), 1.77 (m, 1H), 1.44 – 1.34 (m, 1H), 0.98 (d, *J* = 8.0 Hz, 3H), 0.95 (d, *J* = 8.0 Hz, 3H). **^{13}C NMR (101 MHz, $CDCl_3$)** δ 153.1, 139.8, 128.3, 123.1, 119.3, 63.1, 56.7, 45.5, 25.1, 22.9, 22.6. **HRMS** (ESI) calcd for $C_{26}H_{35}N_4$ $[M+H]^+$: 403.28551; found: 403.28562.

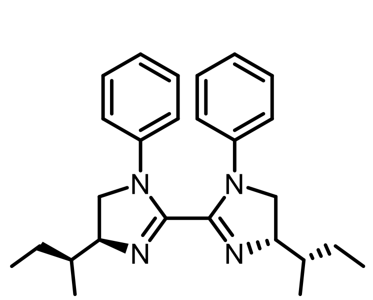

(4S,4'S)-4,4'-di((S)-sec-butyl)-1,1'-diphenyl-4,4',5,5'-tetrahydro-1H,1'H-2,2'-biimidazole (L26): white solid. 44% yield. **^{1}H NMR (400 MHz, $CDCl_3$)** δ 7.05 (t, *J* = 8.0 Hz, 2H), 6.90 (t, *J* = 7.2 Hz, 1H), 6.64 (d, *J* = 7.6 Hz, 2H), 4.14 (m, 1H), 3.63 (t, *J* = 9.6 Hz, 1H), 3.53 (dd, *J* = 11.6, 9.2 Hz, 1H), 1.91 – 1.78 (m, 1H), 1.77 – 1.64 (m, 1H), 1.35 – 1.21 (m, 1H), 0.96 (m, 6H). **^{13}C NMR (101 MHz, $CDCl_3$)** δ 153.3, 139.7, 128.3, 123.2, 119.3, 69.5, 53.2, 39.1, 26.3, 15.0, 11.5. **HRMS** (ESI) calcd for $C_{26}H_{35}N_4$ $[M+H]^+$: 403.28518; found: 403.28562.

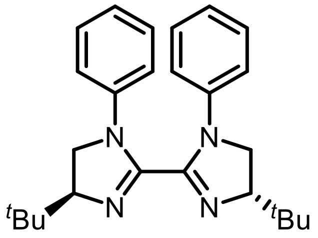

(4S,4'S)-4,4'-di-tert-butyl-1,1'-diphenyl-4,4',5,5'-tetrahydro-1H,1'H-2,2'-biimidazole (L25): ^{1}H NMR (400 MHz, CDCl$_3$) δ 6.99 (t, *J* = 7.6 Hz, 2H), 6.87 (d, *J* = 7.6 Hz, 1H), 6.58 (d, *J* = 7.6 Hz, 2H), 4.04 (dd, *J* = 13.2, 10.0 Hz, 1H), 3.60 (dd, *J* = 13.2, 9.2 Hz, 1H), 3.48 (t, *J* = 9.6 Hz, 1H), 1.06 (s, 9H). **^{13}C NMR (101 MHz, CDCl$_3$)** δ 153.5, 139.6, 128.1, 123.0, 119.1, 74.5, 52.0, 33.7, 26.6. **HRMS** (ESI) calcd for $C_{26}H_{35}N_4$ [M+H]$^+$: 403.28502; found: 403.28562.

Preparation of Aziridines

A round bottle flask was flame dried and charged with $TsNH_2$ (1.4 equiv.), $Cu(MeCN)_4PF_6$ (0.1 equiv.), alkene (1.0 equiv., 10 mmol), activated 3 Å molecular sieves (1.0 g/mmol alkene) and MeCN (2.5 mL/mmol alkene). The mixture was cooled in a 0 °C ice-water bath, and iodosylbenzene (1.4 equiv.) was added in one portion. The mixture was allowed to warm to room temperature and stirred at room temperature overnight. The resulting mixture was filtered through a pad of Celite, and the filtrate was concentrated. The crude was purified with column chromatography.

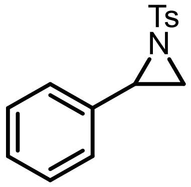

2-phenyl-1-tosylaziridine (1a) Prepared according to Method A, and the title compound was isolated as a white solid (1.6 g, 59% yield) with spectral data in agreement with literature values.

^{1}H NMR (400 MHz, CDCl$_3$) δ 7.87 (d, *J* = 8.4 Hz, 2H), 7.32 (d, *J* = 8.0 Hz, 2H), 7.29-7.26 (m, 3H), 7.23 – 7.18 (m, 2H), 3.77 (dd, *J* = 7.2, 4.4 Hz, 1H), 2.98 (d, *J* = 7.2 Hz, 1H), 2.42 (s, 3H), 2.38 (d, *J* = 4.4 Hz, 1H).

^{13}C NMR (101 MHz, CDCl$_3$) δ 144.6, 135.5, 135.0, 129.7, 128.5, 128.3, 127.9, 126.5,

41.0, 35.9, 21.6.

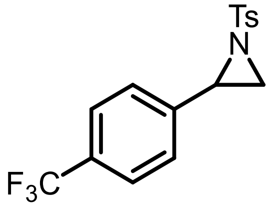

1-tosyl-2-(4-(trifluoromethyl)phenyl)aziridine(1b) Prepared according to Method A, and the title compound was isolated as a white solid (2.7 g, 98% yield) with spectral data in agreement with literature values.

^{1}H NMR (400 MHz, CDCl$_3$) δ 7.87 (d, J = 8.4 Hz, 2H), 7.55 (d, J = 8.0 Hz, 2H), 7.34 (d, J = 8.0Hz, 4H), 3.81 (dd, J = 7.2, 4.4 Hz, 1H), 3.02 (d, J = 7.2 Hz, 1H), 2.44 (s, 3H), 2.37 (d, J = 4.4 Hz, 1H).

^{13}C NMR (101 MHz, CDCl$_3$) δ 145.0, 139.3, 139.2, 134.7, 130.5 (q, J = 32 Hz), 129.9, 128.0, 126.9, 125.6 (q, J = 3.7 Hz), 123.9 (q, J = 270 Hz), 40.2, 36.2, 21.7.

^{19}F NMR (376 MHz, CDCl$_3$) δ -62.68.

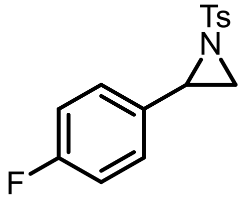

2-(4-fluorophenyl)-1-tosylaziridine(1c) Prepared according to Method A, and the title compound was isolated as a white solid (1.3 g, 45% yield) with spectral data in agreement with literature values.

^{1}H NMR (400 MHz, CDCl$_3$) δ 7.86 (d, J = 8.4 Hz, 2H), 7.34 (d, J = 8.0 Hz, 2H), 7.22 – 7.15 (m, 2H), 6.98 (t, J = 8.8 Hz, 2H), 3.75 (dd, J = 7.2, 4.4 Hz, 1H), 2.97 (d, J = 7.2 Hz, 1H), 2.44 (s, 3H), 2.35 (d, J = 4.4 Hz, 1H).

^{13}C NMR (101 MHz, CDCl$_3$) δ 162.6 (d, J = 247.2 Hz), 144.7, 134.8, 130.8 (d, J = 3.2 Hz), 129.8, 128.2 (d, J = 8.4 Hz), 127.9, 115.5 (d, J = 21.6 Hz), 40.3, 36.0, 21.6.

^{19}F NMR (376 MHz, CDCl$_3$) δ -113.35 – -113.51

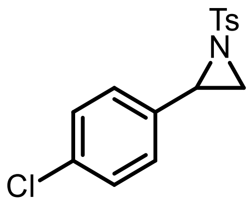

2-(4-chlorophenyl)-1-tosylaziridine(1d) Prepared according to Method A, and the title compound was isolated as a white solid (1.4 g, 44% yield) with spectral data in agreement with literature values.

^{1}H NMR (400 MHz, CDCl$_3$) δ 7.85 (d, *J* = 8.4 Hz, 2H), 7.33 (d, *J* = 8.0 Hz, 2H), 7.27 – 7.25 (m, 2H), 7.15 (d, *J* = 8.4 Hz, 2H), 3.73 (dd, *J* = 7.2, 4.4 Hz, 1H), 2.98 (d, *J* = 7.2 Hz, 1H), 2.44 (s, 3H), 2.34 (d, *J* = 4.4 Hz, 1H).

^{13}C NMR (101 MHz, CDCl$_3$) δ 144.8, 134.8, 134.2, 133.6, 129.8, 128.7, 127.9, 127.9, 40.2, 36.0, 21.6.

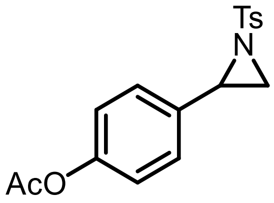

methyl 4-(1-tosylaziridin-2-yl)benzoate(1e) Prepared according to Method A, and the title compound was isolated as a white solid (2.3 g, 88% yield) with spectral data in agreement with literature values.

^{1}H NMR (400 MHz, CDCl$_3$) δ 7.85 (d, *J* = 8.4 Hz, 2H), 7.33 (d, *J* = 8.0 Hz, 2H), 7.22 (d, *J* = 8.4 Hz, 2H), 7.01 (d, *J* = 8.8 Hz, 2H), 3.75 (dd, *J* = 7.2, 4.4 Hz, 1H), 2.97 (d, *J* = 7.2 Hz, 1H), 2.43 (s, 3H), 2.35 (d, *J* = 4.4 Hz, 1H), 2.27 (s, 3H).

^{13}C NMR (101 MHz, CDCl$_3$) δ 169.4, 150.6, 144.8, 134.8, 132.6, 129.8, 127.9, 127.7, 121.8, 40.5, 36.0, 21.6, 21.0.

2.2. Synthetic Application and Characterization of Products

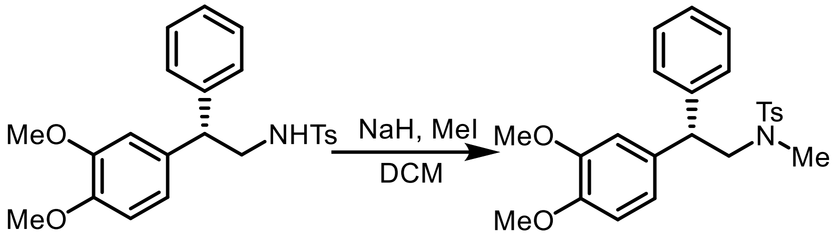

To a solution of the allylic amine **3v** (90 mg, 0.22 mmol, 1.0 equiv.) in DCM (5 mL) was added NaH (26.4 mg, 1.1 mmol, 5 equiv.) and MeI (68 uL, 1.1 mmol, 5 equiv.). The reaction mixture was warmed to 45 ºC and the conversion was followed by TLC. After cooling down to rt, the reaction was quenched at 0 ºC with water and the aqueous solution was extracted with DCM (3x 20 mL). The solvent was removed under reduced pressure and the residue was purified by column chromatography (hexanes/EtOAc, 5:1, v/v) The residue was purified by flash column chromatography (PE:EA:TEA = 50:10:1) to give **3va** (85 mg, 91% ee) in 94% yield as a white solid.

^{1}H NMR (400 MHz, CDCl$_3$) δ 7.62 (d, *J* = 8.0 Hz, 2H), 7.37 – 7.20 (m, 7H), 6.85 (m, 3H), 4.26 (t, *J* = 8.0 Hz, 1H), 3.88 (m, 6H), 3.68 (m , 1H), 3.51 (m, 1H), 2.60 (s, 3H), 2.43 (s, 3H).

^{13}C NMR (101 MHz, CDCl$_3$) δ 149.0, 147.9, 143.3, 142.0, 134.5, 133.9, 129.7, 128.7, 128.1, 127.5, 126.8, 120.3, 111.8, 111.2, 56.0, 55.9, 54.7, 49.5, 35.3, 21.5.

HPLC: Chiralpak OD-H, 15% IPA in hexanes, 30 min run, 1 mL/min.

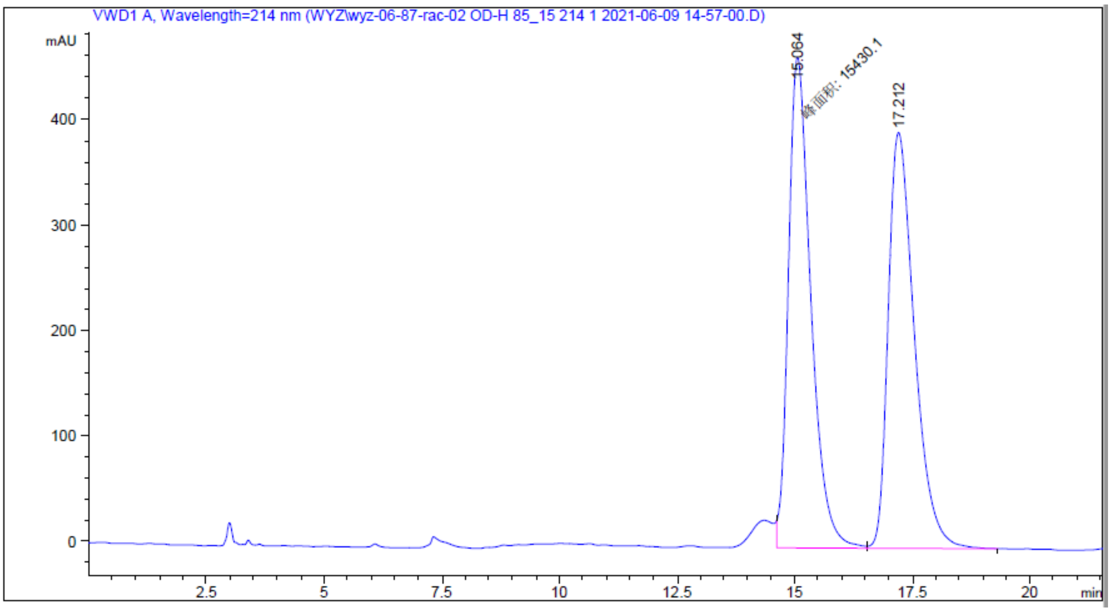

Peak NO	Ret. Time(min)	Area/%
1	15.064	50.3251
2	17.212	49.6749

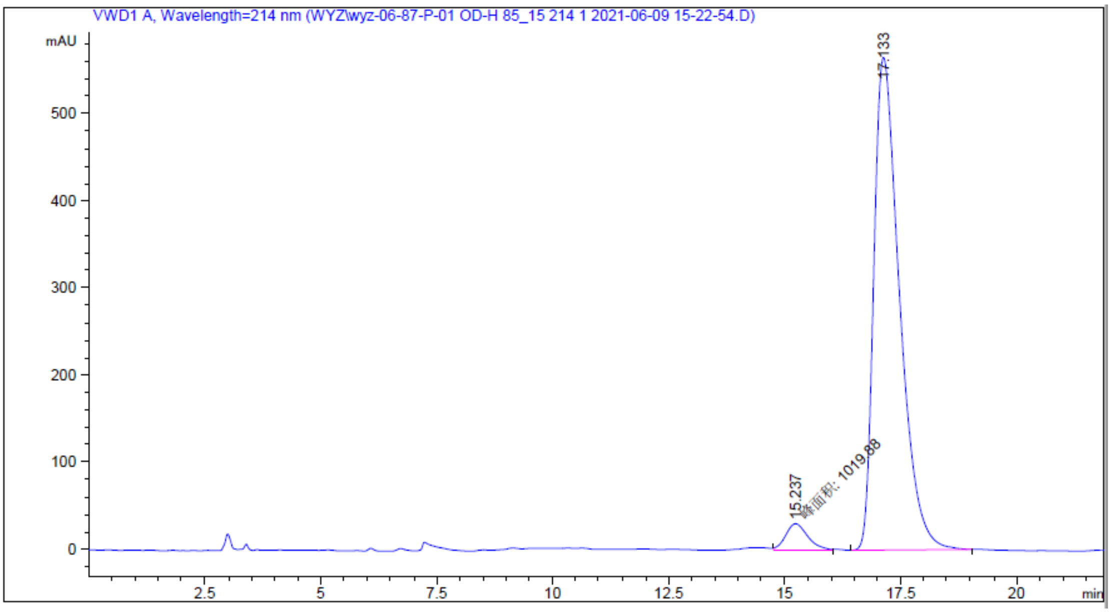

Peak NO	Ret. Time(min)	Area/%
1	15.237	4.4799
2	17.133	95.5201

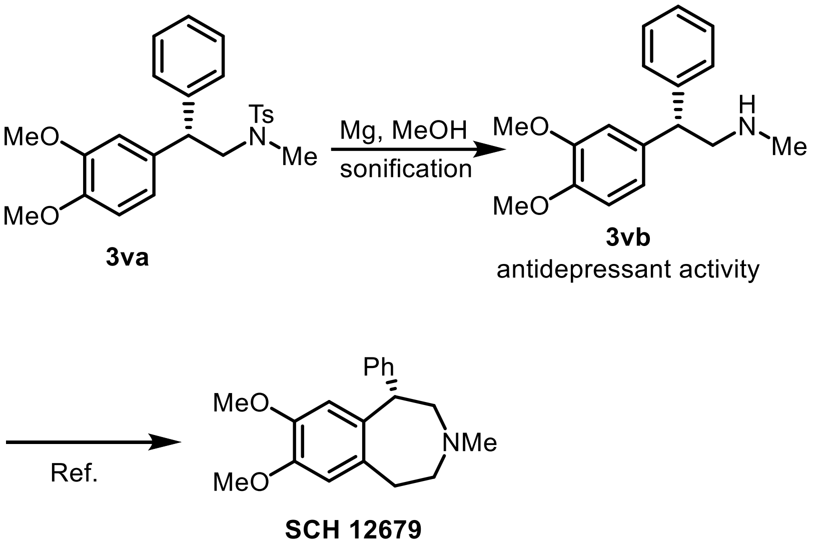

dopamine D2 receptors

In 10 mL sealed tube, **3va** (50 mg, 0.12 mmol, 1.0 equiv.) was added to a mixture of Mg powder (146 mg, 6.1 mmol, 50 equiv.) in MeOH (3 mL) under the atmosphere of Ar. After sonication for 40 minutes, the reaction mixture turned to be gel-like and the Mg powder was dissolved. The organic solvent was removed under reduced pressure.

The residue was filtered through a pad of Celite. The filtrate was evaporated and filtered again with EA/MeOH (10:1). After removal of the solvent under reduced pressure, the residue was purified by flash column chromatography on silica gel with gradient petroleum ether/ethyl acetate/methanol/triethylamine (10:10:1:1) to afford **3vb** as light yellow oil (22 mg, 94% yield, 91% ee). **3vb** can be converted to bioactive **SCH 12679** based on a previous report.[5]

^{1}H NMR (400 MHz, $CDCl_3$) δ 7.35 – 7.22 (m, 4H), 7.19 (t, *J* = 6.8 Hz, 1H), 6.81 (s, 2H), 6.75 (s, 1H), 4.13 (t, *J* = 7.2 Hz, 1H), 3.86 – 3.79 (m, 6H), 3.17 (s, 2H), 2.44 (s, 3H), 1.50 (s, 1H).

^{13}C NMR (101 MHz, $CDCl_3$) δ 149.0, 147.7, 143.2, 135.5, 128.6, 127.9, 126.5, 119.7, 111.6, 111.3, 56.9, 55.9, 55.9, 50.7, 36.5.

HRMS (ESI) calcd for $C_{17}H_{22}N_1O_2$ $[M+H]^+$: 272.16476; found: 272.16451.

Optical Rotation: $[\alpha]_D^{29}$ -1.12 (c 0.4, $CHCl_3$)

HPLC: Chiralpak IC, Hex/EtOH/Diethylamine = 80/20/0.05, 20 min run, 0.8 mL/min.

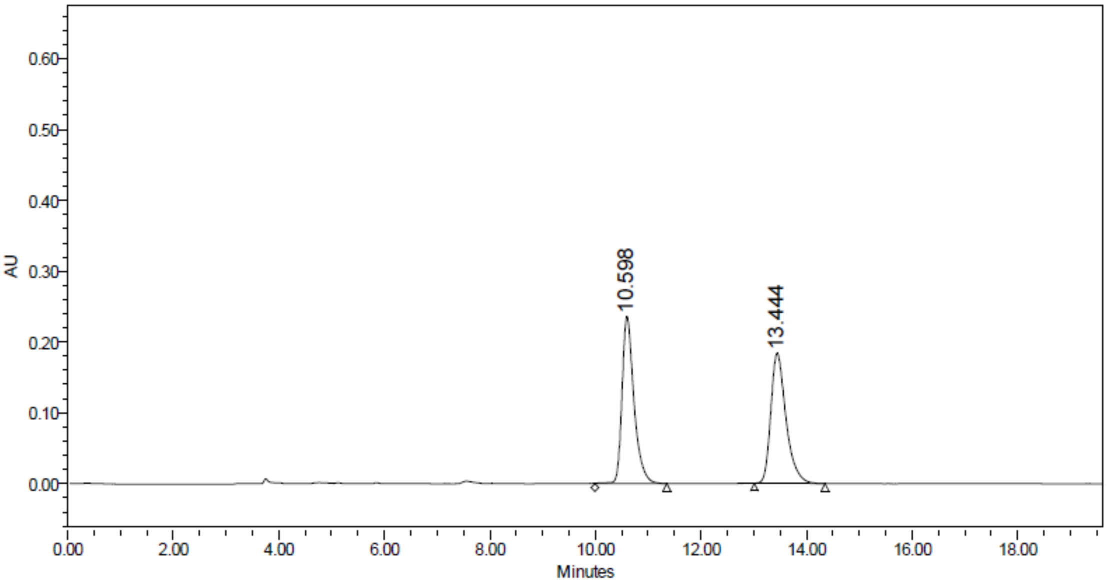

Peak NO	Ret. Time(min)	Area/%
1	10.598	50.34
2	13.444	49.66

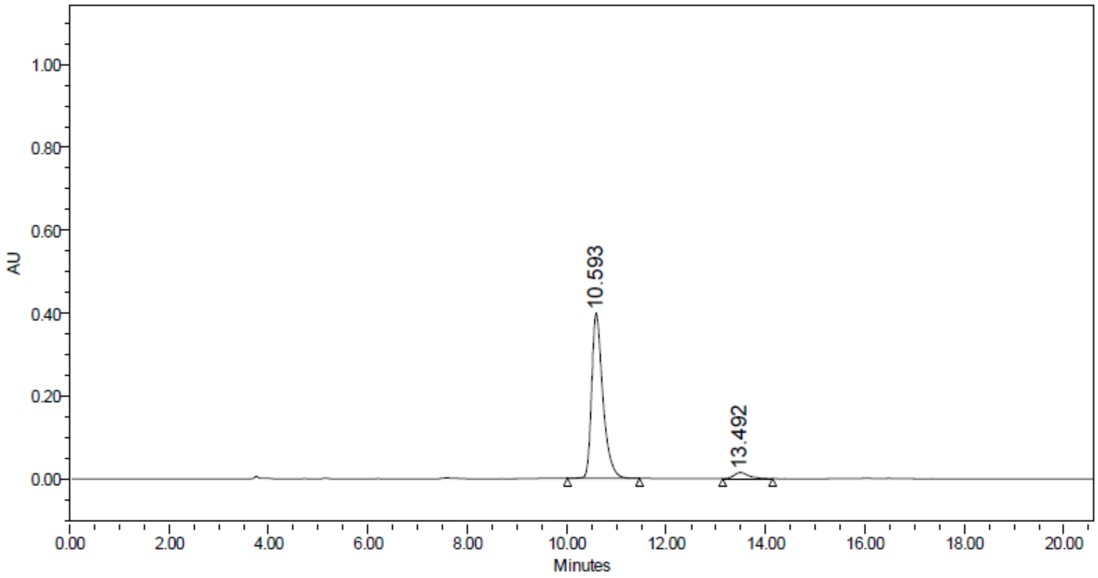

Peak NO	Ret. Time(min)	Area/%
1	10.593	95.39
2	13.492	4.61

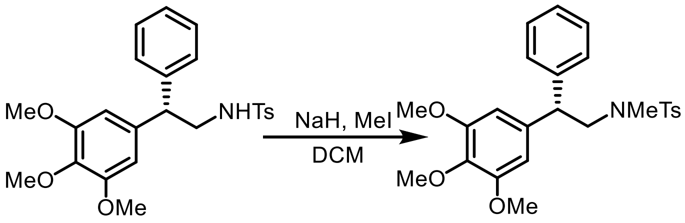

3w **3wa**

To a solution of the allylic amine **3w** (60 mg, 0.136 mmol, 1.0 equiv.) in DCM (3 mL) was added NaH (16.3 mg, 0.68 mmol, 5 equiv.) and MeI (43 uL, 0.68 mmol, 5 equiv.). The reaction mixture was warmed to 45 ºC and the conversion followed by TLC. After cooling down to rt, the reaction was quenched at 0 ºC with water and the aqueous solution was extracted with DCM (3x 20 mL). The solvent was removed under reduced pressure and the residue was purified by column chromatography (hexanes/EtOAc, 5:1, v/v) The residue was purified by flash column chromatography (PE:EA:TEA = 50:10:1) to give **3wa** (45.3 mg, 0.1 mmol) in 73% yield as a white oil.

^{1}H NMR (400 MHz, $CDCl_3$) δ 7.62 (d, *J* = 8.4 Hz, 2H), 7.38 – 7.21 (m, 7H), 6.57 (s, 2H), 4.24 (dd, *J* = 8.4, 7.6 Hz, 1H), 3.85 (m, 9H), 3.76 (m, 1H), 3.41 (m, 1H), 2.62 (s, 3H), 2.42 (s, 3H).

^{13}C NMR (101 MHz, $CDCl_3$) δ 153.2, 143.4, 141.7, 136.8, 134.3, 129.7, 128.7, 128.1,

127.4, 127.0, 105.5, 60.9, 56.2, 54.5, 50.0, 35.2, 21.5.

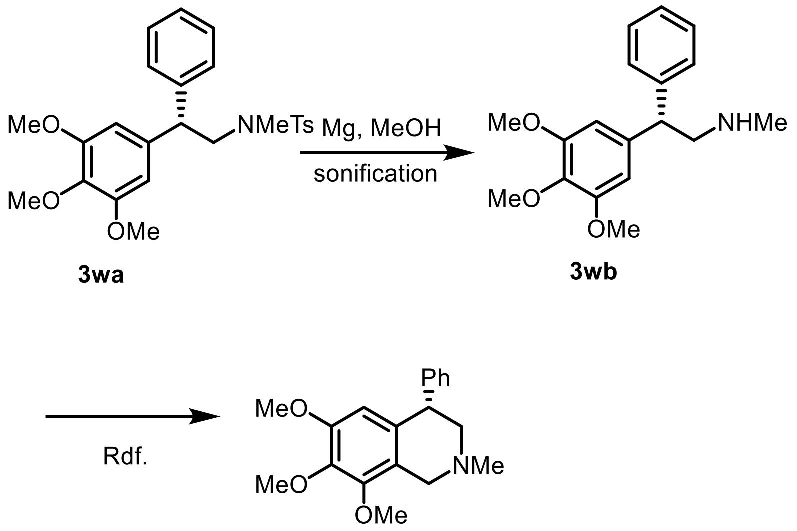

In a 10 mL sealed tube, compound **3wa** (45.3 mg, 0.1 mmol, 1.0 equiv.) was added to a mixture of Mg powder (120 mg, 5.0 mmol, 50 equiv.) in MeOH (5 mL) under the atmosphere of Ar. After sonication for 40 minutes, the reaction mixture turned to be gel-like and the Mg powder was dissolved. The organic solvent was removed under reduced pressure. The residue was filtered through a pad of Celite. The filtrate was evaporated and filtered again with EA/MeOH (10:1). After removal of the solvent under reduced pressure, the residue was purified by flash column chromatography on silica gel with gradient petroleum ether/ethyl acetate/methanol/triethylamine (10:10:1:1) to afford **3wb** as yellow solid (27.3 mg, 91% yield, 92% ee). Finally, enantiopure tetrahydroisoquinoline, as a privileged skeleton, often exists in bioactive molecules and pharmaceuticals. **3wb** could undergo condensation with formaldehyde to generate chiral tetrahydroisoquinoline derivates.[6]

^{1}H NMR (400 MHz, $CDCl_3$) δ 7.36 – 7.17 (m, 5H), 6.47 (s, 2H), 4.15 (t, J = 7.6 Hz, 1H), 3.81 (m, 9H), 3.19 (d, J = 7.6 Hz, 2H), 2.92 (s, 1H), 2.44 (s, 3H).

^{13}C NMR (101 MHz, $CDCl_3$) δ 153.3, 142.5, 138.3, 136.7, 128.7, 127.8, 126.7, 105.0, 60.8, 56.4, 56.1, 51.0, 36.1.

HRMS (ESI) calcd for $C_{18}H_{24}N_1O_3$ [M+H]+: 302.17589; found: 302.17507.

Optical Rotation: $[\alpha]_D^{29}$ -2.76 (c 0.3, $CHCl_3$)

HPLC: Chiralpak IC, Hex/EtOH/Diethylamine = 95/5/0.05, 40 min run, 0.7 mL/min.

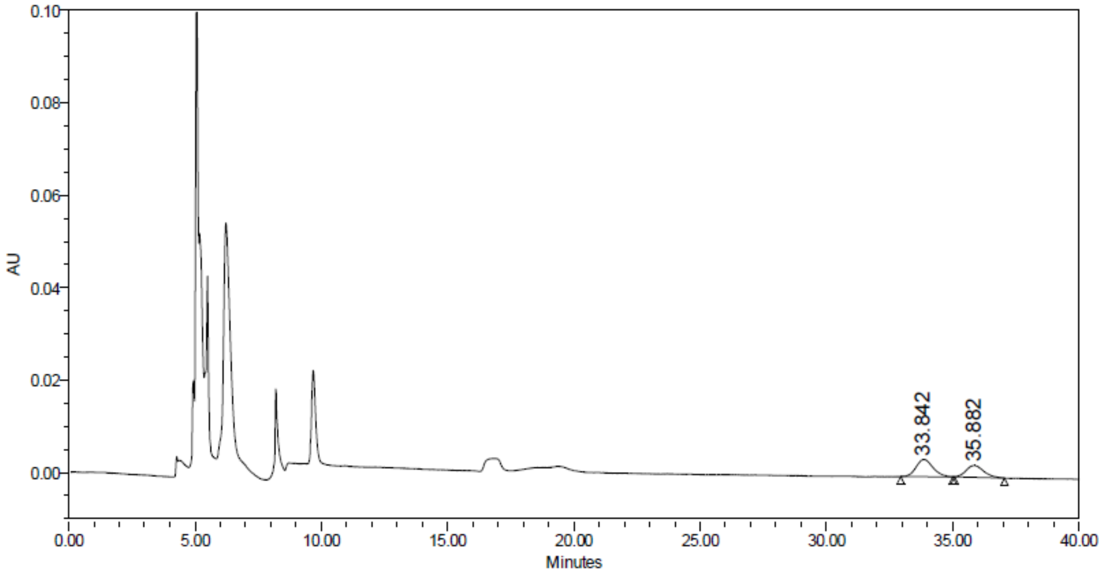

Peak NO	Ret. Time(min)	Area/%
1	33.842	58.55
2	35.882	41.45

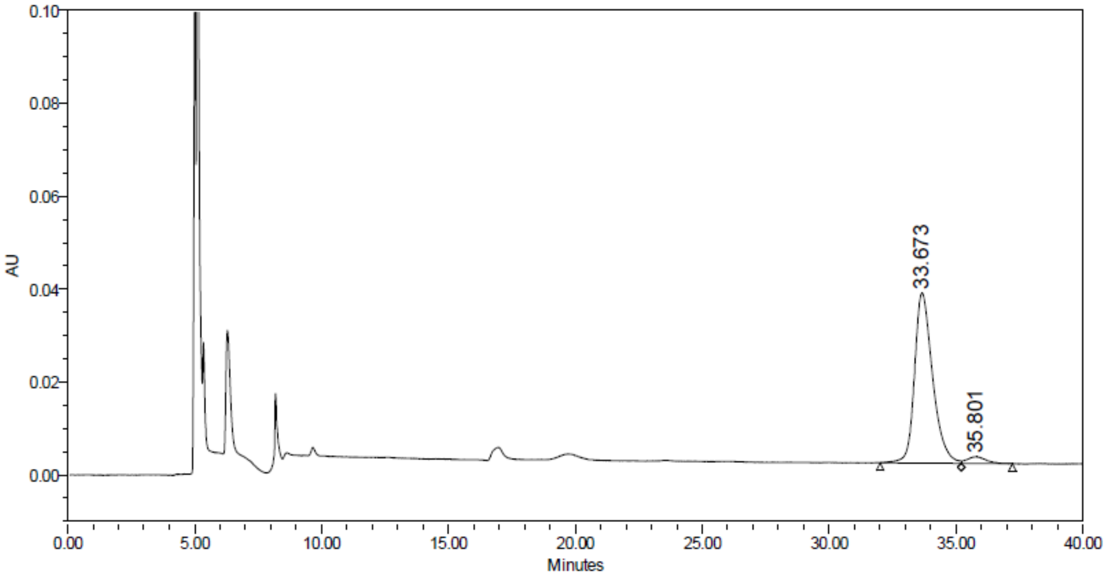

Peak NO	Ret. Time(min)	Area/%
1	33.673	96.18
2	35.801	3.82

2.3. Cyclic Voltammetry Studies

All the voltammetric experiments were recorded with a CHI660E potentiostat at room temperature in DMAc. $^{n}Bu_4NPF_6$(0.1 M) was used as the supporting electrolyte, a Glass Carbon electrode and a platinum wire were used as working and counter electrodes, respectively. The working electrode potentials were measured versus Ag/$AgNO_3$ reference electrode (internal solution, 0.1 M $AgNO_3$in DMAc). The redox potential of ferrocene/ferrocenium (Fc/Fc^+) was measured (same experimental conditions) and used to provide an internal reference. The potential values were then adjusted relative to Fc/Fc^+, and electrochemical studies in organic solvents were recorded accordingly. The scan rate was 0.1V s^{-1}.

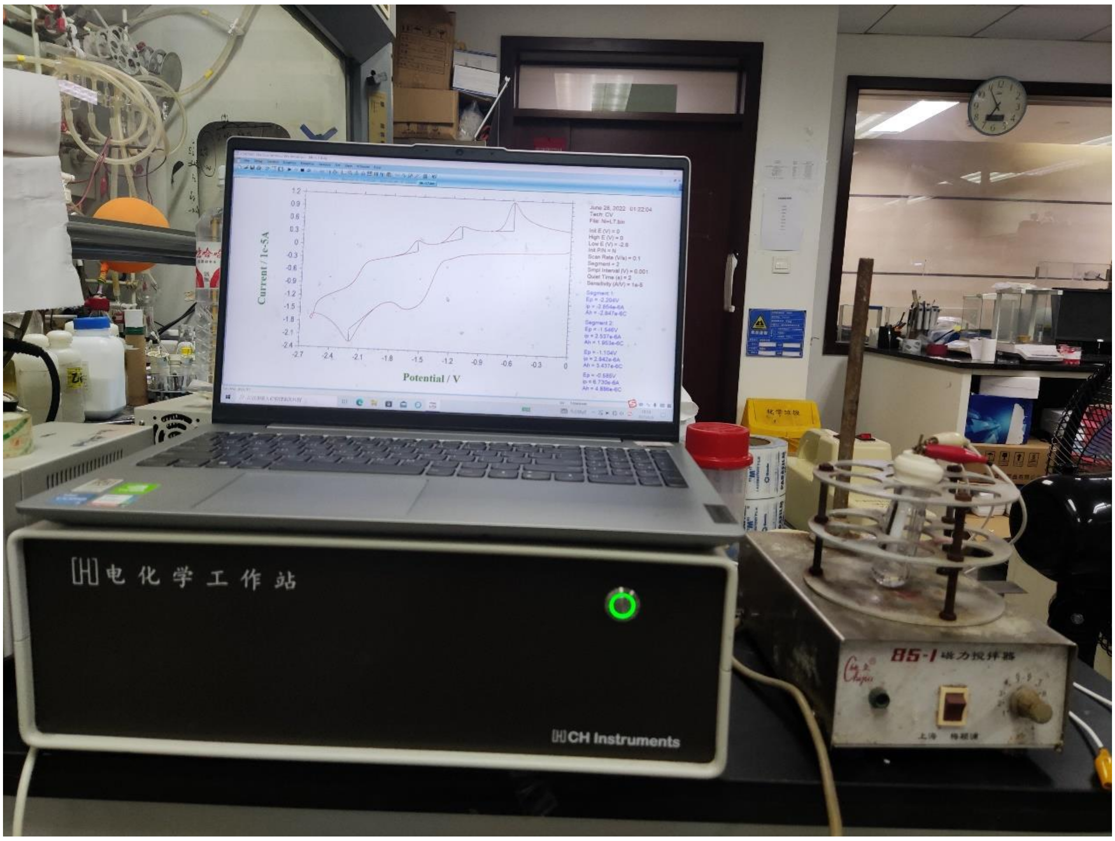

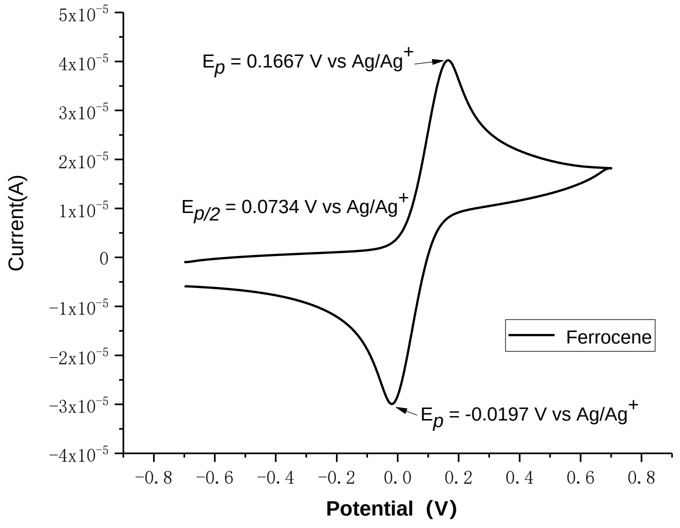

Supplementary Fig. 1 Cyclic voltammograms of 5 mM Ferrocene, DMAc solvent, 0.1 M $^nBu_4NPF_6$ supporting electrolyte, GC working electrode, 100 mV/s scan rate.

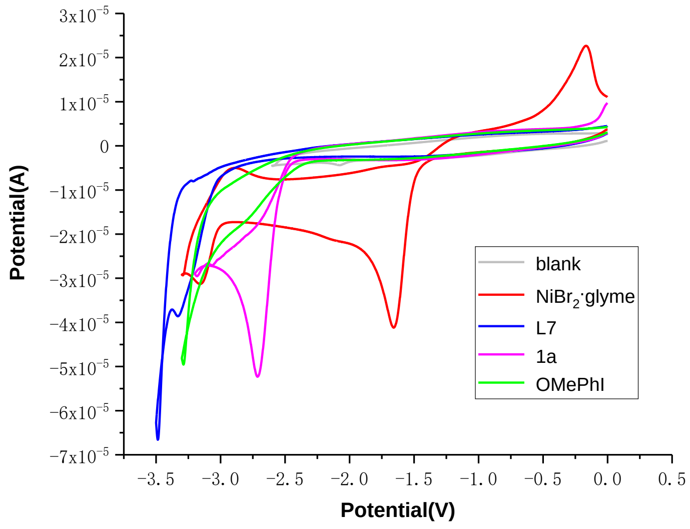

Supplementary Fig. 2 Cyclic voltammograms of blank, 5 mM $NiBr_2$·glyme (red line), 5 mM **L7** (blue line), 5 mM **1a** (carmine line), 5 mM OMePhI (green line), DMAc solvent, 0.1M $^nBu_4NPF_6$ supporting electrolyte, GC working electrode, 100 mV/s scan rate.

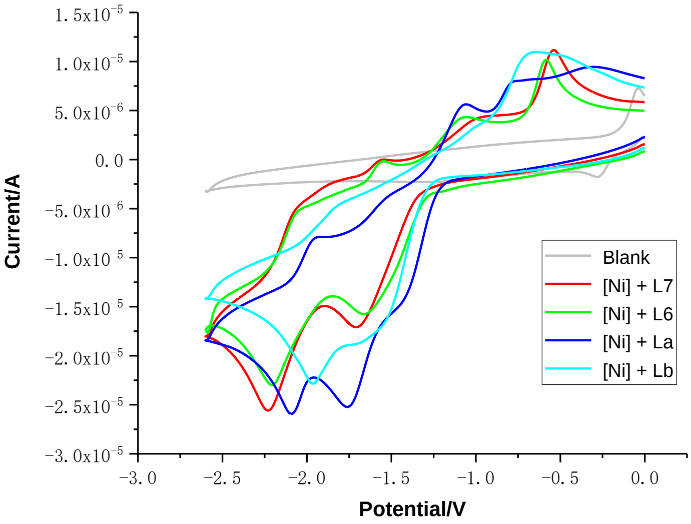

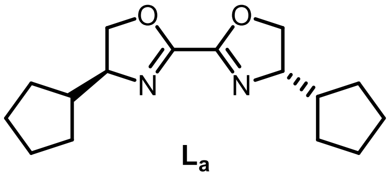

L_a

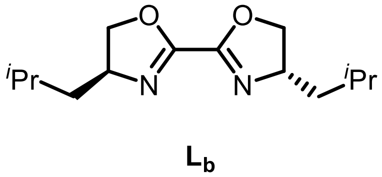

L_b

Supplementary Fig. 3 Cyclic voltammograms of blank, 5 mM $NiBr_2$·glyme + 5 mM **L7** (red line), 5 mM $NiBr_2$·glyme + 5 mM **L6** (green line), 5 mM $NiBr_2$·glyme + 5 mM **La** (blue line), 5 mM $NiBr_2$·glyme + 5 mM **L7** (cyan line), DMAc solvent, 0.1 M $^{n}Bu_4NPF_6$ supporting electrolyte, GC working electrode, 100 mV/s scan rate.

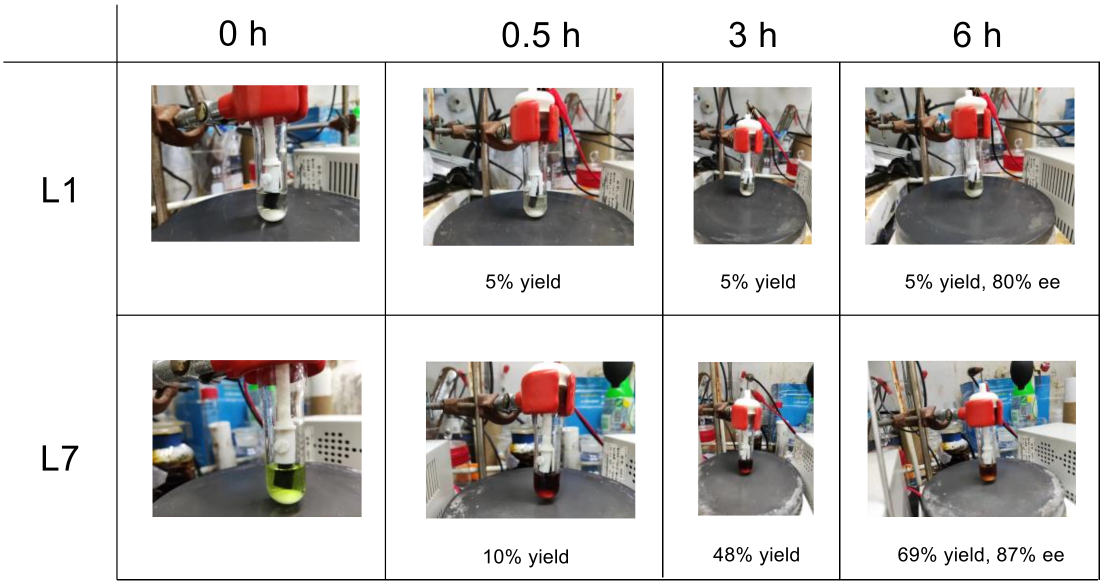

Supplementary Fig. 4 Solution color of $NiBr_2$•glyme with **L1** or **L7**

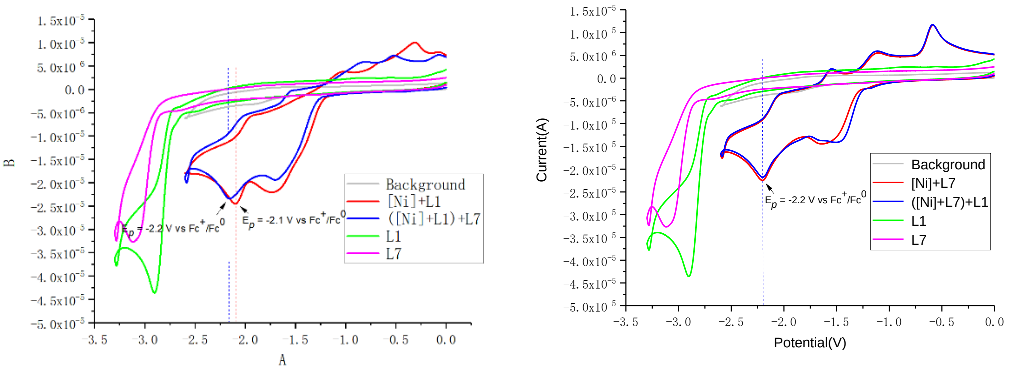

Supplementary Fig. 5. CV analysis for ligand exchange

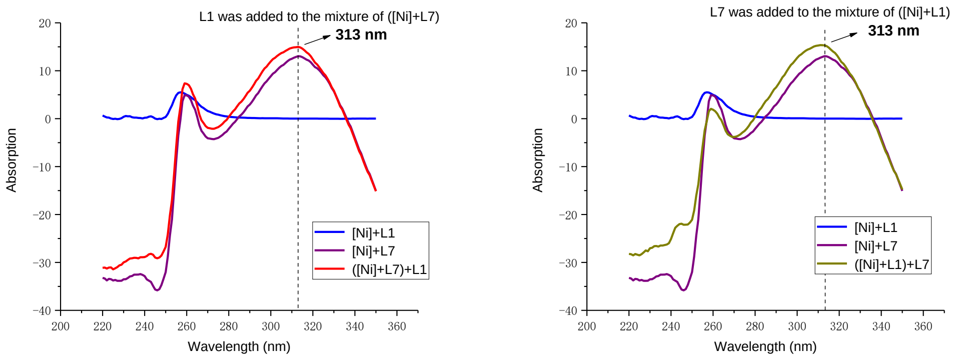

Supplementary Fig. 6. UV-Vis absorption spectroscopy analysis of ligand exchange

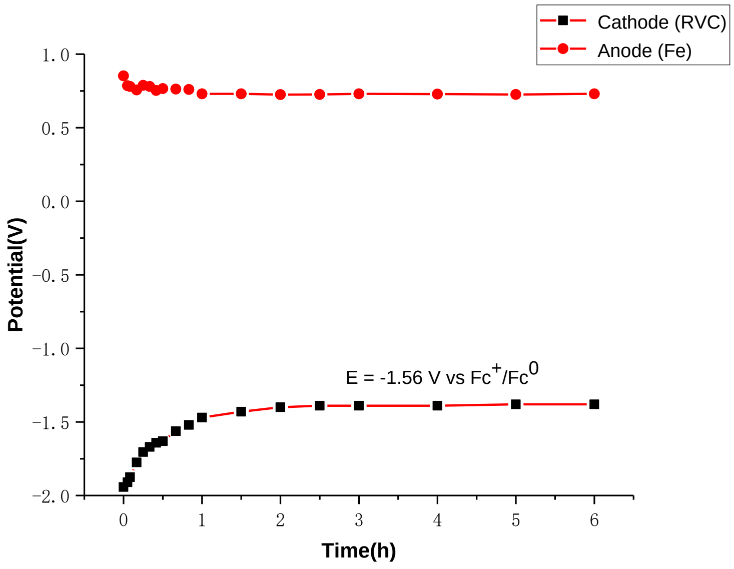

Supplementary Fig. 7. Potential profiles of RVC cathode and Fe anode during the electrolysis.

The cathode potential was around -1.56 V *vs* Fc/Fc^+

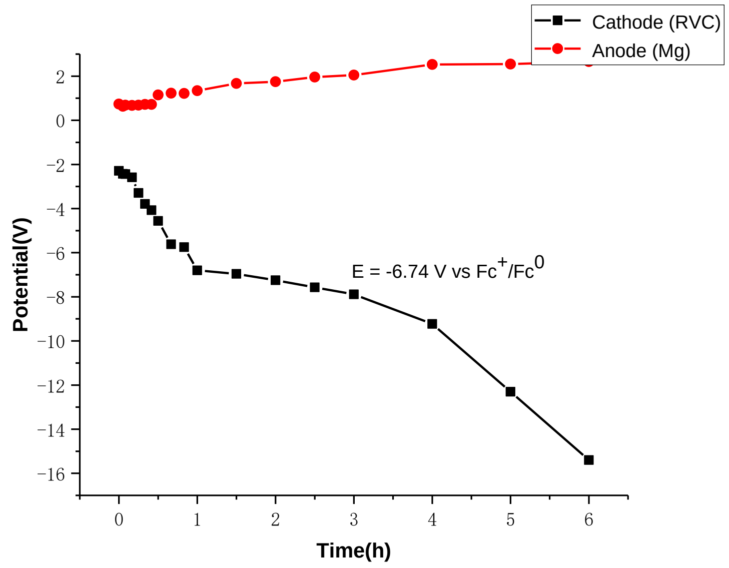

Supplementary Fig.8 Potential profiles of RVC cathode and Mg anode during the electrolysis.

The cathode potential was around -6.74 V *vs* Fc/Fc$^+$

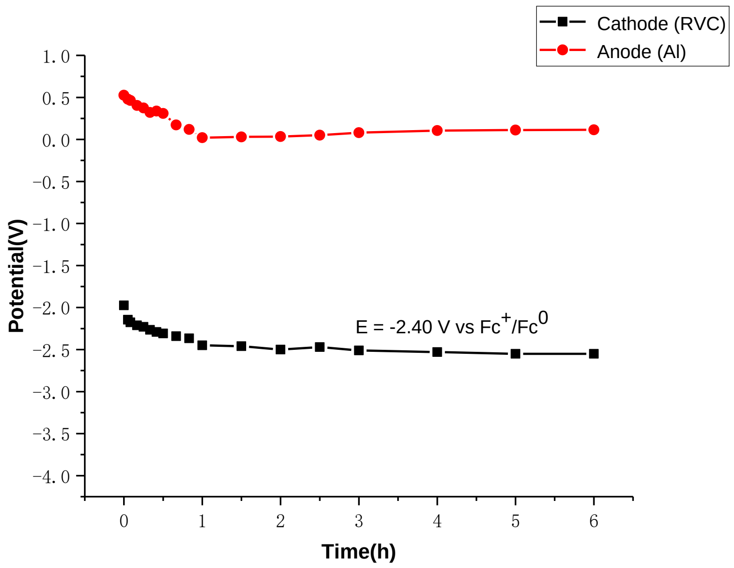

Supplementary Fig. 9 Potential profiles of RVC cathode and Al anode during the electrolysis.

The cathode potential was around -2.4 V *vs* Fc/Fc$^+$

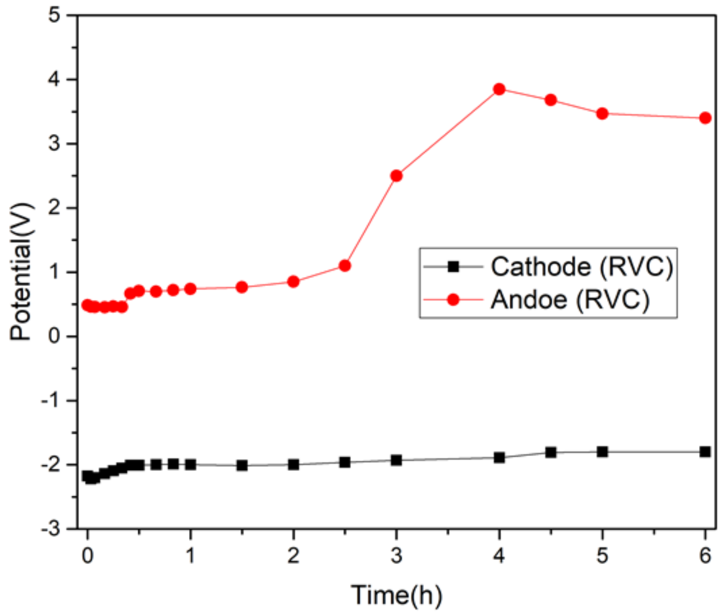

Supplementary Fig. 10 Potential profiles of RVC cathode and RVC anode during the electrolysis.

The cathode potential was around -2.0 V *vs* Fc/Fc$^+$

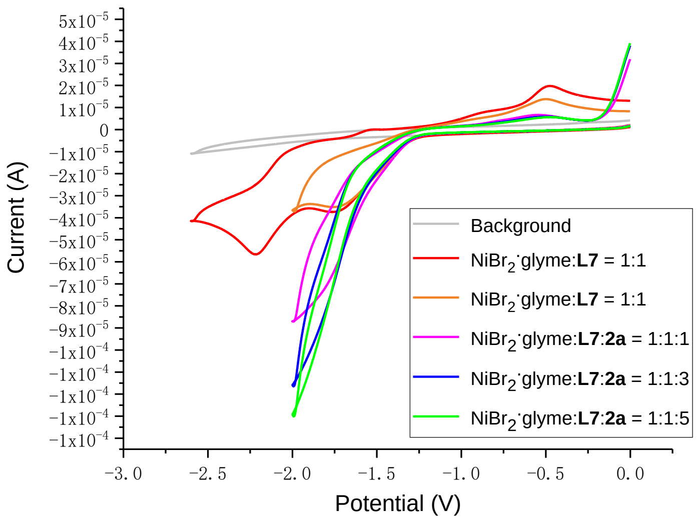

Supplementary Fig. 11 Cyclic voltammograms of blank, 5 mM $NiBr_2$•glyme + 5 mM **L7** (red line and orange line), 5 mM $NiBr_2$•glyme + 5 mM **L7** + 5 mM **2a** (purple line), 5 mM $NiBr_2$•glyme + 5 mM **L7** + 15 mM **2a** (blue line), 5 mM $NiBr_2$•glyme + 5 mM **L7** + 25 mM **2a** (green line), DMAc solvent, 0.1 M $^{n}Bu_4NPF_6$ supporting electrolyte, GC working electrode, 100 mV/s scan rate.

2.4 Optimization Details

Supplementary Table 1. Screening of Ligand for pyridine bisoxazoline, pyridine oxazoline, bisoxazoline and carbazole.

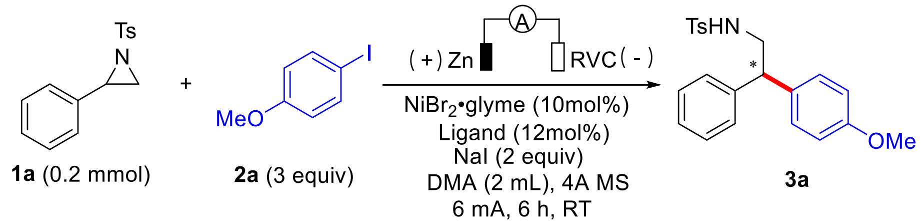

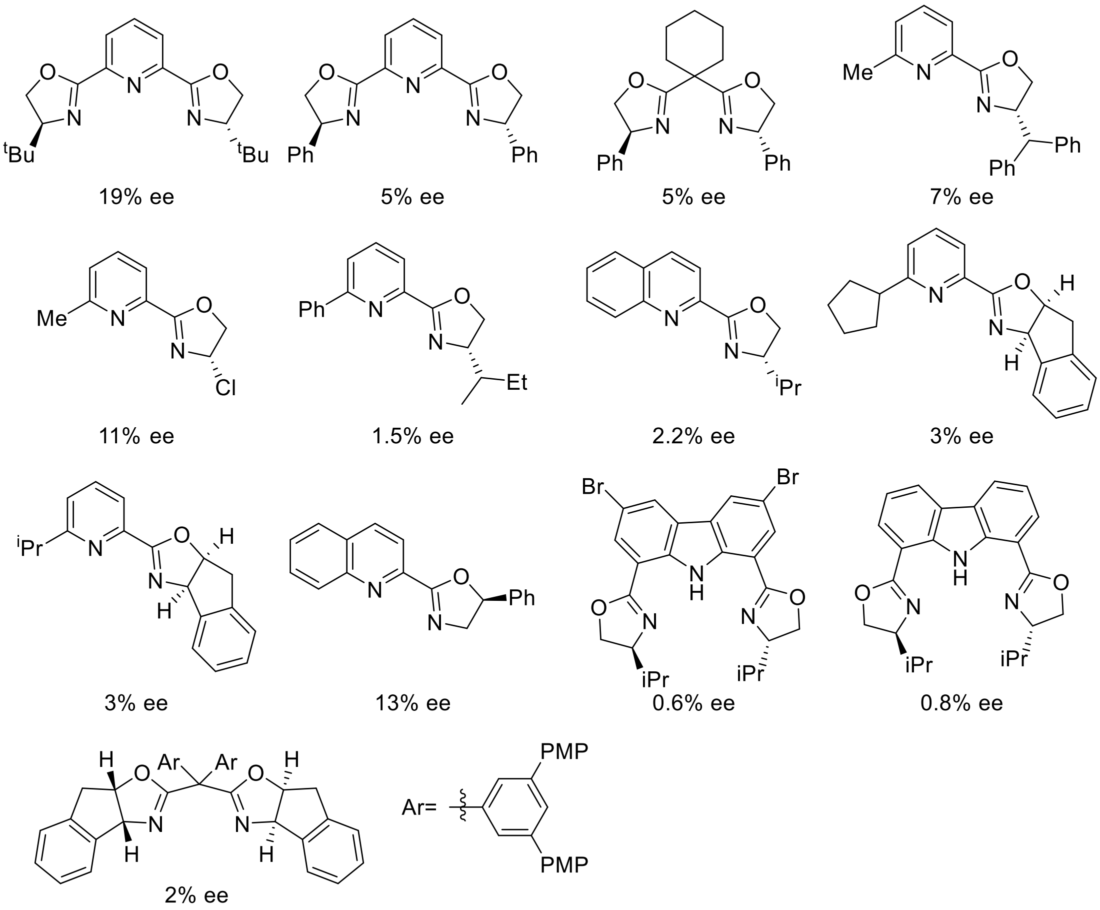

Reaction conditions: **1a** (0.2 mmol), **2a** (3.0 equiv.), $NiBr_2$•glyme (10 mol%), **L** (12 mol%), NaI (2 equiv.), DMAc (2 mL), in an undivided cell subject to 6 mA of current for 6 h using Zinc (1.0 x 1.5 cm^2) and RVC (1.0 x 1.5 x 0.3 cm^3) electrodes.

Supplementary Table 2. Screening of Ligand for bioxazoline.

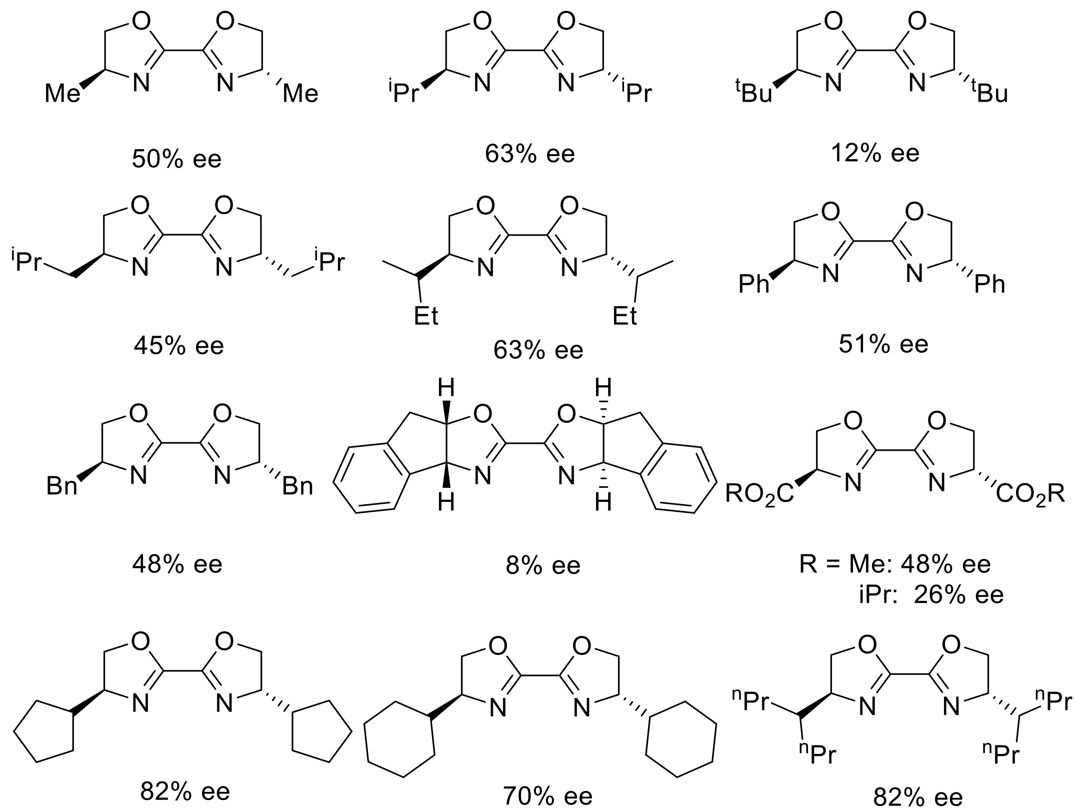

Reaction conditions: **1a** (0.2 mmol), **2a** (3.0 equiv.), $NiBr_2$•glyme (10 mol%), **L** (12 mol%), NaI (2 equiv.), DMAc (2 mL), in an undivided cell subject to 6 mA of current for 6 h using Zinc (1.0 x 1.5 cm^2) and RVC (1.0 x 1.5 x 0.3 cm^3) electrodes.

Supplementary Table 3. Screening of Ligand for bimidazoline.

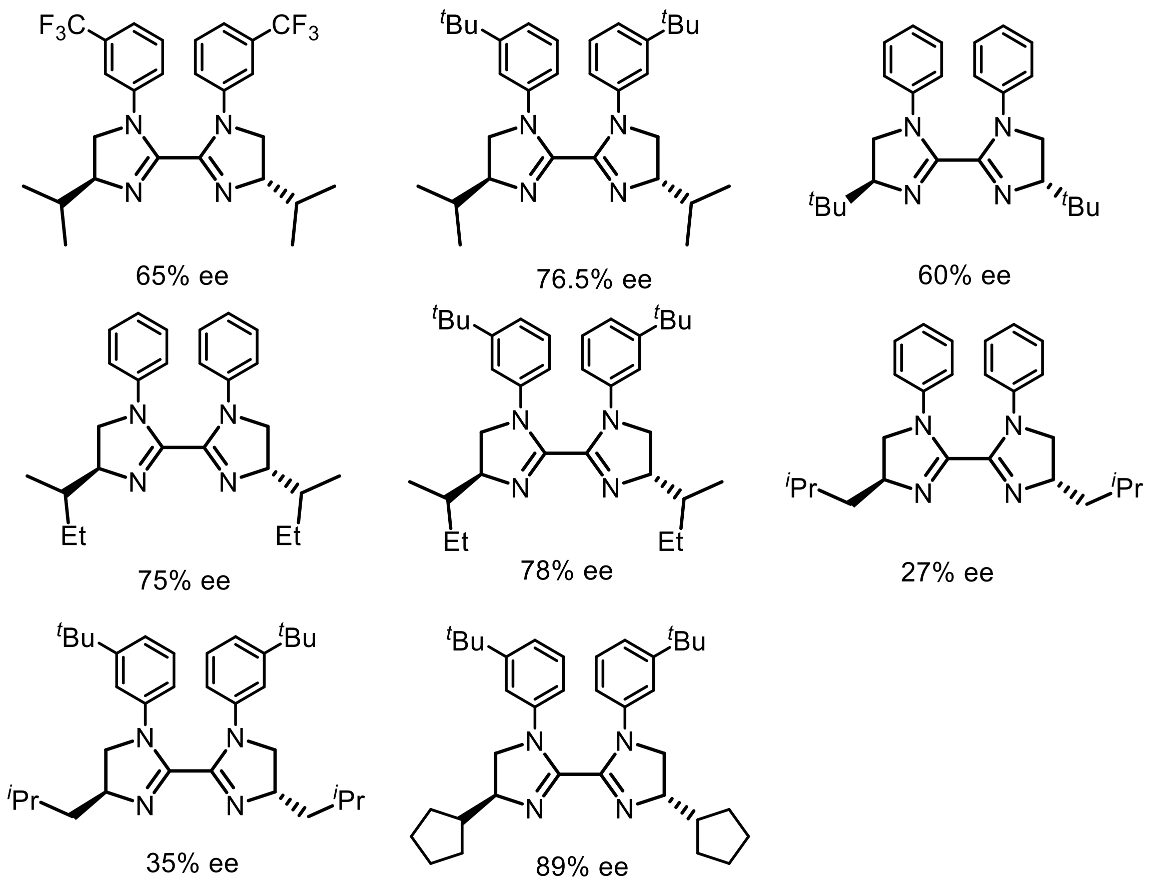

Reaction conditions: **1a** (0.2 mmol), **2a** (3.0 equiv.), NiBr$_2$•glyme (10 mol%), **L** (12 mol%), Et$_3$N (2 equiv.), NaI (2 equiv.), DMAc (2 mL), in an undivided cell subject to 6 mA of current for 6 h using Zinc (1.0 x 1.5 cm^2) and RVC (1.0 x 1.5 x 0.3 cm^3) electrodes.

Supplementary Table 4. Screening of additive.[a,b,c]

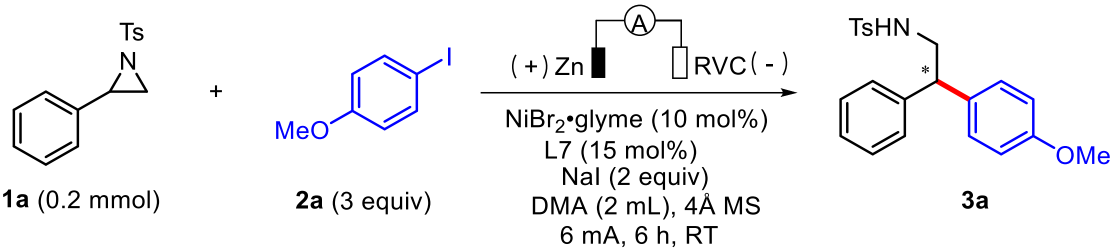

entry	variation from standard conditions[a]	yields(%)[b]	ee(%)[c]
1	TMSCl (0.5equiv)	22	85
2	lutidine (2 equiv)	0	/
**3**	**Et_3N (2 equiv)**	**30**	**88**

[a]Reaction conditions: **1a** (0.2 mmol), **2a** (3.0 equiv.), $NiBr_2$•glyme (10 mol%), **L7** (15 mol%), NaI (2 equiv.), DMAc (2 mL), in an undivided cell subject to 6 mA of current for 6 h using Zinc (1.0 x 1.5 cm^2) and RVC (1.0 x 1.5 x 0.3 cm^3) electrodes. [b]Yields were determined by ^1H NMR using CH_2Br_2 as an internal standard. [c]Enantioselectivities were determined by chiral HPLC analysis.

Supplementary Table 5. Screening of current.[a,b,c]

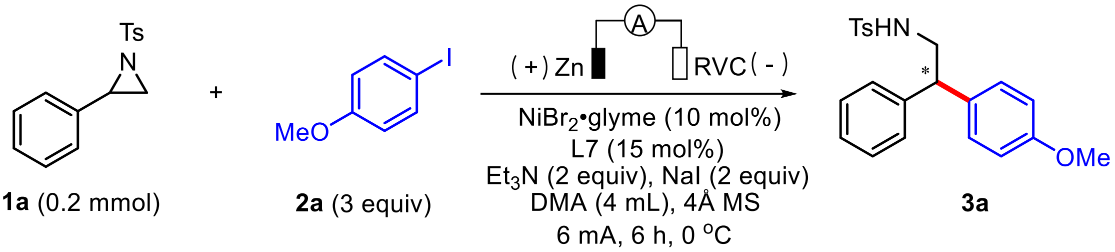

entry	variation from standard conditions[a]	yields(%)[b]	ee(%)[c]
1	none	0	/
2	1.5 mA, 24 h	16.6	89.3
**3**	**2 mA, 18 h**	**40**	**90**
4	3 mA, 12 h	29	90
5	4 mA, 9 h	0	/
6	10 mA, 3 h	0	/

[a]Reaction conditions: **1a** (0.2 mmol), **2a** (3.0 equiv.), $NiBr_2$•glyme (10 mol%), **L7** (15 mol%), Et_3N (2 equiv.), NaI (2 equiv.), DMAc (2 mL), in an undivided cell subject to 6 mA of current for 6 h using Zinc (1.0 x 1.5 cm^2) and RVC (1.0 x 1.5 x 0.3 cm^3) electrodes. [b]Yields were determined by ^1H NMR using CH_2Br_2 as an internal standard. [c]Enantioselectivities were determined by chiral HPLC analysis.

Supplementary Table 6. Screening of electrolyte.[a,b,c]

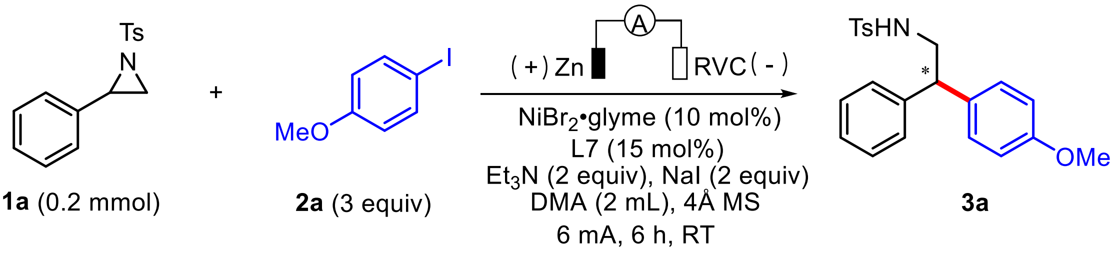

1a (0.2 mmol) **2a** (3 equiv) **3a**

entry	variation from standard conditions[a]	yields(%)[b]	ee(%)[c]
1	$^{n}Bu_4NBF_4$	19	81
2	$^{n}Bu_4NPF_6$	15	84
**3**	$^{n}Bu_4NI$	29	86
4	$^{n}Bu_4NBr$	24	86

[a]Reaction conditions: **1a** (0.2 mmol), **2a** (3.0 equiv.), $NiBr_2$•glyme (10 mol%), **L7** (15 mol%), Et_3N (2 equiv.), NaI (2 equiv.), DMAc (2 mL), in an undivided cell subject to 6 mA of current for 6 h using Zinc (1.0 x 1.5 cm^2) and RVC (1.0 x 1.5 x 0.3 cm^3) electrodes. [b]Yields were determined by ^1H NMR using CH_2Br_2 as an internal standard. [c]Enantioselectivities were determined by chiral HPLC analysis.

Supplementary Table 7. Screening of electrode.[a,b,c]

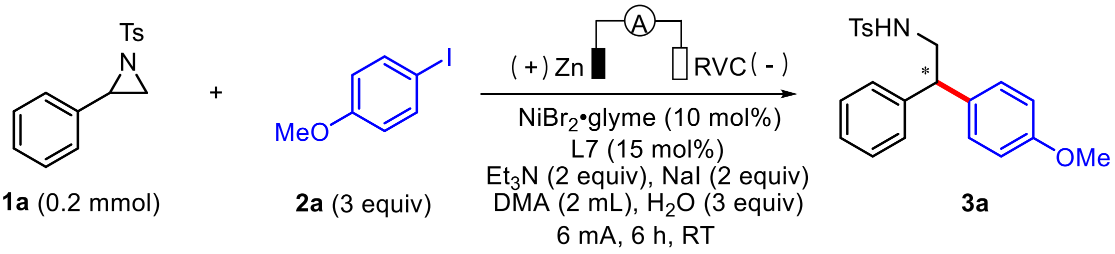

1a (0.2 mmol) **2a** (3 equiv) **3a**

entry	variation from standard conditions[a]	yields(%)[b]	ee(%)[c]
1	Fe (anode)	0	/
2	Mg (anode)	0	/
3	Al (anode)	44	89
4	RVC (anode)	34	90
5	Pt (anode)	0	/
6	C (anode)	0	/
7	Ni foam (cathode)	34	88

[a]Reaction conditions: **1a** (0.2 mmol), **2a** (3.0 equiv.), $NiBr_2$•glyme (10 mol%), **L7** (15 mol%), Et_3N (2 equiv.), H_2O (3 equiv.), NaI (2 equiv.), DMAc (2 mL), in an undivided cell subject to 6 mA of current for 6 h using Zinc (1.0 x 1.5 cm^2) and RVC (1.0 x 1.5 x 0.3 cm^3) electrodes. [b]Yields were determined by ^1H NMR using CH_2Br_2 as an internal standard. [c]Enantioselectivities were determined by chiral HPLC analysis.

Supplementary Table 8. Screening of Proton source.[a,b,c]

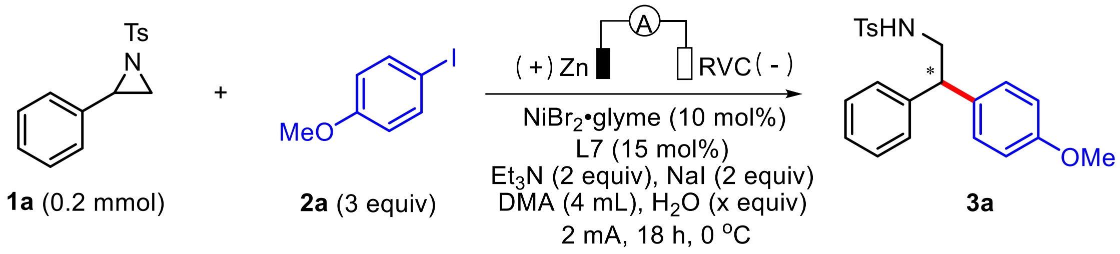

entry	variation from standard conditions[a]	yields(%)[b]	ee(%)[c]
1	H_2O (0.5 equiv)	57	91
2	H_2O (1 equiv)	78	90
**3**	**H_2O (3 equiv)**	**98**	**90**
4	H_2O (5 equiv)	97	90
5	H_2O (50 equiv)	0	/
6	H_2O (100 equiv)	0	/
7	MeOH (3 equiv)	61	88

[a]Reaction conditions: **1a** (0.2 mmol), **2a** (3.0 equiv.), $NiBr_2$•glyme (10 mol%), **L7** (15 mol%), Et_3N (2 equiv.), NaI (2 equiv.), DMAc (4 mL), in an undivided cell subject to 2 mA of current for 18 h using Zinc (1.0 x 1.5 cm^2) and RVC (1.0 x 1.5 x 0.3 cm^3) electrodes. [b]Yields were determined by ^1H NMR using CH_2Br_2 as an internal standard. [c]Enantioselectivities were determined by chiral HPLC analysis.

2.5 Unsuccessful Substrates

Method A:

In Glovebox, an oven-dried electrochemical cell with a stir bar was charged with aziridine (0.2 mmol, 1 equiv.) and aryl iodide (0.6 mmol, 3 equiv.), $NiBr_2$·glyme (0.02 mmol, 10 mol%), ligand **L7** (0.03 mmol, 15 mol%), NaI (0.4 mmol, 2 equiv.), triethylamine (0.4 mmol, 2 equiv.), H_2O (0.6 mmol, 3 equiv.), 2 mL of DMAc. The tube was installed with an RVC as the cathode and zinc as a sacrificial anode. The mixture was stirred at room temperature for 30 min. The reaction mixture was electrolyzed under a constant current of 6 mA at room temperature until the complete consumption of the starting materials as monitored by TLC (about 6 hours). The resulting mixture was diluted with EtOAc, and quenched with sat. NH_4Cl solution, and the aqueous layer extracted with EtOAc (2 x 20 mL). The combined organic layers were dried over $MgSO_4$, the filtrate concentrated, and the crude product was purified by automated silica gel column chromatography (EtOAc/hexanes).

Method B:

In Glovebox, an oven-dried electrochemical cell with a stir bar was charged with aziridine (0.2 mmol, 1 equiv.) and aryl iodide (0.6 mmol, 3 equiv.), $NiBr_2$·glyme (0.02 mmol, 10 mol%), ligand **L7** (0.03 mmol, 15 mol%), NaI (0.4 mmol, 2 equiv.), triethylamine (0.4 mmol, 2 equiv.), H_2O (0.6 mmol, 3 equiv.), 4 mL of DMAc. The tube was installed with an RVC as the cathode and zinc as the sacrificial anode. The mixture was stirred at 0 °C for 30 min. The reaction mixture was electrolyzed under a constant current of 2 mA at 0 °C until the complete consumption of the starting materials was monitored by TLC (about 18 hours). The resulting mixture was diluted with EtOA, and quenched with sat. NH_4Cl solution, and the aqueous layer extracted with EtOAc (2 x 20 mL). The combined organic layers were dried over $MgSO_4$, the filtrate was concentrated, and the crude product was purified by automated silica gel column chromatography (EtOAc/hexanes).

Supplementary Table 9. Catalytic Reactions with Different Aryl Halides

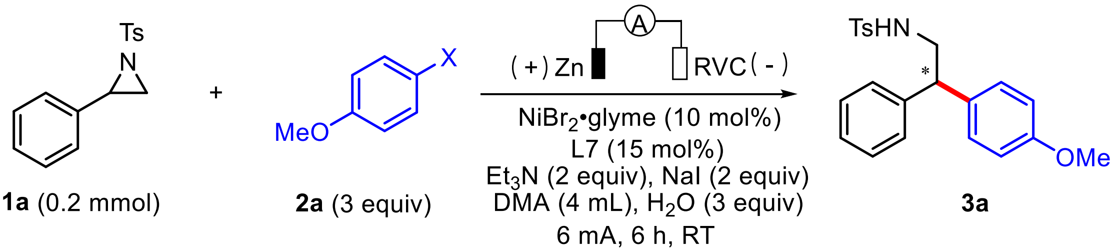

1a (0.2 mmol) **2a** (3 equiv) **3a**

Method A

entry	Ar-X	yield (%)	**1** (%)	**2** (%)	$TsNH_2$ (%)
1	Br	16	0	32	25
2	Cl	2	5.1	61	34
3	OTf	0	7.5	80	27
4	OTs	0	10.3	92	35

Supplementary Table 10. Different Protective Groups

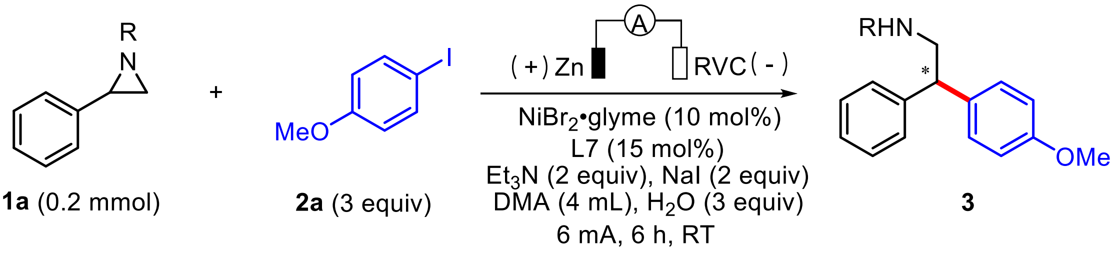

1a (0.2 mmol) **2a** (3 equiv) **3**

Method A

entry	R	conversion (%)	yield (%)
1	NS	46	0
2	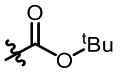	12	0

Supplementary Table 11. Unsuccessful Aziridines and Aryl Iodides

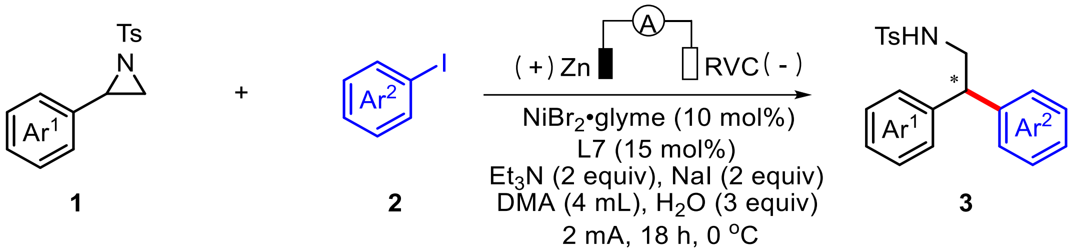

Method B

Ar_1 = Ph, Ar_2 =

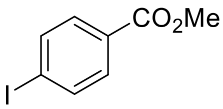

32% yield

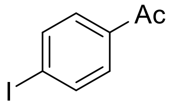

8% yield

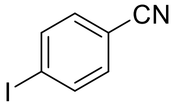

< 5% yield

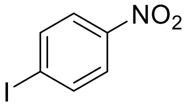

0% yield

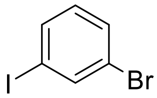

40% yield

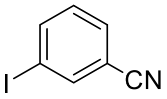

17% yield

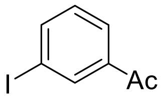

17% yield

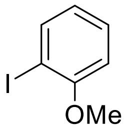

17% yield

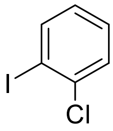

0% yield

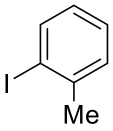

0% yield

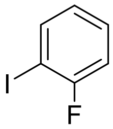

0% yield

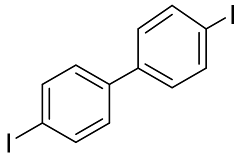

12% yield

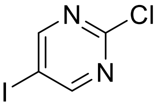

0% yield

Ar_2 = 4-OMePh, Ar1 =

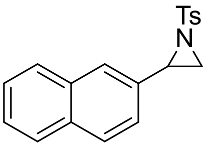

31% yield

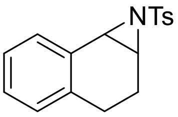

0% yield

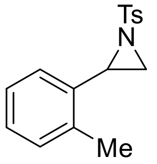

8% yield

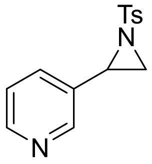

0% yield

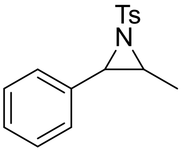

0% yield

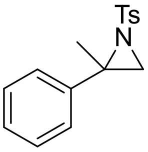

0% yield

Supplementary Table 12. Unsuccessful Alkyl Aziridines

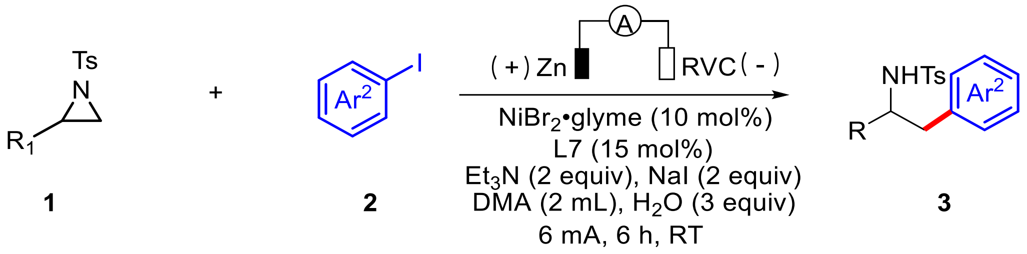

Method A

Ar_2 = 4-OMePh, R =

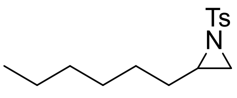	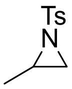	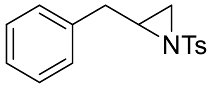
9% yield	15% yield	28% yield

Supplementary Table 13. Unsuccessful Alkyl Halides

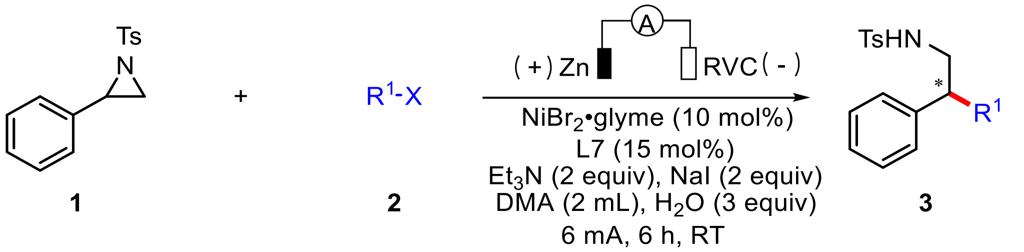

Method A

R^2-X =

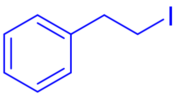	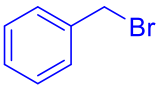	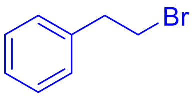	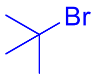	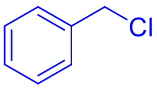
0% yield	0% yield	17% yield, 58% ee	0% yield	0% yield
+	+	+	+	+
$TsNH_2$	$TsNH_2$	$TsNH_2$	$TsNH_2$	$TsNH_2$
35%	46%	<5%	<5%	32%

Supplementary Table 14. Screening of Base.

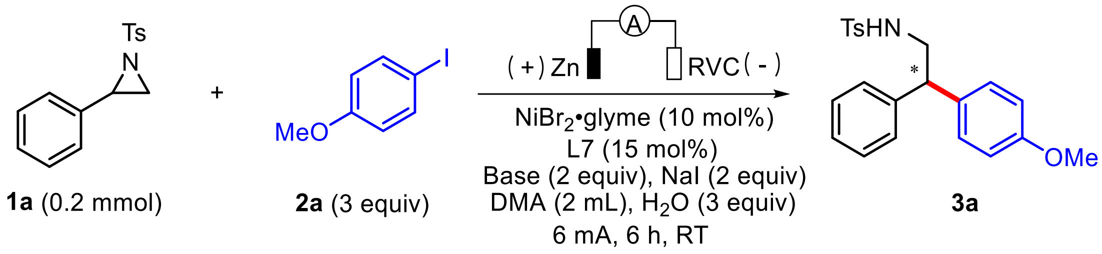

Method A

entry	variation from standard conditions	yields (%)	ee (%)
1	DIPEA	83	88
2	Lutidine	27	87
3	DBU	0	0
4	K_2CO_3	39	87
5	Cs_2CO_3	<5	87

Supplementary Table 15. Screening of Electrolytes.

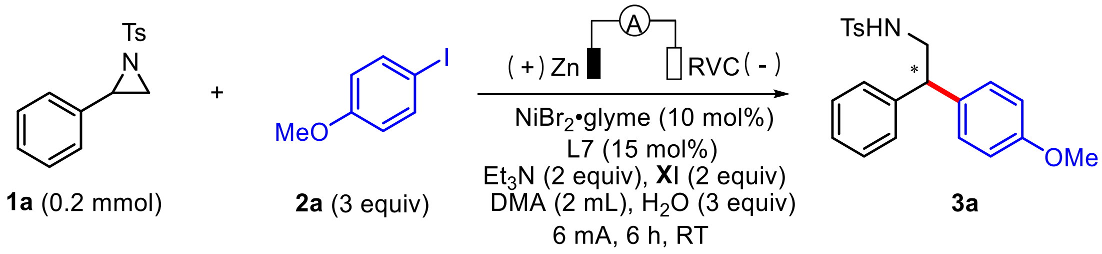

Method A

entry	variation from standard conditions	yield (%)	ee (%)
1	LiI	78	88
2	KI	42	86
3	$^{n}Bu_4NI$	59	88

2.6 Large-Scale Synthesis and Mechanistic Studies

(a) Large-scale synthesis of 3a

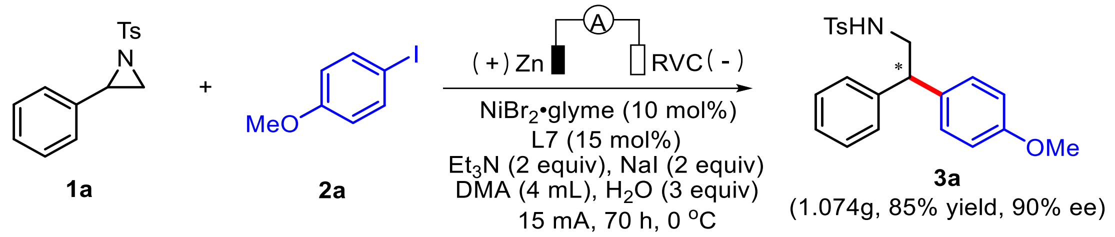

In Glovebox, an oven-dried electrochemical cell with a stir bar was charged with aziridine (3.4 mmol, 0.928 g, 1 equiv.) and aryl iodide (10.2 mmol, 2.387 g, 3 equiv.), $NiBr_2$·glyme (0.34 mmol, 105 mg, 10 mol%), ligand **L7** (0.51 mmol, 277 mg, 15 mol%), NaI (6.8 mmol, 1.02 g, 2 equiv.), triethylamine (6.8 mmol, 0.687 g, 2 equiv.), H_2O (10.2 mmol, 183.6mg, 3 equiv.), 34 mL of DMAc. The tube was installed an RVC as the cathode and zinc as the sacrificial anode. The mixture was stirred at 0 °C for 30 min. The reaction mixture was electrolyzed under a constant current of 15 mA at 0 °C until the complete consumption of the starting materials as monitored by TLC (about 70 hours). The resulting mixture was diluted with EtOAc, and quenched with sat. NH_4Cl solution, and the aqueous layer extracted with EtOAc (2 x 20 mL). The combined organic layers were dried over $MgSO_4$, the filtrate concentrated, and the crude product was purified by automated silica gel column chromatography (EtOAc/hexanes). Affording the desired product 3a in 85% (1.074 g) isolated yield (90% ee)

(b) Free radical capture experiments

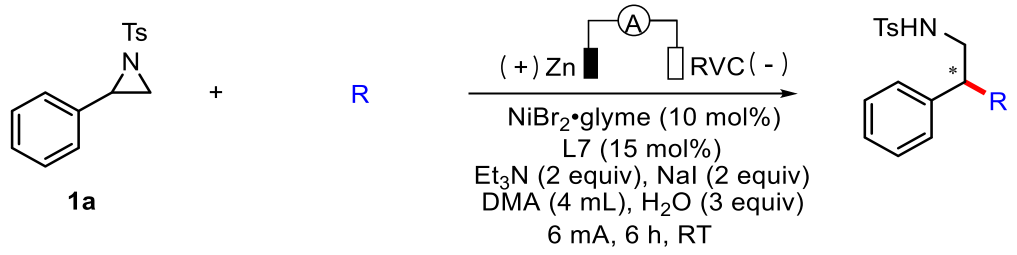

entry	R	conversion (%)	yield (%)
1	B_2pin_2	100	0
2	TEMPO	100	0
3	ABNO	100	0

(c) R/S aziridines

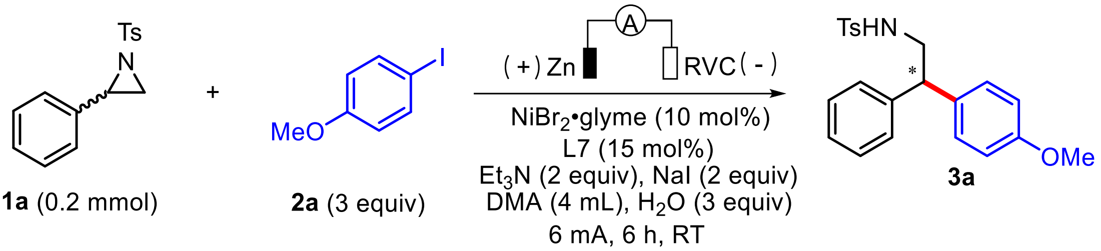

1a (0.2 mmol) **2a** (3 equiv) **3a**

entry	1a	Ligand	conversion (%)	yield (ee)
1	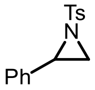	bpp<br>L7	100<br>100	55 (0)<br>85 (89)
2	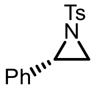 (R)	bpp<br>L7	100<br>100	48 (0)<br>78 (89)
3	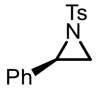 (S)	bpp<br>L7	100<br>100	50 (0)<br>74 (89)

The products have the same stereochemical structure.

(d) No [Ni] and Ligand

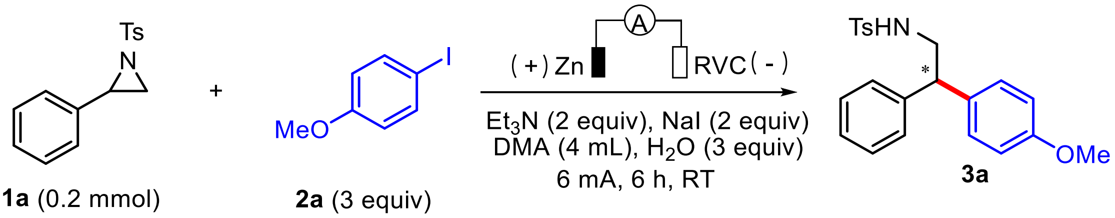

1a (0.2 mmol) **2a** (3 equiv) **3a**

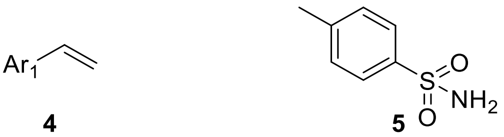

4 **5**

entry	Ar_1	3a (%)	4 (%)	5 (%)
1	Ph	0	/	61
2	Naphthyl	0	70	68

(e) Experiments with intermediate 4a

Standard conditions

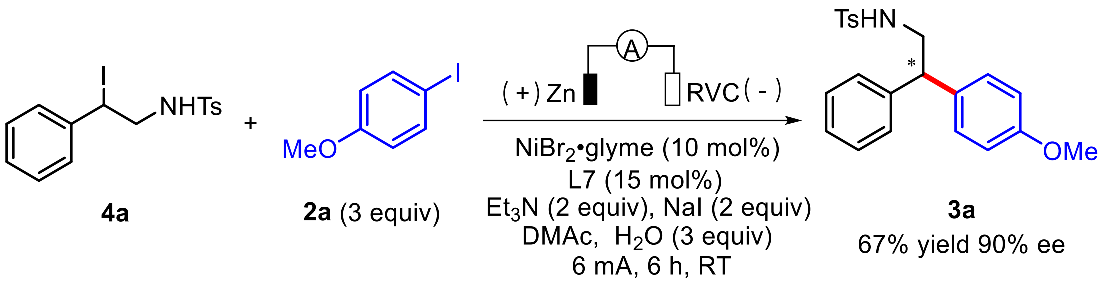

4a + **2a** (3 equiv) → **3a**

67% yield 90% ee

No [Ni] and Ligand

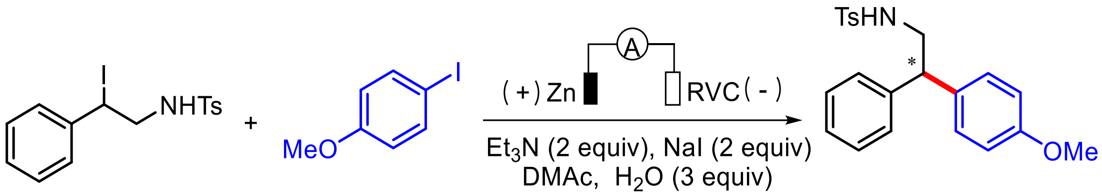

4a + **2a** (3 equiv) → **3a,** 0% yield

58% $TsNH_2$

Mixed substrate

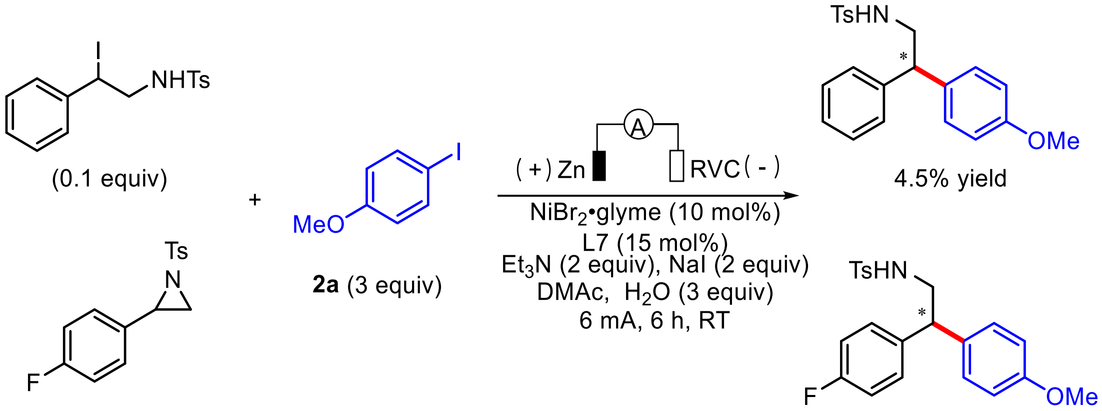

2.7 Synthetic Procedures and Characterization of Products

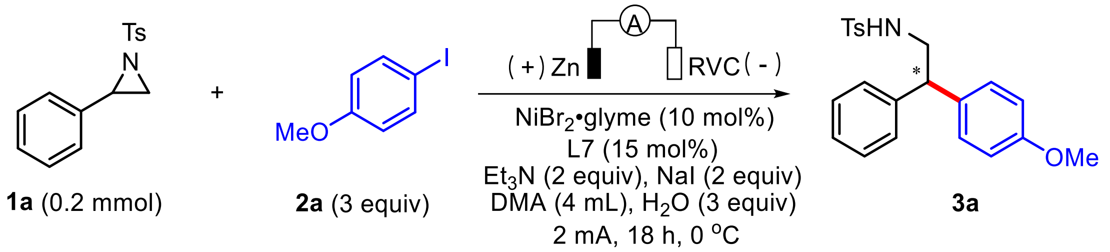

In glovebox, an oven-dried electrochemical cell with a stir bar was charged with aziridine (0.2 mmol, 55 mg, 1 equiv.) and aryl iodide (0.6 mmol, 141 mg, 3 equiv.), $NiBr_2$·glyme (0.02 mmol, 6.2 mg 10 mol%), ligand **L7** (0.03 mmol, 16.1 mg, 15 mol%), NaI (0.4 mmol, 60 mg, 2 equiv.), triethylamine (0.4 mmol, 2 equiv.), H_2O (0.6 mmol, 3 equiv.), 4 mL of DMAc. The tube was installed an RVC as the cathode and zinc as the sacrificial anode. The mixture was stirred at 0 °C for 30 min. The reaction mixture was electrolyzed under a constant current of 2 mA at 0 °C until the complete consumption of the starting materials as monitored by TLC (about 18 hours). The resulting mixture was diluted with EtOAc, and quenched with sat. NH_4Cl solution, and the aqueous layer extracted with EtOAc (2 x 20 mL). The combined organic layers were dried over $MgSO_4$, the filtrate concentrated, and the crude product was purified by automated silica gel column chromatography (EtOAc/hexanes).

Photographic Guide for Electrochemical coupling

Easily hand-made electrochemical cell

(1) Preparation of the electrode

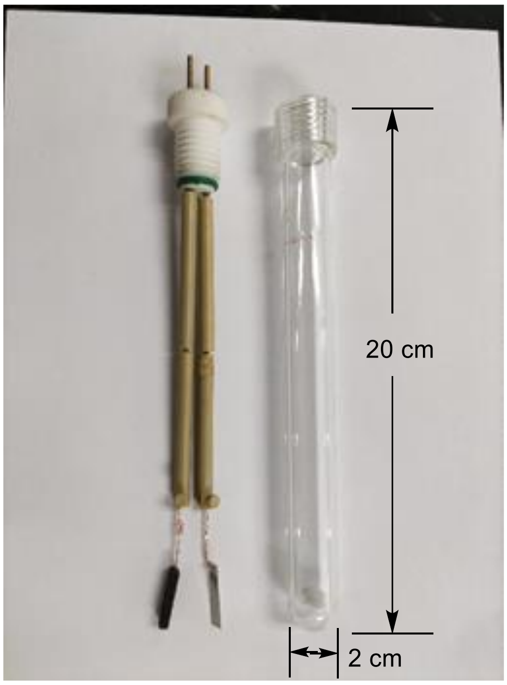

(0.2 mmol for the aziridines, 4.0 mL DMAc, 2.0 mA for 18h, 0 ℃, Zinc (0.8 x 1.5 cm^2) and RVC (0.8 x 1.5 x 0.3 cm^3) electrodes)

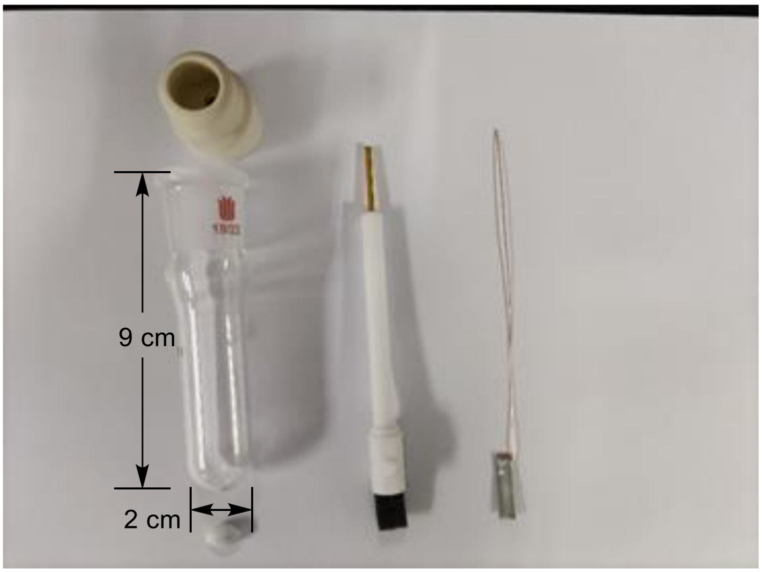

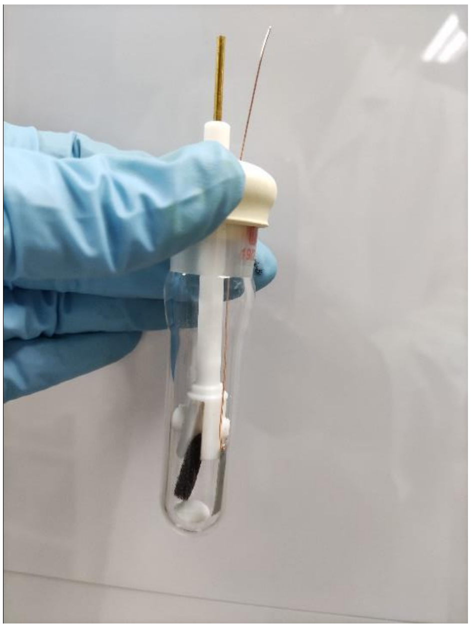

(0.2 mmol for the aziridines, 2.0 ml DMAc, 6.0 mA for 6 h, RT, Zinc (0.8 x 1.5 cm^2) and RVC (1.0 x 1.0 x 0.3 cm^3) electrodes)

(2) Electrolysis

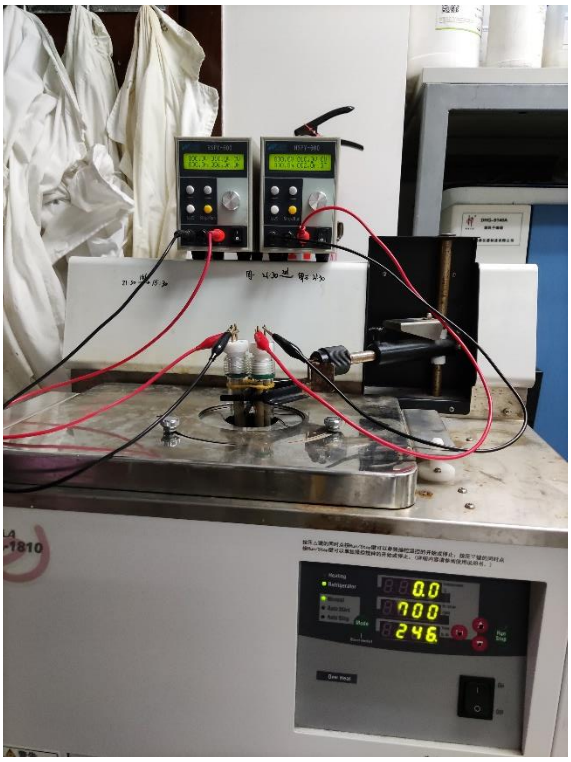

(3) Before and after electrolysis

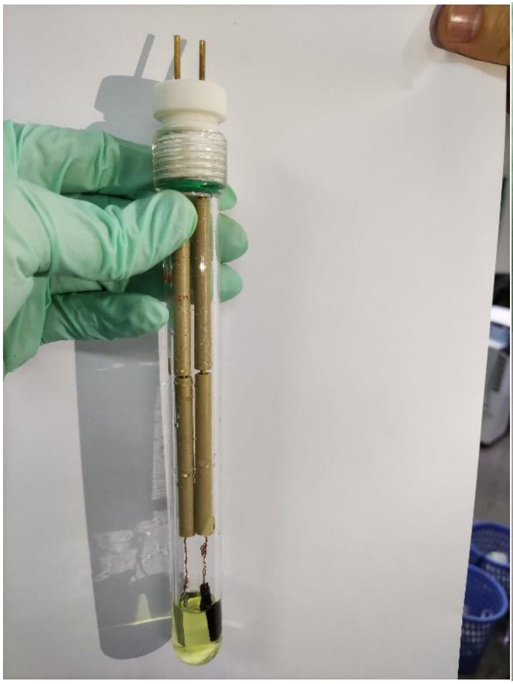

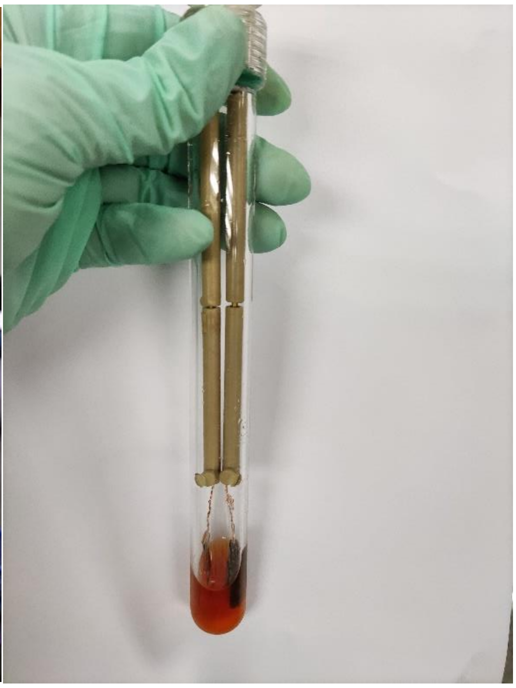

Photographic Guide for CV analysis

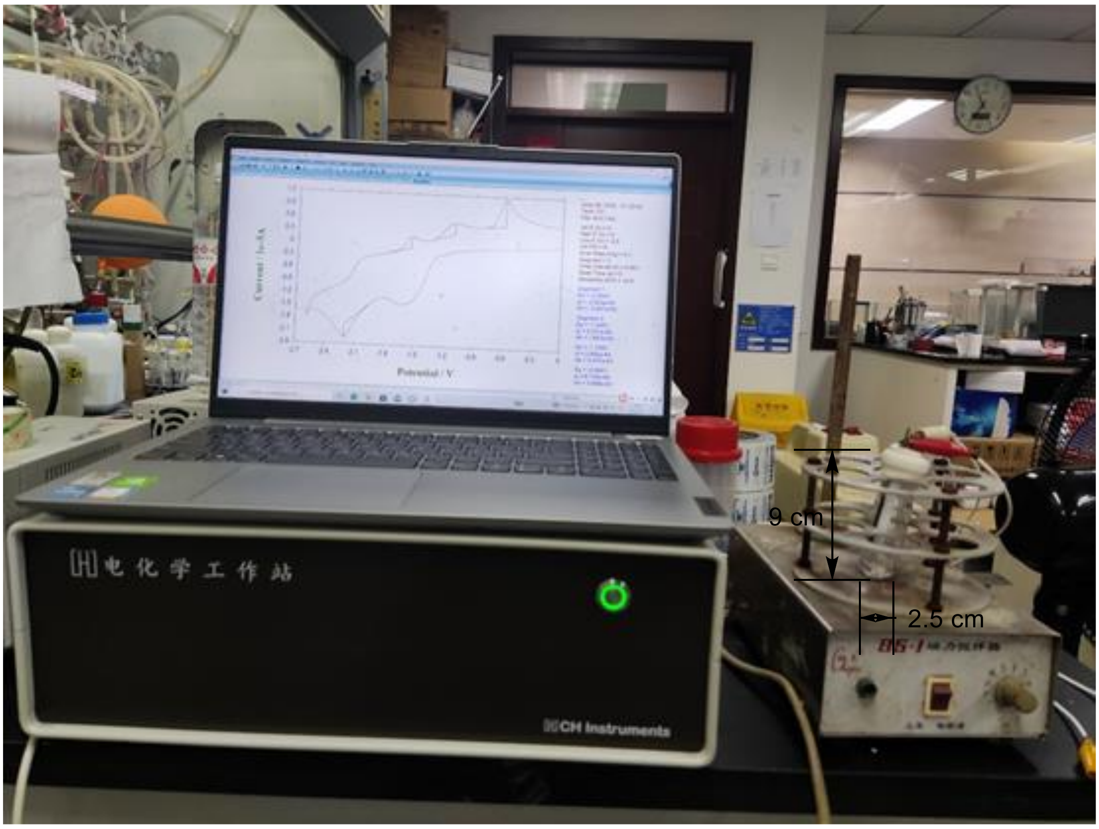

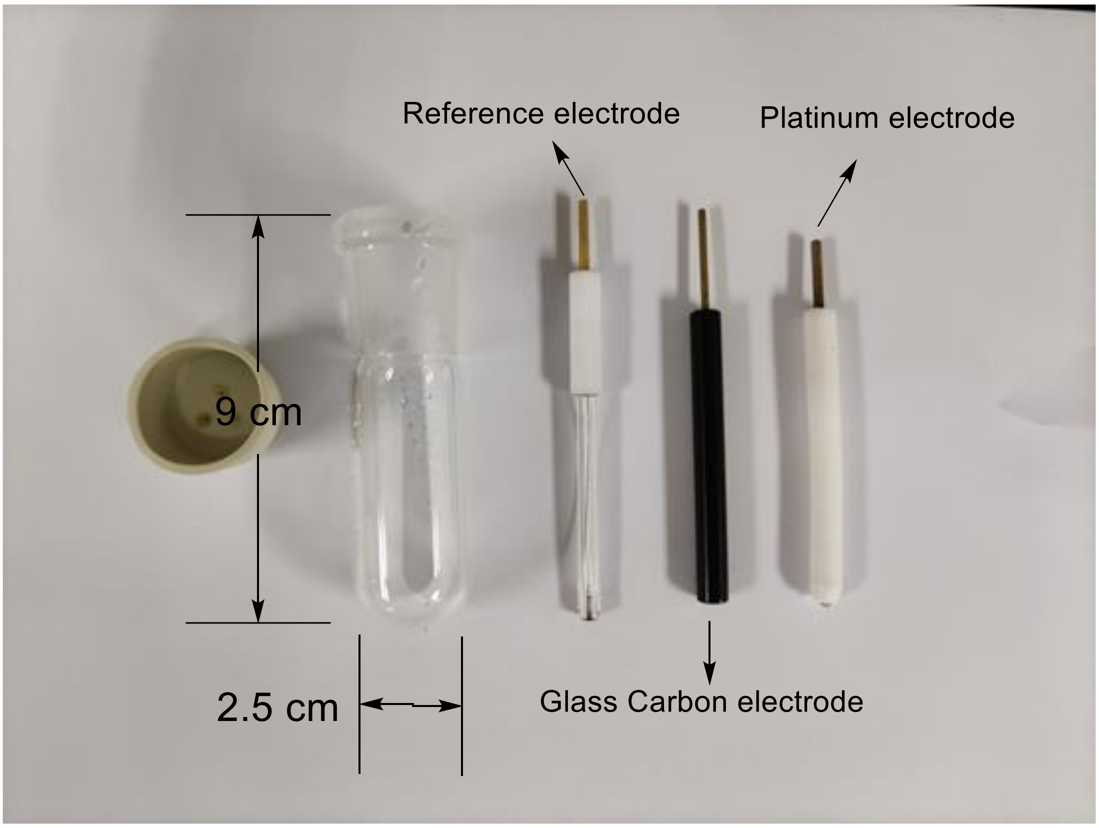

Isolated Yields and Characterization of Products

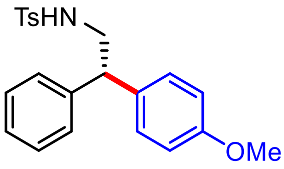

(S)-N-(2-(4-methoxyphenyl)-2-phenylethyl)-4-methylbenzenesulfonamide (3a)

Prepared according to the general procedure using **L7** from **1a** (0.2 mmol) and 1-iodo-4-methoxybenzene (0.6 mmol). The title compound was isolated (gradient 10–30% EtOAc/hexanes) as a white solid (72.5 mg, 95% yield, 90% ee).

^{1}H NMR (400 MHz, $CDCl_3$) δ 7.68 (d, *J* = 8 Hz, 2H), 7.27 (dd, *J* = 8.0, 7.6 Hz, 4H), 7.20 (t, *J* = 7.2 Hz, 1H), 7.09 (d, *J* = 7.2 Hz, 2H), 7.01 (d, *J* = 8.4 Hz, 2H), 6.80 (d, *J* = 8.8 Hz, 2H), 4.51 (s, 1H), 4.02 (t, *J* = 8.0 Hz, 1H), 3.76 (s, 3H), 3.58 – 3.44 (m, 2H), 2.44 (s, 3H).

^{13}C NMR (101 MHz, CDCl$_3$) δ 158.5, 143.4, 141.1, 136.7, 132.7, 129.7, 128.9, 128.8, 127.8, 127.0, 126.9, 114.2, 55.2, 49.7, 47.4, 21.5.

Optical Rotation: $[\alpha]_D^{26}$ 7.94 (c 1.0, CHCl$_3$)

HPLC: Chiralpak IC, 15% IPA in hexanes, 50 min run, 1 mL/min.

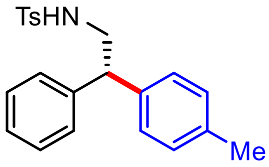

(S)-4-methyl-N-(2-phenyl-2-(p-tolyl)ethyl)benzenesulfonamide (3b)

Prepared according to the general procedure using **L7** from **1a** (0.2 mmol) and 1-iodo-4-methylbenzene (0.6 mmol). The title compound was isolated (gradient 10–30% EtOAc/hexanes) as a white solid (37 mg, 51% yield, 90% ee).

^{1}H NMR (400 MHz, CDCl$_3$) δ 7.69 (d, *J* = 8.0 Hz, 2H), 7.30 (d, *J* = 8.0 Hz, 2H), 7.26 (d, *J* = 8.0 Hz, 2H), 7.20 (t, *J* = 6.0 Hz, 1H), 7.12 – 7.06 (m, 4H), 6.99 (d, *J* = 8.0 Hz, 2H), 4.47 (t, *J* = 6.0 Hz, 1H), 4.03 (t, *J* = 8.0 Hz, 1H), 3.53 (m, 2H), 2.45 (s, 3H), 2.30 (s, 3H).

^{13}C NMR (101 MHz, CDCl$_3$) δ 143.4, 141.0, 137.7, 136.7, 136.7, 129.7, 129.5, 128.8, 127.8, 127.8, 127.1, 127.0, 50.1, 47.3, 21.5, 20.9.

Optical Rotation: $[\alpha]_D^{29}$ 5.22 (c 0.9, CHCl$_3$)

HPLC: Chiralpak IC, 15% IPA in hexanes, 35 min run, 1 mL/min.

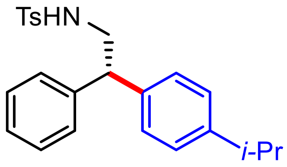

(S)-N-(2-(4-isopropylphenyl)-2-phenylethyl)-4-methylbenzenesulfonamide (3c)

Prepared according to the general procedure using **L7** from **1a** (0.2 mmol) and 1-iodo-4-isopropylbenzene. The title compound was isolated (gradient 10–30% EtOAc/hexanes) as a white solid (49 mg, 62% yield, 88% ee).

^{1}H NMR (400 MHz, CDCl$_3$) δ 7.69 (d, *J* = 8.4 Hz, 2H), 7.31 (d, *J* = 8.0 Hz, 2H), 7.26 (d, *J* = 7.6 Hz, 2H), 7.21 (t, *J* = 7.2 Hz, 1H), 7.12 (m, 4H), 7.02 (d, *J* = 8.0 Hz, 2H), 4.41 (t, *J* = 6.2 Hz, 1H), 4.03 (t, *J* = 8.0 Hz, 1H), 3.54 (dd, *J* = 8.0, 6.2 Hz, 2H), 2.86

(m, 1H), 2.45 (s, 3H), 1.22 (d, *J* = 6.8 Hz, 6H).

^{13}C NMR (101 MHz, CDCl$_3$) δ 147.6, 143.4, 140.1, 137.9, 136.7, 129.7, 128.8, 127.9, 127.7, 127.1, 127.0, 126.8, 50.1, 47.3, 33.6, 23.9, 21.5.

IR (neat): 3276, 3028, 1324, 1119, 868, 852, 694, 548, 484 cm^{-1}

HRMS (ESI) calcd for $C_{24}H_{27}N_1O_2NaS$ $[M+Na]^+$: 416.16465; found: 416.16547.

HPLC: Chiralpak OD-H, 10% IPA in hexanes, 20 min run, 1 mL/min.

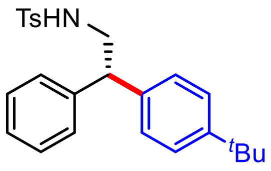

(S)-N-(2-(4-(tert-butyl)phenyl)-2-phenylethyl)-4-methylbenzenesulfonamide (3d)

Prepared according to the general procedure using **L7** from **1a** (0.2 mmol) and 1-(tert-butyl)-4-iodobenzene (0.6 mmol). The title compound was isolated (gradient 10–30% EtOAc/hexanes) as a white solid (53 mg, 65% yield, 81% ee).

^{1}H NMR (400 MHz, CDCl$_3$) δ 7.69 (d, *J* = 8.0 Hz, 2H), 7.29 (m, 6H), 7.21 (t, *J* = 8.0 Hz, 1H), 7.11 (d, *J* = 7.2 Hz, 2H), 7.04 (d, *J* = 8.0 Hz, 2H), 4.46 (d, *J* = 5.6 Hz, 1H), 4.04 (t, *J* = 8.0 Hz, 1H), 3.55 (dd, *J* = 8.0, 6.4 Hz, 2H), 2.45 (s, 3H), 1.29 (s, 9H).

^{13}C NMR (101 MHz, CDCl$_3$) δ 149.9, 143.4, 140.8, 137.5, 136.7, 129.7, 128.8, 127.9, 127.5, 127.1, 127.0, 125.7, 50.1, 47.3, 34.4, 31.2, 21.5.

Optical Rotation: $[\alpha]_D^{26}$ 4.50 (c 0.4, $CHCl_3$)

HPLC: Chiralpak OD-H, 10% IPA in hexanes, 25 min run, 1 mL/min.

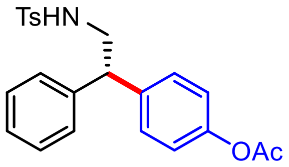

(S)-4-(2-((4-methylphenyl)sulfonamido)-1-phenylethyl)phenyl acetate (3e)

Prepared according to the general procedure using **L7** from **1a** (0.2 mmol) and 4-iodophenyl acetate (0.6 mmol). The title compound was isolated (gradient 10–30% EtOAc/hexanes) as a white solid (60.5 mg, 74% yield, 88% ee).

M. p. : 153.8 – 156.2 °C

^{1}**H NMR (400 MHz, CDCl$_3$)** δ 7.66 (d, *J* = 8.0 Hz, 2H), 7.31 – 7.23 (m, 4H), 7.20 (dd, *J* = 8.4, 6.1 Hz, 1H), 7.09 (dd, *J* = 7.6, 6.0 Hz, 4H), 6.97 (d, *J* = 8.8 Hz, 2H), 4.53 (t, *J* = 6.0 Hz, 1H), 4.07 (t, *J* = 8.0 Hz, 1H), 3.51 (dd, *J* = 8.0, 6.4 Hz, 2H), 2.43 (s, 3H), 2.26 (s, 3H).

^{13}**C NMR (101 MHz, CDCl$_3$)** δ 169.5, 149.6, 143.6, 140.4, 138.5, 136.8, 129.8, 128.94, 128.0, 127.2, 127.2, 121.9, 50.1, 47.3, 21.6, 21.1.

IR (neat): 3291, 1751, 1492, 1366, 1192, 1094, 1071, 855, 703, 560 cm^{-1}

HRMS (ESI) calcd for $C_{23}H_{23}N_1O_4NaS$ $[M+Na]^+$:432.12510; found: 432.12400.

Optical Rotation: $[\alpha]_D^{29}$ 4.20 (c 0.8, $CHCl_3$)

HPLC: Chiralpak OD-H, 15% IPA in hexanes, 35 min run, 1 mL/min.

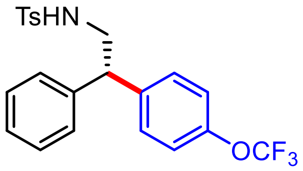

(S)-4-methyl-N-(2-phenyl-2-(4-(trifluoromethoxy)phenyl)ethyl)benzenesulfonamide (3f)

Prepared according to the general procedure using **L7** from **1a** (0.2 mmol) and 1-iodo-4-(trifluoromethoxy)benzene (0.6 mmol). The title compound was isolated (gradient 10–30% EtOAc/hexanes) as a yellow solid (57.5 mg, 66% yield, 84% ee).

M. p. : 119.8 – 121.2 °C

^{1}**H NMR (400 MHz, CDCl$_3$)** δ 7.65 (d, *J* = 8.0 Hz, 2H), 7.31 – 7.20 (m, 5H), 7.11 – 7.00 (m, 4H), 6.93 (t, *J* = 8.4 Hz, 2H), 4.39 (t, *J* = 5.6 Hz, 1H), 4.04 (t, *J* = 7.6Hz, 1H), 3.49 (t, *J* = 7.2 Hz, 2H), 2.43 (s, 3H).

^{13}**C NMR (101 MHz, CDCl$_3$)** δ 161.9, 159.5, 142.5, 139.5, 135.7, 135.5 (d, *J* = 3.3 Hz), 128.7, 128.42 (d, *J* = 8.0 Hz), 127.9, 126.8, 126.2, 126.1, 114.6 (d, *J* = 21.3 Hz), 48.8, 46.3, 20.5.

^{19}**F NMR (376 MHz, CDCl3)** δ -115.49.

IR (neat): 3279, 2924, 1599, 1324, 1264, 1155, 1091, 837, 661, 540 cm^{-1}

Optical Rotation: $[\alpha]_D^{29}$ 2.00 (c 0.5, $CHCl_3$)

HPLC: Chiralpak OJ-H, 15% IPA in hexanes, 45 min run, 0.7 mL/min.

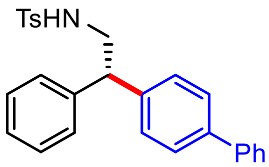

(S)-N-(2-([1,1'-biphenyl]-4-yl)-2-phenylethyl)-4-methylbenzenesulfonamide (3g) Prepared according to the general procedure using **L7** from **1a** (0.2 mmol) and 4-iodo-1,1'-biphenyl (0.6 mmol). The title compound was isolated (gradient 10–30% EtOAc/hexanes) as a yellow solid (42.8 mg, 50% yield, 90% ee).

M. p. : 153.6 – 156.7 °C

^{1}H NMR (400 MHz, $CDCl_3$) δ 7.68 (d, *J* = 8.0 Hz, 2H), 7.53 (d, *J* = 7.2 Hz, 2H), 7.48 (d, *J* = 8.4 Hz, 2H), 7.41 (t, *J* = 7.6 Hz, 2H), 7.33 (d, *J* = 7.2 Hz, 1H), 7.28 (dd, *J* = 7.6, 6.4 Hz, 4H), 7.22 (dd, *J* = 12.0, 4.8 Hz, 2H), 7.15 (dd, *J* = 13.8, 7.6 Hz, 4H), 4.47 (t, *J* = 6.0 Hz, 1H), 4.11 (t, *J* = 8.0 Hz, 1H), 3.64 – 3.51 (m, 2H), 2.42 (s, 3H).

^{13}C NMR (101 MHz, $CDCl_3$) δ 143.6, 140.7, 140.6, 140.1, 139.8, 136.8, 129.8, 129.0, 128.8, 128.4, 128.0, 127.6, 127.4, 127.2, 127.2, 127.0, 50.3, 47.3, 21.6.

IR (neat): 3274, 1597, 1322, 1071, 1005, 871, 780, 728, 661, 548 cm^{-1}

HRMS (ESI) calcd for $C_{27}H_{25}N_1O_2NaS$ $[M+Na]^+$: 450.15113; found: 450.14982.

Optical Rotation: $[\alpha]_D^{29}$ 19.90 (c 0.8, $CHCl_3$)

HPLC: Chiralpak OD-H, 20% IPA in hexanes, 35 min run, 1 mL/min.

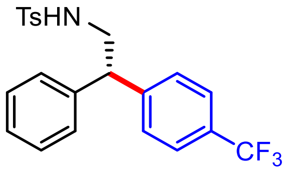

(S)-4-methyl-N-(2-phenyl-2-(4-(trifluoromethyl)phenyl)ethyl)benzenesulfonamide (3h) Prepared according to the general procedure using **L7** from **1a** (0.2 mmol) and 1-iodo-4-(trifluoromethyl)benzene (0.6 mmol). The title compound was isolated (gradient 10–30% EtOAc/hexanes) as a yellow solid (53.5 mg, 64% yield, 88.5% ee).

^{1}H NMR (400 MHz, CDCl3) δ 7.66 (d, *J* = 8.0 Hz, 2H), 7.50 (d, *J* = 8.0 Hz, 2H), 7.31 – 7.21 (m, 7H), 7.09 (d, *J* = 8.0 Hz, 2H), 4.71 (t, *J* = 6.0 Hz, 1H), 4.16 (t, *J* = 8.0 Hz, 1H), 3.56 (m, 2H), 2.44 (s, 3H).

^{13}C NMR (101 MHz, CDCl$_3$) δ 145.0, 143.7, 139.8, 136.7, 129.8, 129.3(q, J = 29 Hz), 129.1, 128.3, 127.9, 127.5, 125.7 (q, J = 4.0 Hz), 127.1, 124.0 (q, J = 270 Hz), 50.6, 47.0, 21.5.

^{19}F NMR (376 MHz, CDCl$_3$) δ -62.58.

Optical Rotation: $[\alpha]_D^{29}$ 3.00 (c 0.6, CHCl$_3$)

HPLC: Chiralpak OD-H, 10% IPA in hexanes, 30 min run, 1 mL/min.

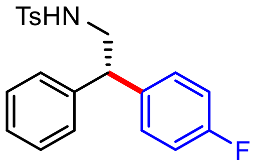

(S)-N-(2-(4-fluorophenyl)-2-phenylethyl)-4-methylbenzenesulfonamide (3i)

Prepared according to the general procedure using **L7** from **1a** (0.2 mmol) and 1-fluoro-4-iodobenzene (0.6 mmol). The title compound was isolated (gradient 10–30% EtOAc/hexanes) as a white solid (65.7 mg, 89% yield, 88% ee).

^{1}H NMR (400 MHz, CDCl$_3$) δ 7.67 (d, J = 8.0 Hz, 2H), 7.34 – 7.24 (m, 4H), 7.24 – 7.18 (m, 1H), 7.07 (t, J = 7.2 Hz, 4H), 6.93 (t, J = 8.4 Hz, 2H), 4.62 (t, J = 5.6 Hz, 1H), 4.07 (t, J = 7.6 Hz, 1H), 3.51 (t, J = 6.8 Hz, 2H), 2.44 (s, 3H).

^{13}C NMR (101 MHz, CDCl$_3$) δ 163.0, 160.6, 143.6, 140.7, 136.7, 136.7, 136.7, 129.8, 129.5, 129.5, 128.9, 127.9, 127.2, 127.1, 115.7, 115.5, 49.9, 47.4, 21.6.

^{19}F NMR (376 MHz, CDCl$_3$) δ -115.55.

IR (neat): 3300, 1598, 1496, 1318, 1150, 1122, 1094, 1074, 753, 537 cm^{-1}

Optical Rotation: $[\alpha]_D^{29}$ -2.19 (c 0.6, CHCl$_3$)

HPLC: Chiralpak OD-H, 10% IPA in hexanes, 30 min run, 1 mL/min.

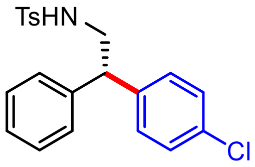

(S)-N-(2-(4-chlorophenyl)-2-phenylethyl)-4-methylbenzenesulfonamide (3j)

Prepared according to the general procedure using **L7** from **1a** (0.2 mmol) and 1-chloro-4-iodobenzene (0.6 mmol). The title compound was isolated (gradient 10–30% EtOAc/hexanes) as a white solid (68.2 mg, 85% yield, 88% ee).

^{1}H NMR (400 MHz, CDCl$_3$) δ 7.66 (d, *J* = 8.4 Hz, 2H), 7.27 (t, *J* = 8.0 Hz, 4H), 7.24 – 7.18 (m, 3H), 7.08 (d, *J* = 7.2 Hz, 2H), 7.04 (d, *J* = 8.4 Hz, 2H), 4.69 (t, *J* = 5.6 Hz, 1H), 4.06 (t, *J* = 8.0 Hz, 1H), 3.56 – 3.44 (m, 2H), 2.44 (s, 3H).

^{13}C NMR (101 MHz, CDCl$_3$) δ 143.5, 140.3, 139.4, 136.5, 132.7, 129.7, 129.3, 128.9, 128.8, 127.8, 127.2, 127.0, 50.0, 47.1, 21.5.

Optical Rotation: $[\alpha]_D^{28}$ 9.90 (c 0.6, CHCl$_3$)

HPLC: Chiralpak IC, 15% IPA in hexanes, 40 min run, 1 mL/min.

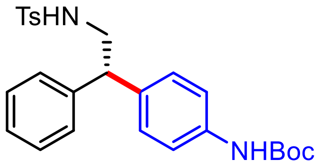

tert-butyl (S)-(4-(2-((4-methylphenyl)sulfonamido)-1-phenylethyl)phenyl)carbamate (3k)

Prepared according to the general procedure using **L7** from **1a** (0.2 mmol) and tert-butyl (4-iodophenyl)carbamate (0.6 mmol). The title compound was isolated (gradient 10–30% EtOAc/hexanes) as a white solid (87.6 mg, 94% yield, 89% ee).

M. p. : 146.7 – 148.0 °C

^{1}H NMR (400 MHz, CDCl$_3$) δ 7.68 (d, *J* = 8.4 Hz, 2H), 7.31 (d, *J* = 8.0 Hz, 2H), 7.26 (m, 5H), 7.07 (d, *J* = 7.2 Hz, 2H), 7.00 (d, *J* = 8.4 Hz, 2H), 6.48 (s, 1H), 4.32 (q, *J* = 6.0 Hz, 1H), 4.12 (q, *J* = 7.2 Hz, 1H), 4.01 (t, *J* = 7.6 Hz, 1H), 3.50 (dd, *J* = 14.0, 6.8 Hz, 2H), 2.45 (s, 3H), 1.50 (s, 9H).

^{13}C NMR (101 MHz, CDCl$_3$) δ 152.7, 143.5 , 140.8 , 137.3 , 136.7 , 135.1 , 129.7 , 128.8 , 128.5 , 127.8 , 127.1, 119.0 , 60.4 , 49.9 , 47.3 , 28.3 , 21.5 .

IR (neat): 3336, 2977, 1702, 1520, 1366. 1153, 1092, 734, 699, 543 cm^{-1}

HRMS (ESI) calcd for $C_{26}H_{30}N_2O_4NaS$ $[M+Na]^+$: 489.18099; found: 489.18185.

Optical Rotation: $[\alpha]_D^{27}$ 11.65 (c 1.5, CHCl$_3$)

HPLC: Chiralpak ID3, 70% IPA in hexanes, 30 min run, 0.7 mL/min.

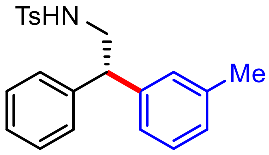

(S)-4-methyl-N-(2-phenyl-2-(m-tolyl)ethyl)benzenesulfonamide (3l)

Prepared according to the general procedure using **L7** from **1a** (0.2 mmol) and 1-iodo-3-methylbenzene (0.6 mmol). The title compound was isolated (gradient 10–30% EtOAc/hexanes) as a white solid (55 mg, 75% yield, 91% ee).

^{1}H NMR (400 MHz, CDCl$_3$) δ 7.67 (d, J = 8.4 Hz, 2H), 7.27 (m, 4H), 7.22 – 7.11 (m, 2H), 7.08 (d, J = 7.2 Hz, 2H), 7.01 (d, J = 7.6 Hz, 1H), 6.87 (s, 2H), 4.39 (s, 1H), 4.01 (t, J = 8.0 Hz, 1H), 3.52 (s, 2H), 2.43 (s, 3H), 2.27 (s, 3H).

^{13}C NMR (101 MHz, CDCl$_3$) δ 143.5, 140.8, 140.5, 138.5, 136.7, 129.7, 128.8, 128.7, 128.7, 127.9, 127.8, 127.1, 127.0, 124.8, 50.4, 47.2, 21.5, 21.4.

Optical Rotation: $[\alpha]_D^{28}$ 1.96 (c 0.5, CHCl$_3$)

HPLC: Chiralpak AD-H, 5% IPA in hexanes, 45 min run, 1 mL/min.

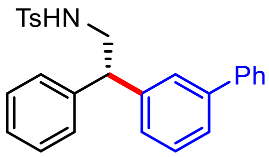

(S)-N-(2-([1,1'-biphenyl]-3-yl)-2-phenylethyl)-4-methylbenzenesulfonamide (3m)

Prepared according to the general procedure using **L7** from **1a** (0.2 mmol) and 3-iodo-1,1'-biphenyl (0.6 mmol). The title compound was isolated (gradient 10–30% EtOAc/hexanes) as a yellow oil (47 mg, 55% yield, 94% ee).

^{1}H NMR (400 MHz, CDCl$_3$) δ 7.66 (d, J = 8.0 Hz, 2H), 7.48 (d, J = 8.0 Hz, 2H), 7.45 – 7.37 (m, 3H), 7.33 (t, J = 7.6 Hz, 2H), 7.29 – 7.17 (m, 6H), 7.13 (d, J = 7.2 Hz, 2H), 7.08 (d, J = 7.6 Hz, 1H), 4.51 (t, J = 6.0 Hz, 1H), 4.13 (t, J = 8.0 Hz, 1H), 3.58 (t, J = 6.6 Hz, 2H), 2.39 (s, 3H).

^{13}C NMR (101 MHz, CDCl$_3$) δ 143.6, 141.9, 141.3, 140.9, 140.7, 136.8, 129.8, 129.3, 129.0, 128.8, 128.0, 127.5, 127.2, 127.2, 126.9, 126.1, 50.7, 47.4, 21.6.

IR (neat): 3275, 1597, 1323, 1153, 1073, 839, 728, 697, 549 cm^{-1}

HRMS (ESI) calcd for $C_{27}H_{25}N_1O_2NaS$ $[M+Na]^+$: 450.15074; found: 450.14982.

Optical Rotation: $[\alpha]_D^{28}$ 6.04 (c 0.5, CHCl$_3$)

HPLC: Chiralpak OD-H, 20% IPA in hexanes, 40 min run, 1 mL/min.

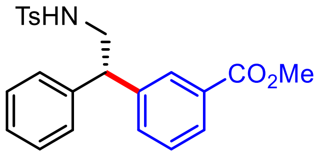

methyl (S)-3-(2-((4-methylphenyl)sulfonamido)-1-phenylethyl)benzoate (3n)

Prepared according to the general procedure using **L7** from **1a** (0.2 mmol) and methyl 3-iodobenzoate (0.6 mmol). The title compound was isolated (gradient 10–30% EtOAc/hexanes) as a white solid (38.5 mg, 47% yield, 88% ee).

M. p. : 138.3 – 139.1 °C

^{1}H NMR (400 MHz, $CDCl_3$) δ 7.89 (d, J = 7.2 Hz, 1H), 7.80 (s, 1H), 7.67 (d, J = 8.0 Hz, 2H), 7.32 (ddd, J = 10.8, 10.4, 4.9 Hz, 6H), 7.22 (t, J = 7.2 Hz, 1H), 7.10 (d, J = 6.8 Hz, 2H), 4.40 (s, 1H), 4.13 (t, J = 8.0 Hz, 1H), 3.89 (s, 3H), 3.65 – 3.50 (m, 2H), 2.44 (s, 3H).

^{13}C NMR (101 MHz, $CDCl_3$) δ 166.8, 143.6, 141.4, 140.2, 136.8, 132.6, 130.7, 129.8, 129.0, 129.0, 128.8, 128.4, 127.9, 127.4, 127.1, 52.2, 50.6, 47.1, 21.6.

IR (neat): 3263, 1713, 1152, 1009, 817, 621, 579, 556, 547, 497 cm^{-1}

HRMS (ESI) calcd for $C_{23}H_{23}N_1O_4NaS$ $[M+Na]^+$: 432.12497; found: 432.12400.

Optical Rotation: $[\alpha]_D^{27}$ 20.04 (c 0.1, $CHCl_3$)

HPLC: Chiralpak AD-H, 20% IPA in hexanes, 25 min run, 1 mL/min.

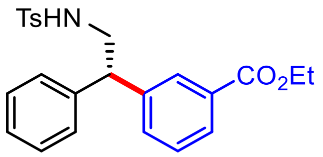

ethyl (S)-3-(2-((4-methylphenyl)sulfonamido)-1-phenylethyl)benzoate (3o)

Prepared according to the general procedure using **L7** from **1a** (0.2 mmol) and ethyl 3-iodobenzoate (0.6 mmol). The title compound was isolated (gradient 10–30% EtOAc/hexanes) as a white solid (43.1 mg, 51% yield, 90% ee).

^{1}H NMR (400 MHz, $CDCl_3$) δ 7.86 (d, J = 6.8 Hz, 1H), 7.79 (s, 1H), 7.65 (d, J = 8.0 Hz, 2H), 7.39 – 7.13 (m, 7H), 7.09 (d, J = 7.2 Hz, 2H), 4.57 (s, 1H), 4.33 (q, J = 6.8 Hz, 2H), 4.12 (t, J = 8.0 Hz, 1H), 3.55 (t, J = 6.4 Hz, 2H), 2.42 (s, 3H), 1.36 (t, J = 7.2 Hz, 3H).

^{13}C NMR (101 MHz, $CDCl_3$) δ 166.3, 143.5, 141.2, 140.2, 136.6, 132.4, 130.9, 129.7, 128.9, 128.8, 128.2, 127.8, 127.2, 127.0, 61.1, 50.5, 47.1, 21.5, 14.3.

Optical Rotation: $[\alpha]_D^{27}$ 12.52 (c 0.2, $CHCl_3$)

HPLC: Chiralpak AD-H, 15% IPA in hexanes, 30 min run, 1 mL/min.

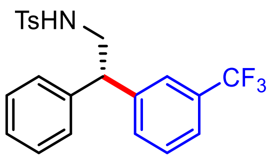

(S)-4-methyl-N-(2-phenyl-2-(3-(trifluoromethyl)phenyl)ethyl)benzenesulfonamide(3p)

Prepared according to the general procedure using **L7** from **1a** (0.2 mmol) and 1-iodo-3-(trifluoromethyl)benzene (0.6 mmol). The title compound was isolated (gradient 10–30% EtOAc/hexanes) as a white solid (59.5 mg, 71% yield, 90% ee).

^{1}H NMR (400 MHz, CDCl$_3$) δ 7.66 (d, *J* = 8.0 Hz, 2H), 7.46 (d, *J* = 7.6 Hz, 1H), 7.38 (t, *J* = 7.6 Hz, 1H), 7.35 – 7.20 (m, 7H), 7.08 (d, *J* = 7.2 Hz, 2H), 4.53 (s, 1H), 4.14 (t, *J* = 8.0 Hz, 1H), 3.61 – 3.49 (m, 2H), 2.44 (s, 3H).

^{13}C NMR (101 MHz, CDCl$_3$) δ 143.7, 142.0, 139.9, 136.7, 131.4, 131.1(q, *J* = 32 Hz), 129.9, 129.3, 129.1, 127.9, 127.5, 127.1, 124.6 (q, *J* = 3.7 Hz), 124.0 (q, *J* = 3.7 Hz), 124.0 (q, *J* = 270 Hz), 50.5, 47.1, 21.6.

^{19}F NMR (376 MHz, CDCl$_3$) δ -62.57.

Optical Rotation: $[\alpha]_D^{27}$ 6.21 (c 0.5, $CHCl_3$)

HPLC: Chiralpak AD-H, 10% IPA in hexanes, 20 min run, 1 mL/min.

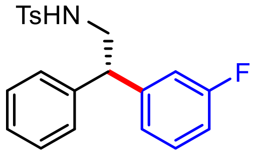

(S)-N-(2-(3-fluorophenyl)-2-phenylethyl)-4-methylbenzenesulfonamide (3q)

Prepared according to the general procedure using **L7** from **1a** (0.2 mmol) and 1-fluoro-3-iodobenzene (0.6 mmol). The title compound was isolated (gradient 10–30% EtOAc/hexanes) as a yellow solid (63.1 mg, 85.5% yield, 88% ee).

M. p. : 100.8 – 101.1 °C

^{1}H NMR (400 MHz, CDCl$_3$) δ 7.67 (d, *J* = 8.0 Hz, 2H), 7.36 – 7.19 (m, 6H), 7.09 (d, *J* = 7.2 Hz, 2H), 6.90 (m, 2H), 6.76 (d, *J* = 10.0 Hz, 1H), 4.57 (s, 1H), 4.08 (t, *J* = 7.6 Hz, 1H), 3.52 (t, *J* = 7.2 Hz, 2H), 2.44 (s, 3H).

^{13}C NMR (101 MHz, CDCl$_3$) δ 164.2, 161.8, 143.7, 143.5 (d, *J* = 6.9 Hz), 140.2, 136.7, 130.3 (d, *J* = 8.3 Hz), 129.8, 129.0, 127.9, 127.4, 127.1, 123.7, 123.6, 114.9 (d, *J* = 21.6 Hz), 114.0 (d, *J* = 20.9 Hz), 50.4, 50.4, 47.1, 21.6.

^{19}F NMR (376 MHz, $CDCl_3$) δ -112.25.

IR (neat): 3274, 2923, 1614, 1447, 1152, 1007, 908, 765, 689, 548 cm^{-1}

HRMS (ESI) calcd for $C_{21}H_{20}N_1O_2FNaS$ $[M+Na]^+$: 392.11056; found: 392.10910.

Optical Rotation: $[\alpha]_D{}^{28}$ 1.14 (c 0.9, $CHCl_3$)

HPLC: Chiralpak OD-H, 10% IPA in hexanes, 30 min run, 1 mL/min.

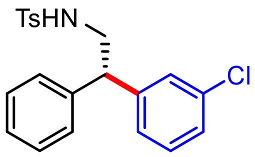

(S)-N-(2-(3-chlorophenyl)-2-phenylethyl)-4-methylbenzenesulfonamide(3r)

Prepared according to the general procedure using **L7** from **1a** (0.2 mmol) and 1-chloro-3-iodobenzene (0.6 mmol). The title compound was isolated (gradient 10–30% EtOAc/hexanes) as a white solid (53.3 mg, 69% yield, 88% ee).

^{1}H NMR (400 MHz, $CDCl_3$) δ 7.67 (d, J = 8.4 Hz, 2H), 7.34 – 7.27 (m, 3H), 7.26 (d, J = 3.2 Hz, 1H), 7.25 – 7.21 (m, 1H), 7.21 – 7.15 (m, 2H), 7.10 – 7.05 (m, 2H), 7.02 (dd, J = 7.2, 4.8 Hz, 2H), 4.46 (t, J = 6.0 Hz, 1H), 4.04 (t, J = 8.0 Hz, 1H), 3.52 (dd, J = 8.0, 6.4 Hz, 2H), 2.44 (s, 3H).

^{13}C NMR (101 MHz, $CDCl_3$) δ 143.7, 143.0, 140.0, 136.7, 134.7, 130.1, 129.9, 129.0, 128.1, 127.9, 127.4, 127.3, 127.1, 126.2, 50.4, 47.1, 21.6.

Optical Rotation: $[\alpha]_D{}^{28}$ 1.19 (c 0.9, $CHCl_3$)

HPLC: Chiralpak IC, 15% IPA in hexanes, 40 min run, 1 mL/min.

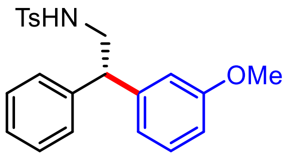

(S)-N-(2-(3-methoxyphenyl)-2-phenylethyl)-4-methylbenzenesulfonamide(3s)

Prepared according to the general procedure using **L7** from **1a** (0.2 mmol) and 1-iodo-3-methoxybenzene (0.6 mmol). The title compound was isolated (gradient 10–30% EtOAc/hexanes) as a white solid (62.5 mg, 82% yield, 91% ee).

^{1}H NMR (400 MHz, $CDCl_3$) δ 7.71 (d, J = 8.0 Hz, 2H), 7.36 – 7.26 (m, 4H), 7.26 – 7.19 (m, 2H), 7.13 (d, J = 7.2 Hz, 2H), 6.78 (dd, J = 8.0, 2.4 Hz, 1H), 6.73 (d, J = 7.6 Hz, 1H), 6.67 (s, 1H), 4.49 (t, J = 6.0 Hz, 1H), 4.07 (t, J = 8.0 Hz, 1H), 3.76 (s, 3H),

3.61 – 3.51 (m, 2H), 2.47 (s, 3H).

^{13}C NMR (101 MHz, $CDCl_3$) δ 159.9, 143.5, 142.3, 140.6, 136.7, 129.8, 129.7, 128.8, 127.9, 127.1, 120.2, 114.1, 112.0, 55.1, 50.6, 47.2, 21.6.

Optical Rotation: $[\alpha]_D^{26}$ 2.40 (c 0.4, $CHCl_3$)

HPLC: Chiralpak IC, 15% IPA in hexanes, 50 min run, 1 mL/min.

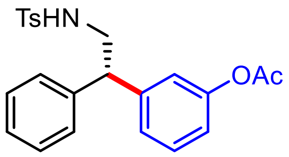

(S)-3-(2-((4-methylphenyl)sulfonamido)-1-phenylethyl)phenyl acetate(3t)

Prepared according to the general procedure using **L7** from **1a** (0.2 mmol) and 3-iodophenyl acetate (0.6 mmol). The title compound was isolated (gradient 10–30% EtOAc/hexanes) as a white solid (53.2 mg, 65% yield, 89% ee).

M. p. : 102.1 – 104.5 °C

^{1}H NMR (400 MHz, $CDCl_3$) δ 7.68 (d, *J* = 8.0 Hz, 2H), 7.34 – 7.25 (m, 5H), 7.21 (d, *J* = 7.2 Hz, 1H), 7.09 (d, *J* = 7.2 Hz, 2H), 6.97 (t, *J* = 9.2 Hz, 2H), 6.82 (s, 1H), 4.49 (s, 1H), 4.09 (t, *J* = 8.0 Hz, 1H), 3.53 (t, *J* = 7.2 Hz, 2H), 2.44 (s, 3H), 2.26 (s, 3H).

^{13}C NMR (101 MHz, $CDCl_3$) δ 169.3, 151.0, 143.6, 142.6, 140.2, 136.8, 129.8, 129.8, 129.0, 128.0, 127.3, 127.2, 125.4, 121.1, 120.4, 50.4, 47.2, 21.6, 21.2.

IR (neat): 3279, 1761, 1489, 1210, 1150, 1073, 1009, 796, 558, 487 cm^{-1}

HRMS (ESI) calcd for $C_{23}H_{23}N_1O_4NaS$ $[M+Na]^+$: 432.12501; found: 432.12400.

Optical Rotation: $[\alpha]_D^{28}$ 7.25 (c 0.2, $CHCl_3$)

HPLC: Chiralpak AD-H, 15% IPA in hexanes, 40 min run, 1 mL/min.

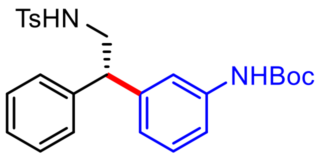

***Tert*-butyl (S)-(3-(2-((4-methylphenyl)sulfonamido)-1-phenylethyl)phenyl)carbamate(3u)** Prepared according to the general procedure using **L7** from **1a** (0.2 mmol) and tert-butyl (3-iodophenyl)carbamate (0.6 mmol). The title compound was isolated (gradient 10–30% EtOAc/hexanes) as a yellow oil (70 mg, 74% yield, 90% ee).

^{1}H NMR (400 MHz, CDCl$_3$) δ 7.70 (d, *J* = 8.0 Hz, 2H), 7.36 – 7.23 (m, 5H), 7.19 (m, 2H), 7.11 (d, *J* = 6.4 Hz, 3H), 6.78 (d, *J* = 7.6 Hz, 1H), 6.66 (s, 1H), 4.68 (t, *J* = 6.0 Hz, 1H), 4.04 (t, *J* = 8.0 Hz, 1H), 3.54 (t, *J* = 6.4 Hz, 2H), 2.45 (s, 3H), 1.52 (s, 9H).

^{13}C NMR (101 MHz, CDCl$_3$) δ 152.7, 143.5, 141.7, 140.8, 138.9, 136.8, 129.8, 129.5, 128.8, 127.9, 127.2, 127.1, 122.5, 118.1, 117.3, 50.6, 47.2, 28.4, 21.6.

IR (neat): 3336, 1704, 1596, 1492, 1152, 1092, 769, 729, 700, 549 cm^{-1}

HRMS (ESI) calcd for $C_{26}H_{30}N_2O_4NaS$ $[M+Na]^+$: 489.18147; found: 489.18185.

Optical Rotation: $[\alpha]_D^{27}$ 6.00 (c 0.4, CHCl$_3$)

HPLC: Chiralpak AD-H, 20% IPA in hexanes, 30 min run, 1 mL/min.

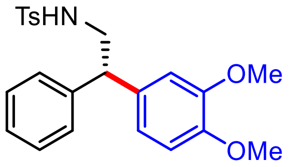

(S)-N-(2-(3,4-dimethoxyphenyl)-2-phenylethyl)-4-methylbenzenesulfonamide(3v)

Prepared according to the general procedure using **L7** from **1a** (0.2 mmol) and 4-iodo-1,2-dimethoxybenzene. The title compound was isolated (gradient 10–30% EtOAc/hexanes) as a white solid (74 mg, 90% yield, 92% ee).

M. p. : 137.3 – 138.2 °C

^{1}H NMR (400 MHz, CDCl$_3$) δ 7.68 (d, *J* = 8.0 Hz, 2H), 7.29 (m, 3H), 7.21 (t, *J* = 7.2 Hz, 1H), 7.11 (d, *J* = 7.2 Hz, 2H), 6.78 (d, *J* = 8.0 Hz, 1H), 6.67 (dd, *J* = 8.0, 1.6 Hz, 1H), 6.59 (d, *J* = 1.6 Hz, 1H), 4.50 (d, *J* = 60 Hz, 1H), 4.03 (t, *J* = 8.0 Hz, 1H), 3.84 (s, 3H), 3.77 (s, 3H), 3.59 – 3.45 (m, 2H), 2.45 (s, 3H).

^{13}C NMR (101 MHz, CDCl$_3$) δ 149.2, 148.1, 143.5, 141.1, 136.7, 133.1, 129.8, 128.9, 127.8, 127.2, 127.1, 119.8, 111.4, 111.3, 55.9, 55.8, 50.1, 47.4, 21.6.

IR (neat): 3281, 1590, 1493, 1292, 1230, 1093, 994, 864, 799, 599, 520 cm^{-1}

Optical Rotation: $[\alpha]_D^{27}$ 5.08 (c 0.6, CHCl$_3$)

HPLC: Chiralpak OD-H, 15% IPA in hexanes, 30 min run, 1 mL/min.

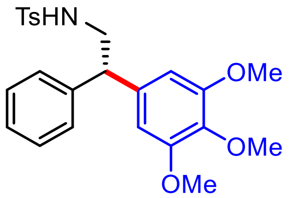

(S)-4-methyl-N-(2-phenyl-2-(3,4,5-trimethoxyphenyl)ethyl)benzenesulfonamide(3w) Prepared according to the general procedure using **L7** from **1a** (0.2 mmol) and 5-iodo-1,2,3-trimethoxybenzene (0.6 mmol). The title compound was isolated (gradient 10–30% EtOAc/hexanes) as a white solid (78.5 mg, 89% yield, 93% ee).

^{1}H NMR (400 MHz, CDCl$_3$) δ 7.65 (d, *J* = 8.0 Hz, 2H), 7.29 – 7.18 (m, 5H), 7.10 (d, *J* = 7.2 Hz, 2H), 6.30 (s, 2H), 4.64 (s, 1H), 4.00 (t, *J* = 7.6Hz, 1H), 3.77 (s, 3H), 3.73 (s, 6H), 3.56 – 3.41 (m, 2H), 2.40 (s, 3H).

^{13}C NMR (101 MHz, CDCl$_3$) δ 153.4, 143.5, 140.7, 136.7, 136.2, 129.7, 128.8, 127.7, 127.2, 127.1, 126.1, 105.1, 60.8, 56.1, 50.8, 47.3, 21.5.

HRMS (ESI) calcd for $C_{24}H_{27}N_1O_5NaS$ $[M+Na]^+$: 464.14979; found: 464.15021.

Optical Rotation: $[\alpha]_D^{26}$ 0.13 (c 0.3, CHCl$_3$)

HPLC: Chiralpak OD-H, 15% IPA in hexanes, 30 min run, 1 mL/min.

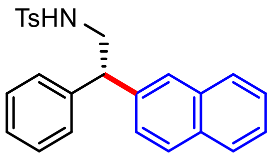

(S)-4-Methyl-N-(2-(naphthalen-2-yl)-2-phenylethyl)benzenesulfonamide(3x) Prepared according to the general procedure using **L7** from **1a** (0.2 mmol) and 2-iodonaphthalene (0.6 mmol). The title compound was isolated (gradient 10–30% EtOAc/hexanes) as a white solid (55 mg, 68% yield, 90% ee).

^{1}H NMR (400 MHz, CDCl$_3$) δ 7.77 (d, *J* = 7.6 Hz, 1H), 7.71 (d, *J* = 8.0 Hz, 2H), 7.65 (d, *J* = 8.0 Hz, 2H), 7.54 (s, 1H), 7.50 – 7.40 (m, 2H), 7.30 – 7.11 (m, 8H), 4.63 (d, *J* = 5.6 Hz, 1H), 4.23 (t, *J* = 7.6 Hz, 1H), 3.65 (t, *J* = 6.8 Hz, 2H), 2.42 (s, 3H).

^{13}C NMR (101 MHz, CDCl$_3$) δ 143.4, 140.7, 138.1, 136.6, 133.3, 132.4, 129.7, 128.8, 128.6, 128.0, 127.7, 127.5, 127.4, 127.1, 126.5, 126.3, 126.2, 125.9, 50.0, 47.1, 21.5.

Optical Rotation: $[\alpha]_D^{28}$ 20.33 (c 0.3, CHCl$_3$)

HPLC: Chiralpak IC, 15% IPA in hexanes, 50 min run, 1 mL/min.

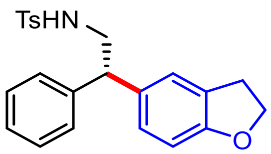

(S)-N-(2-(2,3-dihydrobenzofuran-5-yl)-2-phenylethyl)-4-methylbenzenesulfonamide(3y) Prepared according to the general procedure using **L7** from **1a** (0.2 mmol) and 5-iodo-2,3-dihydrobenzofuran (0.6 mmol). The title compound was isolated (gradient 10–30% EtOAc/hexanes) as a white solid (60 mg, 75% yield, 90% ee).

^{1}H NMR (400 MHz, $CDCl_3$) δ 7.66 (d, J = 8.0 Hz, 2H), 7.31 – 7.15 (m, 5H), 7.07 (d, J = 7.2 Hz, 2H), 6.89 (s, 1H), 6.79 (d, J = 8.0 Hz, 1H), 6.64 (d, J = 8.0 Hz, 1H), 4.51 (t, J = 8.6 Hz, 2H), 4.40 (t, J = 5.6 Hz, 1H), 3.97 (t, J = 7.8 Hz, 1H), 3.48 (dd, J = 14.6, 7.8 Hz, 2H), 3.10 (t, J = 8.4 Hz, 2H), 2.43 (s, 3H).

^{13}C NMR (101 MHz, $CDCl_3$) δ 159.1, 143.4, 141.2, 136.7, 132.5, 129.7, 128.7, 127.7, 127.7, 127.4, 127.1, 126.9, 124.5, 109.3, 71.2, 49.9, 47.4, 29.6, 21.5.

IR (neat): 3289, 1598, 1453, 1152, 1071, 980, 829, 697, 548 cm^{-1}

Optical Rotation: $[\alpha]_D^{25}$ 2.56 (c 0.5, $CHCl_3$)

HPLC: Chiralpak IC, 20% IPA in hexanes, 80 min run, 1 mL/min.

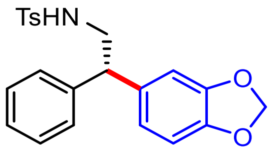

(S)-N-(2-(benzo[d][1,3]dioxol-5-yl)-2-phenylethyl)-4-methylbenzenesulfonamide(3z) Prepared according to the general procedure using **L7** from **1a** (0.2 mmol) and 5-iodobenzo[d][1,3]dioxole (0.6 mmol). The title compound was isolated (gradient 10–30% EtOAc/hexanes) as a yellow solid (55.3 mg, 70% yield, 89% ee).

^{1}H NMR (400 MHz, $CDCl_3$) δ 7.68 (d, J = 8.4 Hz, 2H), 7.28 (m, 4H), 7.20 (t, J = 7.2 Hz, 1H), 7.08 (d, J = 7.2 Hz, 2H), 6.70 (d, J = 8.0 Hz, 1H), 6.57 (dd, J = 8.0, 1.6 Hz, 1H), 6.53 (d, J = 1.6 Hz, 1H), 5.90 (s, 2H), 4.47 (t, J = 6.0 Hz, 1H), 3.97 (t, J = 8.0 Hz, 1H), 3.55 – 3.42 (m, 2H), 2.44 (s, 3H).

^{13}C NMR (101 MHz, $CDCl_3$) δ 148.0, 146.5, 143.5, 140.8, 136.7, 134.5, 129.7, 128.8, 127.7, 127.1, 121.0, 109.9, 108.4, 108.2, 101.7, 50.2, 47.2, 21.5.

Optical Rotation: $[\alpha]_D^{27}$ 3.85 (c 0.3, $CHCl_3$)

HPLC: Chiralpak IC, 15% IPA in hexanes, 60 min run, 1 mL/min.

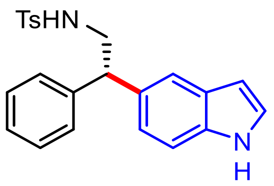

(S)-N-(2-(1H-indol-5-yl)-2-phenylethyl)-4-methylbenzenesulfonamide(3aa)

Prepared according to the general procedure using **L7** from **1a** (0.2 mmol) and 5-iodo-1*H*-indole (0.6 mmol). The title compound was isolated (gradient 10–30% EtOAc/hexanes) as a white solid (61.6 mg, 79% yield, 90% ee).

M. p. : 147.9 – 150.0 °C

^{1}H NMR (400 MHz, CDCl$_3$) δ 7.79 (d, *J* = 8.0 Hz, 1H), 7.67 (d, *J* = 8.4 Hz, 2H), 7.32 – 7.25 (m, 7H), 7.13 (d, *J* = 7.2 Hz, 2H), 6.89 (d, *J* = 7.2 Hz, 1H), 6.45 (s, 1H), 4.97 (s, 1H), 4.47 (t, *J* = 6.0 Hz, 1H), 4.15 (s, 1H), 3.66 – 3.53 (m, 2H), 2.45 (s, 3H).

^{13}C NMR (101 MHz, CDCl$_3$) δ 143.5, 141.8, 136.7, 134.9, 131.8, 129.7, 128.7, 127.9, 127.1, 126.8, 126.4, 124.9, 122.3, 119.6, 111.6, 102.4, 50.4, 47.6, 21.5.

IR (neat): 3267, 3028, 1597, 1321, 1154, 1092, 894, 812, 730, 551 cm^{-1}

HRMS (ESI) calcd for $C_{23}H_{22}N_2O_2NaS$ [M+Na]$^+$: 413.13038; found: 413.12942.

Optical Rotation: $[\alpha]_D{}^{25}$ 5.14 (c 0.3, CHCl$_3$)

HPLC: Chiralpak AD-H, 15% IPA in hexanes, 65 min run, 1 mL/min.

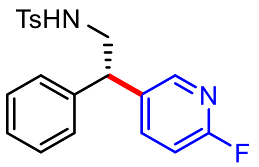

(S)-N-(2-(6-fluoropyridin-3-yl)-2-phenylethyl)-4-methylbenzenesulfonamide(3ab)

Prepared according to the general procedure using **L7** from **1a** (0.2 mmol) and 2-fluoro-5-iodopyridine (0.6 mmol). The title compound was isolated (gradient 10–30% EtOAc/hexanes) as a white solid (30 mg, 40% yield, 70.5% ee).

^{1}H NMR (400 MHz, CDCl$_3$) δ 8.00 (s, 1H), 7.69 (d, *J* = 8.0 Hz, 2H), 7.57 (td, *J* = 8.0, 2.4 Hz, 1H), 7.36 – 7.24 (m, 6H), 7.11 (d, *J* = 7.2 Hz, 2H), 6.84 (dd, *J* = 8.4, 2.8 Hz, 1H), 4.77 (t, *J* = 6.0 Hz, 1H), 4.15 (t, *J* = 8.0 Hz, 1H), 3.61 – 3.49 (m, 2H), 2.46 (s, 3H).

^{13}C NMR (101 MHz, cdcl$_3$) δ 147.0, 146.8, 143.8, 140.7, 140.6, 139.4, 136.5, 129.8, 129.2, 127.8, 127.6, 127.0, 109.6 (d, *J* = 37.0 Hz), 47.6, 47.0, 21.5.

^{19}F NMR (377 MHz, CDCl$_3$) δ -70.23.

IR (neat): 3283, 2922, 1597, 1425, 1251, 1129, 1038, 745, 582, 504 cm^{-1}

Optical Rotation: $[\alpha]_D^{24}$ 0.03 (c 0.5, CHCl$_3$)

HPLC: Chiralpak AD-H, 10% IPA in hexanes, 50 min run, 1 mL/min.

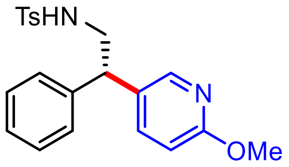

(S)-N-(2-(6-methoxypyridin-3-yl)-2-phenylethyl)-4-methylbenzenesulfonamide (3ac) Prepared according to the general procedure using **L7** from **1a** (0.2 mmol) and 5-iodo-2-methoxypyridine (0.6 mmol). The title compound was isolated (gradient 10–30% EtOAc/hexanes) as a white solid (42 mg, 55% yield, 80% ee). **M. p. :** 113.0 – 115.5 °C

^{1}H NMR (400 MHz, CDCl$_3$) δ 7.94 (d, J = 2.4 Hz, 1H), 7.69 (d, J = 8.0 Hz, 2H), 7.34 – 7.27 (m, 5H), 7.27 – 7.21 (m, 1H), 7.10 (d, J = 7.2 Hz, 2H), 6.65 (d, J = 8.4 Hz, 1H), 4.61 (t, J = 6.2 Hz, 1H), 4.03 (t, J = 8.0 Hz, 1H), 3.90 (s, 3H), 3.53 (dd, J = 7.6, 6.4 Hz, 2H), 2.46 (s, 3H).

^{13}C NMR (101 MHz, cdcl$_3$) δ 163.2, 145.9, 143.6, 140.1, 138.1, 136.6, 129.8, 129.2, 129.0, 127.8, 127.3, 127.1, 111.1, 53.4, 47.5, 47.1, 21.5.

IR (neat): 3276, 1604, 1491, 1318, 1287, 1152, 1074, 1024, 776, 698, 546 cm^{-1}

HRMS (ESI) calcd for $C_{21}H_{23}N_2O_3S$ $[M+H]^+$: 383.14167; found: 383.14239.

Optical Rotation: $[\alpha]_D^{29}$ -0.65 (c 0.3, CHCl$_3$)

HPLC: Chiralpak AD-H, 20% IPA in hexanes, 30 min run, 1 mL/min.

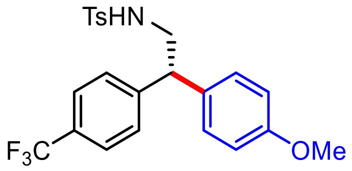

(S)-N-(2-(4-methoxyphenyl)-2-(4-(trifluoromethyl)phenyl)ethyl)-4-methylbenzenesulfonamide (3ad) Prepared according to the general procedure using **L7** from 1-tosyl-2-(4-(trifluoromethyl)phenyl)aziridine (0.2 mmol) and **2a** (0.6 mmol). The title compound was isolated (gradient 10–30% EtOAc/hexanes) as a white solid (80.8 mg, 90% yield, 77% ee).

M. p. : 128.1 – 129.7 °C

^{1}H NMR (400 MHz, $CDCl_3$) δ 7.66 (d, J = 8.0 Hz, 2H), 7.50 (d, J = 8.0 Hz, 2H), 7.29 (d, J = 8.0 Hz, 2H), 7.21 (d, J = 8.0 Hz, 2H), 7.00 (d, J = 8.8 Hz, 2H), 6.82 (d, J = 8.8 Hz, 2H), 4.52 (t, J = 6.0 Hz, 1H), 4.10 (t, J = 8.0 Hz, 1H), 3.77 (s, 3H), 3.59 – 3.44 (m, 2H), 2.44 (s, 3H).

^{13}C NMR (101 MHz, $CDCl_3$) δ 158.8, 145.4, 143.7, 136.7, 131.8, 129.8,129.2 (q, J = 32.4 Hz), 129.0, 128.2, 127.1,125.7(q, J = 3.7 Hz), 122.7, 114.5, 55.3, 49.7, 47.2, 21.5.

IR (neat): 3282, 1617, 1246, 1185, 1082, 853, 836, 665, 552, 535, 496 cm^{-1}

HRMS (ESI) calcd for $C_{23}H_{22}N_1O_3F_3NaS$ $[M+Na]^+$: 472.11730; found: 472.11647.

Optical Rotation: $[\alpha]_D^{28}$ -4.73 (c 0.2, $CHCl_3$)

HPLC: Chiralpak IC, 15% IPA in hexanes, 35 min run, 1 mL/min.

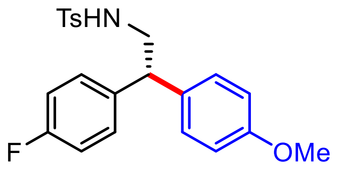

(R)-N-(2-(4-fluorophenyl)-2-(4-methoxyphenyl)ethyl)-4-methylbenzenesulfonamide(3ae) Prepared according to the general procedure using **L7** from 2-(4-fluorophenyl)-1-tosylaziridine (0.2 mmol) and **2a** (0.6 mmol). The title compound was isolated (gradient 10–30% EtOAc/hexanes) as a white solid (71.8 mg, 90% yield, 88% ee).

M. p. : 124.3 – 126.5 °C

^{1}H NMR (400 MHz, $CDCl_3$) δ 7.66 (d, J = 8.4 Hz, 2H), 7.29 (d, J = 8.0 Hz, 2H), 7.08 – 7.02 (m, 2H), 6.98 (t, J = 5.6 Hz, 2H), 6.93 (t, J = 8.4 Hz, 2H), 6.83 – 6.78 (m, 2H), 4.55 (d, J = 5.6 Hz, 1H), 4.01 (t, J = 8.0 Hz, 1H), 3.76 (s, 3H), 3.47 (m, 2H), 2.44 (s, 3H).

^{13}C NMR (101 MHz, $CDCl_3$) δ 162.9, 160.5, 158.7, 143.6, 136.8, 132.6, 129.8, 129.4(d, J = 8.0 Hz), 128.9, 127.1, 115.6 (d, J = 22.0 Hz), 114.3, 55.3, 49.1, 47.5, 21.6.

IR (neat): 3282, 1601, 1427, 1222, 1095, 1013, 875, 726, 708, 583 cm^{-1}

HRMS (ESI) calcd for $C_{22}H_{22}N_1O_3FNaS$ $[M+Na]^+$: 422.12040; found: 422.11966.

Optical Rotation: $[\alpha]_D^{29}$ 20.47 (c 0.3, $CHCl_3$)

HPLC: Chiralpak AD-H, 15% IPA in hexanes, 50 min run, 1 mL/min.

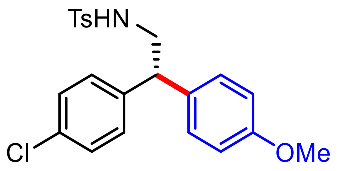

(R)-N-(2-(4-chlorophenyl)-2-(4-methoxyphenyl)ethyl)-4-methylbenzenesulfonamide(3af) Prepared according to the general procedure using **L7** from 2-(4-chlorophenyl)-1-tosylaziridine (0.2 mmol) and **2a** (0.6 mmol). The title compound was isolated (gradient 10–30% EtOAc/hexanes) as a white solid (72.5 mg, 92% yield, 83% ee).

M. p. : 151.1 – 154.2 °C

^{1}H NMR (400 MHz, CDCl$_3$) δ 7.65 (d, *J* = 8.4 Hz, 2H), 7.28 (d, *J* = 8.0 Hz, 2H), 7.20 (d, *J* = 8.4 Hz, 2H), 7.00 (dd, *J* = 14.0, 8.4 Hz, 4H), 6.80 (d, *J* = 8.8 Hz, 2H), 4.66 (d, *J* = 4.8 Hz, 1H), 4.01 (t, *J* = 8.0 Hz, 1H), 3.76 (s, 3H), 3.53 – 3.40 (m, 2H), 2.44 (s, 3H).

^{13}C NMR (101 MHz, CDCl$_3$) δ 158.7, 143.6, 139.9, 136.7, 132.7, 132.4, 129.8, 129.2, 128.9, 128.9, 127.1, 114.3, 55.3, 49.2, 47.3, 21.6.

IR (neat): 3283, 1609, 1320, 1247, 1152, 1083, 817, 666, 552 cm^{-1}

HRMS (ESI) calcd for $C_{22}H_{22}N_1O_3NaSCl$ $[M+Na]^+$: 438.08952; found: 438.09011.

Optical Rotation: $[\alpha]_D{}^{29}$ -4.77 (c 0.4, CHCl$_3$)

HPLC: Chiralpak IC, 15% IPA in hexanes, 50 min run, 1 mL/min.

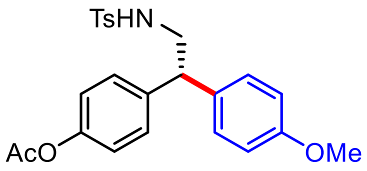

(R)-4-(1-(4-methoxyphenyl)-2-((4-methylphenyl)sulfonamido)ethyl)phenyl acetate(3ag) Prepared according to the general procedure using **L7** from 4-(1-tosylaziridin-2-yl)phenyl acetate (0.2 mmol) and **2a** (0.6 mmol). The title compound was isolated (gradient 10–30% EtOAc/hexanes) as a white solid (50.4 mg, 58% yield, 85.5% ee).

M. p. : 158.1 – 162.6 °C

^{1}H NMR (400 MHz, CDCl$_3$) δ 7.68 (d, *J* = 8.4 Hz, 2H), 7.30 (d, *J* = 8.0 Hz, 2H), 7.08

(d, J = 8.4 Hz, 2H), 6.99 (t, J = 8.0 Hz, 4H), 6.81 (d, J = 8.8 Hz, 2H), 4.39 (t, J = 6.0 Hz, 1H), 4.02 (t, J = 8.0 Hz, 1H), 3.77 (s, 3H), 3.56 – 3.39 (m, 2H), 2.44 (s, 3H), 2.27 (s, 3H).

^{13}C NMR (101 MHz, $CDCl_3$) δ 169.5, 158.7, 149.5, 143.6, 138.8, 136.8, 132.3, 129.8, 129.0, 128.8, 127.2, 121.8, 114.4, 55.3, 49.2, 47.5, 21.6, 21.1.

IR (neat): 3276, 1752, 1502, 1247, 1154, 1094, 1012, 873, 813, 706, 633 cm^{-1}

HRMS (ESI) calcd for $C_{24}H_{25}N_1O_5NaS$ $[M+Na]^+$: 462.13519; found: 462.13456.

Optical Rotation: $[\alpha]_D^{29}$ 0.66 (c 0.3, $CHCl_3$)

HPLC: Chiralpak AD-H, 15% IPA in hexanes, 40 min run, 1 mL/min.

3. Supplementary Figures

3.1 Mathematical modeling

Geometry optimizations and frequency calculations were carried out using Gaussian 16 software.[1] The functional used for DFT of the substrates' calculation is M06-2X, which was previously benchmarked for thermodynamic and kinetic accuracy of main group elements, and for non-covalent interactions.[2,3] The basis-set used was def2-SVP. All regression models were calculated using R (V 4.1.3)[4] and R Studio[5] and the packages[6–22] used in code are caret, car, ggrepel, tidyr, reshape2, scales, tibble, caret, plyr, dplyr, data.table, nnet, ggplot2, knitr, stringr, pracma, and matlib. Depictions and graphic representations were made using Chem draw (v.20.0), Adobe Illustrator[23], Origin lab[24] and CYLview[25].

Parameters

Sterimol parameters were extracted using an in-house program (SteRimol, available at https://github.com/Milo-group/SteRimol), based on Verloop's original definitions.[26,27] Natural Population Analysis (NPA) was used as an indication of the partial charge and values were extracted using Gaussian's NBO 3.1.
Supplementary Fig. 12 is a representation of the intermediate used for the substrate in this paper and for the paper from the Doyle group[28].

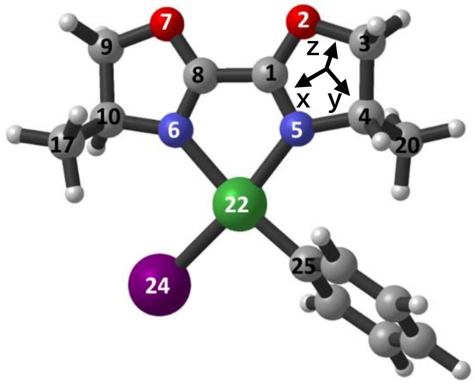

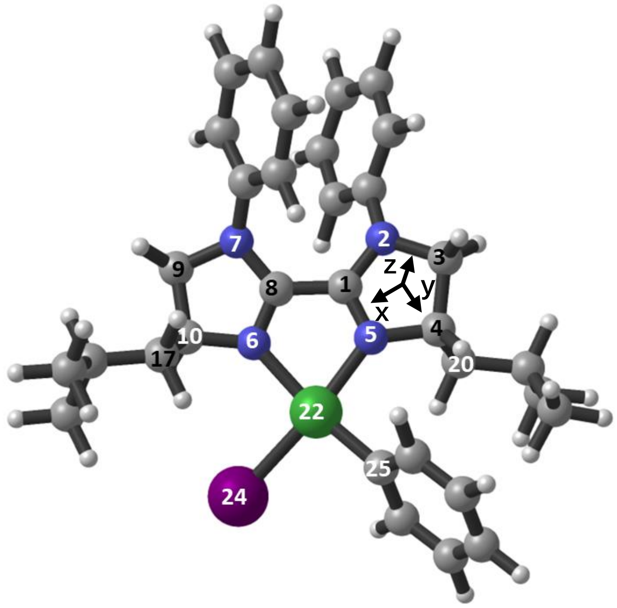

Supplementary Fig. 13 *Representation of the numbering on the Intermediates for BiIm and BisOx*

Distances (d) between the atom pairs: C1−N2, C1−N5, C1−C8, C10−C17, C4−C20, N6−C8, N6−Ni22, N5−Ni22, Ni22−Ni24.
Vibration (ν) was measured on atoms C1−N5, C8−N6.
Dipole was taken both as a general dipole (μ_{tot}) and divided according to xyz coordinate system [28]. On the Y axis (μ_y) is from atom 2 to 4 (towards the substituent). The XY plane is on the surface of the 5-membered ring, and the dipole on the Z axis (μ_z) is perpendicular to the 5-membered ring surface.
NPA charges were extracted for all the atoms numbered on Supplementary Fig. 13.
Differences between the NPA charges (ΔNPA) were calculated for the following pairs: C1−N5, N6−C8, Ni22−I24, Ni22−C25.
The following dihedral angels ($<_{tor}$) were extracted: N5−C1−C8−N6, O2−C1−C8−N6, C1−N5−Ni22−C25 and C8−N6−Ni22−I24.
The following angles (<) were extracted: N5−Ni22−C25, C25−Ni22−I24, I24−Ni22−N6 and N5−Ni22−N6.
Anisotropic polarizability and isotropic polarizability were extracted.

Possible confirmations

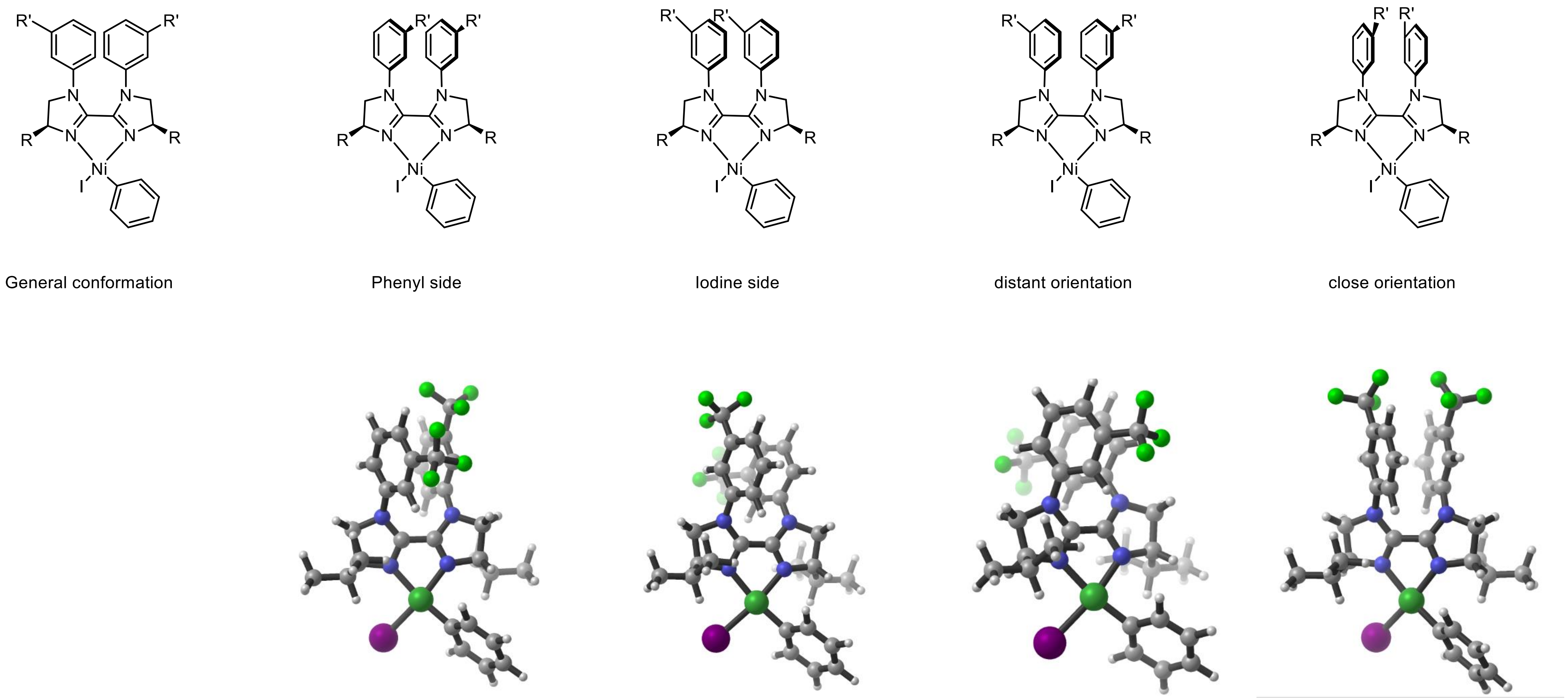

Supplementary Fig. 14 *Conformations considered for BiIm. All the conformations showed similar models. The conformer chosen was of the 'Iodine side' which was slightly better than the rest.*

Supplementary Fig. 15 *Scope of ligands. The following color code is used for numbering the molecules: ones that are only included in this work are in green and those included in this work and Doyle 2021*[28] *are in purple, current work (ee% in black) and Doyle 2021 (ee% in blue).*

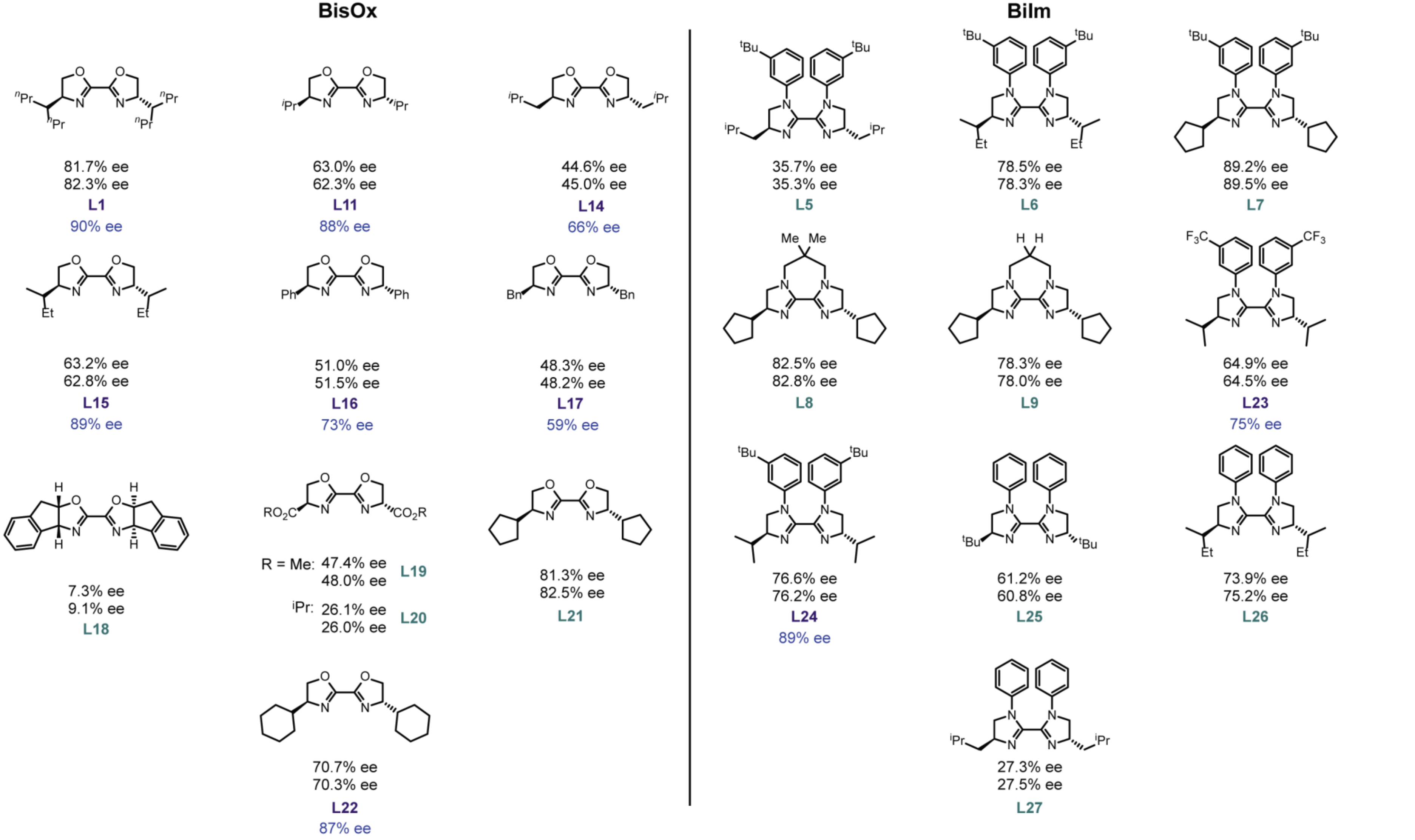

Supplementary Fig. 16 *Ligands – Doyle, 2021*

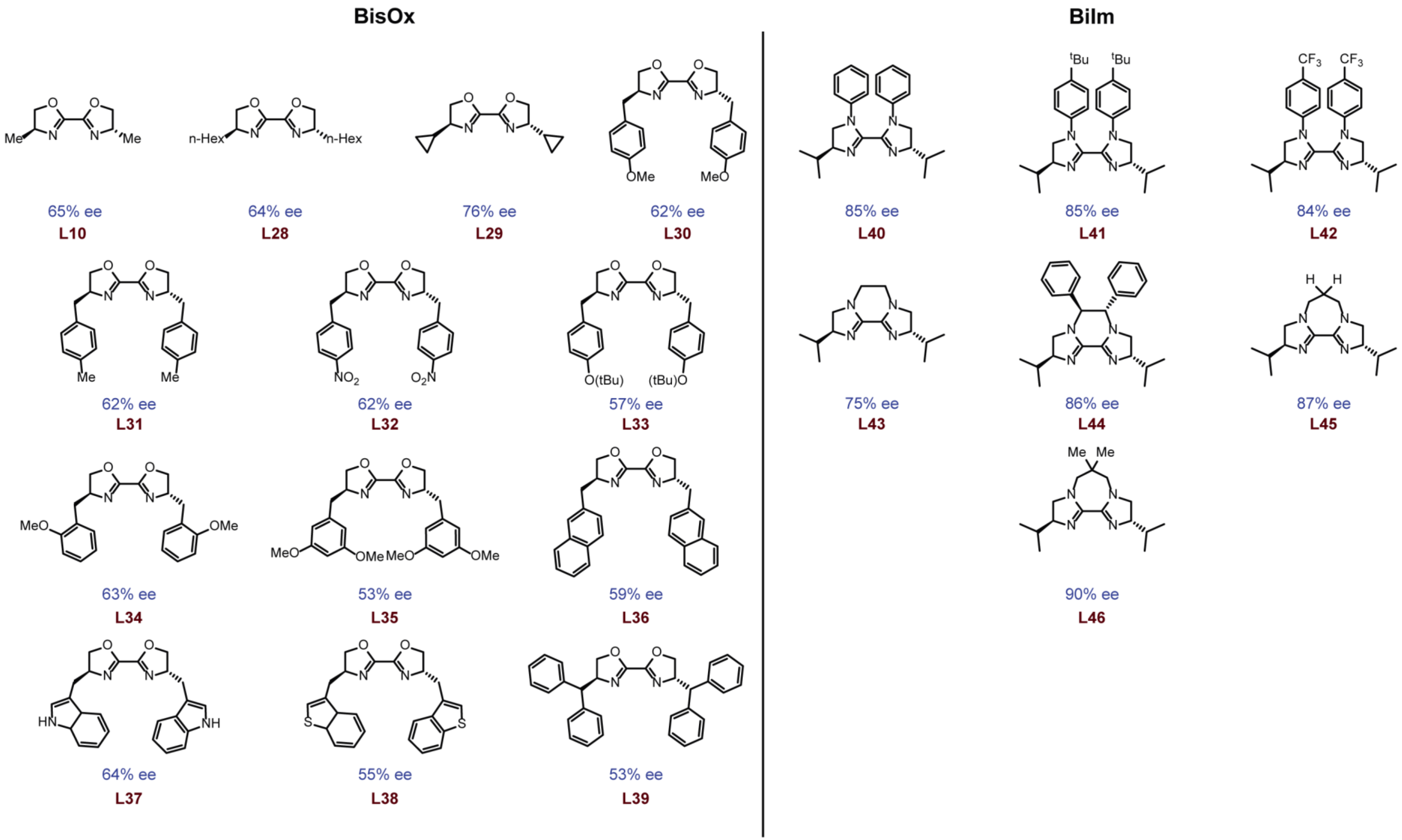

Supplementary Fig. 17 *Yield and reduction potentials of different ligands*

	Yield(%)	E_p1	E_p2	E_p3
L10(Biox-Me)	<5%	-1.62V	/	/
L11(Biox-*i*Pr)	<5%	-1.72V	-2.06V	/
L12(Biox-*t*Bu)	<5%	-1.65V	-2.09V	/
L14(Biox-CH_2^{i}Pr)	<5%	-1.57V	-1.96V	/
L15(Biox-*s*Bu)	<5%	-1.43V	-1.87V	/
L16(Biox-Ph)	<5%	-1.68V	-1.92V	/
L17(Biox-Bn)	<5%	-1.69V	-1.93V	/
L18(Biox-Indyl)	<5%	-1.51V	-1.96V	/
L19(Biox-CO_2Me)	<5%	-1.54V	-1.93V	/
L20(Biox-CO_2iPr)	<5%	-1.52V	-1.94V	/
L21(Biox-cyclopentyl)	<5%	-1.76V	-2.08V	/
L22(Biox-Cy)	<5%	-1.38V	-1.67V	-2.08V
L1 (Biox-4Heptyl)	<5%	-1.73V	-2.11V	/
L23(BilM-*i*Pr-mCF_3Ph)	42%	-1.50V	-1.95V	-2.47V
L24(BilM-*i*Pr-m^{t}BuPh)	25%	-1.42V	-2.16V	/
L25(BilM-*t*Bu-Ph)	<5%	-1.36V	-1.67V	-2.12V
L26(BilM-*s*Bu-Ph)	70%	-1.46V	-2.13V	/
L27 (BilM-CH_2*i*Pr-Ph)	30%	-1.48%	-2.11V	/
L7(BilM- cyclopentyl - m^{t}BuPh)	97%	-1.70V	-2.20V	/

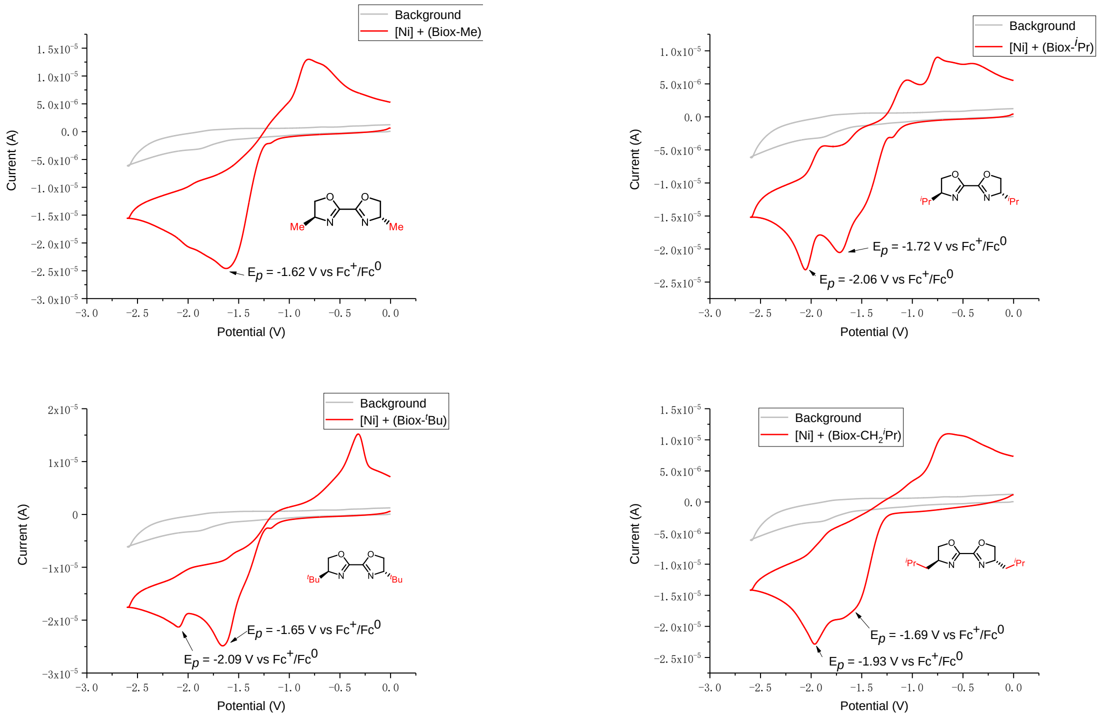

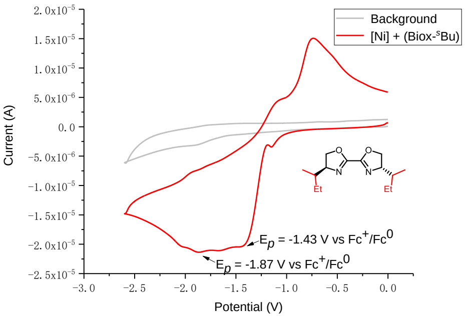
Background
[Ni] + (Biox-^{s}Bu)
E_p = -1.43 V vs Fc^+/Fc^0
E_p = -1.87 V vs Fc^+/Fc^0
Current (A)
Potential (V)

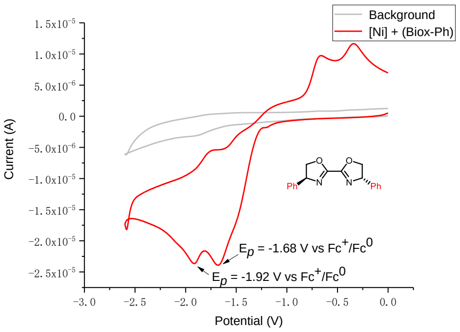
Background
[Ni] + (Biox-Ph)
E_p = -1.68 V vs Fc^+/Fc^0
E_p = -1.92 V vs Fc^+/Fc^0
Current (A)
Potential (V)

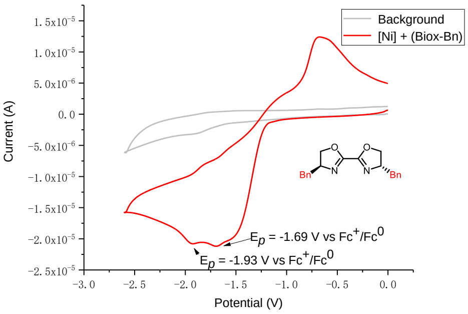
Background
[Ni] + (Biox-Bn)
E_p = -1.69 V vs Fc^+/Fc^0
E_p = -1.93 V vs Fc^+/Fc^0
Current (A)
Potential (V)

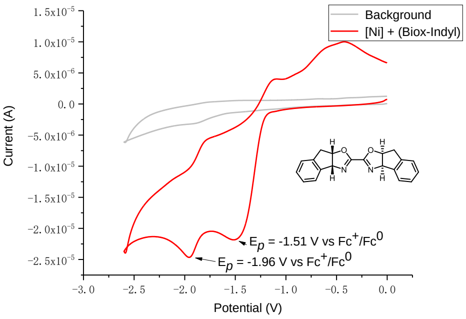
Background
[Ni] + (Biox-Indyl)
E_p = -1.51 V vs Fc^+/Fc^0
E_p = -1.96 V vs Fc^+/Fc^0
Current (A)
Potential (V)

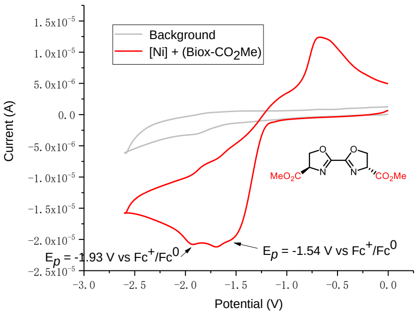
Background
[Ni] + (Biox-CO_2Me)
E_p = -1.93 V vs Fc^+/Fc^0
E_p = -1.54 V vs Fc^+/Fc^0
Current (A)
Potential (V)

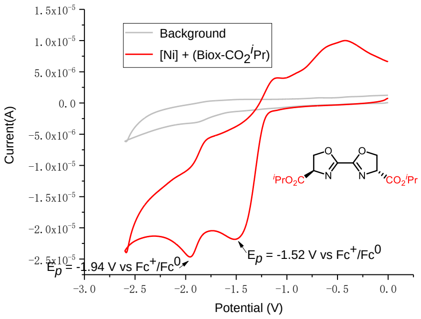
Background
[Ni] + (Biox-$CO_2{}^i$Pr)
E_p = -1.94 V vs Fc^+/Fc^0
E_p = -1.52 V vs Fc^+/Fc^0
Current(A)
Potential (V)

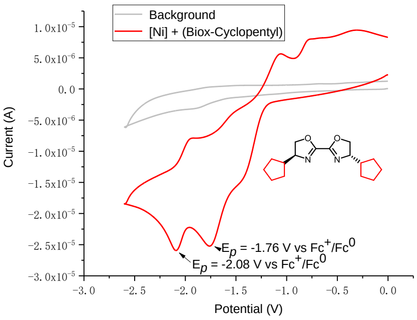
Background
[Ni] + (Biox-Cyclopentyl)
E_p = -1.76 V vs Fc^+/Fc^0
E_p = -2.08 V vs Fc^+/Fc^0
Current (A)
Potential (V)

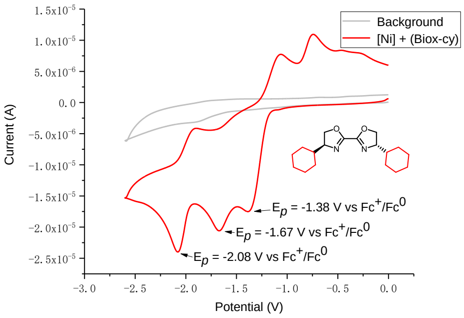
Background
[Ni] + (Biox-cy)
E_p = -1.38 V vs Fc^+/Fc^0
E_p = -1.67 V vs Fc^+/Fc^0
E_p = -2.08 V vs Fc^+/Fc^0
Current (A)
Potential (V)

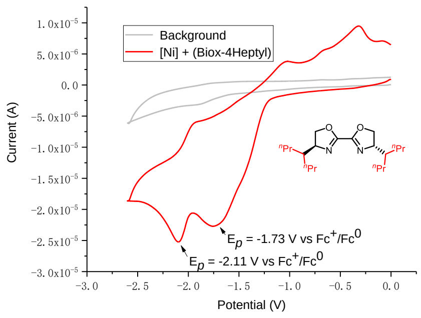
Background
[Ni] + (Biox-4Heptyl)
Current (A)
Potential (V)
Ep = -1.73 V vs Fc+/Fc0
Ep = -2.11 V vs Fc+/Fc0

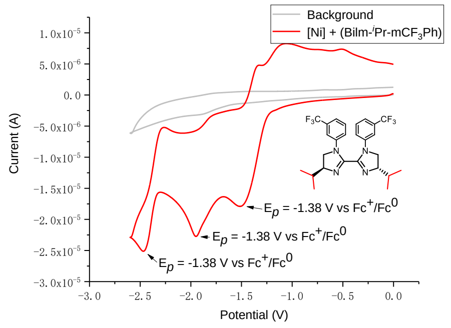
Background
[Ni] + (Bilm-iPr-mCF3Ph)
Current (A)
Potential (V)
Ep = -1.38 V vs Fc+/Fc0
Ep = -1.38 V vs Fc+/Fc0
Ep = -1.38 V vs Fc+/Fc0

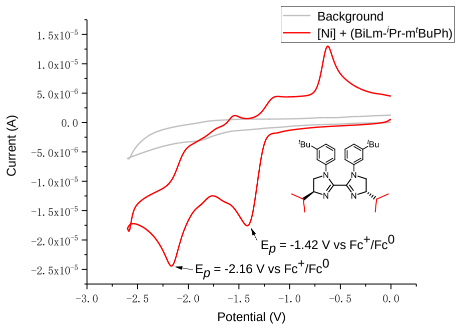
Background
[Ni] + (BiLm-iPr-mtBuPh)
Current (A)
Potential (V)
Ep = -1.42 V vs Fc+/Fc0
Ep = -2.16 V vs Fc+/Fc0

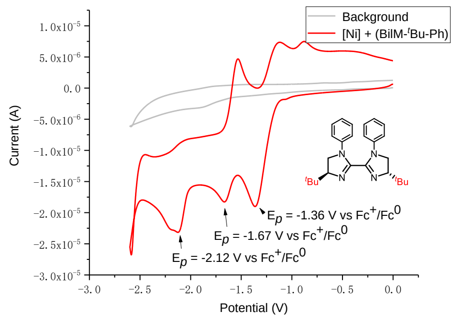
Background
[Ni] + (BilM-tBu-Ph)
Current (A)
Potential (V)
Ep = -1.36 V vs Fc+/Fc0
Ep = -1.67 V vs Fc+/Fc0
Ep = -2.12 V vs Fc+/Fc0

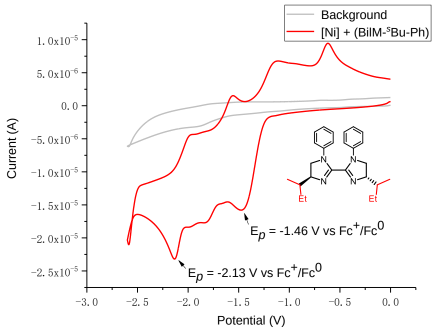
Background
[Ni] + (BilM-sBu-Ph)
Current (A)
Potential (V)
Ep = -1.46 V vs Fc+/Fc0
Ep = -2.13 V vs Fc+/Fc0

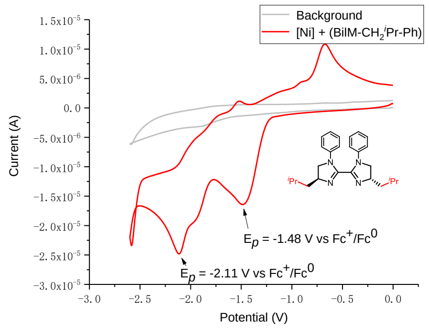
Background
[Ni] + (BilM-CH2iPr-Ph)
Current (A)
Potential (V)
Ep = -1.48 V vs Fc+/Fc0
Ep = -2.11 V vs Fc+/Fc0

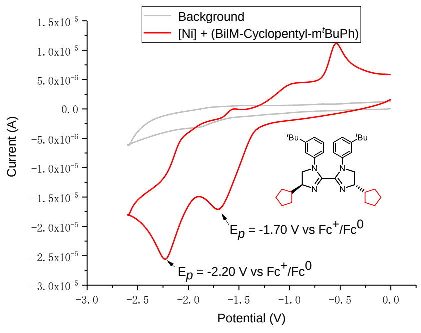
Background
[Ni] + (BilM-Cyclopentyl-mtBuPh)
Current (A)
Potential (V)
Ep = -1.70 V vs Fc+/Fc0
Ep = -2.20 V vs Fc+/Fc0

Models from main text

Given the option, we selected to focus on models with a minimal number of parameters overall but tested all option up to a ratio of 5:1 between experimental examples and the number of parameters in the model.

Current work:

3 parameters

formula	**R.sq**	Q.sq	MAE
ddg ~ d C10−C17 + d C4−C20 + <C25−Ni22−I24	**0.870**	0.787	0.160

Multicollinearity:

	x
d C10−C17	5.904
d C4−C20	6.796
< C25−Ni22−I24	2.073

We fear that this model may be skewed and of limited interpretability due to the high degree of collinearity between parameters

2 parameters

formula	**R.sq**	Q.sq	MAE
ddg ~ d C10−C17 + d C4−C20	**0.835**	0.772	0.178
ddg ~ d C10−C17 + $<_{tor}$C8−C6−Ni22−I24.	**0.789**	0.704	0.196

Multicollinearity:

	x
d C10−C17	3.283
d C4−C20	3.283

Scaled Coefficients:

	Estimate	Std. Error	t value	Pr(>|t|)
(Intercept)	0.898	0.039	22.781	0.00e+00
d C10−C17	0.630	0.073	8.608	1.00e-07
d C4−C20	-0.358	0.073	-4.893	1.17e-04

Plot for 2 parameter model:

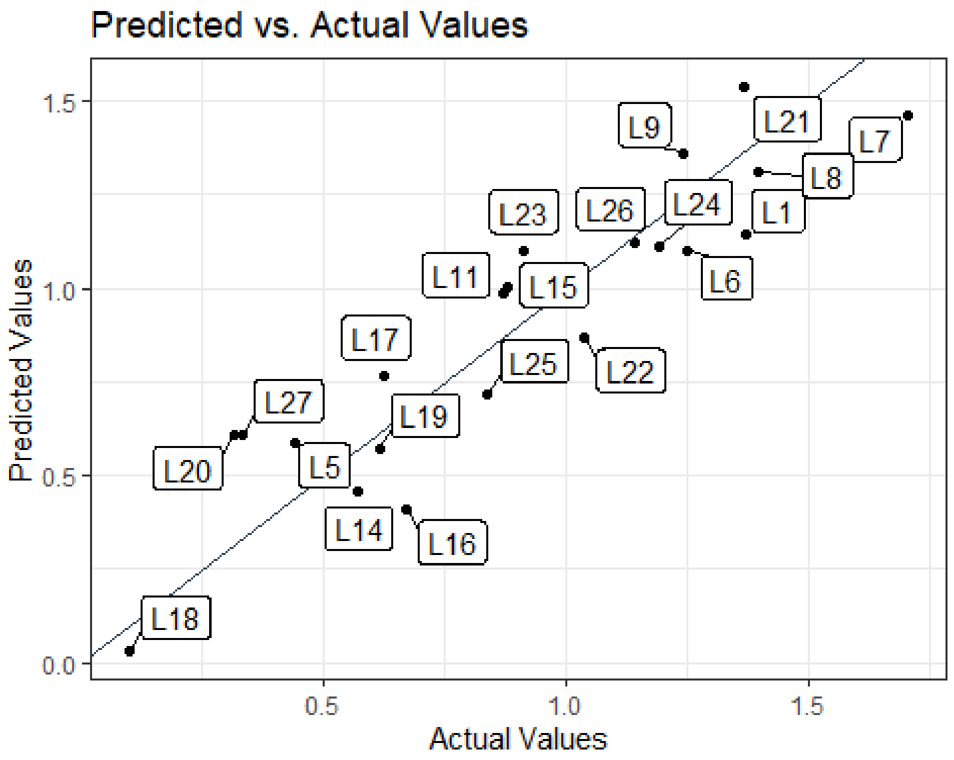

Doyle 2021:

formula	R.sq	Q.sq	MAE
ddg ~ d N5−Ni22 + d Ni22−I24 + NPA Ni22	**0.812**	0.761	0.138
ddg ~ ν C1−N5 + d N5−Ni22 + $<_{tor}$ N5−C1−C8−N6.	**0.752**	0.691	0.155

Multicollinearity

	x
d N5−Ni22	1.155
d Ni22−I24	2.531
NPA Ni22	2.550

Scaled Coefficients:

	Estimate	Std. Error	t value	Pr(>|t|)
(Intercept)	1.165	0.032	36.880	0e+00
d N5−Ni22	0.331	0.035	9.572	0e+00
d Ni22−I24	0.350	0.051	6.850	4e-07
NPA Ni22	-0.354	0.051	-6.887	3e-07

Plot:

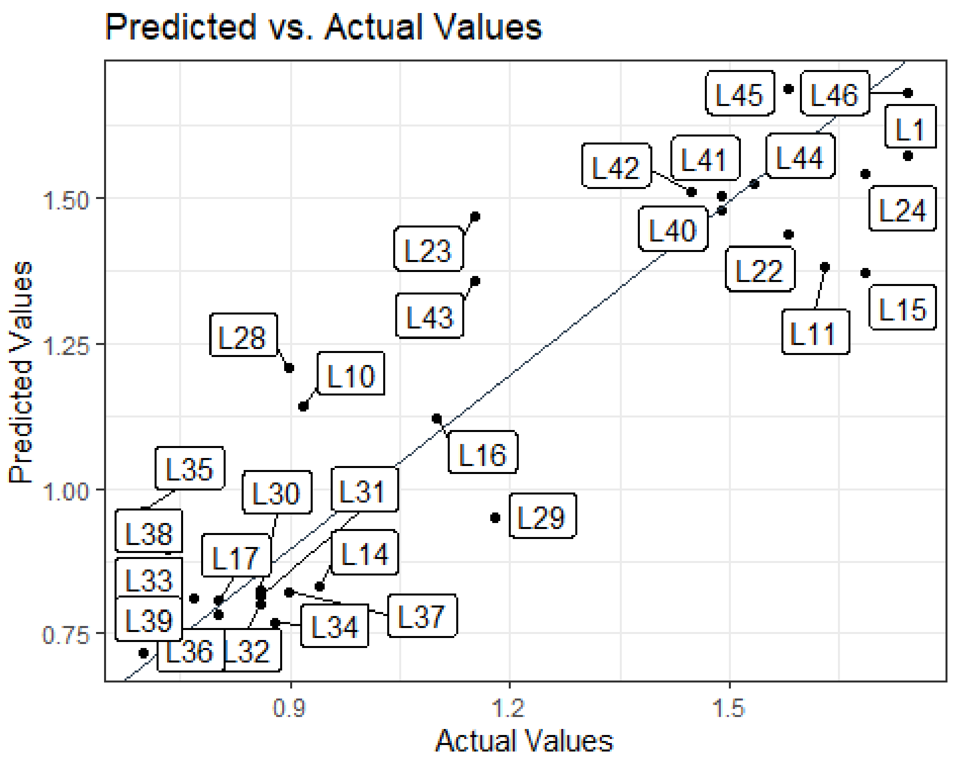

Additional Comparisons

To probe whether the difference between the models stems from the reaction conditions or from the difference in the ligands included to produce each model, we performed a few additional comparisons. We also added a third set from the Doyle group from 2017 that includes an aziridine substrate to try to gauge whether the differences stem merely from the reaction conditions or from the requirements of the substrate.[30] **Only common ligands were considered for each comparison**, which led to different models in each comparison, and limited our statistical ability to use all the parameters required to describe the variance in each set. However, at the same time, this limited comparison provided some indication on whether the set of ligands, the substrates reacted, or the reaction conditions are at the origin of the differences between the models.

Reaction modeled from this work

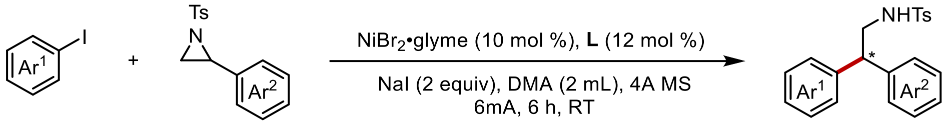

Reaction modeled from Doyle 2021[28]

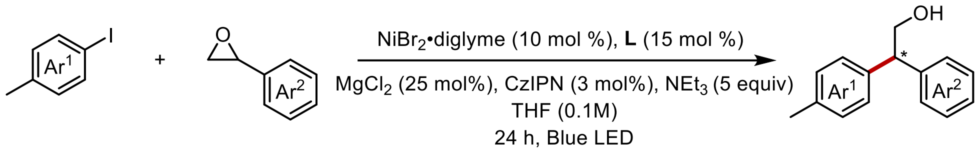

Reaction modeled from Doyle 2017[30]

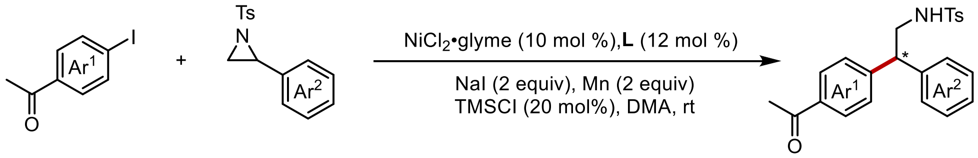

Comparing current work and Doyle 2021

L1, L10, L11, L14, L15, L16, L17, L22, L23, L24

Current work:

formula	**R.sq**	Q.sq	MAE
ddg ~ d N5−Ni22 + Iso polarizability	**0.731**	0.621	0.096

Models with high multicollinearity were removed

Multicollinearity

	x
d N5−Ni22	1.062
Iso polarizability	1.062

Scaled Coefficients:

	Estimate	Std. Error	t value	Pr(>|t|)
(Intercept)	0.878	0.049	18.007	0.0000004
d N5−Ni22	0.179	0.053	3.385	0.0116765
Iso polarizability	0.184	0.053	3.482	0.0102426

Doyle 2021:

formula	**R.sq**	Q.sq	MAE
ddg ~ d N5−Ni22 + Iso polarizability	**0.747**	0.535	0.227

Multicollinearity

	x
d N5−Ni22	1.062219
Iso polarizability	1.062219

Scaled Coefficients:

	Estimate	Std. Error	t value	Pr(>|t|)
(Intercept)	1.323	0.067	19.627	0.0000002
d N5−Ni22	0.317	0.073	4.334	0.0034226
Iso polarizability	0.175	0.073	2.385	0.0485180

This comparison reveals models with the same parameters for both sets. Yet, the coefficient of the distance between the metal and the ligand in the Doyle 2021 set is almost doubled. As this distance is considered an electronic parameter the subtle difference between the models may still allude to a more electronic character in the photochemically assisted reaction. Specifically, the electronic character of the ligand in Doyle 2021 would have a stronger impact on the enantioselectivity of the reaction. Still, given that the same parameters appear in both cases and the moderate goodness-of-fit, this result is inconclusive as to whether the difference between the models in both cases stems from the set of ligands included in each model or is an indication of the requirements from the ligands under the specific reaction conditions.

Comparing current work and Doyle 2017

L1, L10, L11, L17, L19, L21, L22

Current work:

formula	**R.sq**	Q.sq	MAE
ddg ~ d C1−C8	**0.831**	0.744	0.138

Scaled Coefficients:

	Estimate	Std. Error	t value	Pr(>|t|)
(Intercept)	0.934	0.057	16.481	0.0000150
d C1−C8	-0.304	0.061	-4.965	0.0042296

Abigail Doyle, 2017

formula	**R.sq**	Q.sq	MAE
ddg ~ NPA Ni22	**0.683**	0.526	0.159

Scaled Coefficients:

	Estimate	Std. Error	t value	Pr(>|t|)
(Intercept)	0.876	0.062	14.096	0.0000323
NPA Ni22	0.220	0.067	3.285	0.0218314

A correlation with the charge on the metal was identified for the 2017 set from Doyle. This feature is of clear electronic character compared to the ligand C−C distance for the same ligands in the current work. Since in both cases the substrate used was an aziridine, this comparison indicates that the difference in parameters between the models stem from the different conditions: in the current work an electrochemical reduction was used to turn over the catalyst, whereas in Doyle 2017, Mn was used as a stoichiometric reductant. Again alluding to the steric requirement from the ligands under the current electrochemical conditions.

Comparing Doyle 2017 and 2021

L1, L10, L11, L17, L22, L28, L29, L30, L39

Doyle 2017

formula	**R.sq**	Q.sq	MAE
ddg ~ NPA Ni22	**0.850**	0.740	0.121

Scaled Coefficients:

	Estimate	Std. Error	t value	Pr(>|t|)
(Intercept)	0.679	0.045	15.166	0.0000013
NPA Ni22	0.299	0.0475	6.295	0.0004063

Doyle 2021

formula	**R.sq**	Q.sq	MAE
ddg ~ NPA Ni22	**0.595**	0.405	0.284

Scaled Coefficients:

	Estimate	Std. Error	t value	Pr(>|t|)
(Intercept)	1.145	0.091	12.567	0.0000047
NPA Ni22	0.310	0.097	3.209	0.0148796

Whereas both sets are modeled using the same parameter, namely the charge on the metal center, the set from 2017 reveals a better correlation in terms of goodness-of-fit. The set from 2017 refers to a reaction with an aziridine compared to a reaction with an epoxide in the set from 2021. The poor fit for Doyle 2021 indicates there are additional effects that are not captured by the charge on the nickel. Nevertheless, this comparison further highlights that it does not seem to be the nature of the substrate dictating the required parameters, but rather the conditions.

Yield in current work

The yield of reaction with different ligands can be described by the vibration of the bond connecting carbon 1 and nitrogen 5.
We tested an additional basis set and functionals that may provide a more accurate description of vibrational frequencies in molecules containing metal atoms.

M06-2X functional and Def2-SVP

formula	R.sq	Q.sq	MAE
Yield ~ ν C1-N5	0.803	0.689	16.349

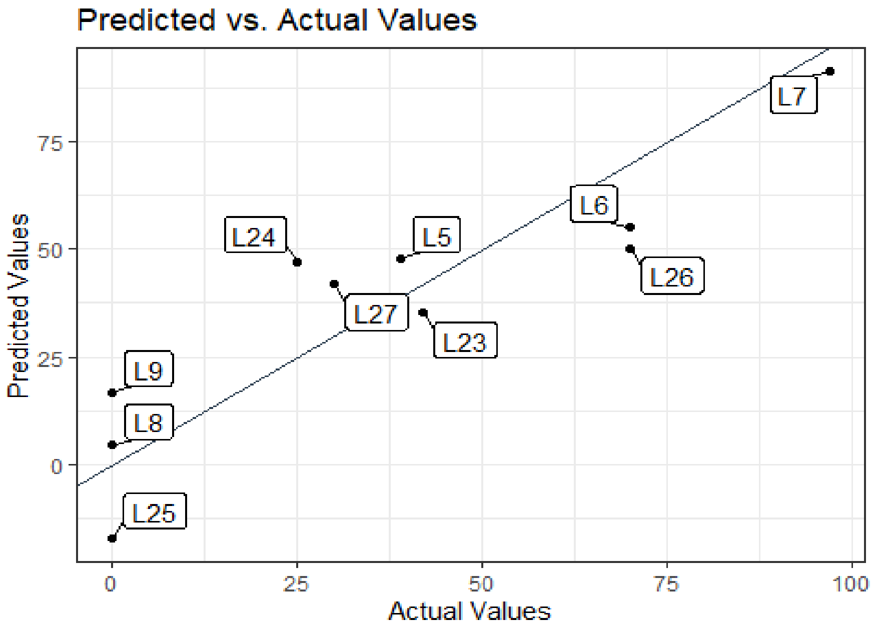

M06-2X functional and Def2-TZVP

formula	R.sq	Q.sq	MAE
Yield ~ ν C1-N5	0.719	0.583	19.462

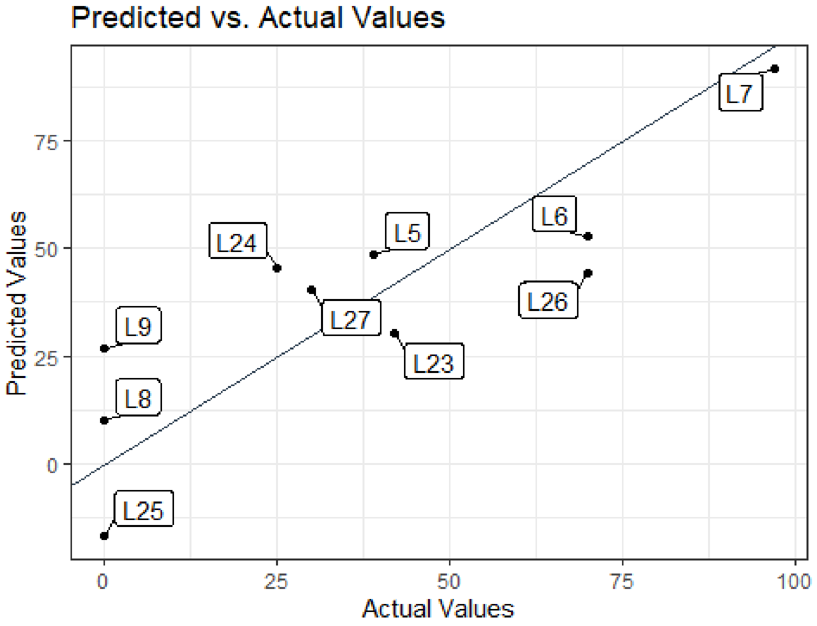

MN15 functional and Def2-TZVP

formula	R.sq	Q.sq	MAE
Yield ~ ν C1-N5	0.767	0.689	14.509

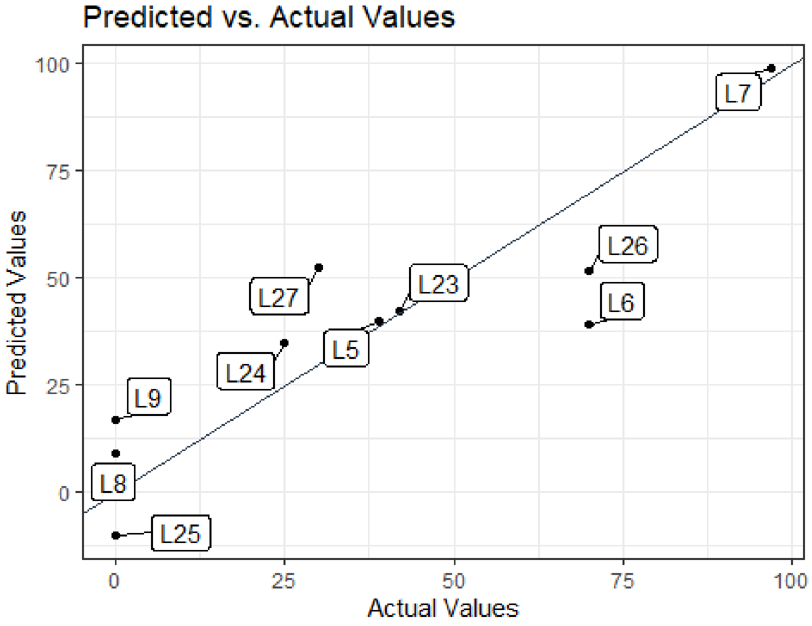

Distinction between BiIm and BisOx ligands using different parameters:

NPA C1:

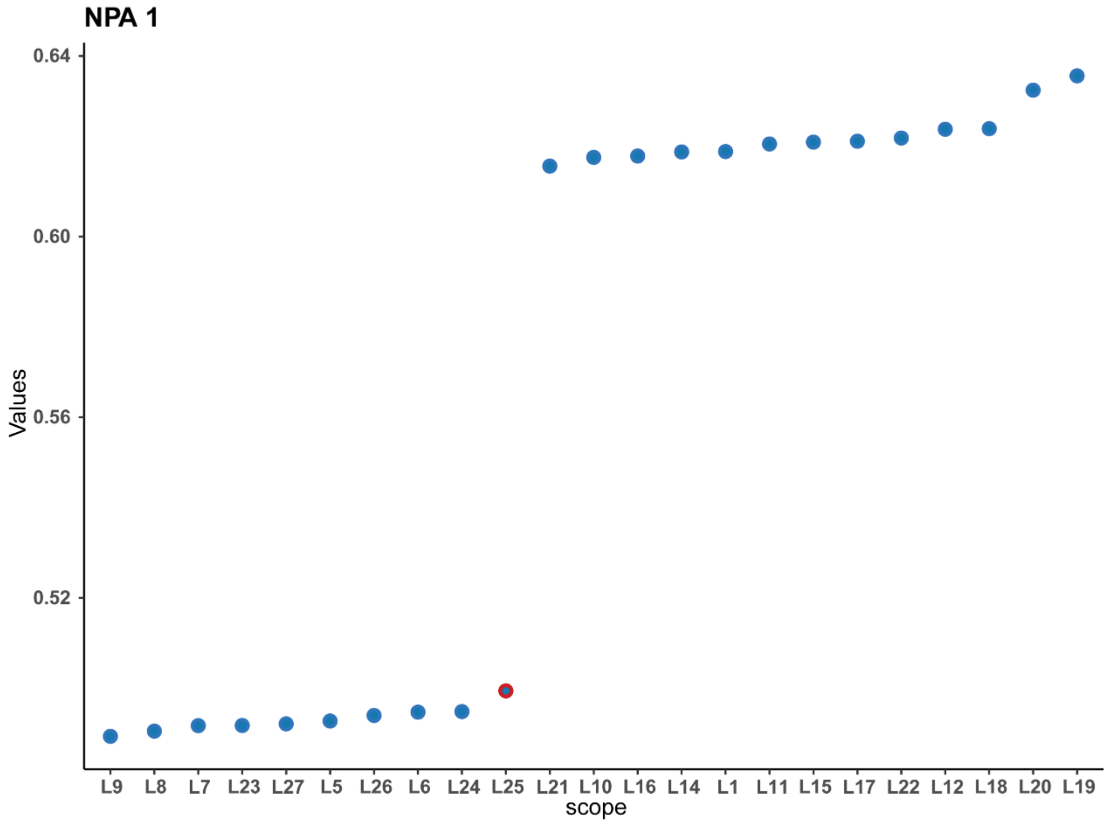

Supplementary Fig. 18 Separation of BiIM and BiOx by parameters (NPA C1).

ν C1-N5

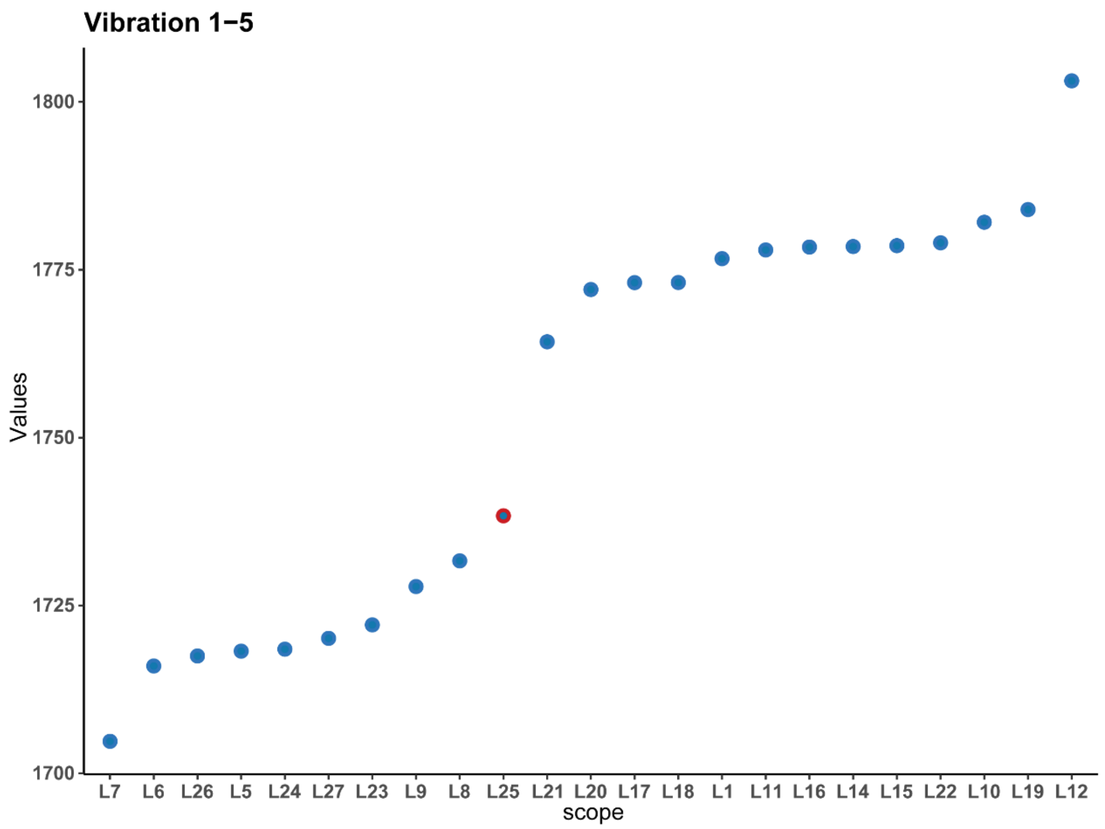

Supplementary Fig. 19 Separation of BiIM and BiOx by parameters (vibration 1-5).

Data

Data is provided in an excel attached to this paper, or at the following GitHub link:
https://github.com/Milo-group/Tian-ShengMei2022Collab
at the Excel_Files folder

XYZ coordinates for the optimized geometry

Data is provided at the following GitHub link:
https://github.com/Milo-group/Tian-ShengMei2022Collab
at the XYZ folder

References:

(1) Frisch, M. J.; Trucks, G. W.; Schlegel, H. B.; Scuseria, G. E.; Robb, M. A.; Cheeseman, J. R.; Scalmani, G.; Barone, V.; Petersson, G. A.; Nakatsuji, H.; Li, X.; Caricato, M.;

Marenich, A. V.; Bloino, J.; Janesko, B. G.; Gomperts, R.; Mennucci, B.; Hratch, D. J. Gaussian, Inc., Wallingford CT. **2016**.
(2) Zhao, Y.; Truhlar, D. G.; Zhao, Y.; Truhlar, D G. The M06 Suite of Density Functionals for Main Group Thermochemistry, Thermochemical Kinetics, Noncovalent Interactions, Excited States, and Transition Elements: Two New Functionals and Systematic Testing of Four M06-Class Functionals and 12 Other Functionals. *Theoretical Chemistry Accounts 2007 120:1* **2007**, *120* (1), 215–241.
(3) Valero, R.; Gomes, J. R. B.; Truhlar, D. G.; Illas, F. Good Performance of the M06 Family of Hybrid Meta Generalized Gradient Approximation Density Functionals on a Difficult Case: CO Adsorption on MgO(001). *Journal of Chemical Physics* **2008**, *129* (12).
(4) R Core Team. R: A Language and Environment for Statistical Computing. **2020**.
(5) RStudio Team. Integrated Development for R. RStudio, Inc. **2019**.
(6) Friendly, M.; Fox, J.; Chalmers, P. Matlib: Matrix Functions for Teaching and Learning Linear Algebra and Multivariate Statistics. **2020**.
(7) Borchers, H. W. Pracma: Practical Numerical Math Functions. **2021**, p R package version 2.3.3.
(8) Hadley Wickham. Stringr: Simple, Consistent Wrappers for Common String Operations. **2019**, p R package version 1.4.0.
(9) Yihui Xie. Knitr: A General-Purpose Package for Dynamic Report Generation in R. **2021**, p R package version 1.31.
(10) Wickham, H. *Ggplot2: Elegant Graphics for Data Analysis*; Springer-Verlag New York, **2016**.
(11) Wickham, H.; Francois, R.; Henry, L.; Muller, K. Dplyr: A Grammar of Data Manipulation. **2021**, p R package version 1.0.5.
(12) Dowle, M.; Srinivasan, A. Data.Table: Extension of `data.Frame`. **2021**, p R package version 1.14.0.
(13) Hadley Wickham. The Split-Apply-Combine Strategy for Data Analysis. *J Stat Softw* **2011**, *40*, 1–29.
(14) Max Kuhn. Caret: Classification and Regression Training. **2020**, p R package version 6.0-86.
(15) Muller, K.; Wickham, H. Tibble: Simple Data Frames. **2021**, p R package version 3.1.0.
(16) Wickham, H.; Dana, S. Scales: Scale Functions for Visualization. **2019**, p R package version 1.1.0.
(17) Wickham, H. Reshaping Data with the {reshape} Package. *J Stat Softw* **2007**, *21*, 1–20.
(18) Wickham, H.; Henry, L. Tidyr: Tidy Messy Data. **2020**, p R package version 1.1.1.
(19) Venables, W. N.; Ripley, B. D. *Modern Applied Statistics with S*, Fourth.; Springer: New York, **2002**.
(20) Kamil Slowikowski. Ggrepel: Automatically Position Non-Overlapping Text Labels with “Ggplot2.” **2021**.
(21) John Fox; Sanford Weisberg. An {R} Companion to Applied Regression. Sage: Thousand Oaks {CA} **2019**.
(22) Max Kuhn. Caret: Classification and Regression Training. **2021**.
(23) Adobe Inc. Adobe Illustrator. **2022**.

(24) OriginLab Corporation. *Origin(Pro)* **2022**

(25) Claude Y. Legault. CYLview20. **2020**.
(26) Verloop, A.; Tipker, J. Use of Linear Free Energy Related and Other Parameters in the Study of Fungicidal Selectivity. *Pestic Sci* **1976**, *7*, 379–390.
(27) Verloop, A.; Hoogenstraaten, W.; Tipker, J. *Development and Application of New Steric Substituent Parameters in Drug Design*; Academic Press, Inc., **1976**; Vol. **1962**.
(28) Lau, S. H.; Borden, M. A.; Steiman, T. J.; Wang, L. S.; Parasram, M.; Doyle, A. G. Ni/Photoredox-Catalyzed Enantioselective Cross-Electrophile Coupling of Styrene Oxides with Aryl Iodides. *J. Am. Chem. Soc.* **2021**, *143*, 15873–15881.
(29) Dhayalan, V.; Gadekar, S. C.; Alassad, Z.; Milo, A. Unravelling Mechanistic Features of Organocatalysis with in Situ Modifications at the Secondary Sphere. *Nat. Chem.* **2019**, *11*, 543–551.
(30) Woods, B. P.; Orlandi, M.; Huang, C. Y.; Sigman, M. S.; Doyle, A. G. Nickel-Catalyzed Enantioselective Reductive Cross-Coupling of Styrenyl Aziridines. *J. Am. Chem. Soc.* **2017**, *139*, 5688–5691.

3.2. X-Ray Crystal X-Ray Structures and Data

X-Ray Crystal Structure of 3a (CCDC 2161740)

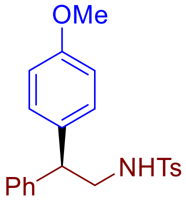

3a

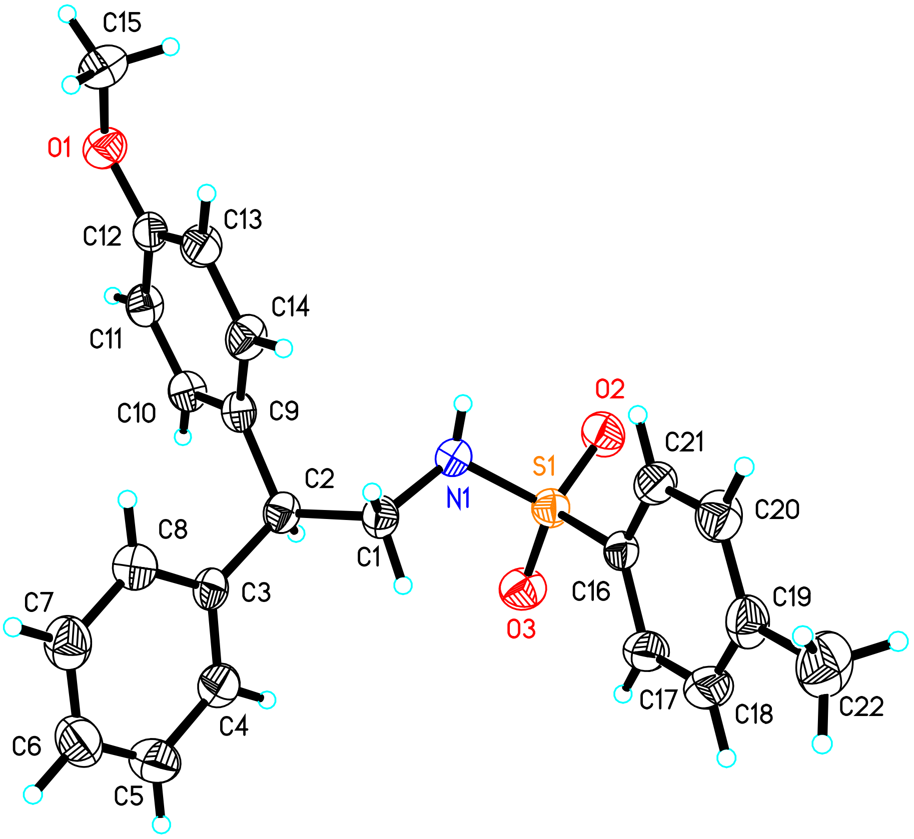

Table 1. Crystal data and structure refinement for **3a**.

Identification code	mo_d8v21365_0m	
Empirical formula	C24 H26 N2 O3 S	
Formula weight	422.53	
Temperature	293(2) K	
Wavelength	0.71073 Å	
Crystal system	Orthorhombic	
Space group	P 21 21 21	
Unit cell dimensions	a = 5.7284(2) Å	α= 90°.
	b = 13.9723(6) Å	β= 90°.
	c = 28.3107(10) Å	γ = 90°.
Volume	2265.96(15) $Å^3$	
Z	4	
Density (calculated)	1.239 Mg/m^3	
Absorption coefficient	0.170 mm^{-1}	
F(000)	896	
Crystal size	0.200 x 0.140 x 0.110 mm^3	
Theta range for data collection	2.604 to 25.998°.	
Index ranges	-6<=h<=7, -14<=k<=17, -34<=l<=30	
Reflections collected	11455	
Independent reflections	4425 [R(int) = 0.0322]	
Completeness to theta = 25.242°	99.4 %	
Absorption correction	Semi-empirical from equivalents	
Max. and min. transmission	0.7456 and 0.6774	
Refinement method	Full-matrix least-squares on F^2	
Data / restraints / parameters	4425 / 0 / 279	
Goodness-of-fit on F^2	1.064	
Final R indices [I>2sigma(I)]	R1 = 0.0402, wR2 = 0.0856	
R indices (all data)	R1 = 0.0556, wR2 = 0.0945	
Absolute structure parameter	0.03(4)	
Extinction coefficient	0.019(3)	
Largest diff. peak and hole	0.138 and -0.180 e.$Å^{-3}$	

X-Ray Crystal Structure of L7 (CCDC 2161735)

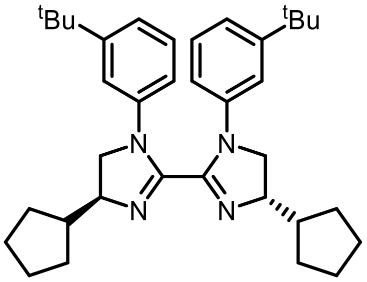

L7

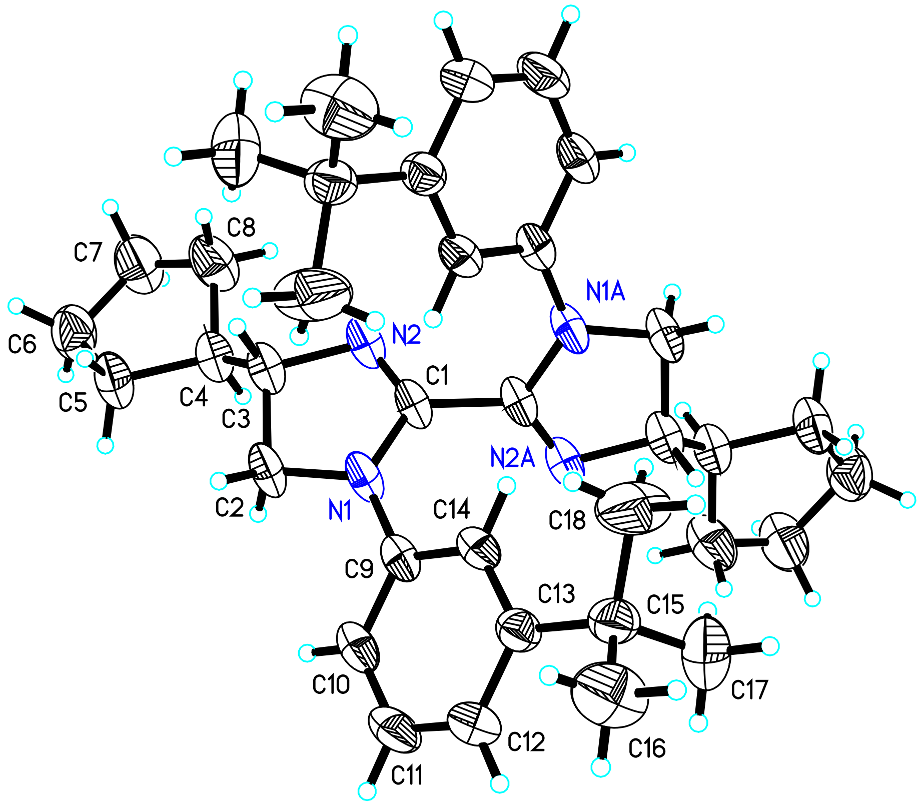

Table 1. Crystal data and structure refinement for **L7**.

Identification code	cu_d8v21312_0m	
Empirical formula	C36 H50 N4	
Formula weight	538.80	
Temperature	293(2) K	
Wavelength	1.54178 Å	
Crystal system	Monoclinic	
Space group	C 2	
Unit cell dimensions	a = 23.9420(8) Å	α= 90°.
	b = 10.6902(3) Å	β= 101.0980(10)°.
	c = 12.9634(4) Å	γ = 90°.
Volume	3255.87(17) Å^3	
Z	4	
Density (calculated)	1.099 Mg/m^3	
Absorption coefficient	0.487 mm^{-1}	
F(000)	1176	
Crystal size	0.200 x 0.150 x 0.130 mm^3	
Theta range for data collection	3.474 to 67.976°.	
Index ranges	-28<=h<=28, -12<=k<=12, -14<=l<=15	
Reflections collected	27936	
Independent reflections	5766 [R(int) = 0.0373]	
Completeness to theta = 67.679°	97.8 %	
Absorption correction	Semi-empirical from equivalents	
Max. and min. transmission	0.7533 and 0.5503	
Refinement method	Full-matrix least-squares on F^2	
Data / restraints / parameters	5766 / 1 / 367	
Goodness-of-fit on F^2	1.025	
Final R indices [I>2sigma(I)]	R1 = 0.0532, wR2 = 0.1500	
R indices (all data)	R1 = 0.0559, wR2 = 0.1550	
Absolute structure parameter	0.03(14)	
Extinction coefficient	n/a	
Largest diff. peak and hole	0.218 and -0.133 e.Å^{-3}	

3.3 ^{1}H NMR, ^{13}C NMR, ^{19}F NMR

Supplementary Fig. 20. Compound L7 ^{1}H NMR (400 MHz, $CDCl_3$)

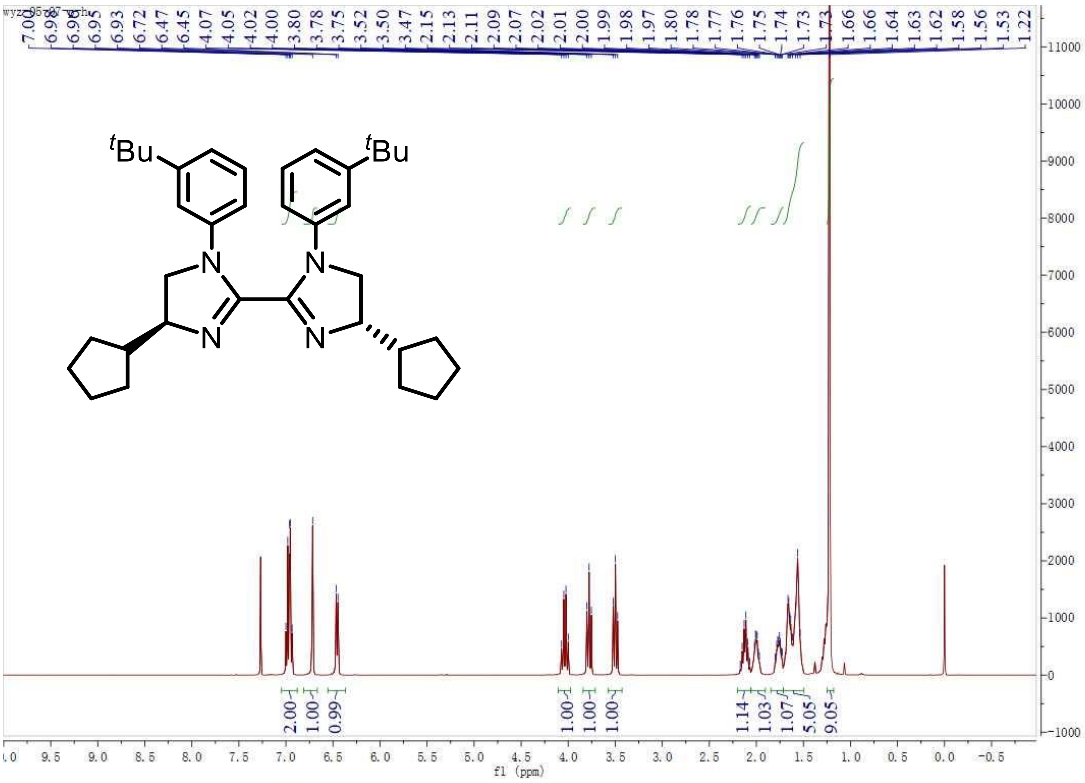

Supplementary Fig. 21. Compound L7 ^{13}C NMR (101 MHz, $CDCl_3$)

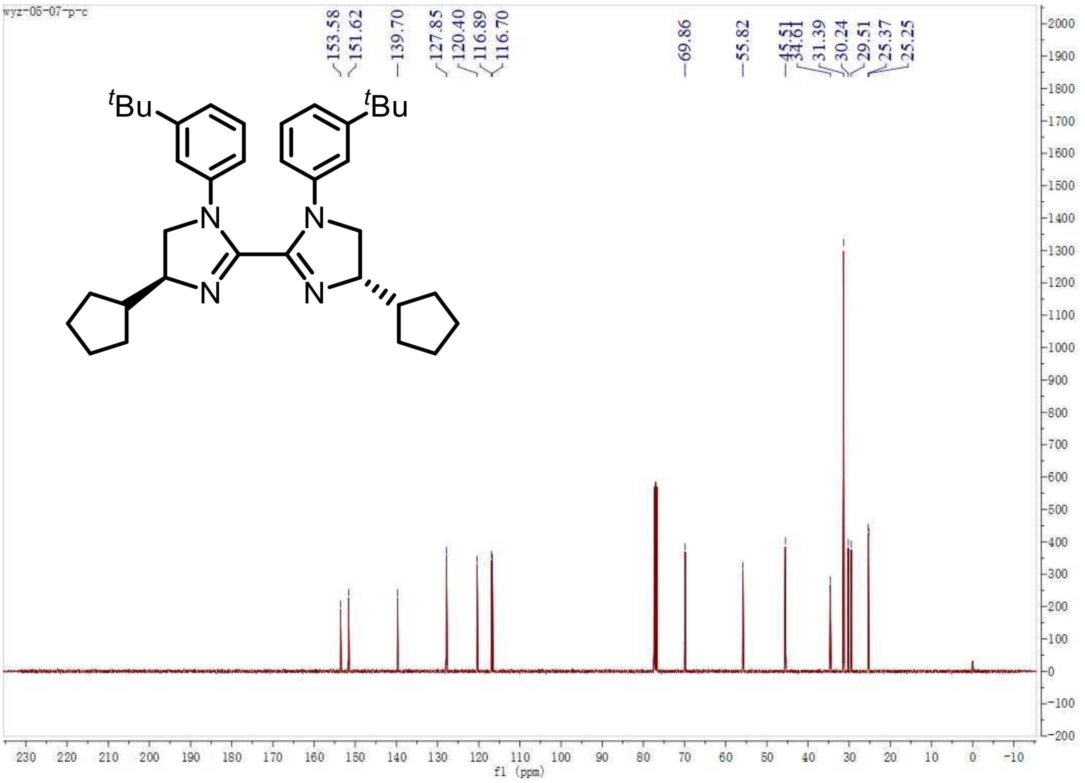

Supplementary Fig. 22. Compound L5 ^{1}H NMR (400 MHz, $CDCl_3$)

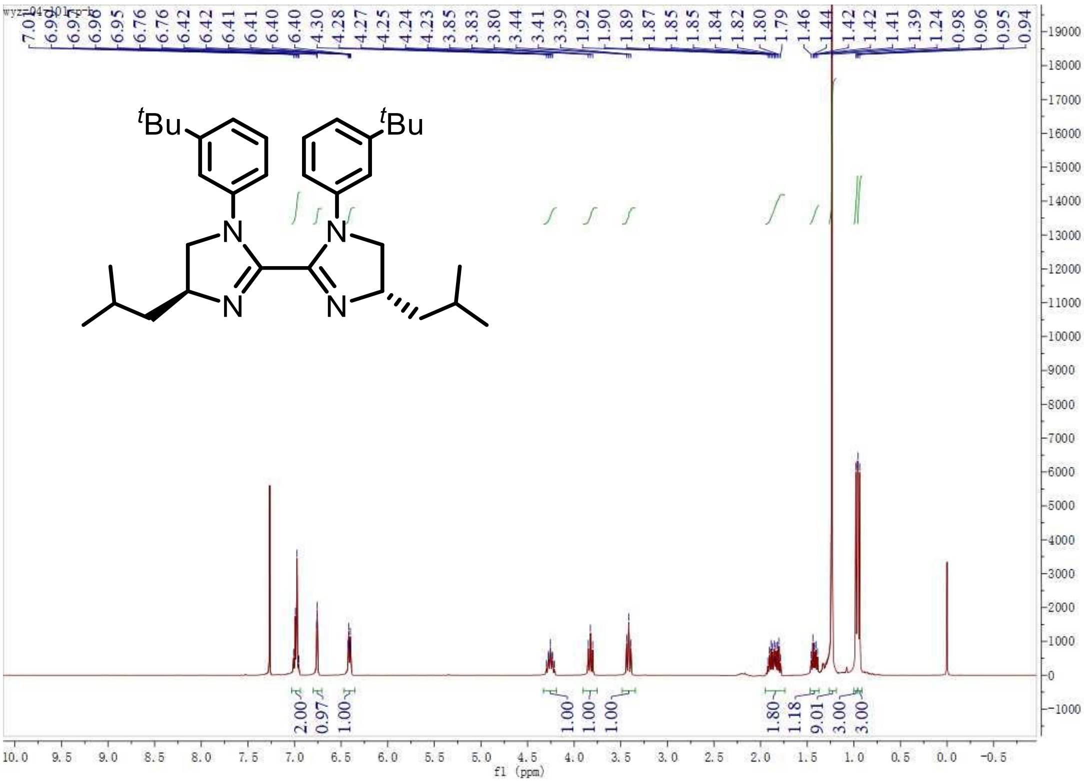

Supplementary Fig. 23. Compound L5 ^{13}C NMR (101 MHz, $CDCl_3$)

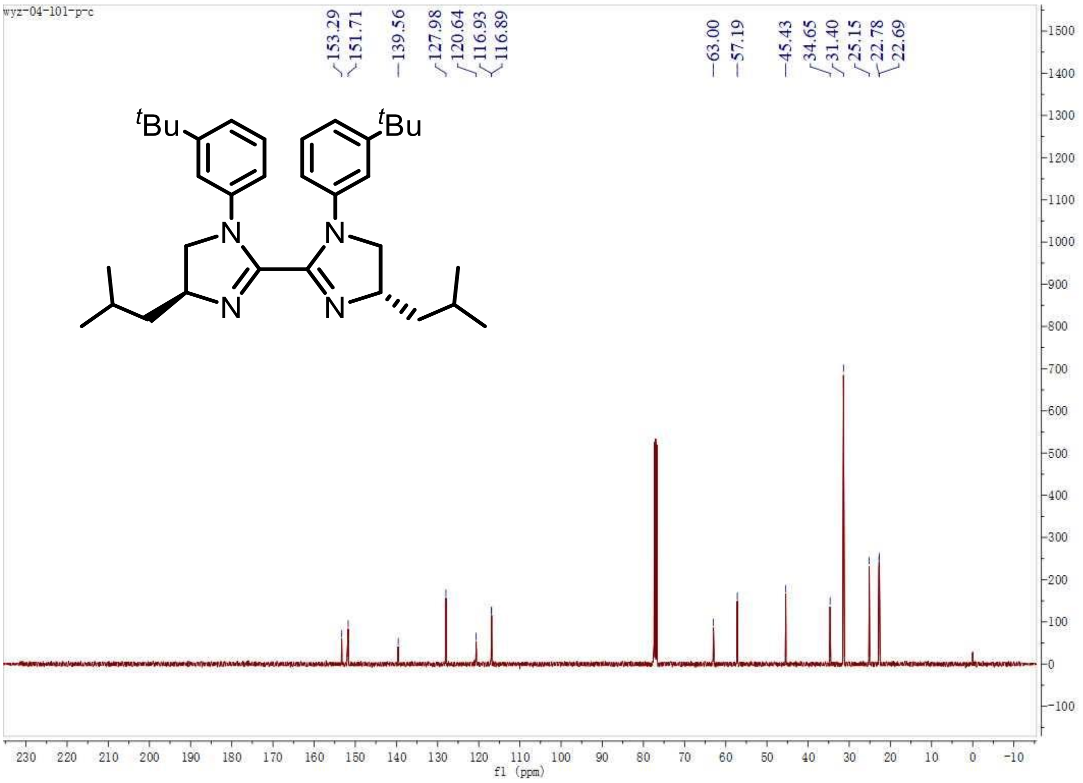

Supplementary Fig. 24. Compound L6 ^{1}H NMR (400 MHz, CDCl$_3$)

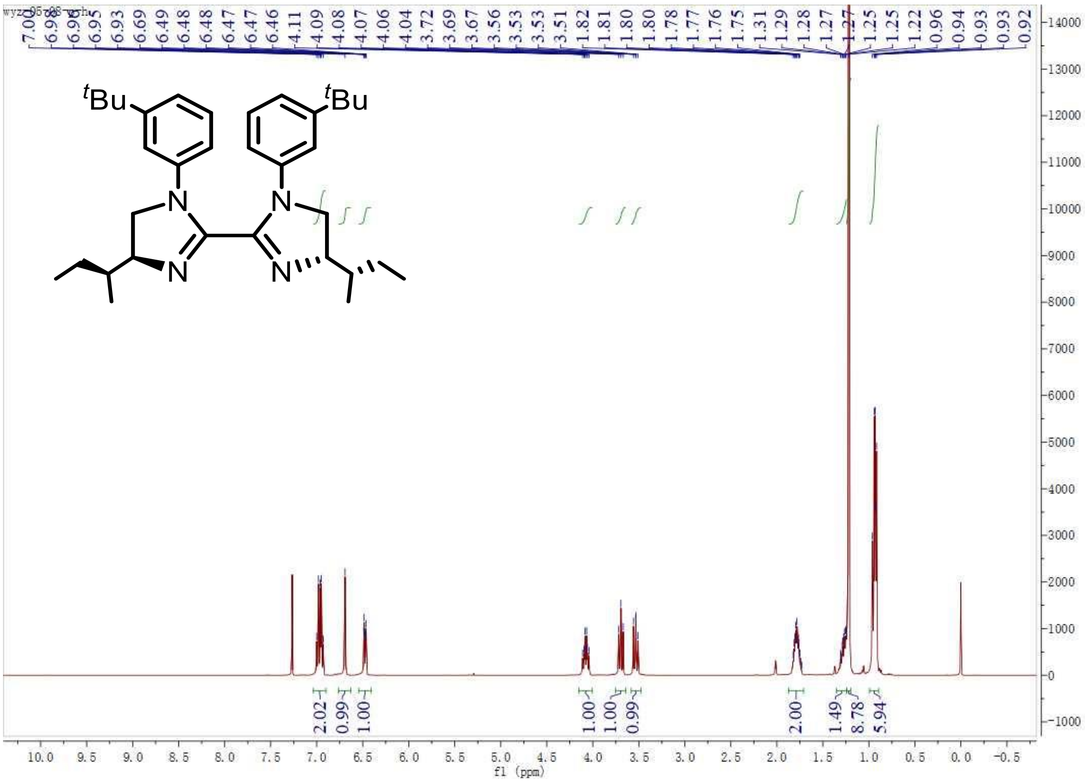

Supplementary Fig. 25. Compound L6 ^{13}C NMR (101 MHz, CDCl$_3$)

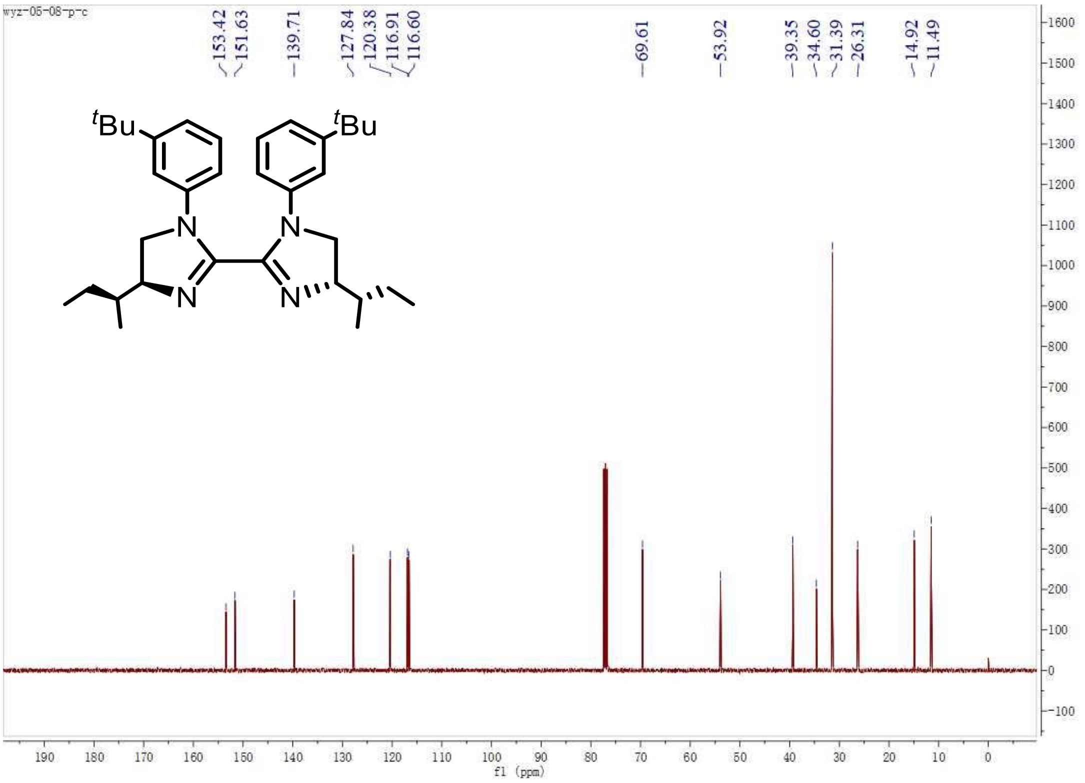

Supplementary Fig. 26. Compound L9 ^{1}H NMR (400 MHz, $CDCl_3$)

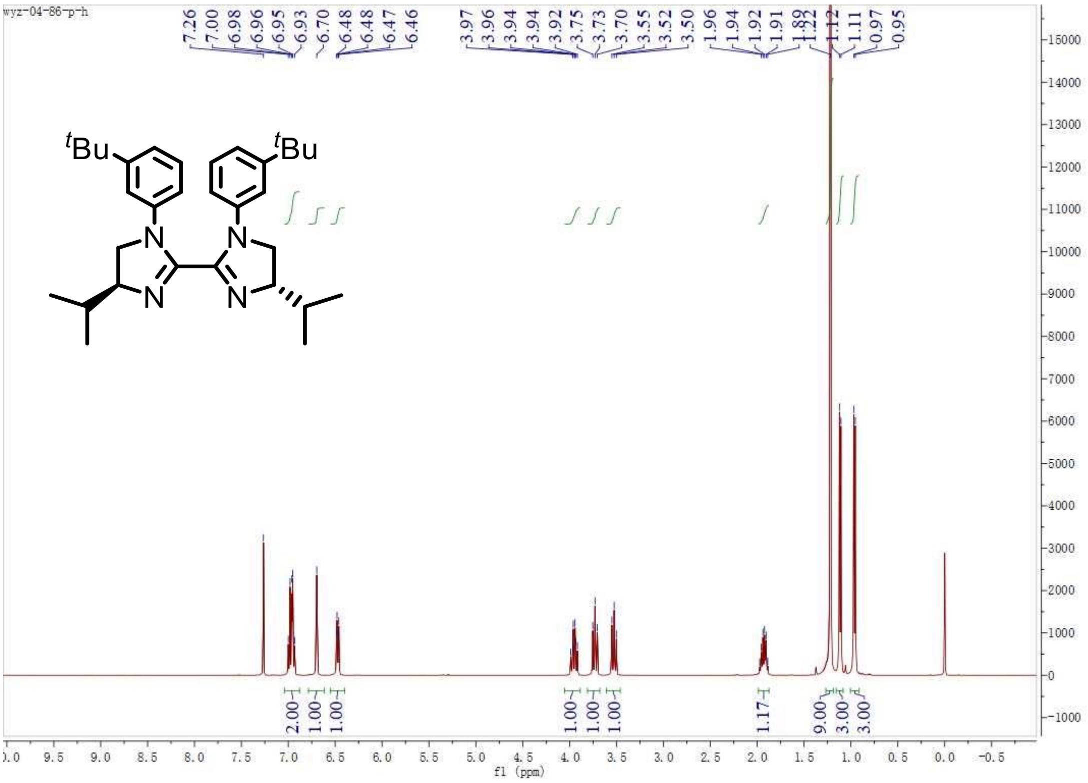

Supplementary Fig. 27. Compound L9 ^{13}C NMR (101 MHz, $CDCl_3$)

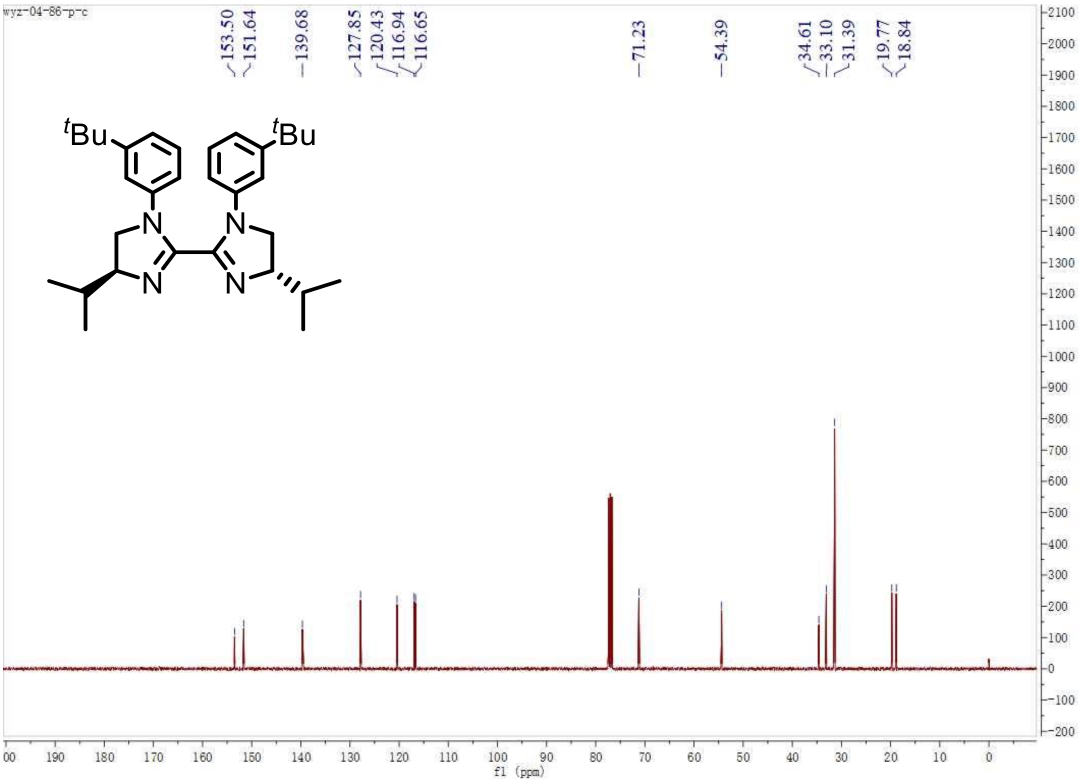

Supplementary Fig. 28. Compound L10 ^{1}H NMR (400 MHz, $CDCl_3$)

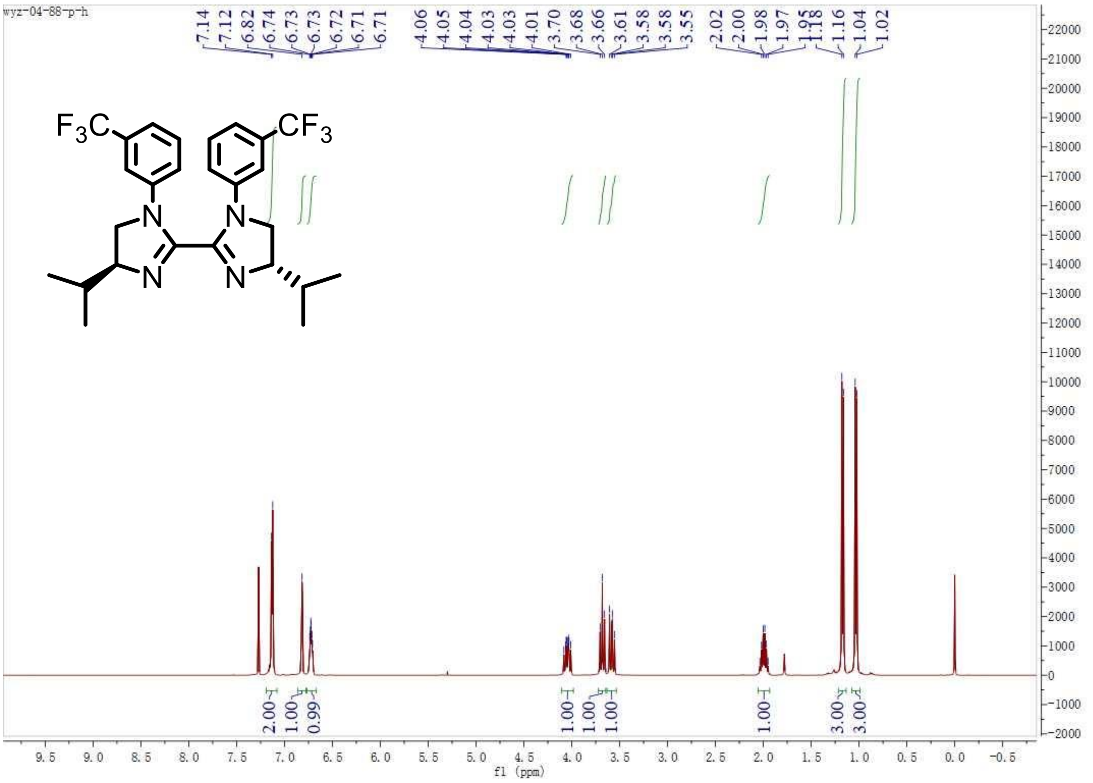

Supplementary Fig. 29. Compound L10 ^{13}C NMR (101 MHz, $CDCl_3$)

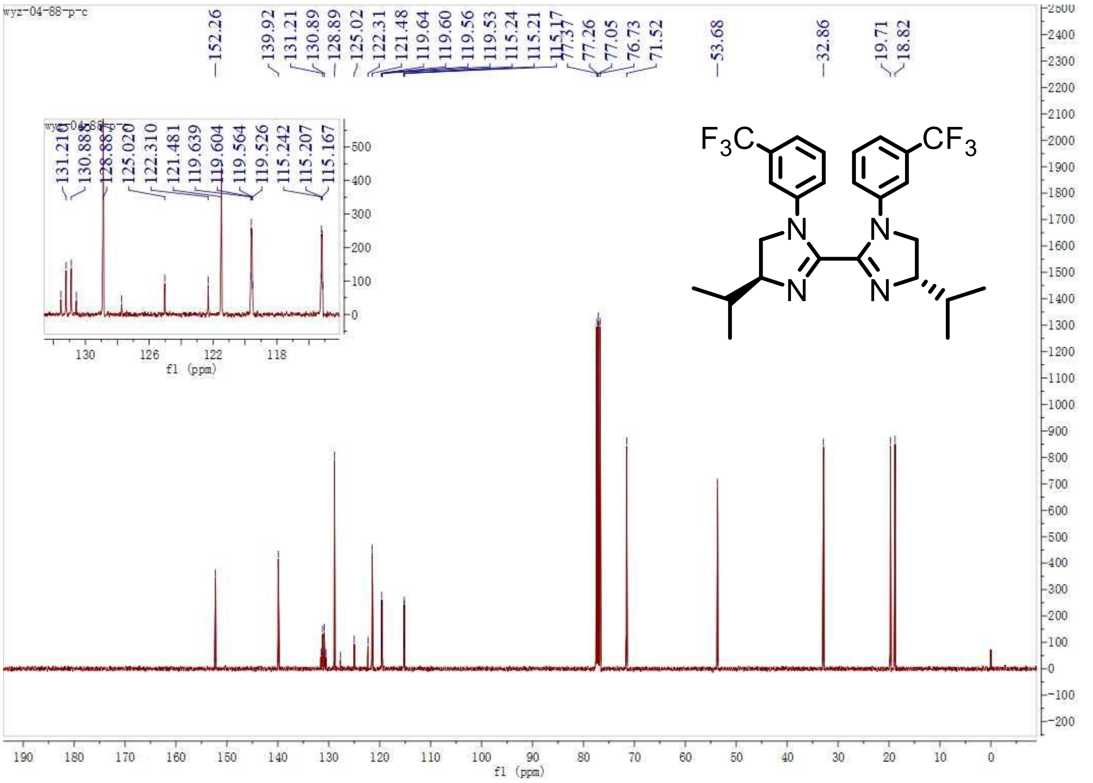

Supplementary Fig. 30. Compound L11 ^{1}H NMR (400 MHz, $CDCl_3$)

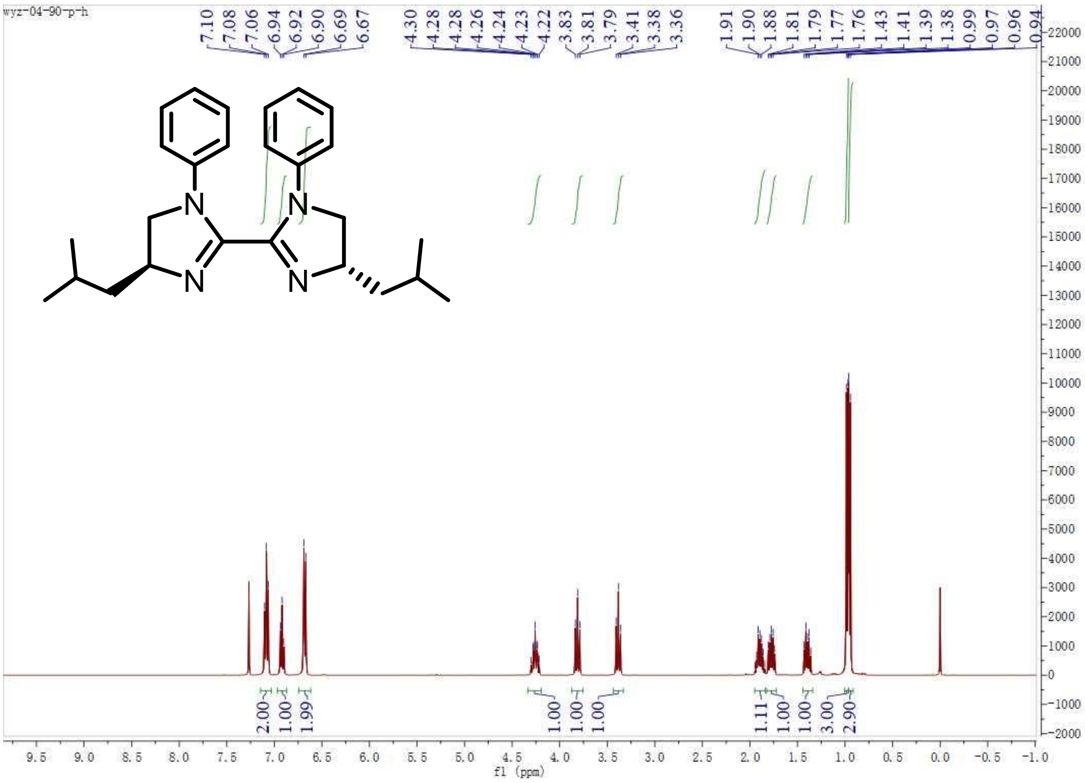

Supplementary Fig. 31. Compound L11 ^{13}C NMR (101 MHz, $CDCl_3$)

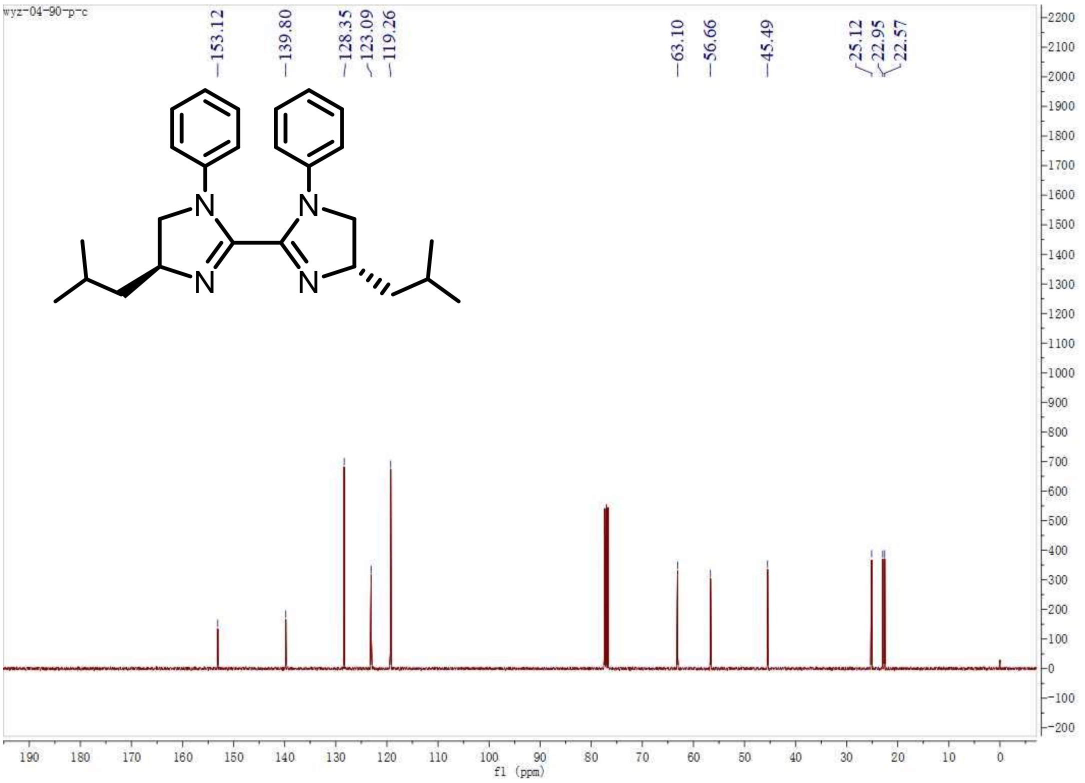

Supplementary Fig. 32. Compound L12 ^{1}H NMR (400 MHz, CDCl$_3$)

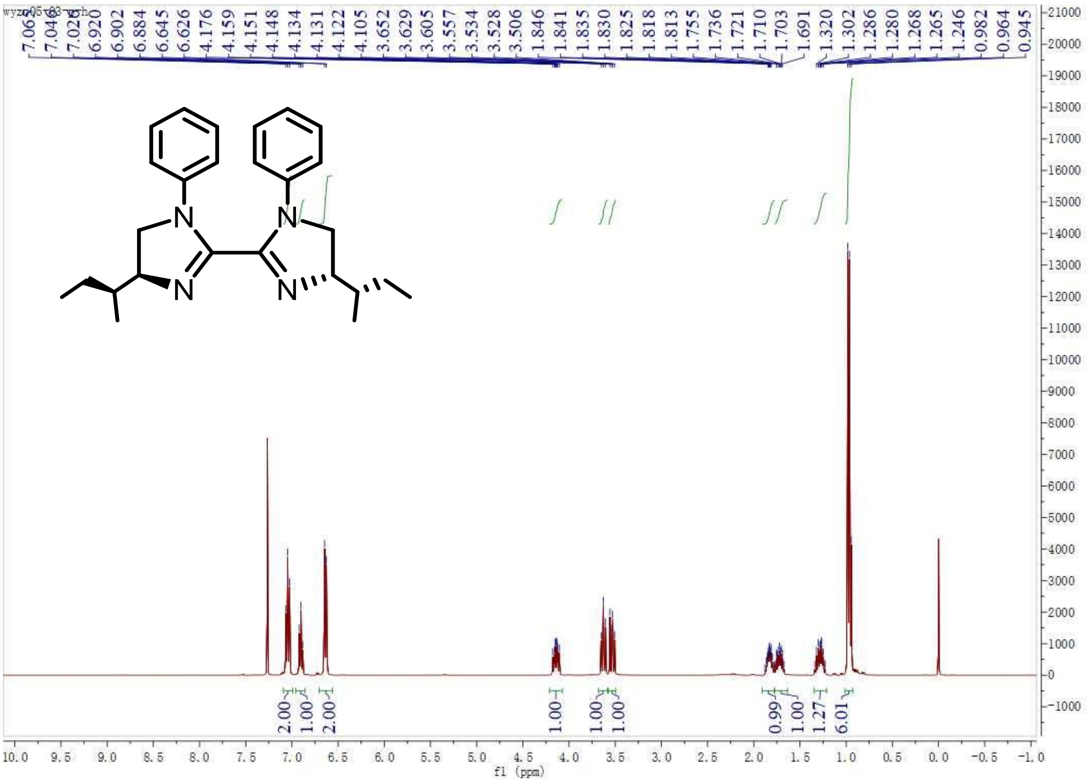

Supplementary Fig. 33. Compound L12 ^{13}C NMR (101 MHz, CDCl$_3$)

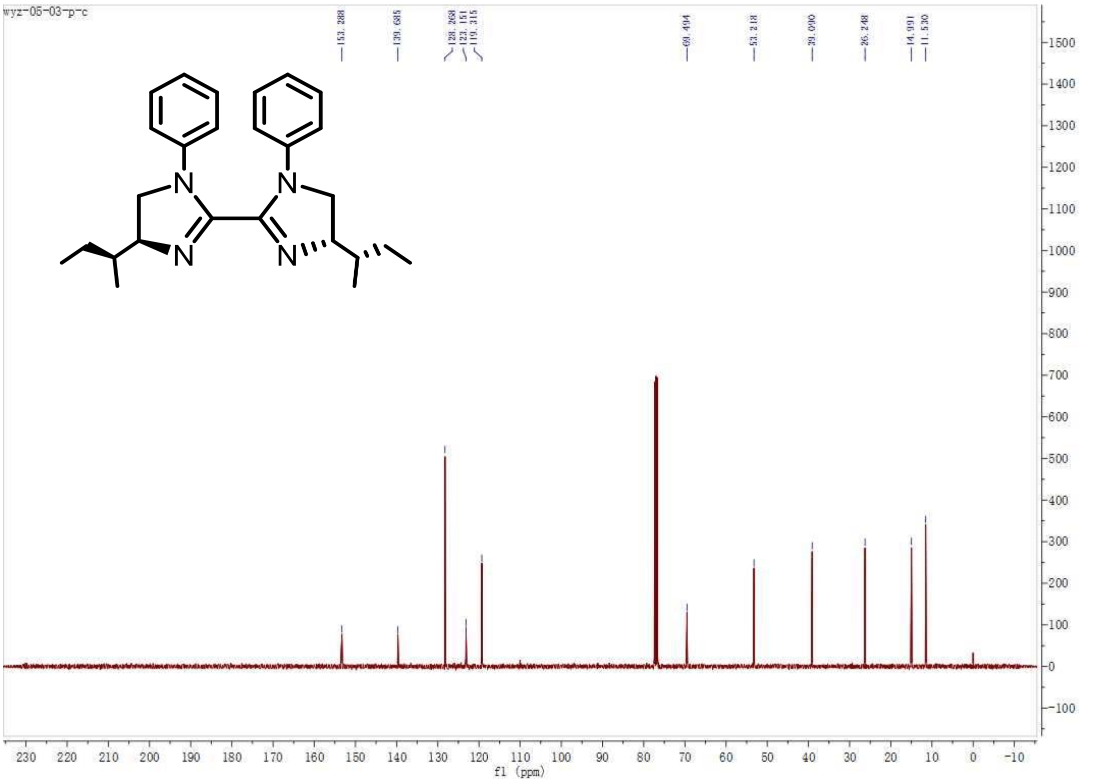

Supplementary Fig. 34. Compound L13 ^{13}C NMR (101 MHz, CDCl$_3$)

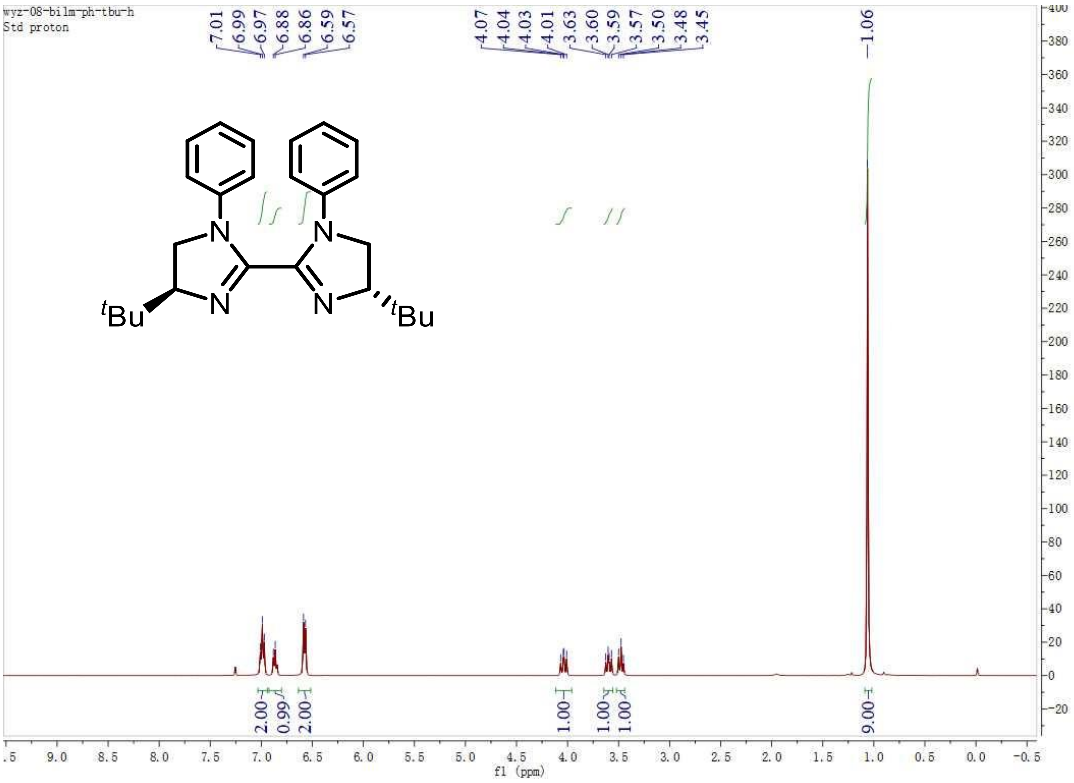

Supplementary Fig. 35. Compound L13 ^{13}C NMR (101 MHz, CDCl$_3$)

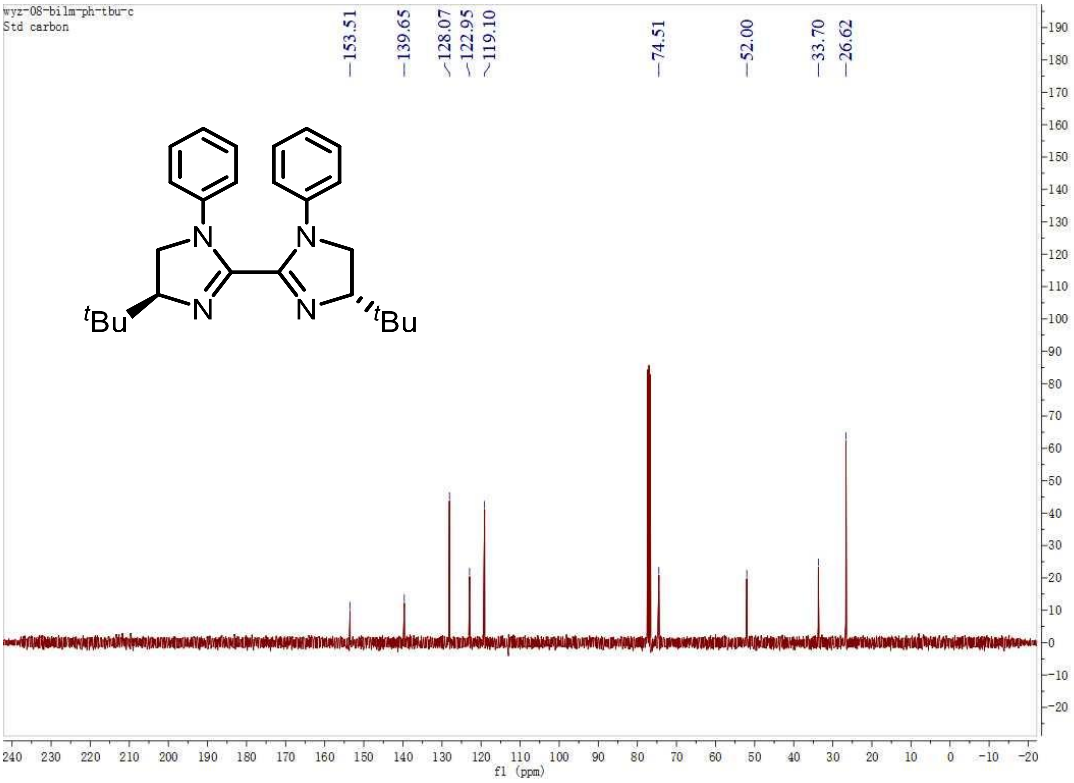

Supplementary Fig. 36. Compound 3a ^{1}H NMR (400 MHz, $CDCl_3$)

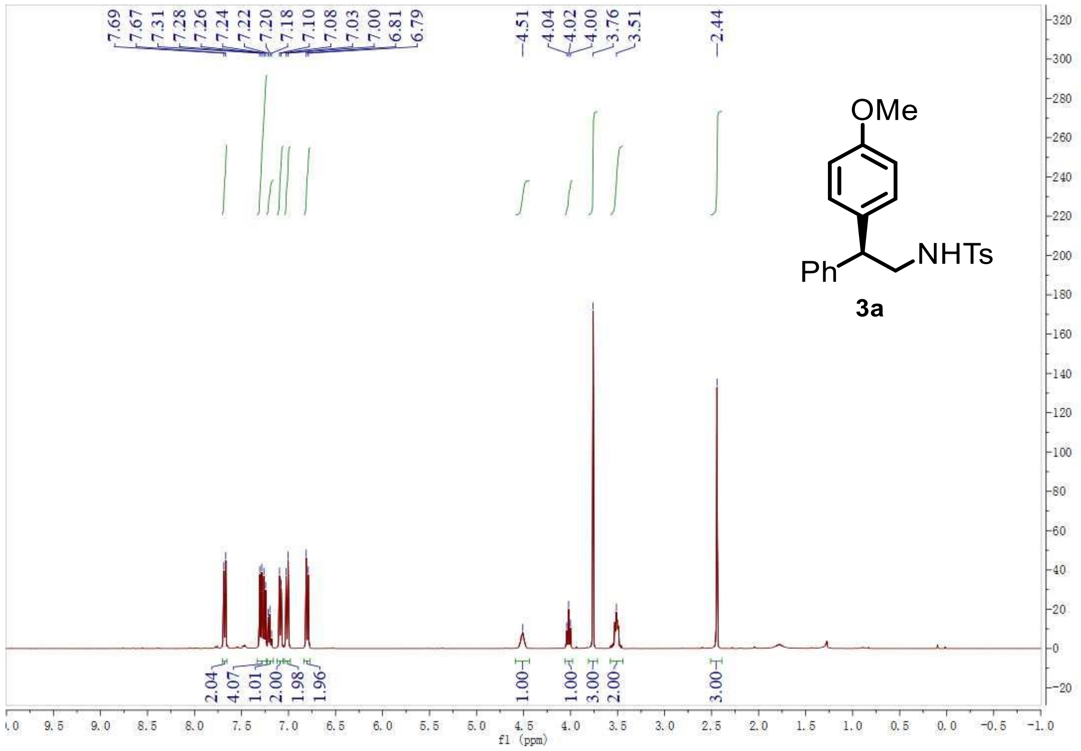

Supplementary Fig. 37. Compound 3a ^{13}C NMR (101 MHz, $CDCl_3$)

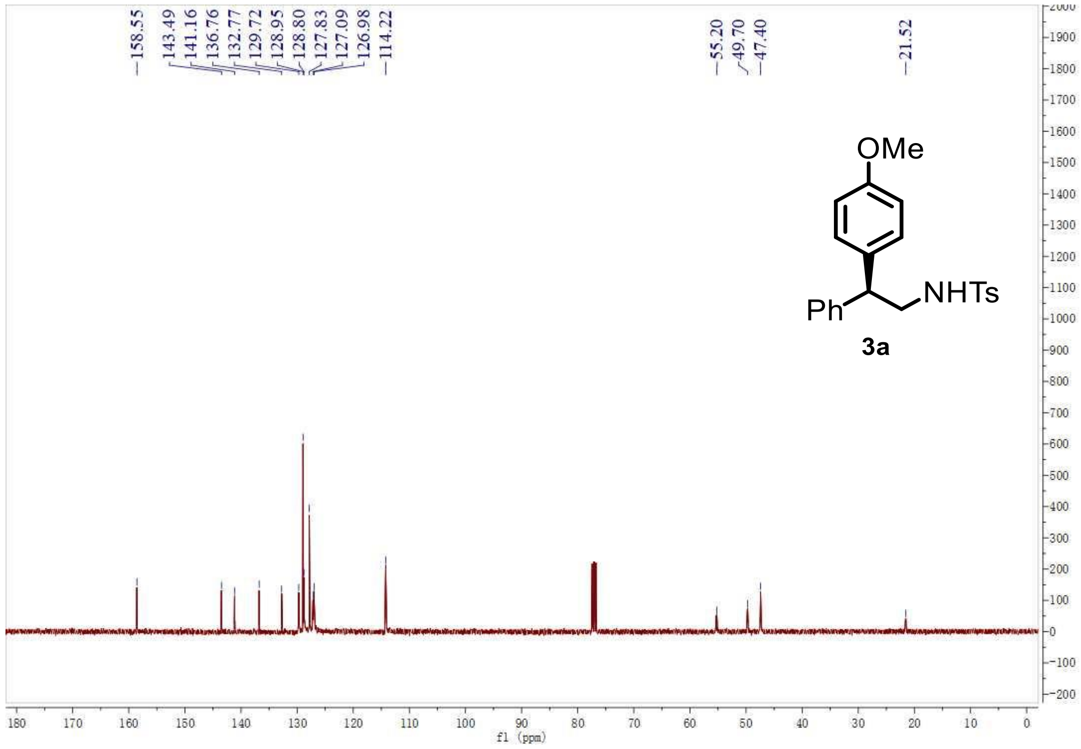

Supplementary Fig. 38. Compound 3b ^{1}H NMR (400 MHz, $CDCl_3$)

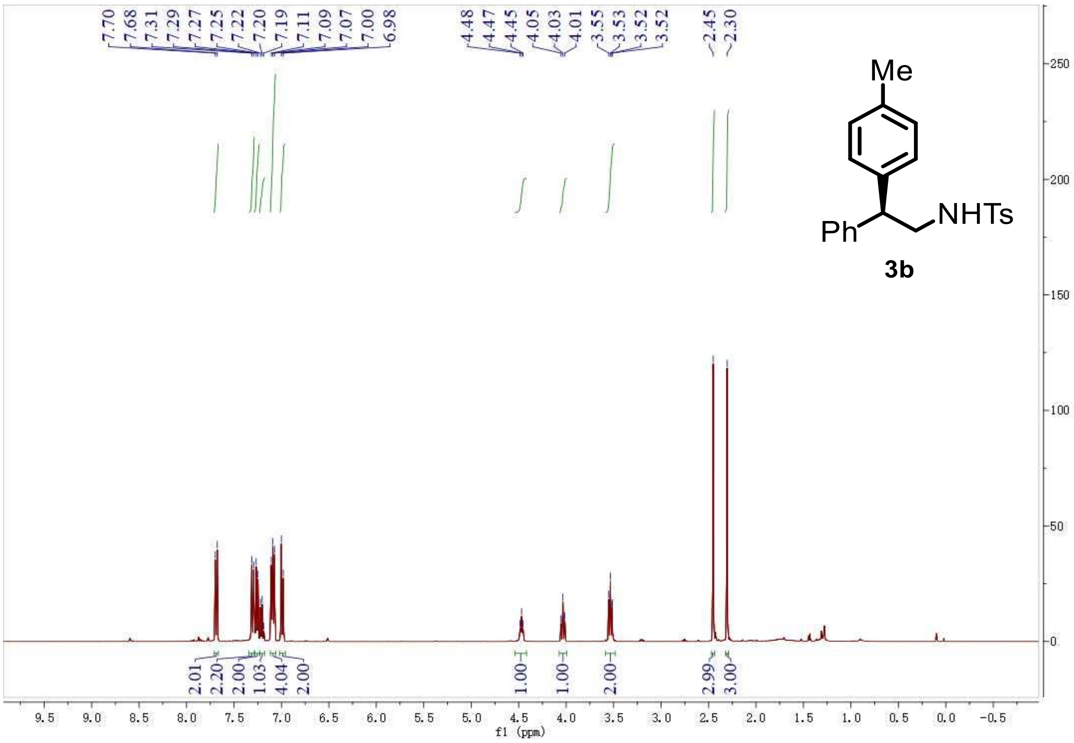

Supplementary Fig. 39. Compound 3b ^{13}C NMR (101 MHz, $CDCl_3$)

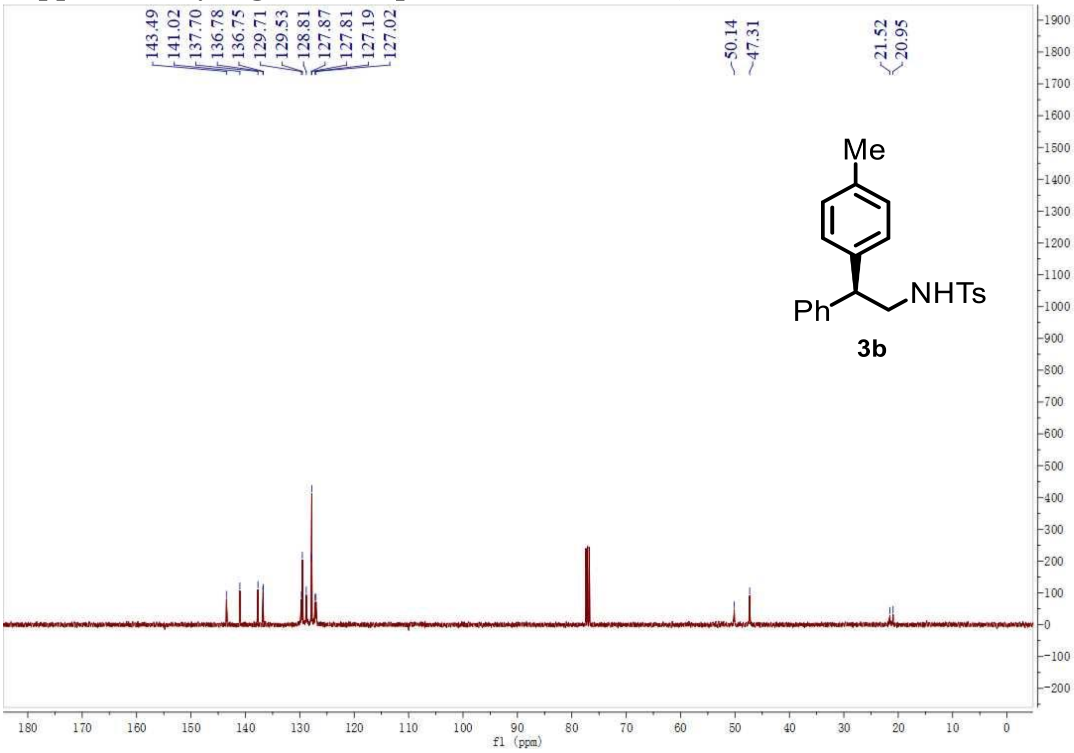

Supplementary Fig. 40. Compound 3c ^{1}H NMR (400 MHz, $CDCl_3$)

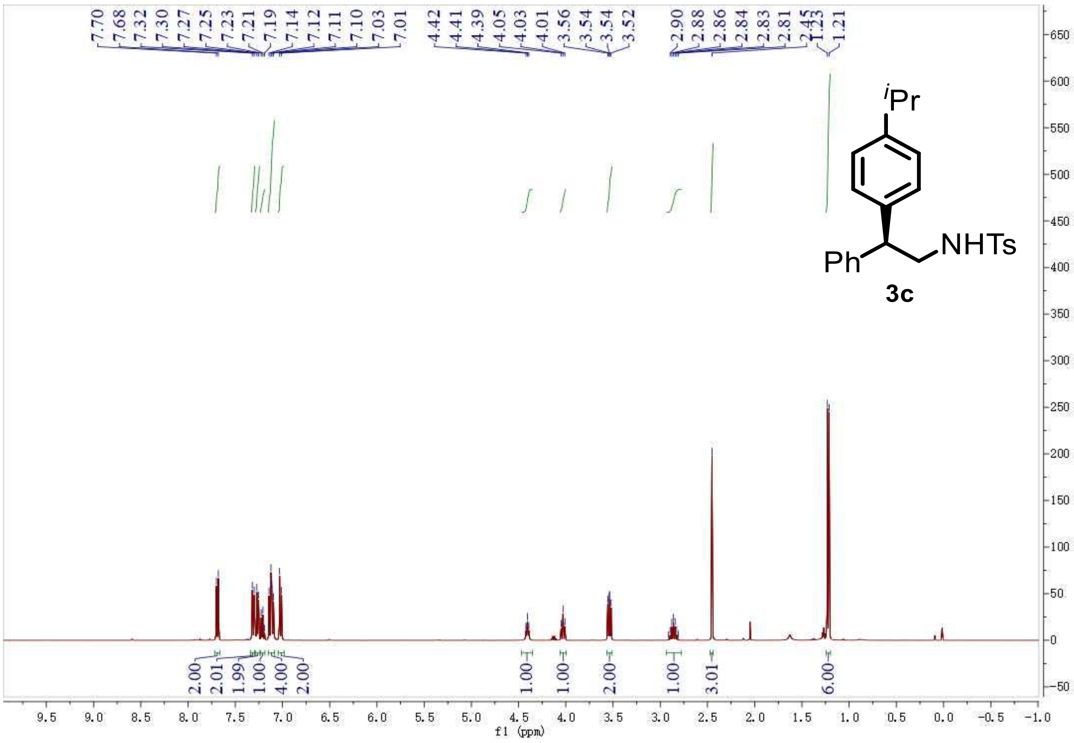

Supplementary Fig. 41. Compound 3c ^{13}C NMR (101 MHz, $CDCl_3$)

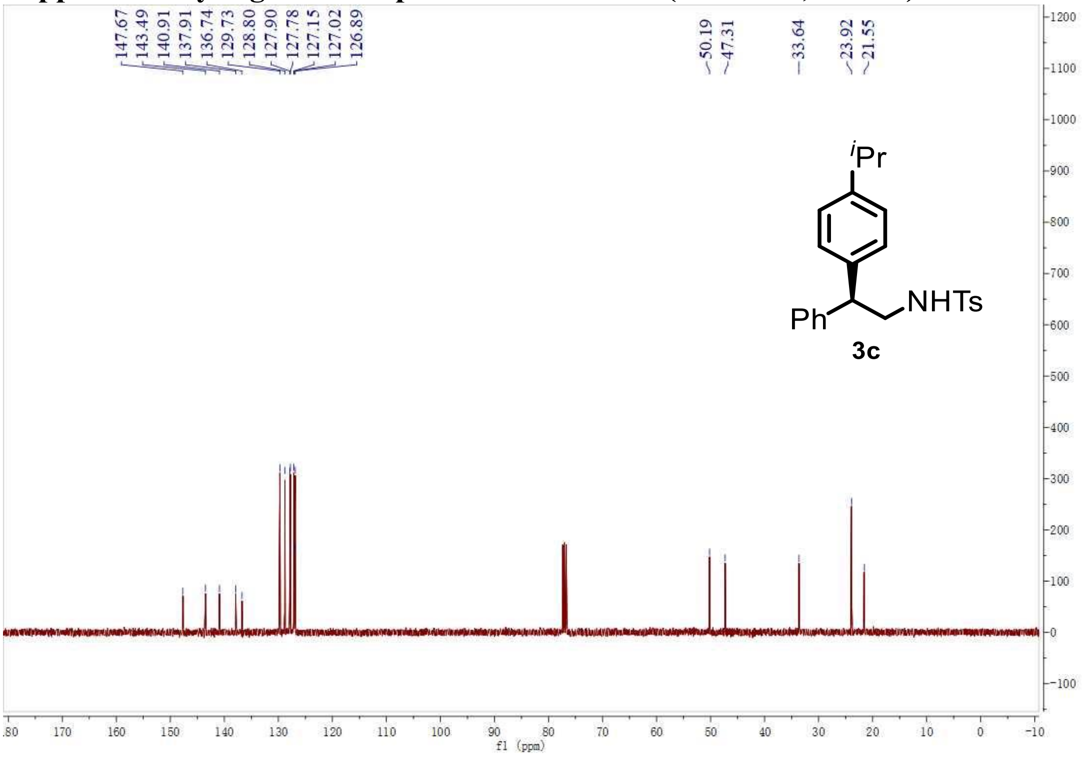

Supplementary Fig. 42. Compound 3d ^{1}H NMR (400 MHz, $CDCl_3$)

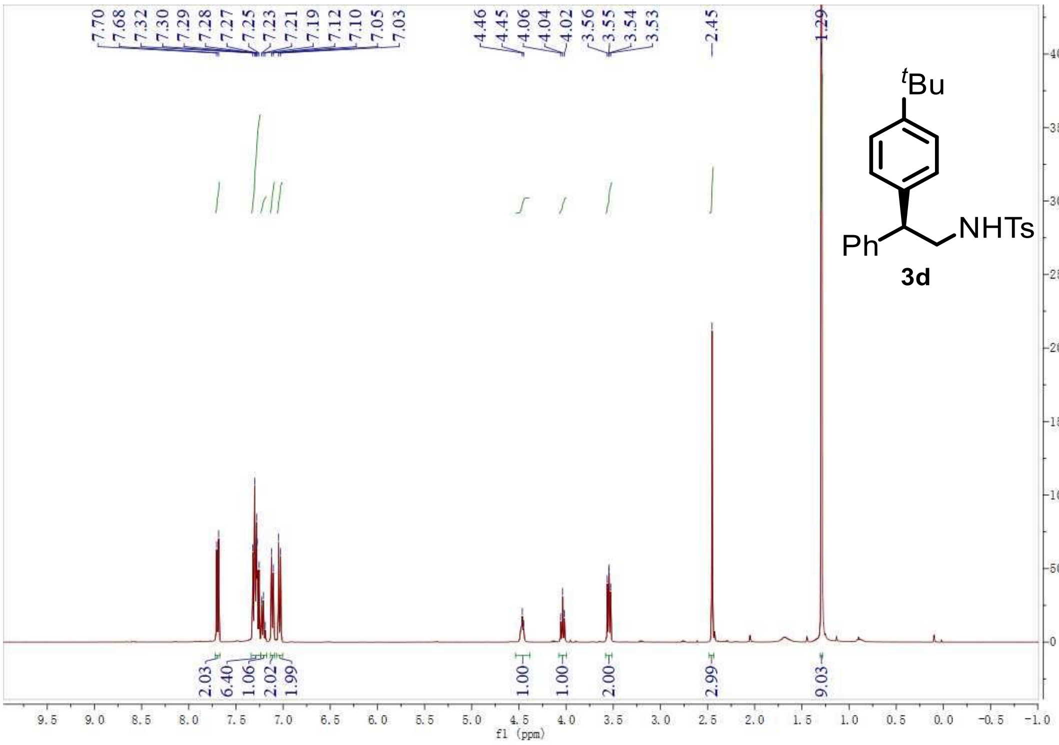

Supplementary Fig. 43. Compound 3d ^{13}C NMR (101 MHz, $CDCl_3$)

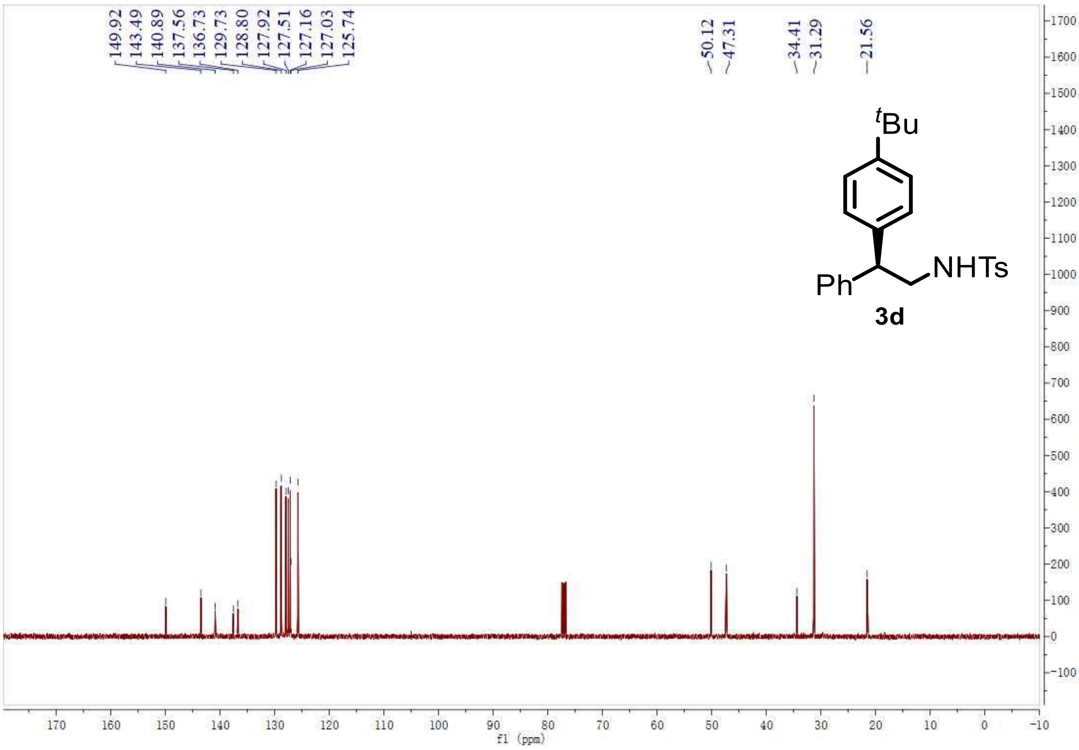

Supplementary Fig. 44. Compound 3e ^{1}H NMR (400 MHz, $CDCl_3$)

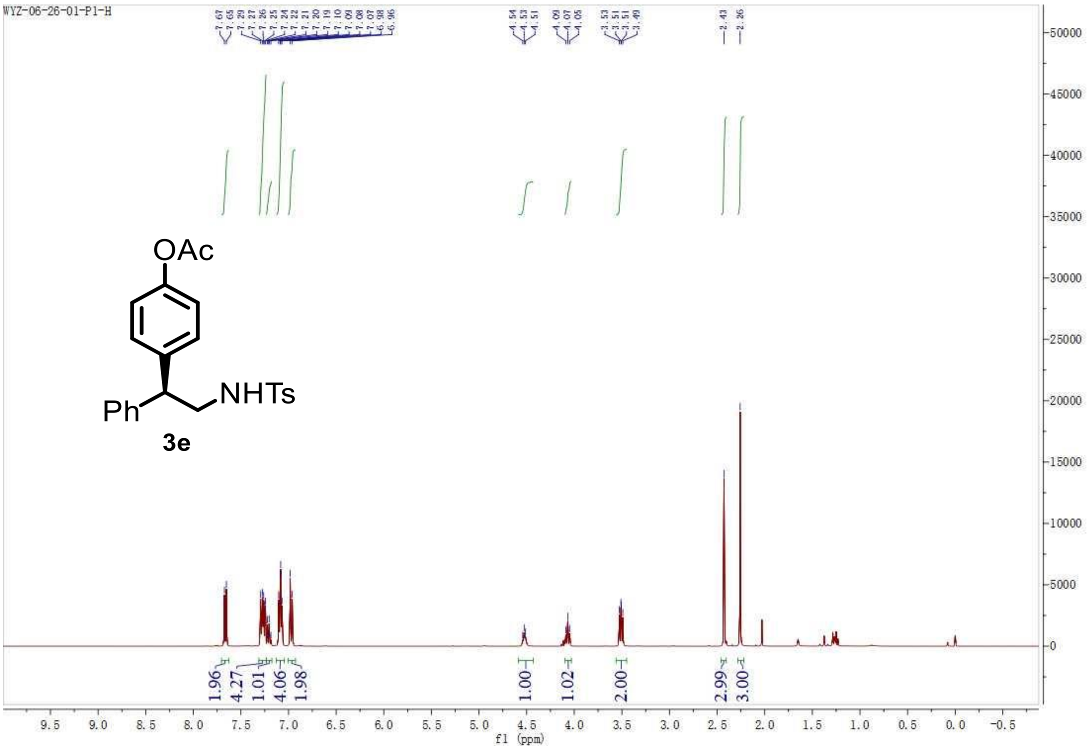

Supplementary Fig. 45. Compound 3e ^{13}C NMR (101 MHz, $CDCl_3$)

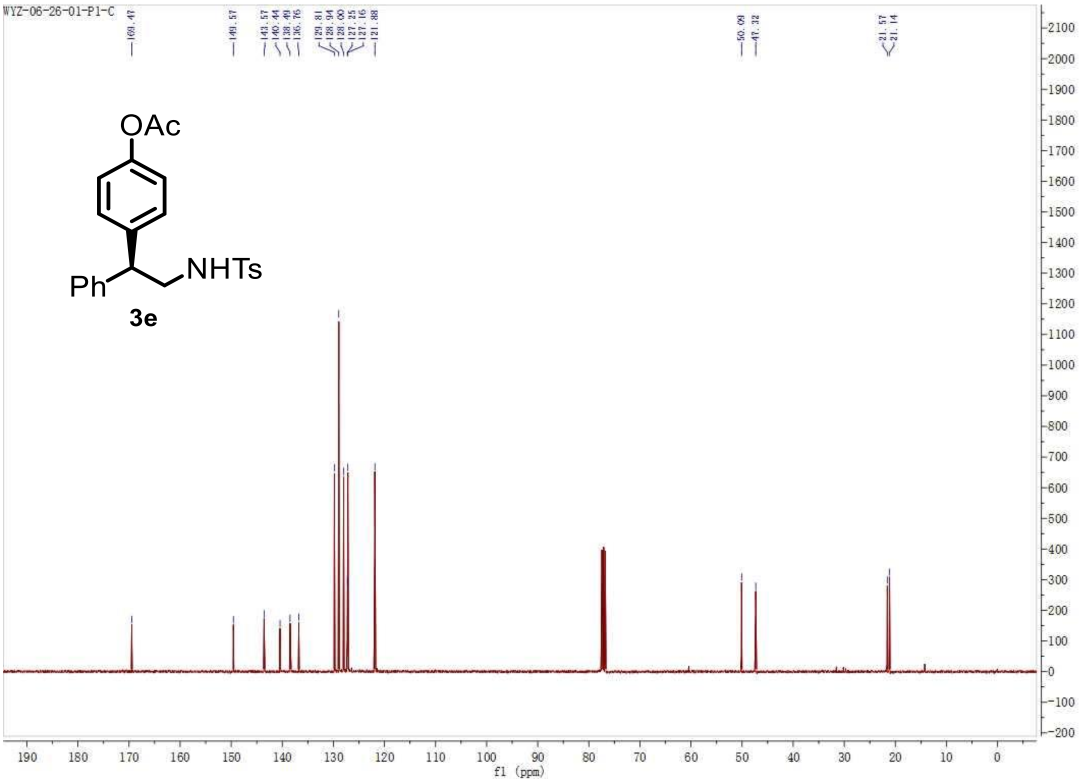

Supplementary Fig. 46. Compound 3f ^{1}H NMR (400 MHz, $CDCl_3$)

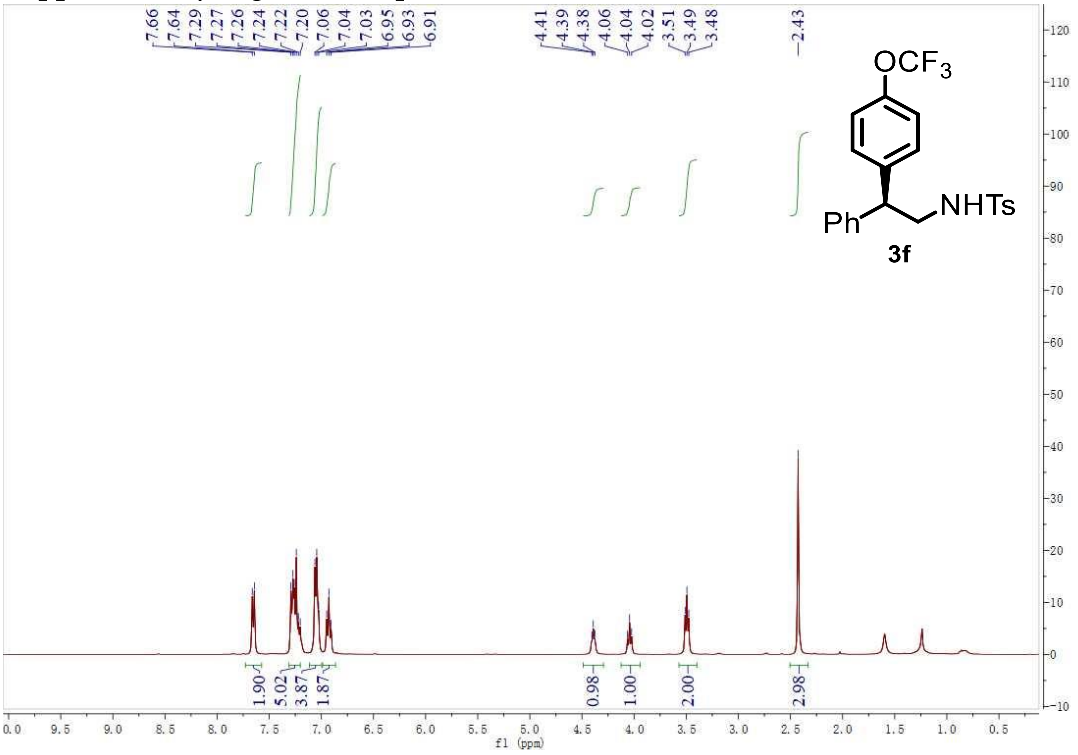

Supplementary Fig. 47. Compound 3f ^{13}C NMR (101 MHz, $CDCl_3$)

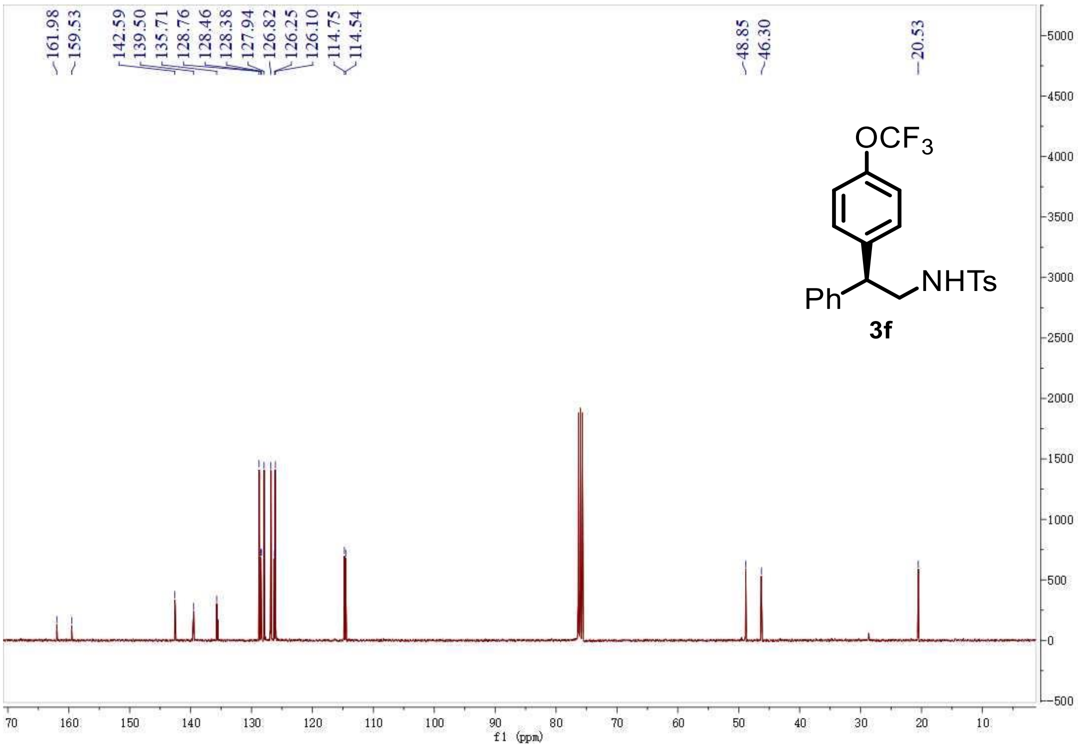

Supplementary Fig. 48. Compound 3f ^{19}F NMR (376 MHz, $CDCl_3$)

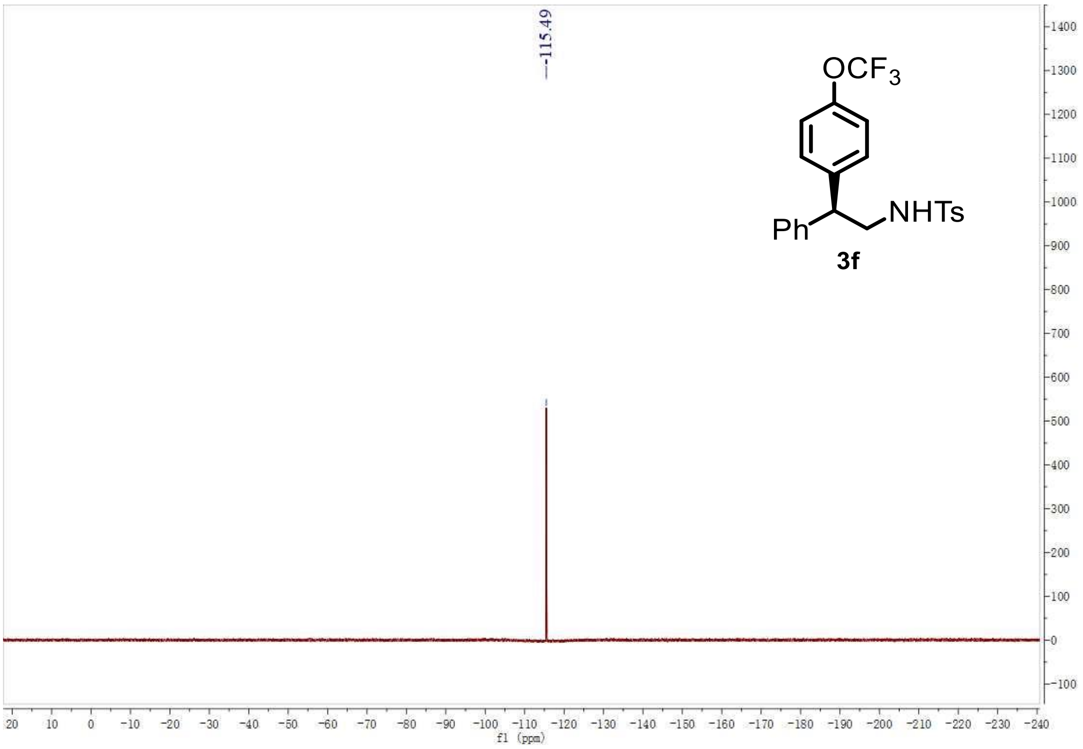

Supplementary Fig. 49. Compound 3g ^{1}H NMR (400 MHz, $CDCl_3$)

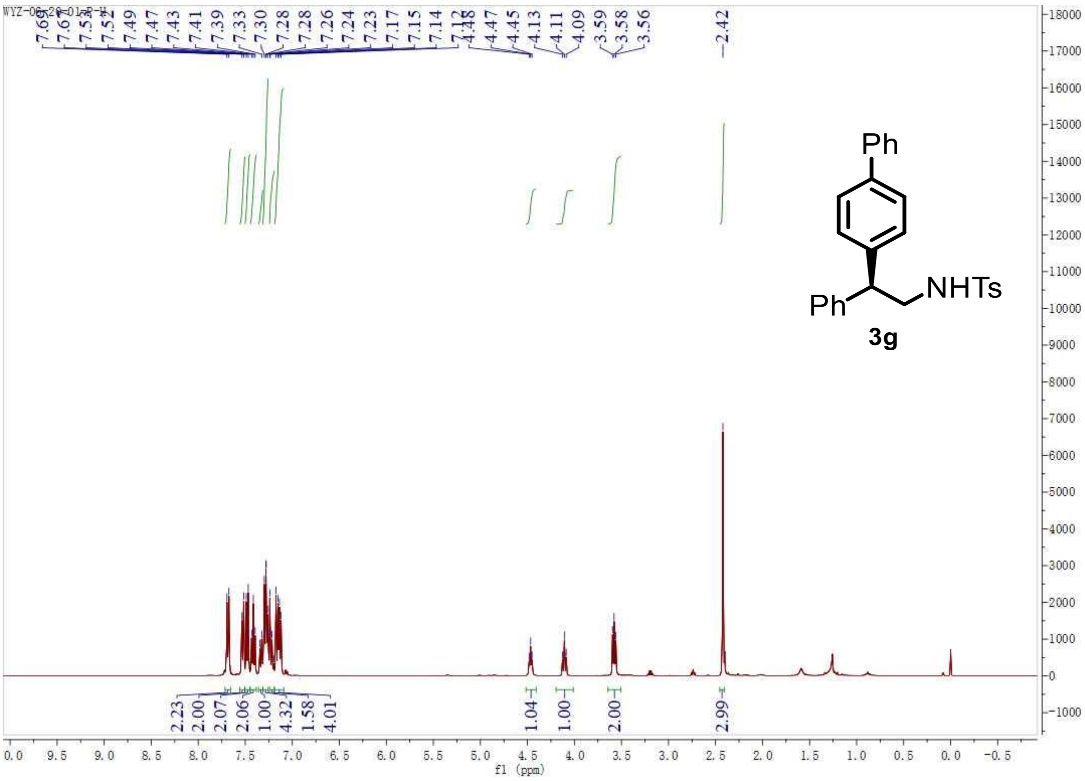

Supplementary Fig. 50. Compound 3g ^{13}C NMR (101 MHz, $CDCl_3$)

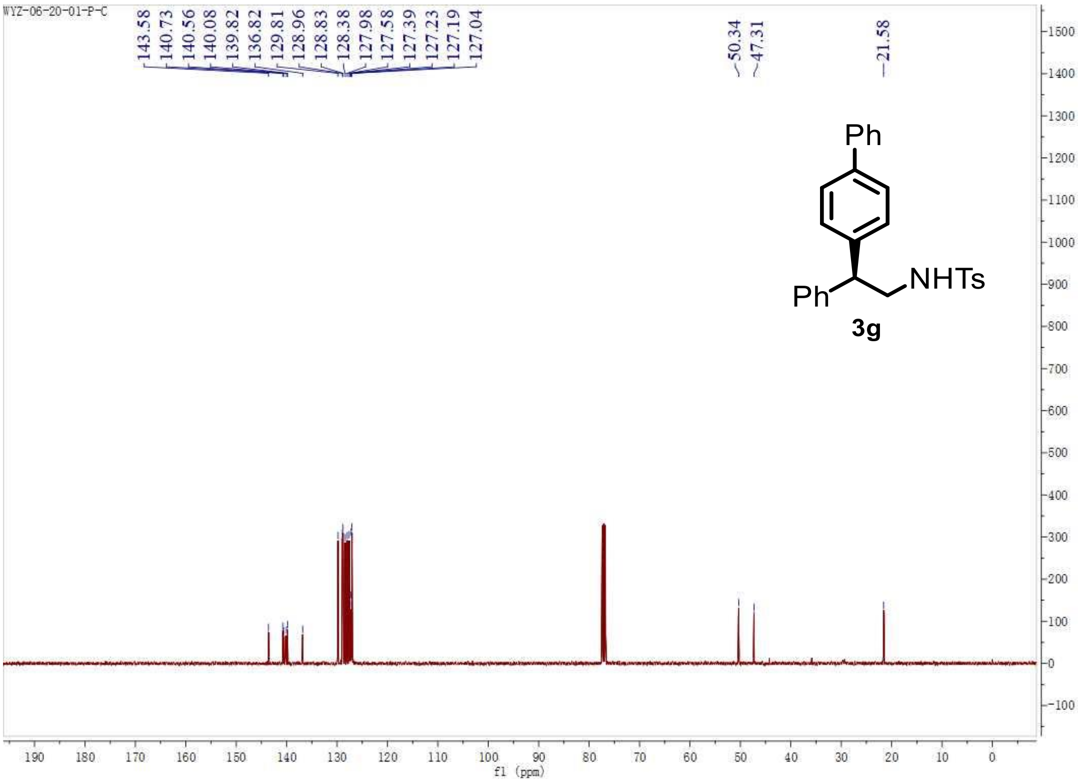

Supplementary Fig. 51. Compound 3h ^{1}H NMR (400 MHz, $CDCl_3$)

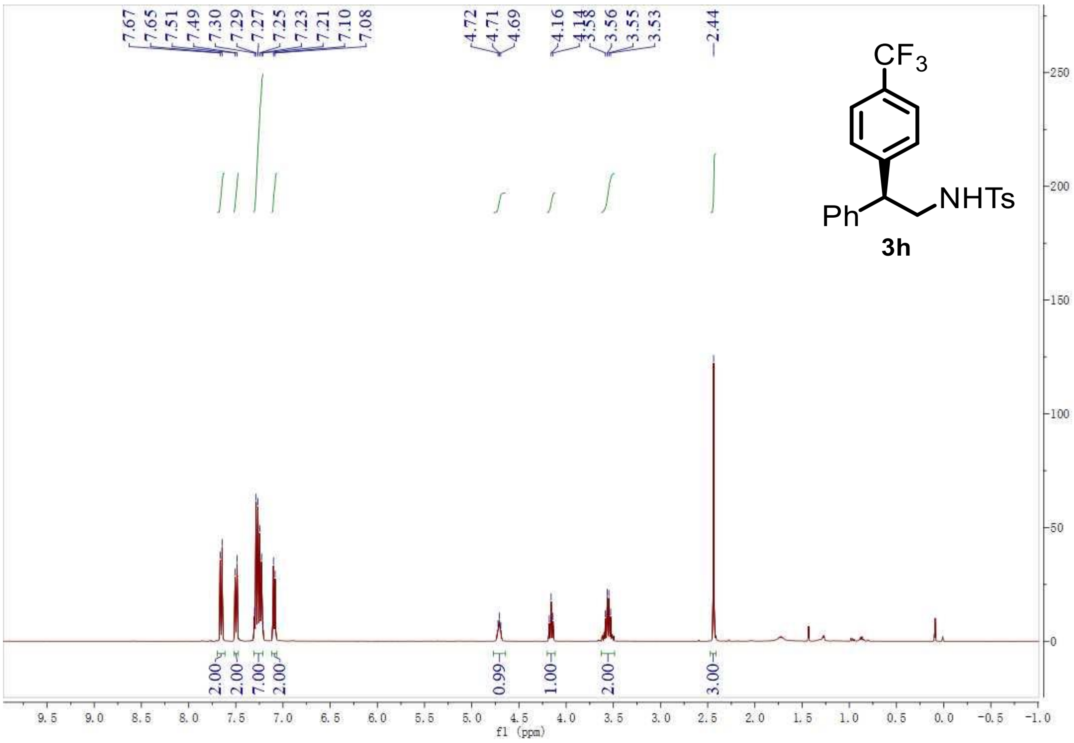

Supplementary Fig. 52. Compound 3h ^{13}C NMR (101 MHz, CDCl$_3$)

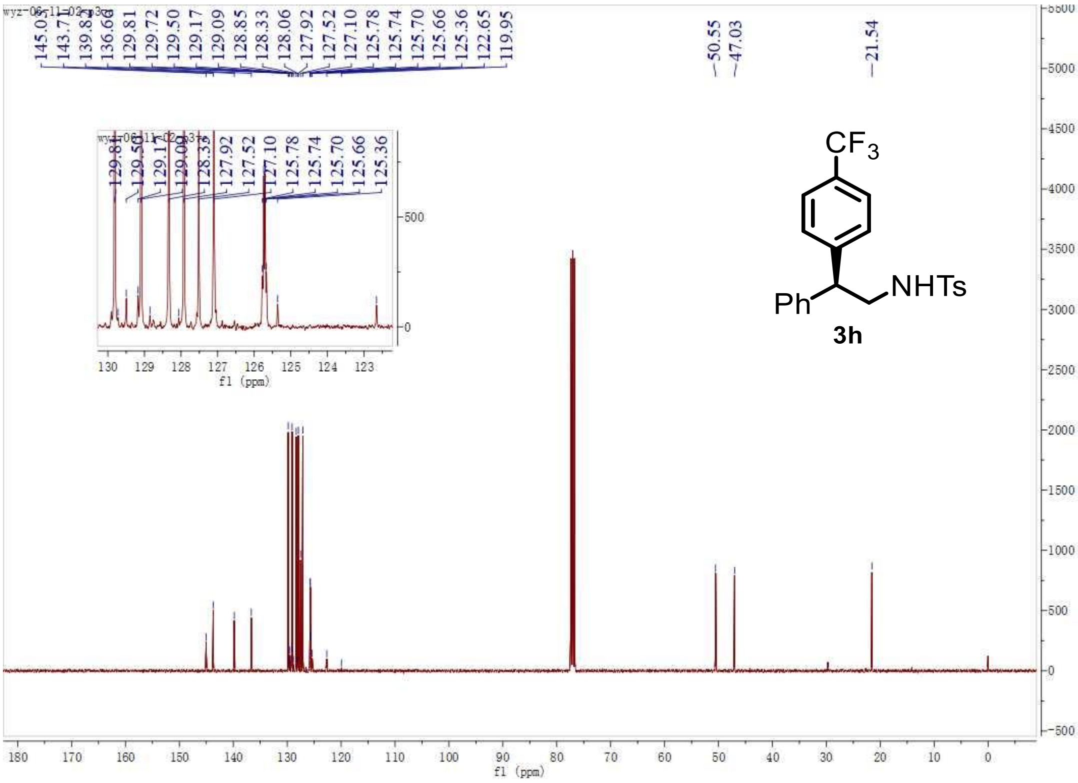

Supplementary Fig. 53. Compound 3h ^{19}F NMR (376 MHz, CDCl$_3$)

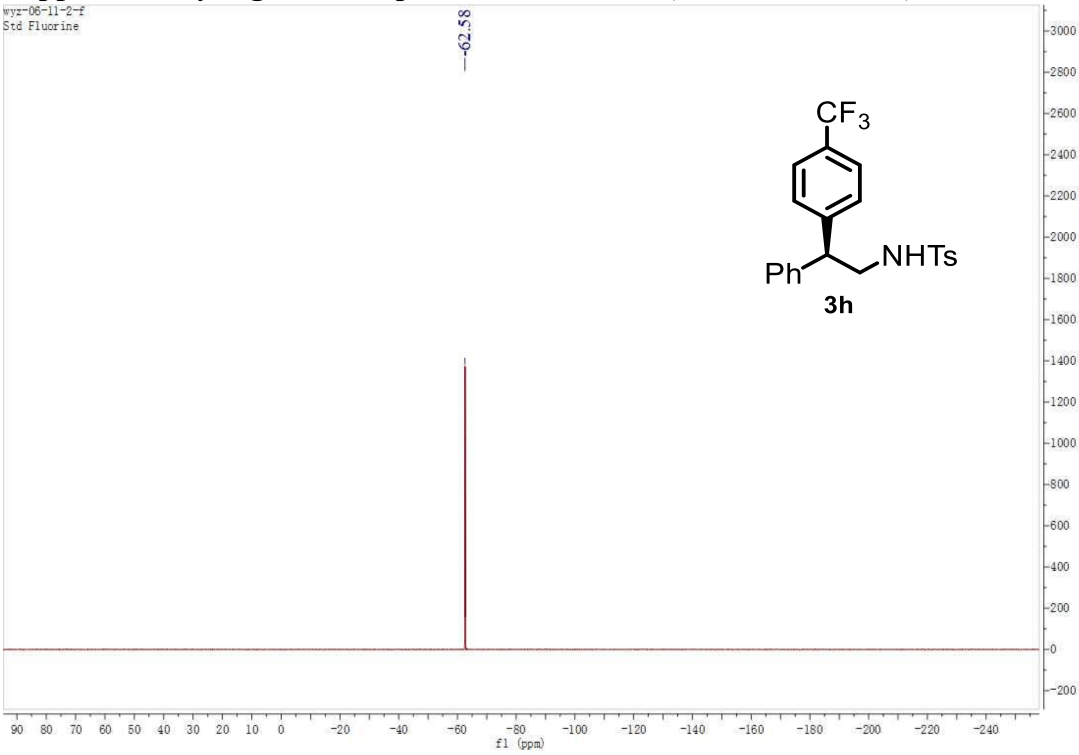

Supplementary Fig. 54. Compound 3i ^{1}H NMR (400 MHz, $CDCl_3$)

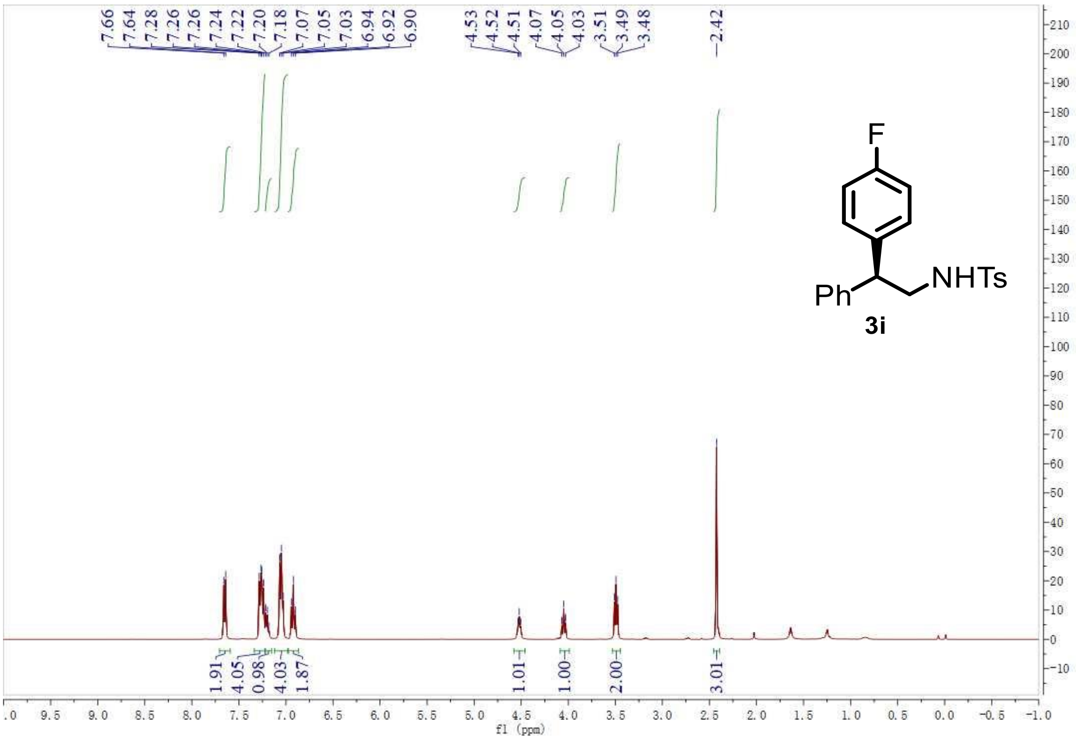

Supplementary Fig. 55. Compound 3i ^{13}C NMR (101 MHz, $CDCl_3$)

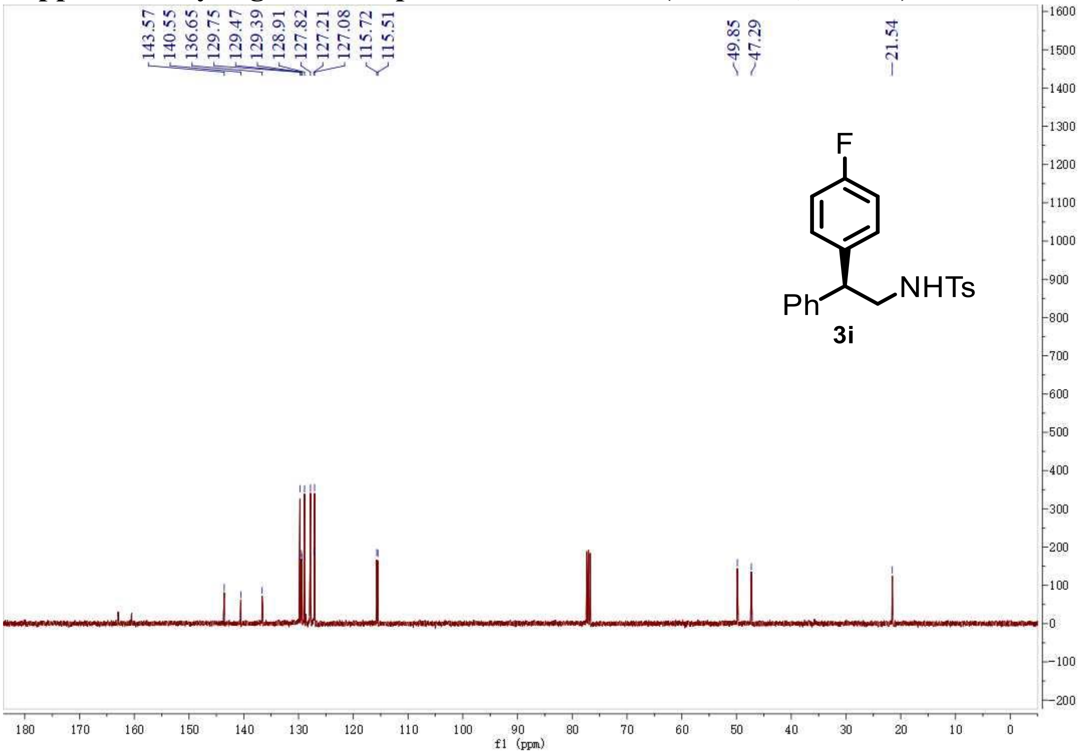

Supplementary Fig. 56. Compound 3i ^{19}F NMR (376 MHz, $CDCl_3$)

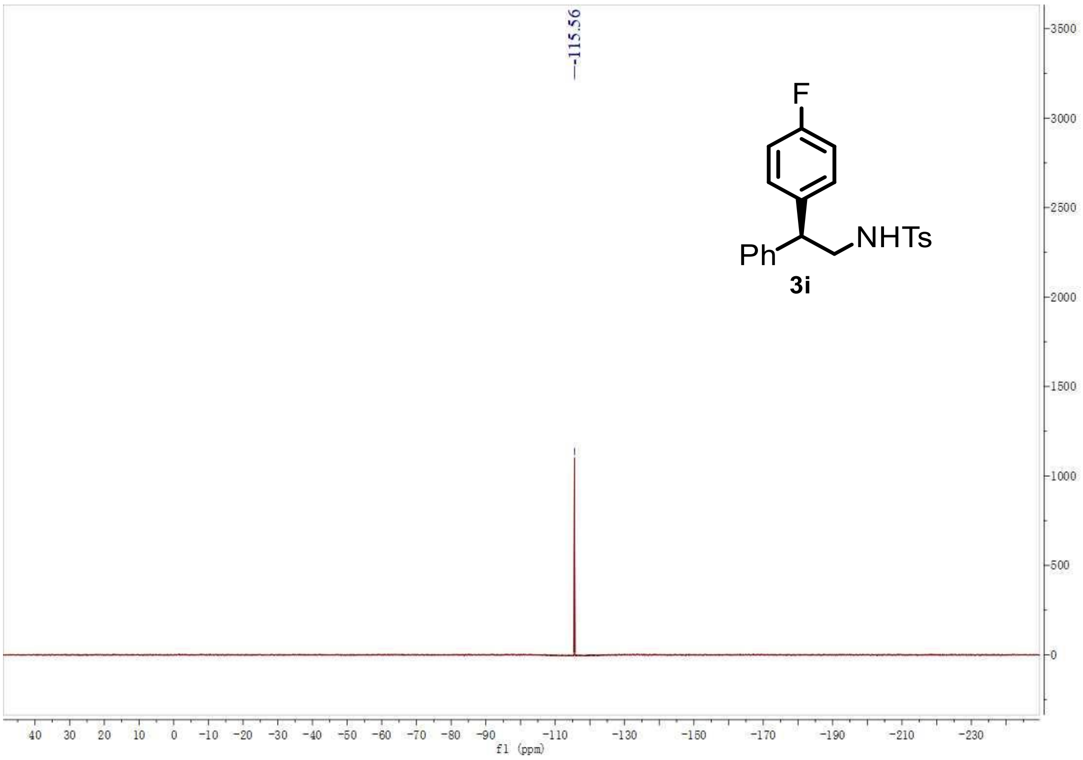

Supplementary Fig. 57. Compound 3j ^{1}H NMR (400 MHz, $CDCl_3$)

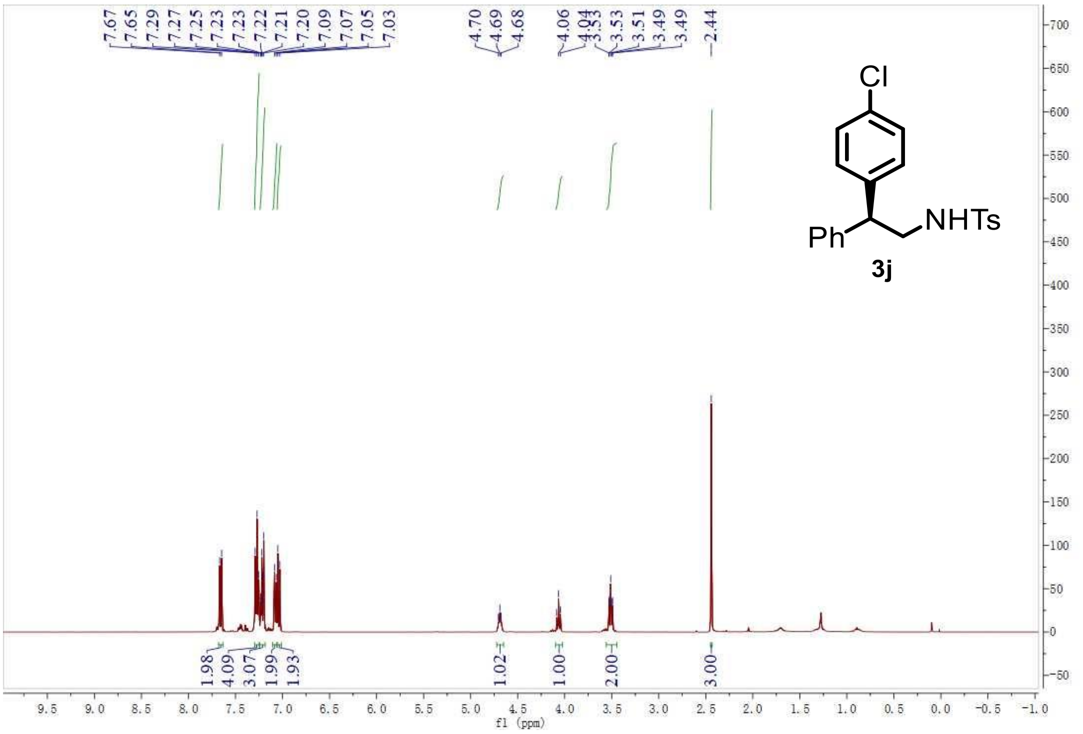

Supplementary Fig. 58. Compound 3j ^{13}C NMR (101 MHz, $CDCl_3$)

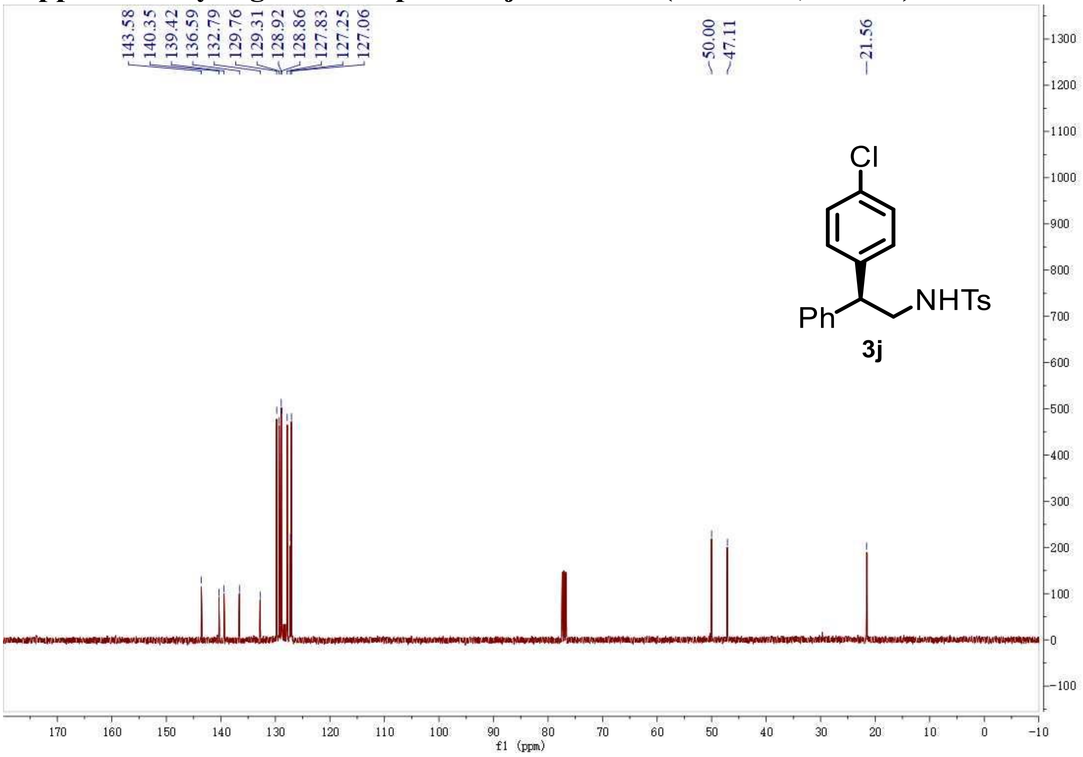

Supplementary Fig. 59. Compound 3k ^{1}H NMR (400 MHz, $CDCl_3$)

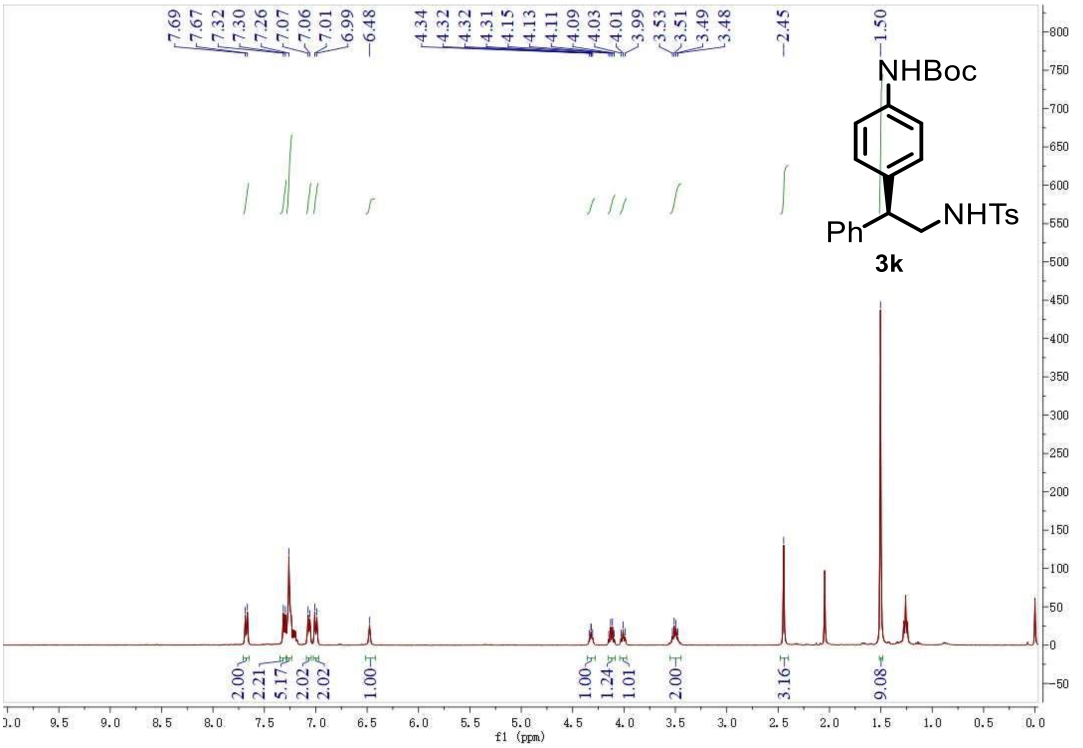

Supplementary Fig. 60. Compound 3k ^{13}C NMR (101 MHz, $CDCl_3$)

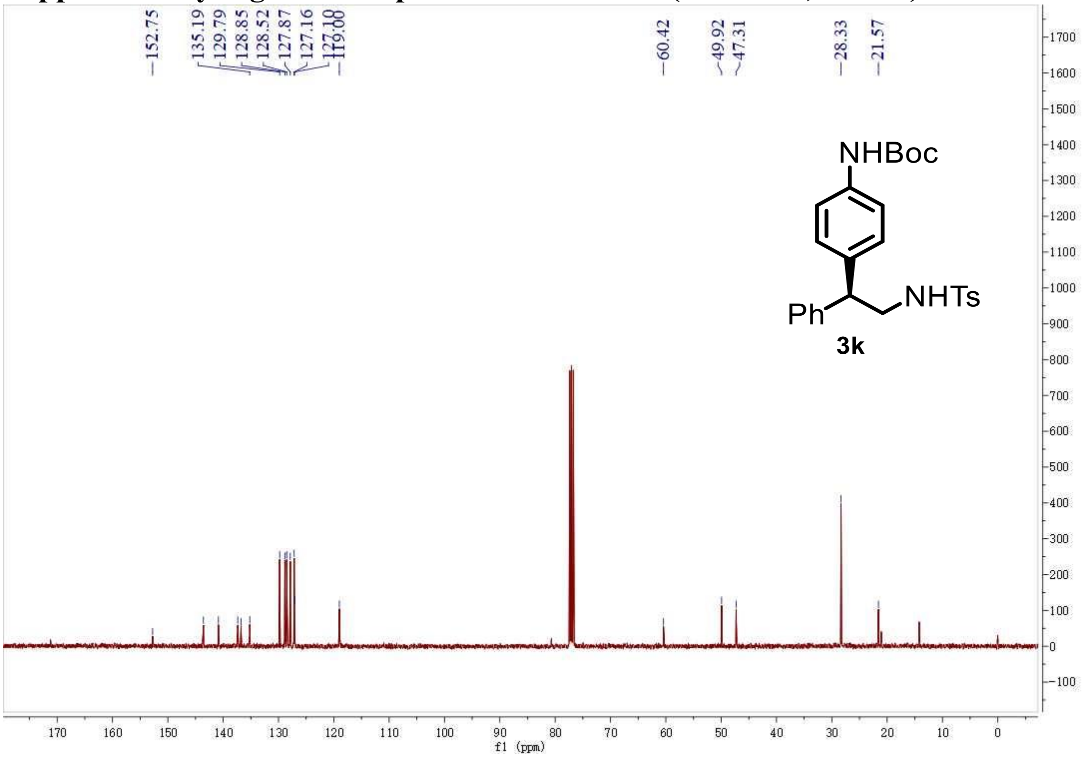

Supplementary Fig. 61. Compound 3l ^{1}H NMR (400 MHz, $CDCl_3$)

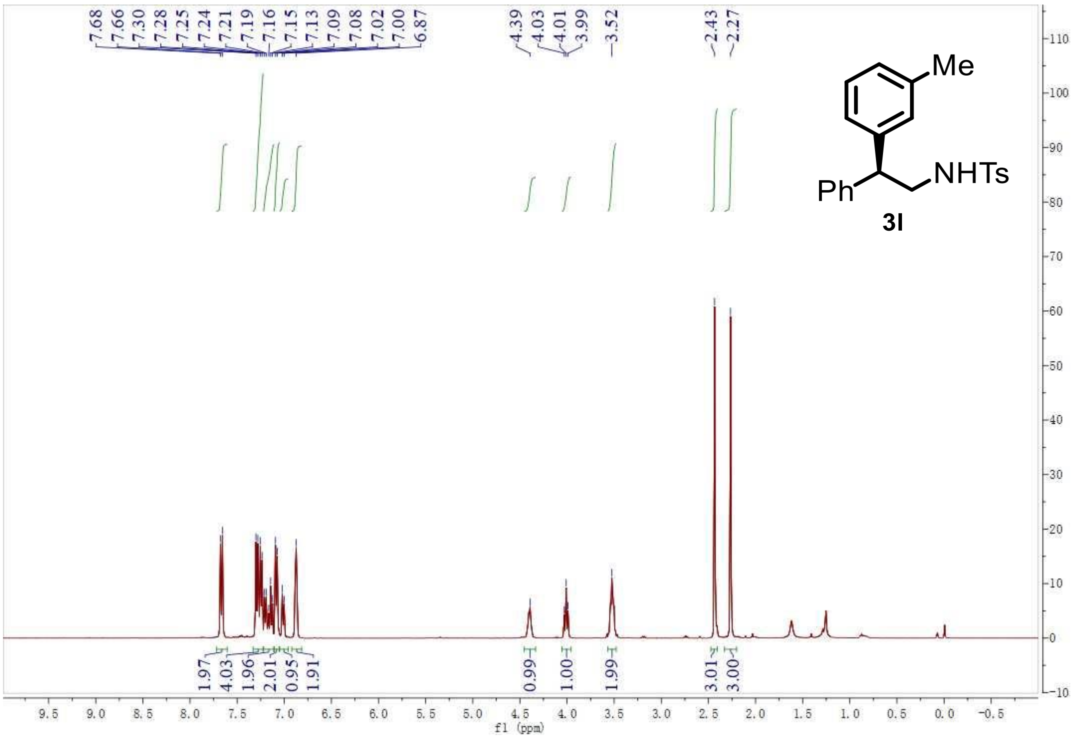

Supplementary Fig. 62. Compound 3l ^{13}C NMR (101 MHz, $CDCl_3$)

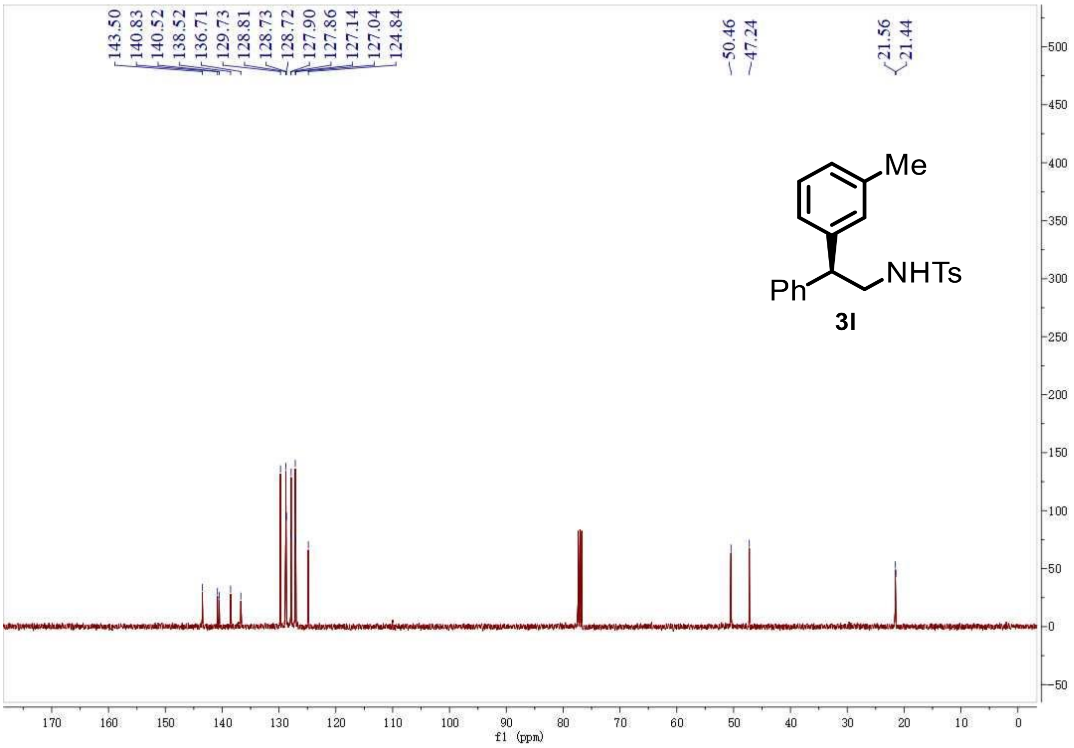

Supplementary Fig. 63. Compound 3m ^{1}H NMR (400 MHz, $CDCl_3$)

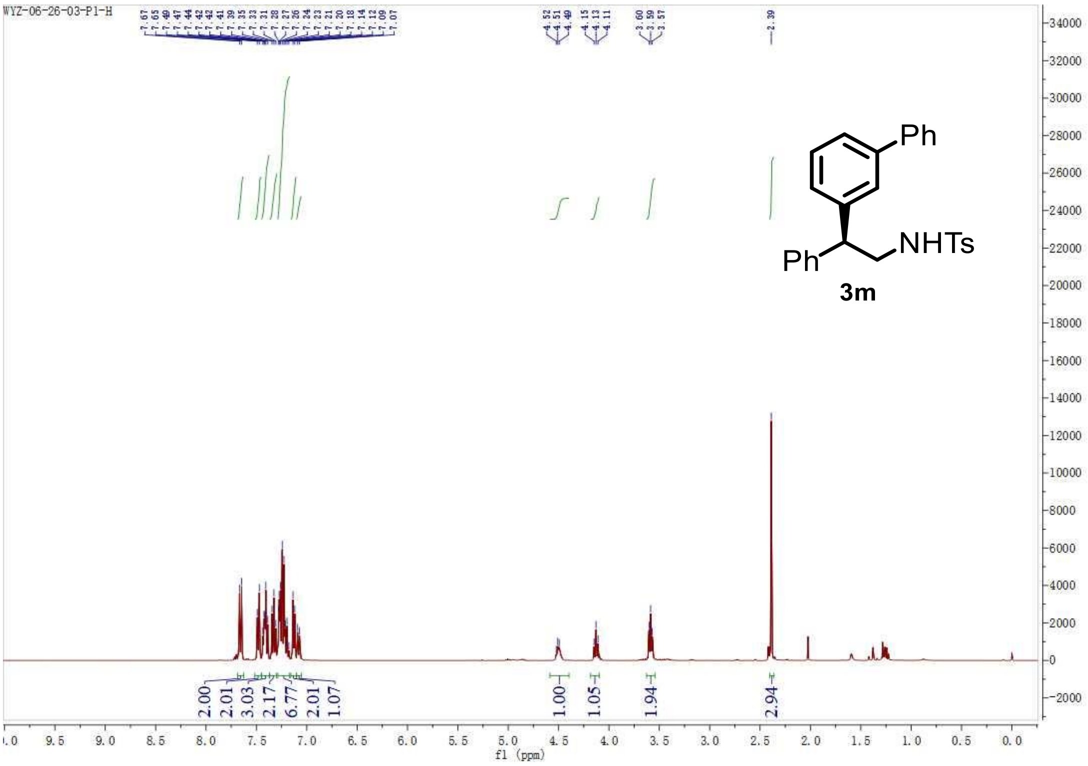

Supplementary Fig. 64. Compound 3m ^{13}C NMR (101 MHz, $CDCl_3$)

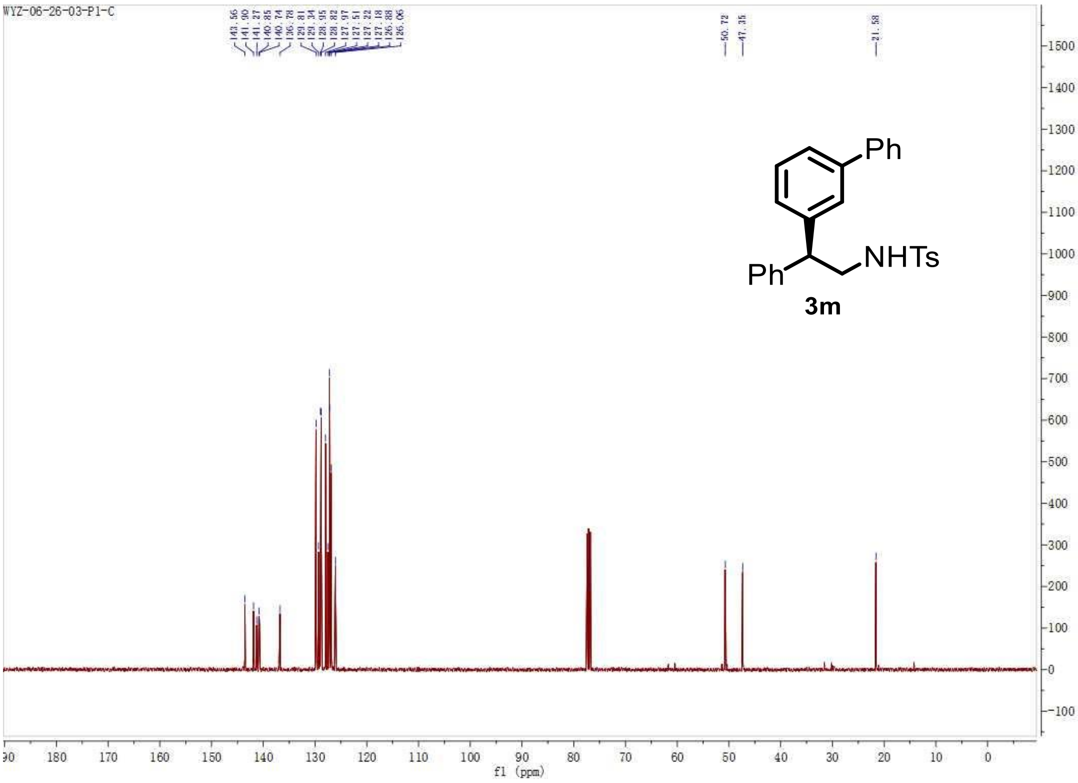

Supplementary Fig. 65. Compound 3n ^{1}H NMR (400 MHz, $CDCl_3$)

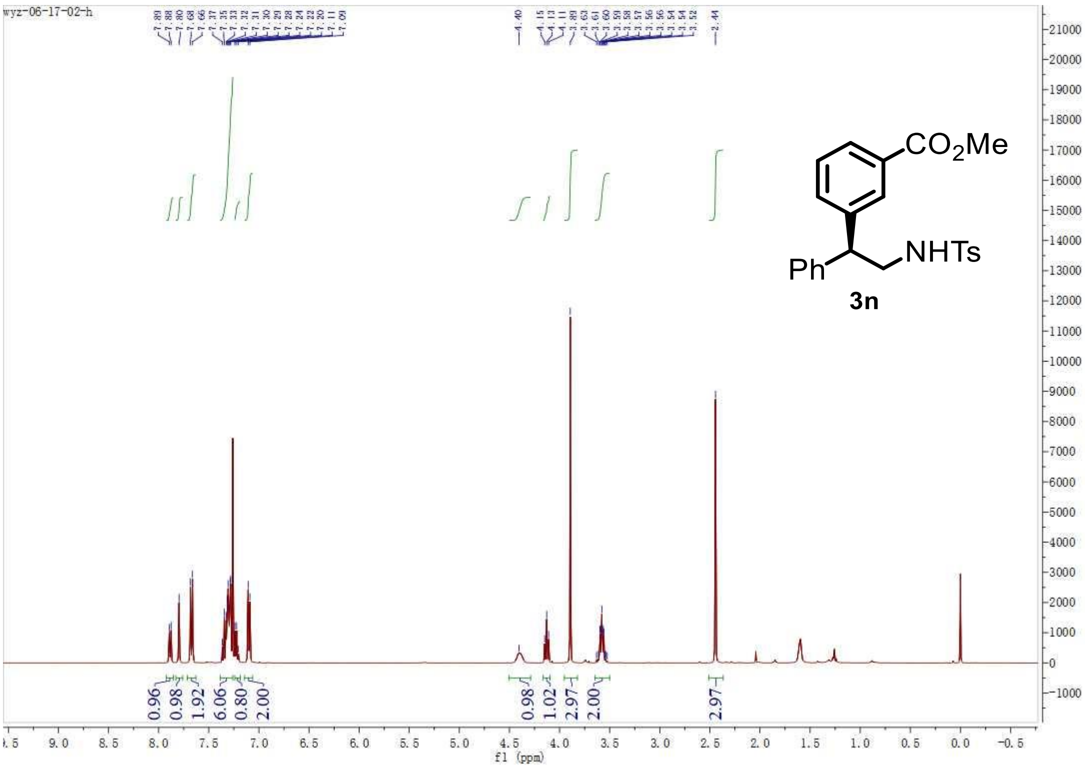

Supplementary Fig. 66. Compound 3n ^{13}C NMR (101 MHz, $CDCl_3$)

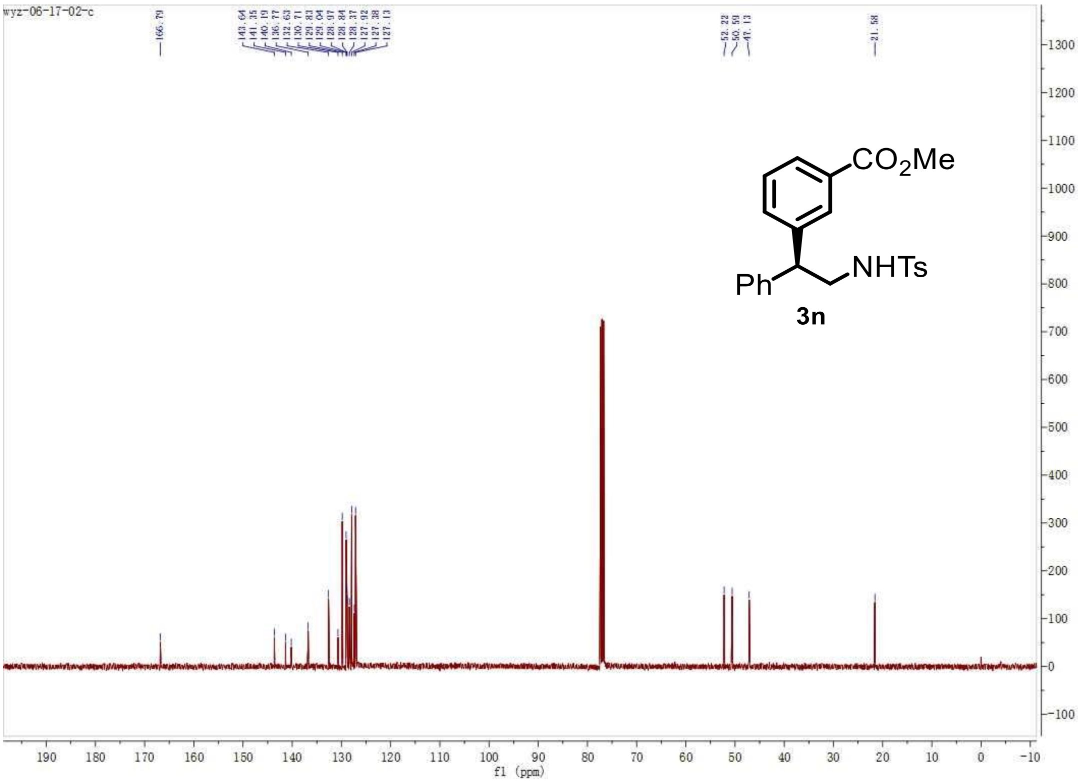

Supplementary Fig. 67. Compound 3o ^{1}H NMR (400 MHz, $CDCl_3$)

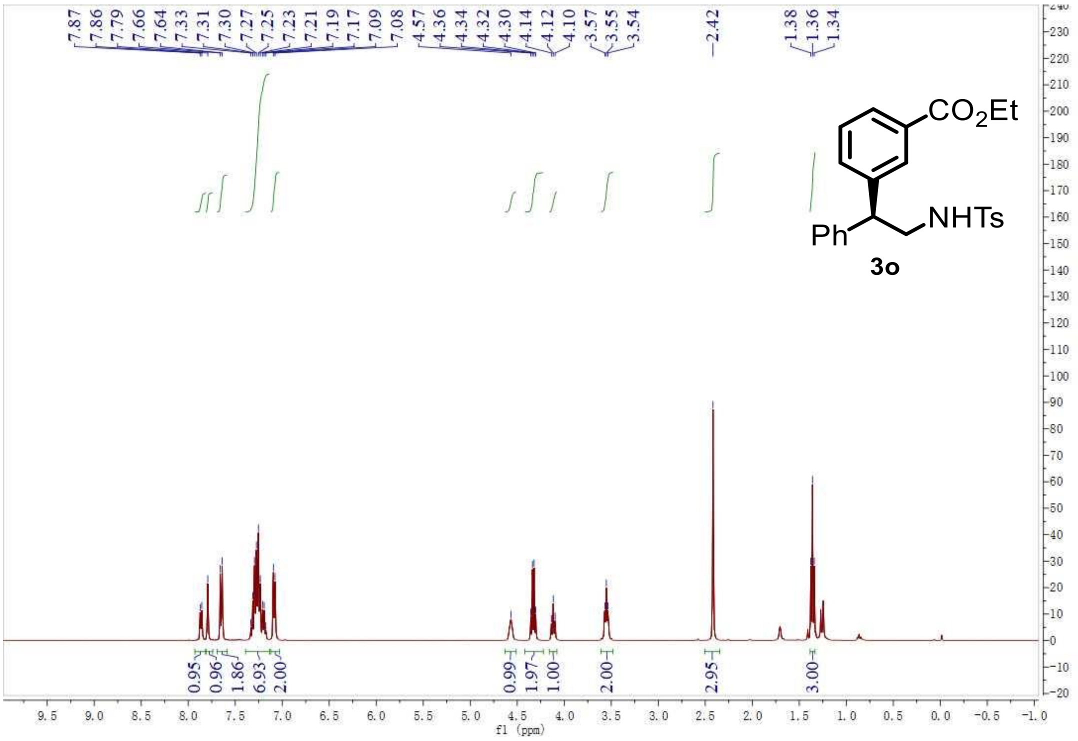

Supplementary Fig. 68. Compound 3o ^{13}C NMR (101 MHz, $CDCl_3$)

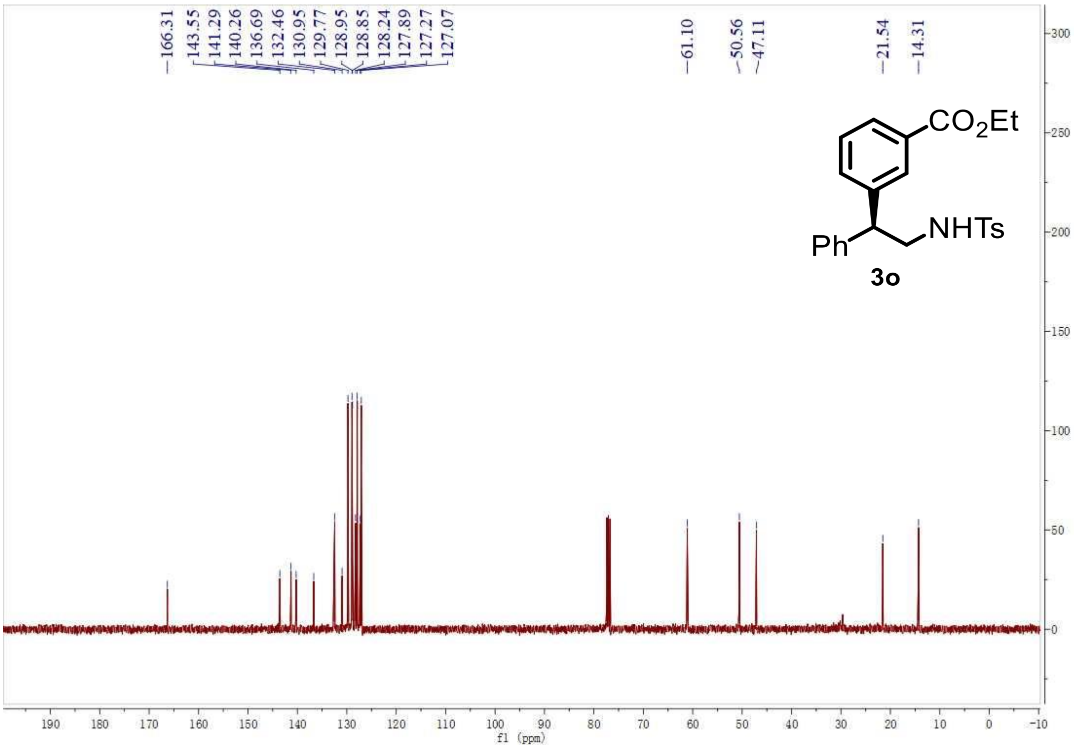

Supplementary Fig. 69. Compound 3p ^{1}H NMR (400 MHz, $CDCl_3$)

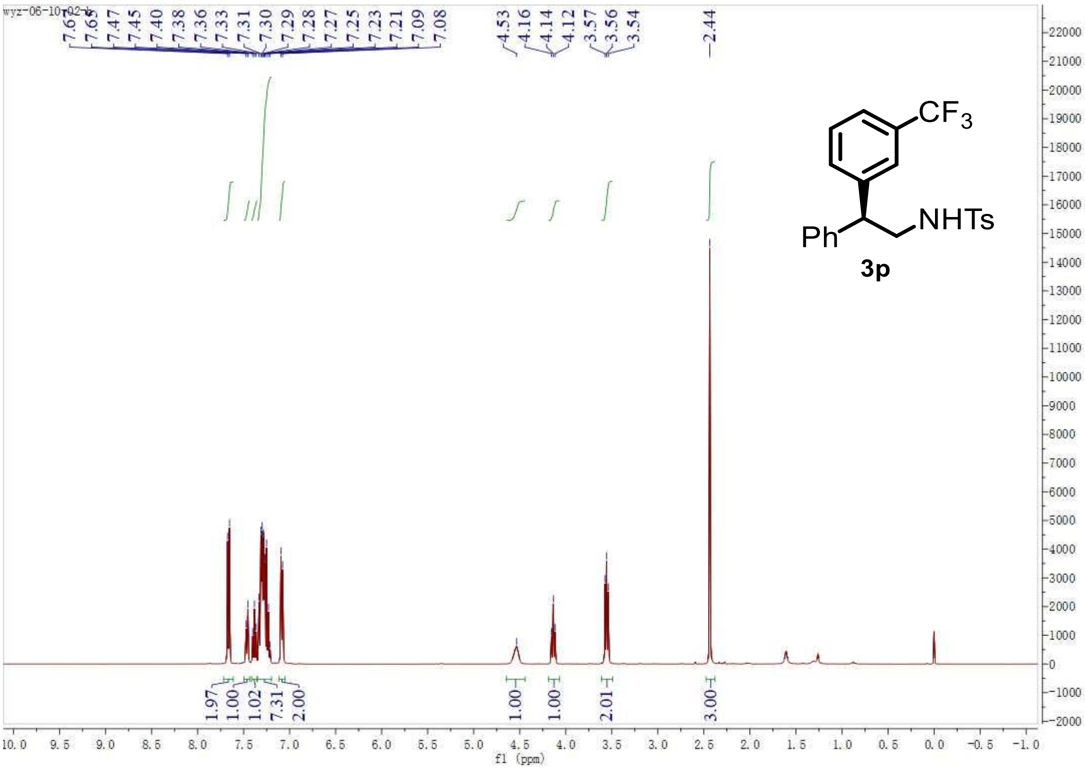

Supplementary Fig. 70. Compound 3p ^{13}C NMR (101 MHz, $CDCl_3$)

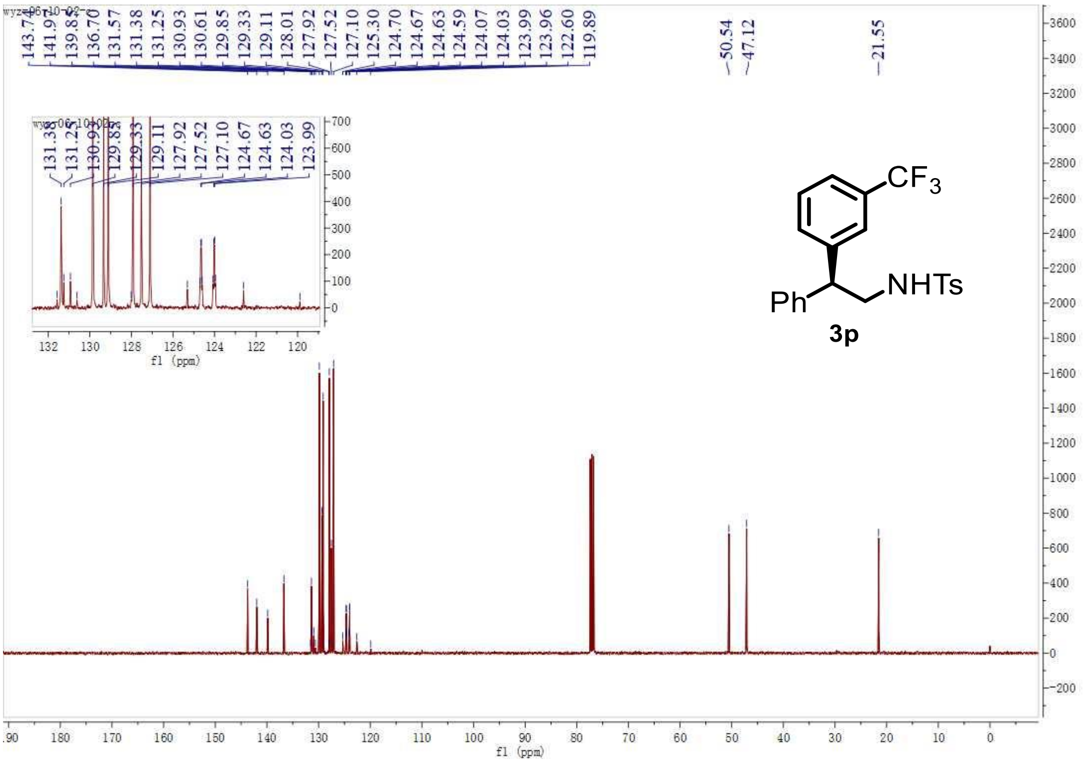

Supplementary Fig. 71. Compound 3p ^{19}F NMR (376 MHz, $CDCl_3$)

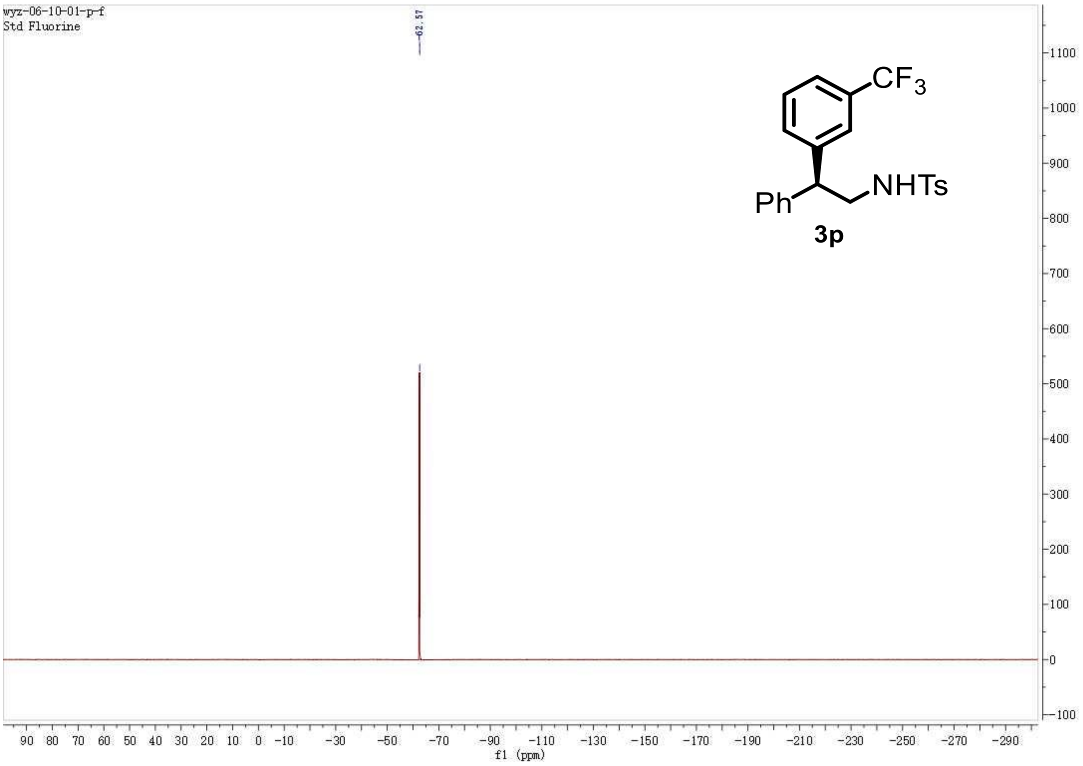

Supplementary Fig. 72. Compound 3q ^{1}H NMR (400 MHz, $CDCl_3$)

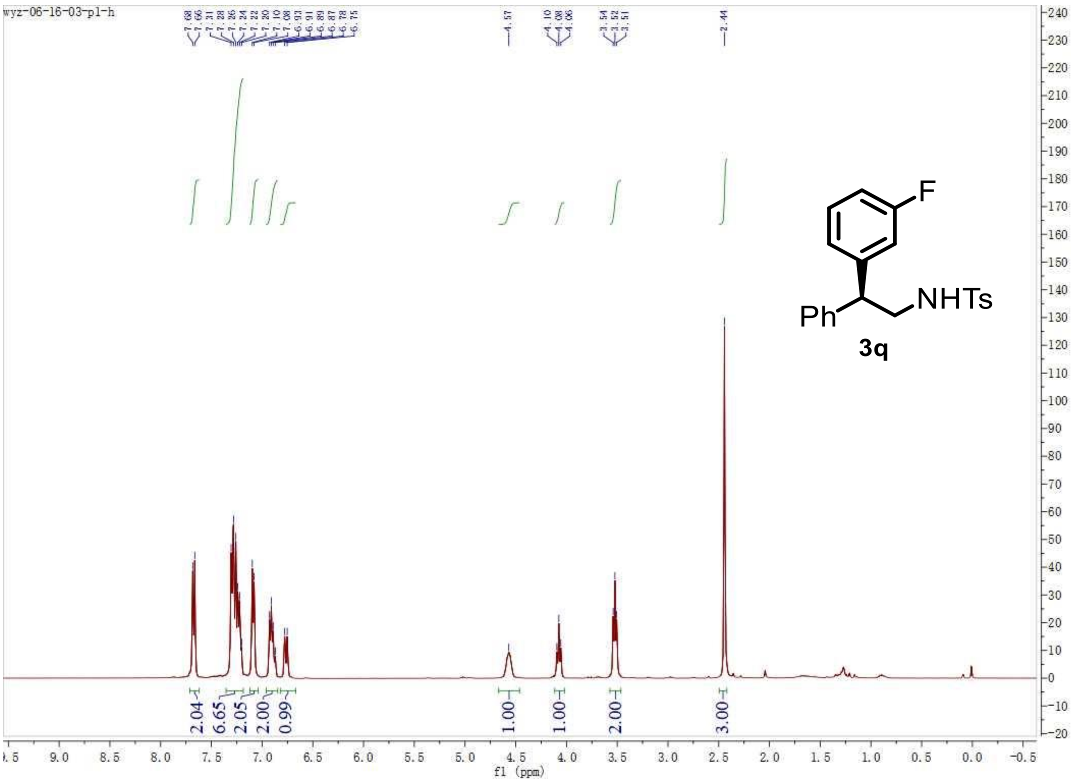

Supplementary Fig. 73. Compound 3q ^{13}C NMR (101 MHz, $CDCl_3$)

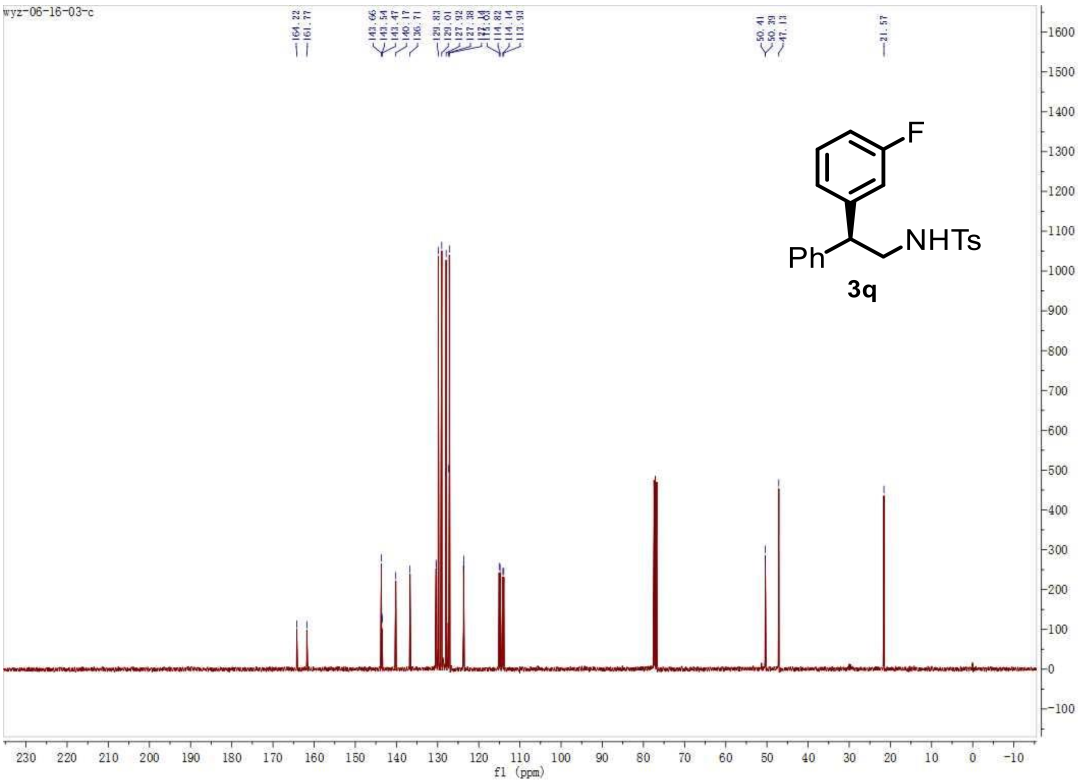

Supplementary Fig. 74. Compound 3q ^{19}F NMR (376 MHz, $CDCl_3$)

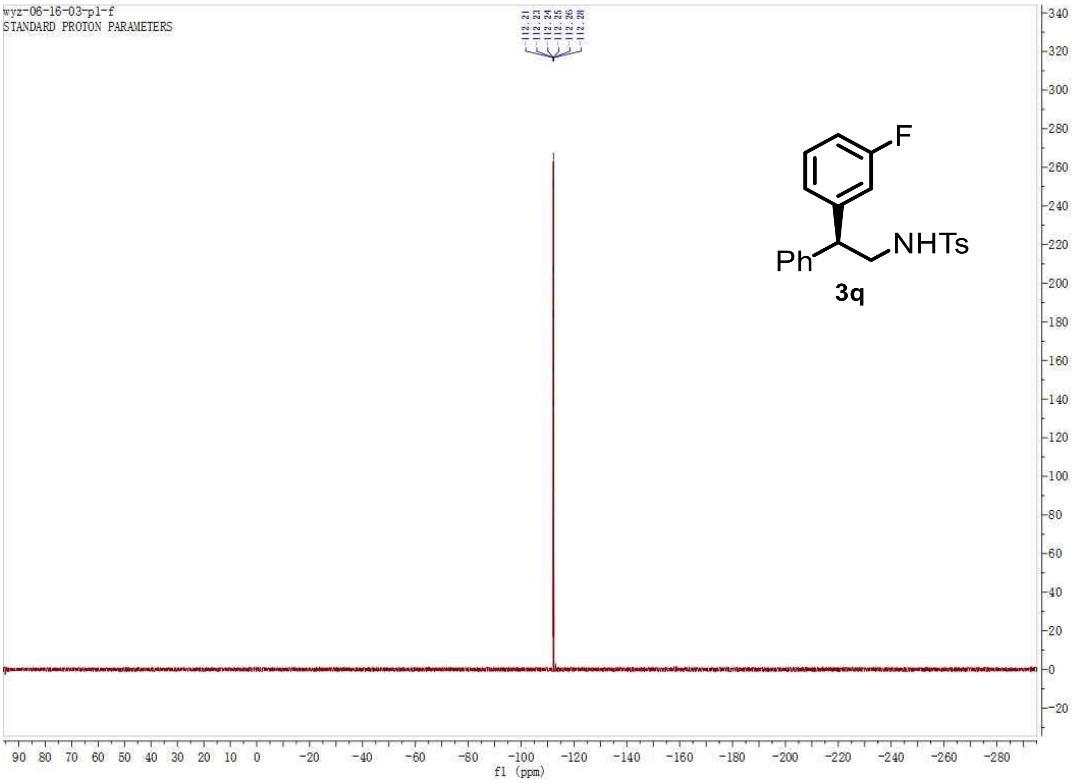

Supplementary Fig. 75. Compound 3r ^{1}H NMR (400 MHz, $CDCl_3$)

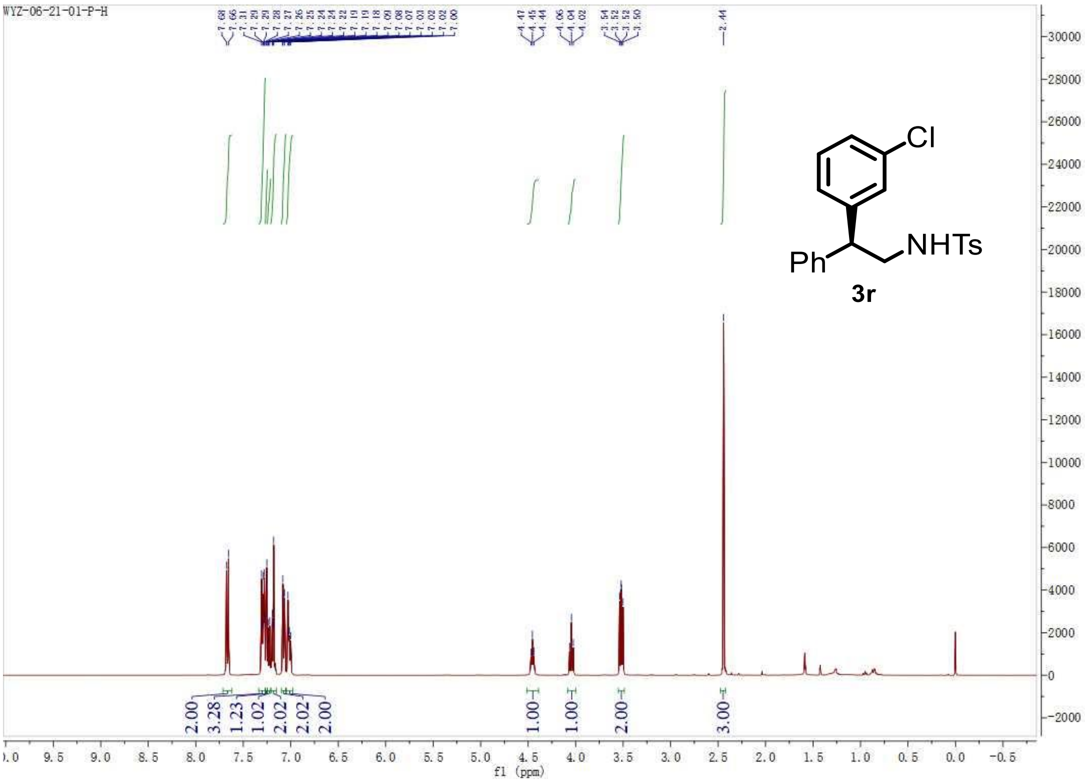

Supplementary Fig. 76. Compound 3r ^{13}C NMR (101 MHz, $CDCl_3$)

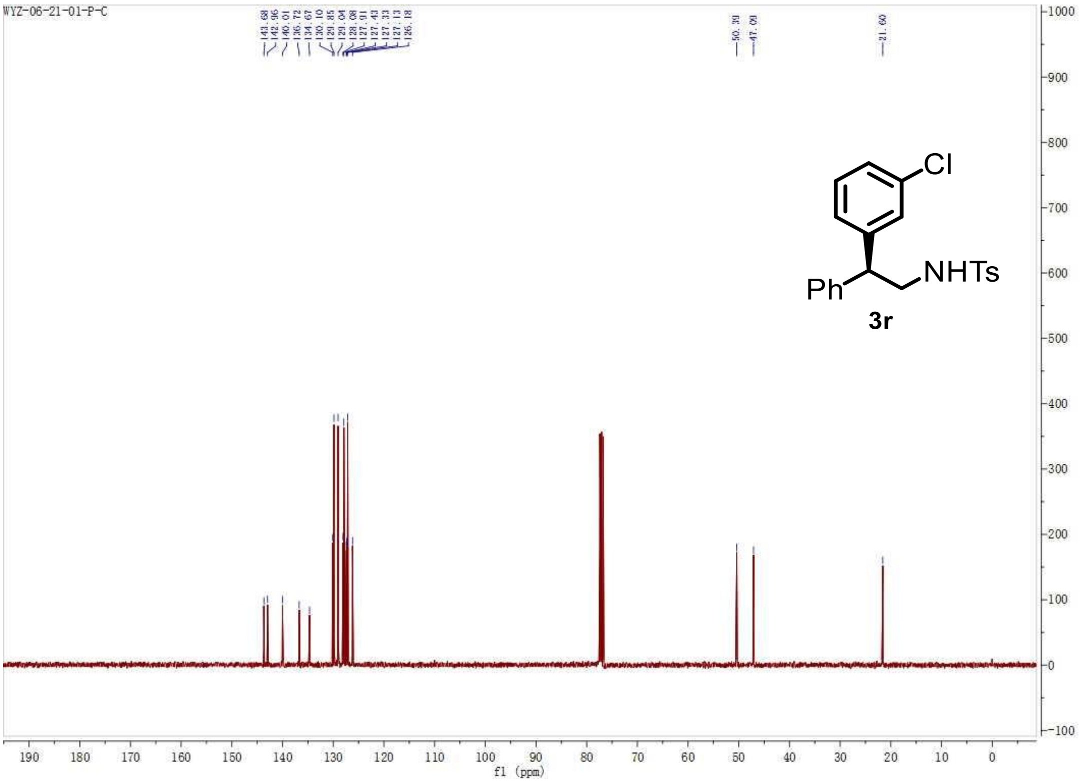

Supplementary Fig. 77. Compound 3s ^{1}H NMR (400 MHz, $CDCl_3$)

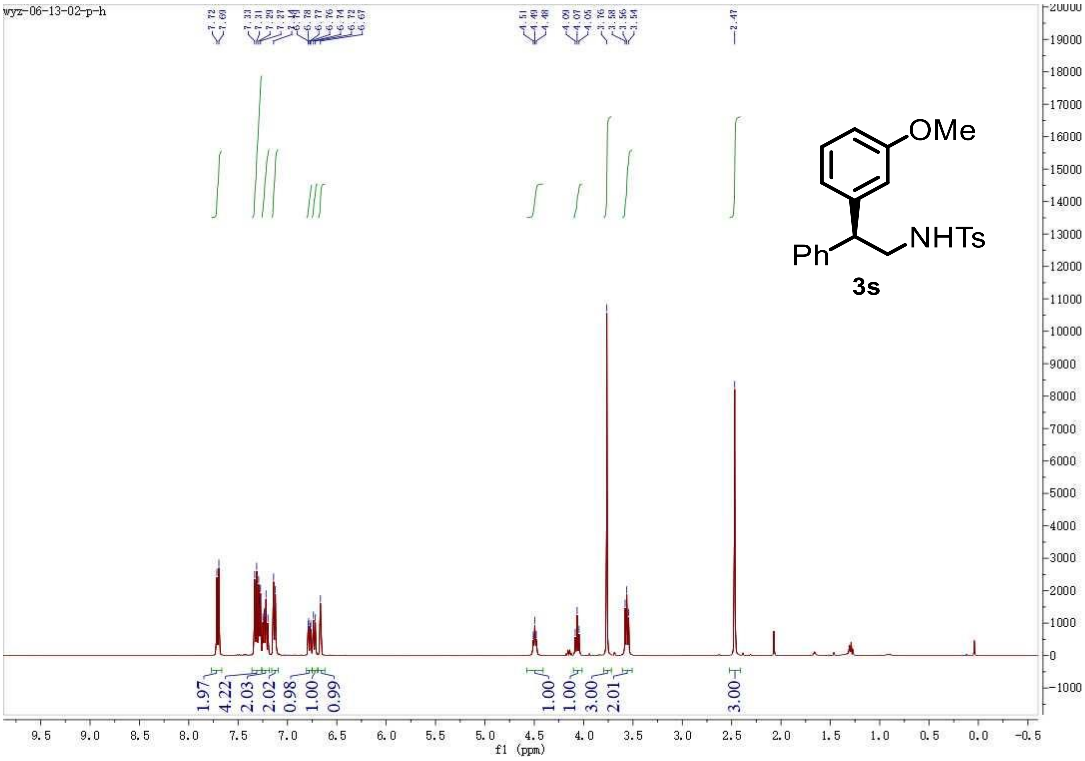

Supplementary Fig. 78. Compound 3s ^{13}C NMR (101 MHz, $CDCl_3$)

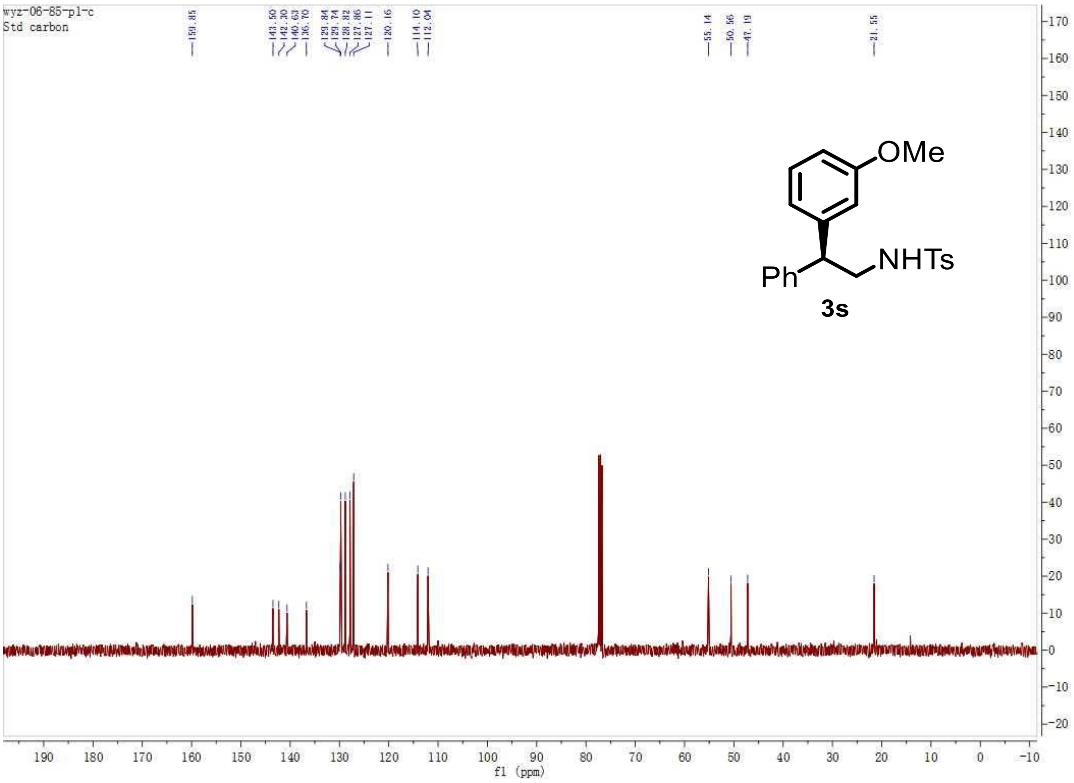

Supplementary Fig. 79. Compound 3t ^{1}H NMR (400 MHz, $CDCl_3$)

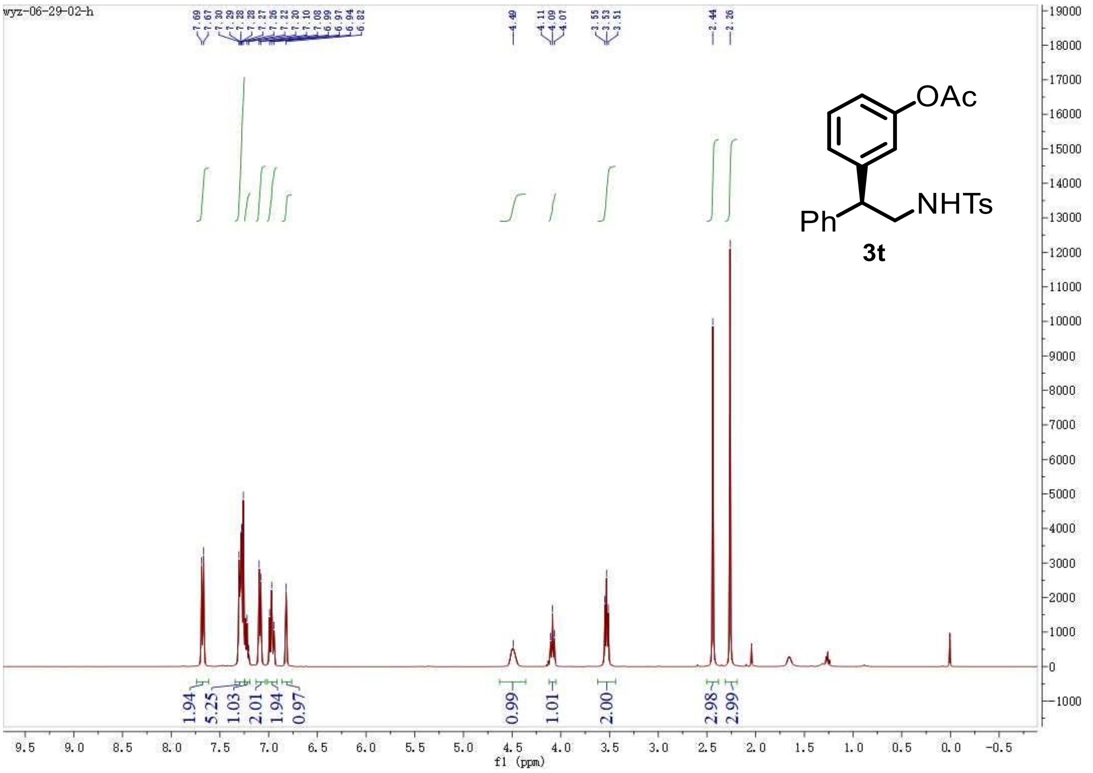

Supplementary Fig. 80. Compound 3t ^{13}C NMR (101 MHz, $CDCl_3$)

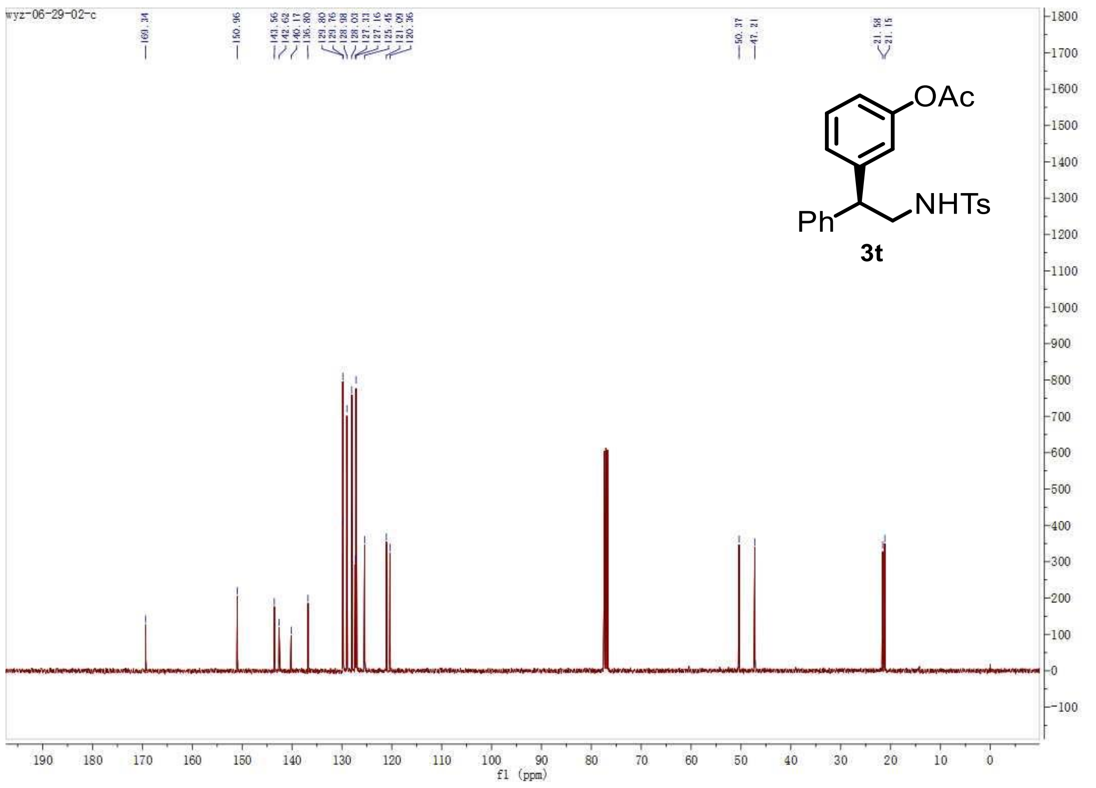

Supplementary Fig. 81. Compound 3u ^{1}H NMR (400 MHz, $CDCl_3$)

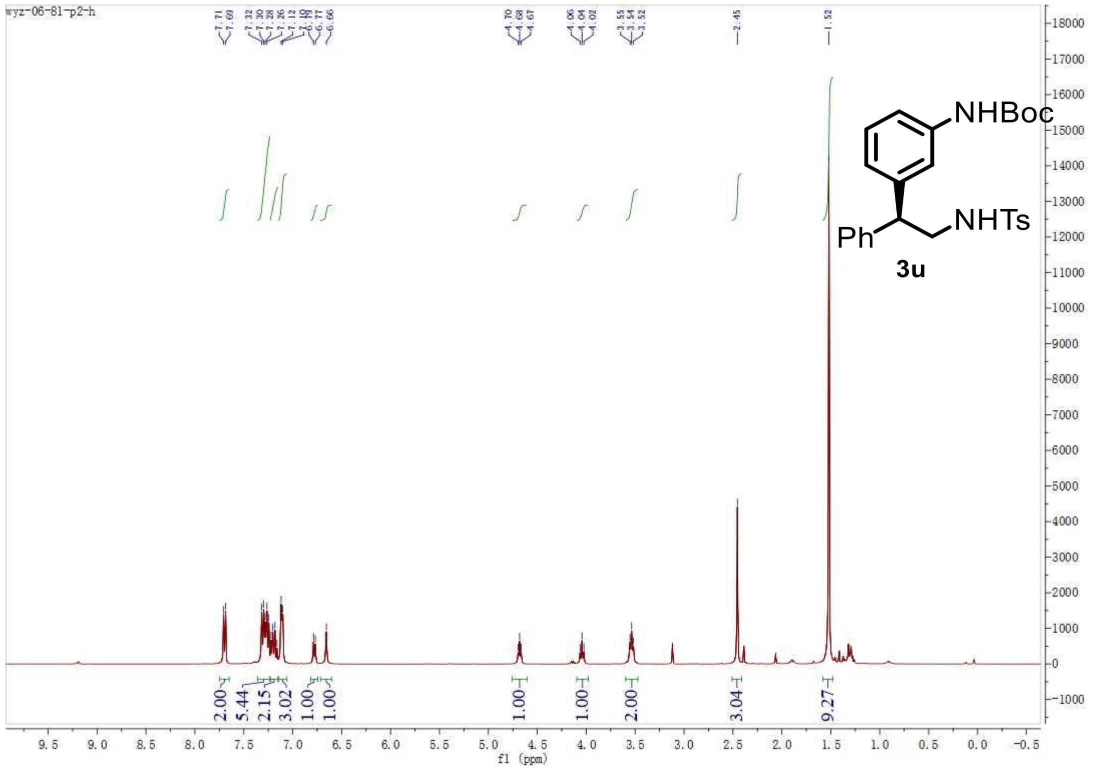

Supplementary Fig. 82. Compound 3u ^{13}C NMR (101 MHz, $CDCl_3$)

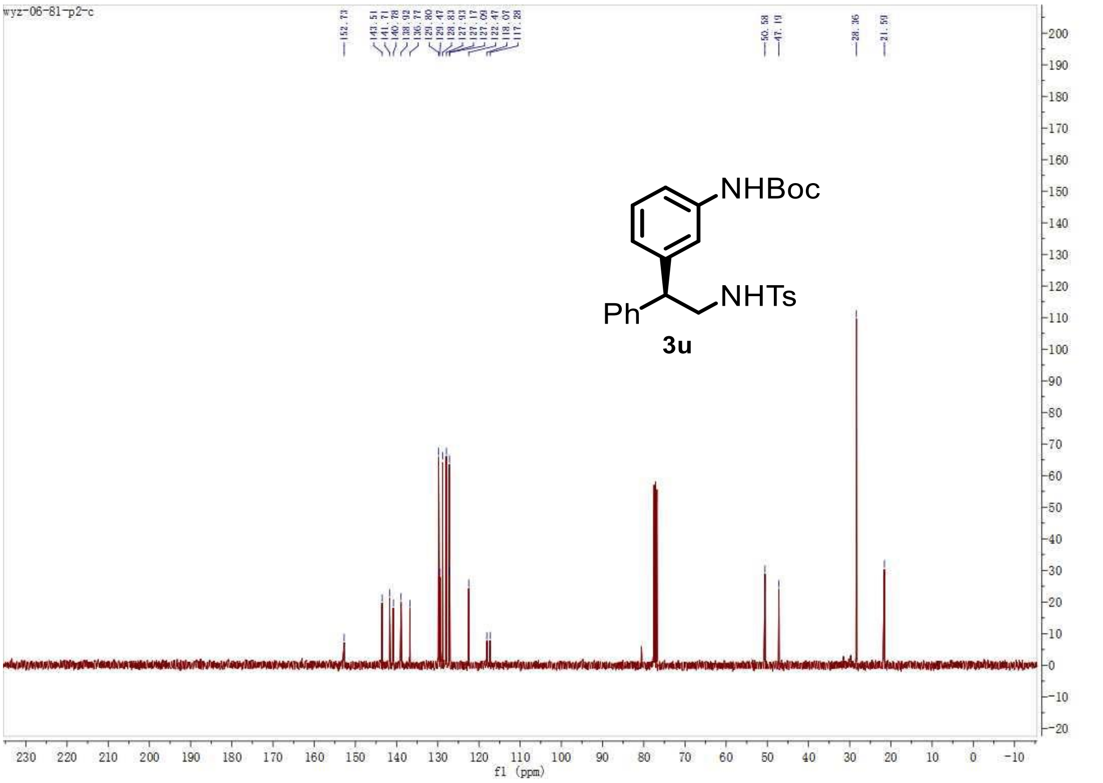

Supplementary Fig. 83. Compound 3v ^{1}H NMR (400 MHz, $CDCl_3$)

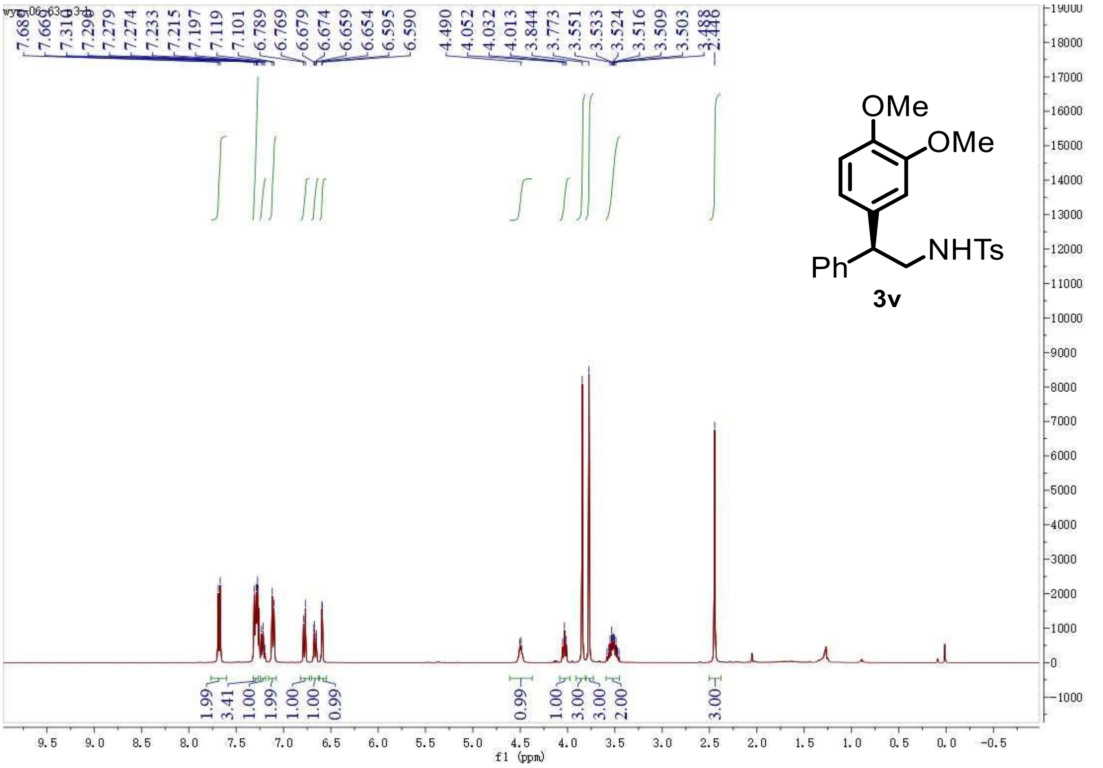

Supplementary Fig. 84. Compound 3v ^{13}C NMR (101 MHz, $CDCl_3$)

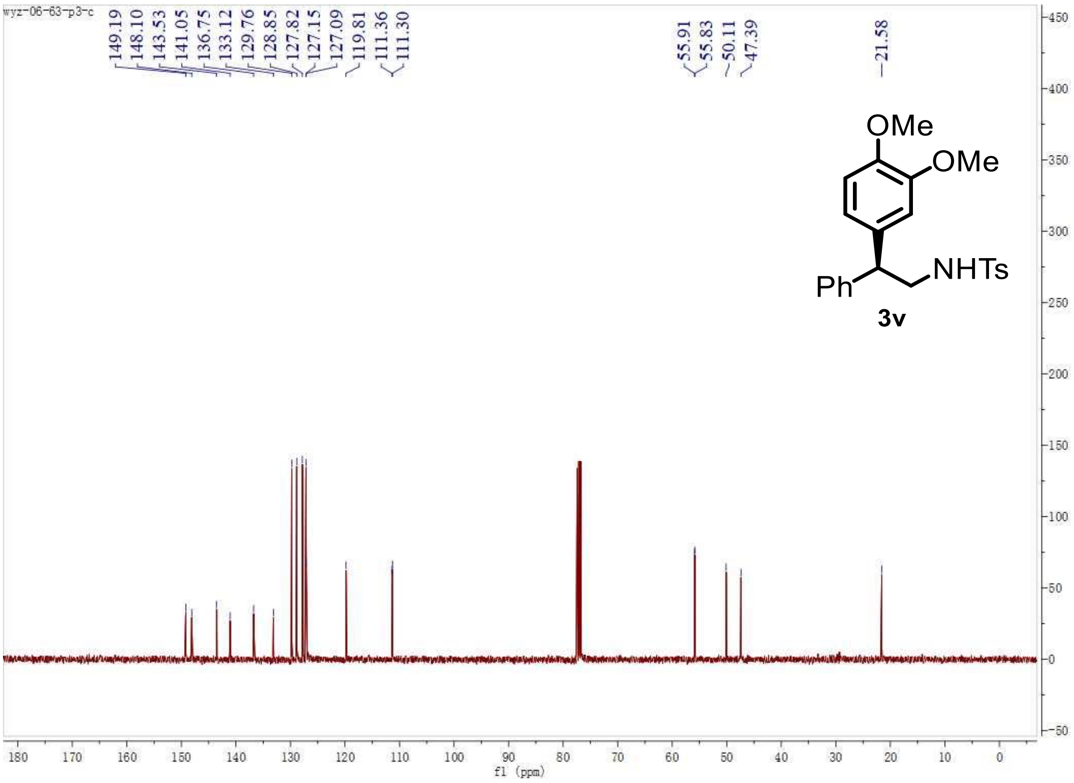

Supplementary Fig. 85. Compound 3w ^{1}H NMR (400 MHz, $CDCl_3$)

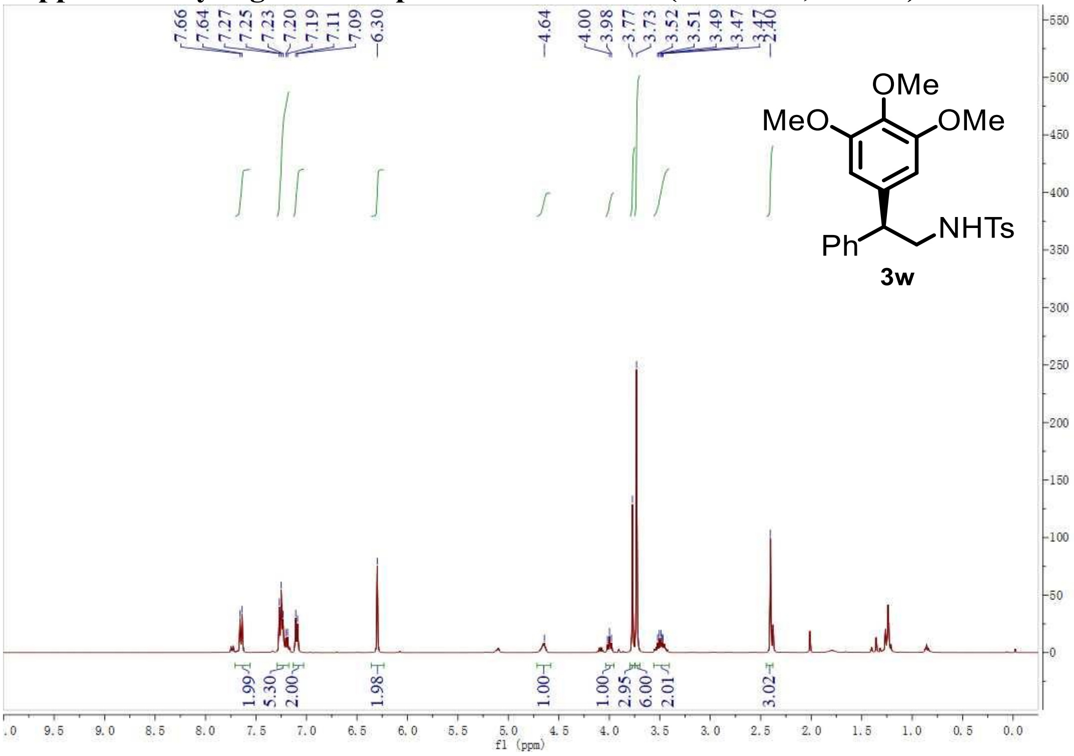

Supplementary Fig. 86. Compound 3w ^{13}C NMR (101 MHz, $CDCl_3$)

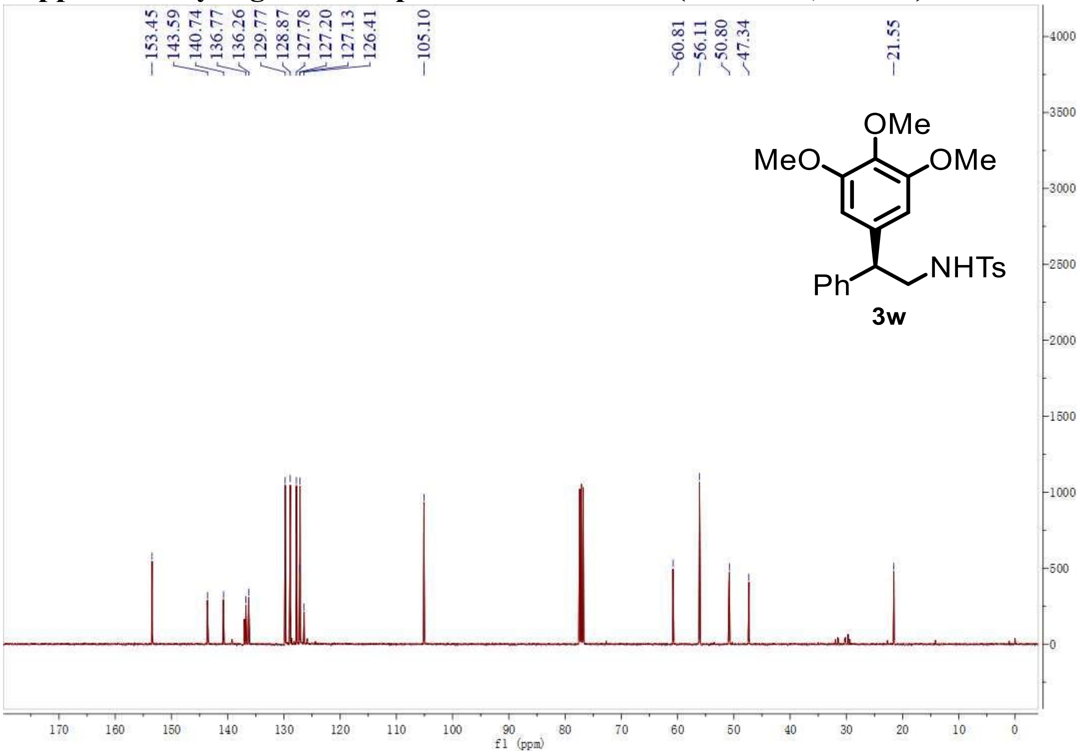

Supplementary Fig. 87. Compound 3x ^{1}H NMR (400 MHz, $CDCl_3$)

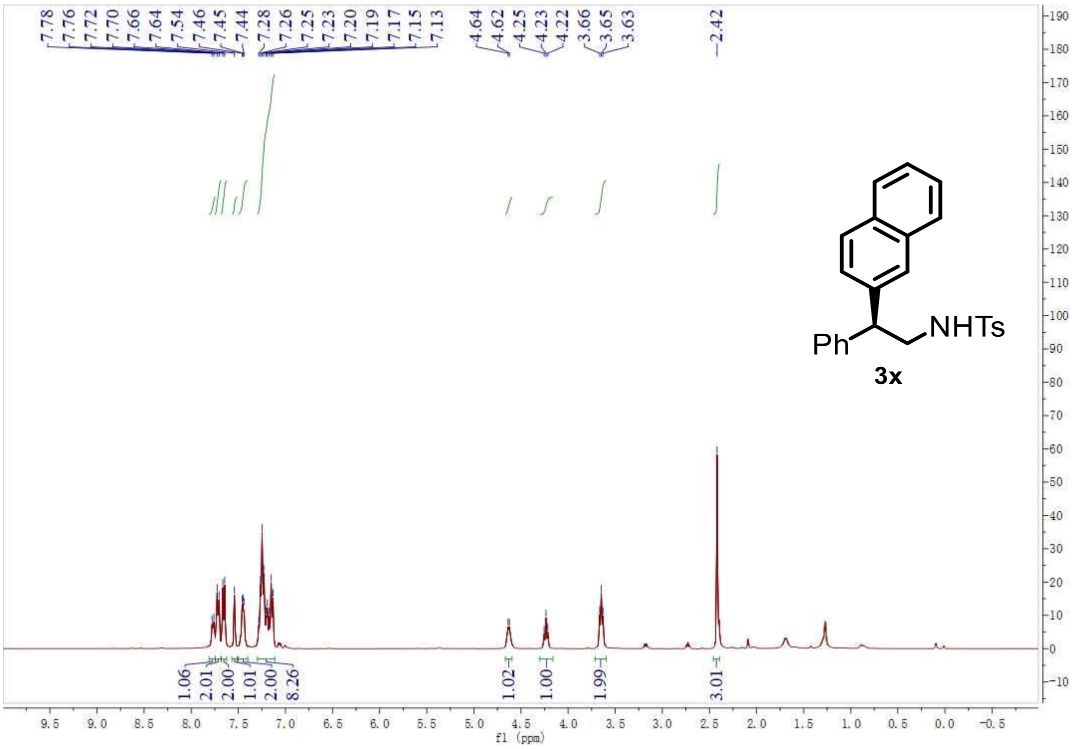

Supplementary Fig. 88. Compound 3x ^{13}C NMR (101 MHz, $CDCl_3$)

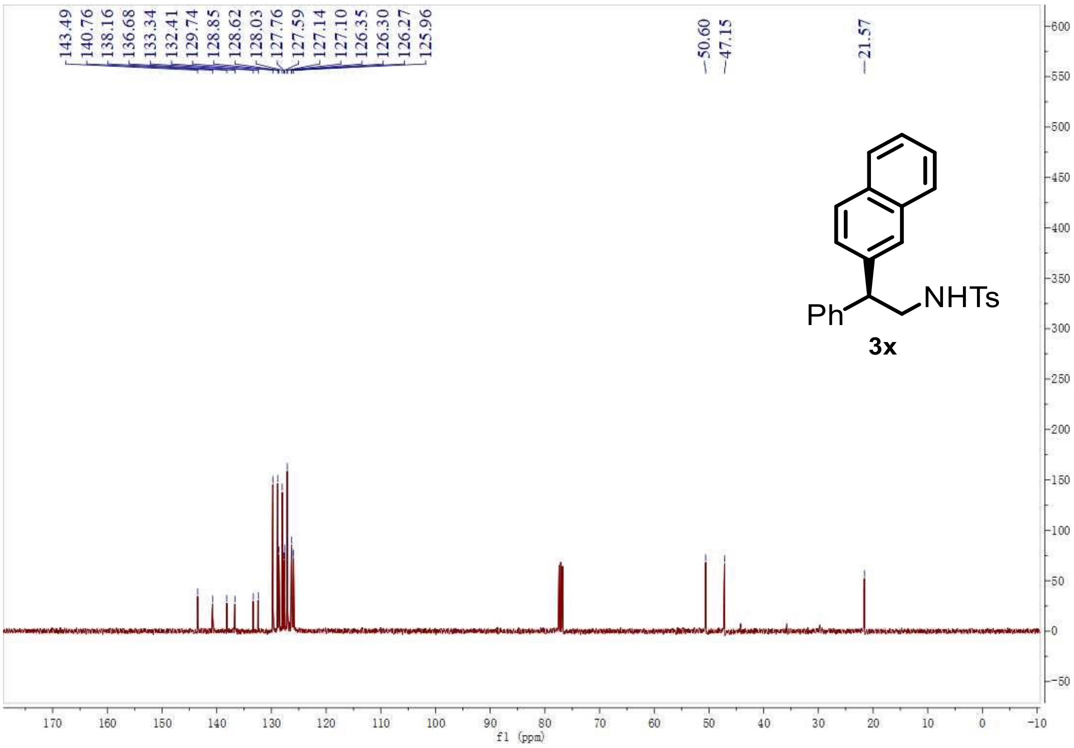

Supplementary Fig. 89. Compound 3y ^{1}H NMR (400 MHz, $CDCl_3$)

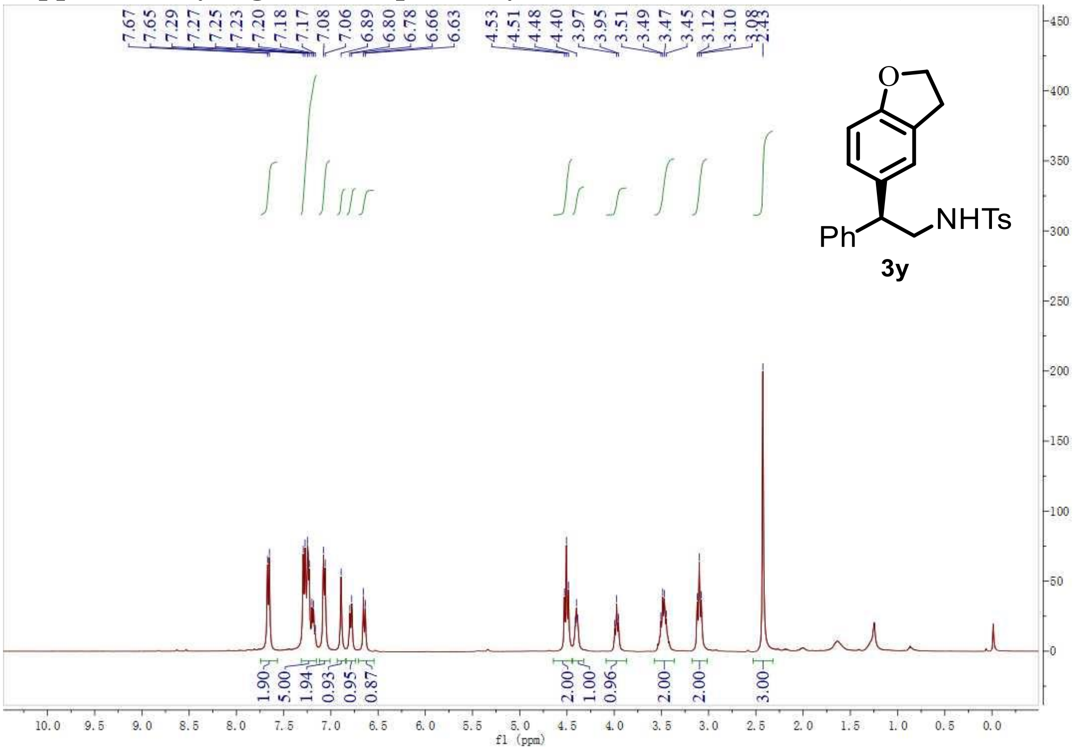

Supplementary Fig. 90. Compound 3y ^{13}C NMR (101 MHz, CDCl$_3$)

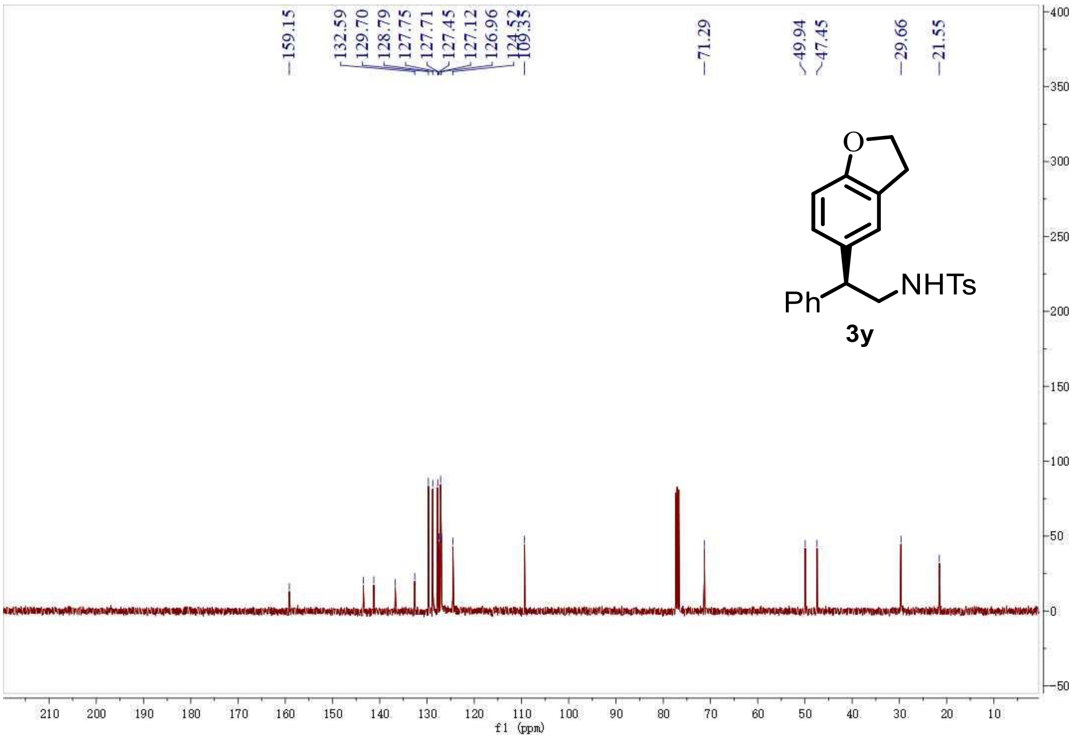

Supplementary Fig. 91. Compound 3z ^{1}H NMR (400 MHz, CDCl$_3$)

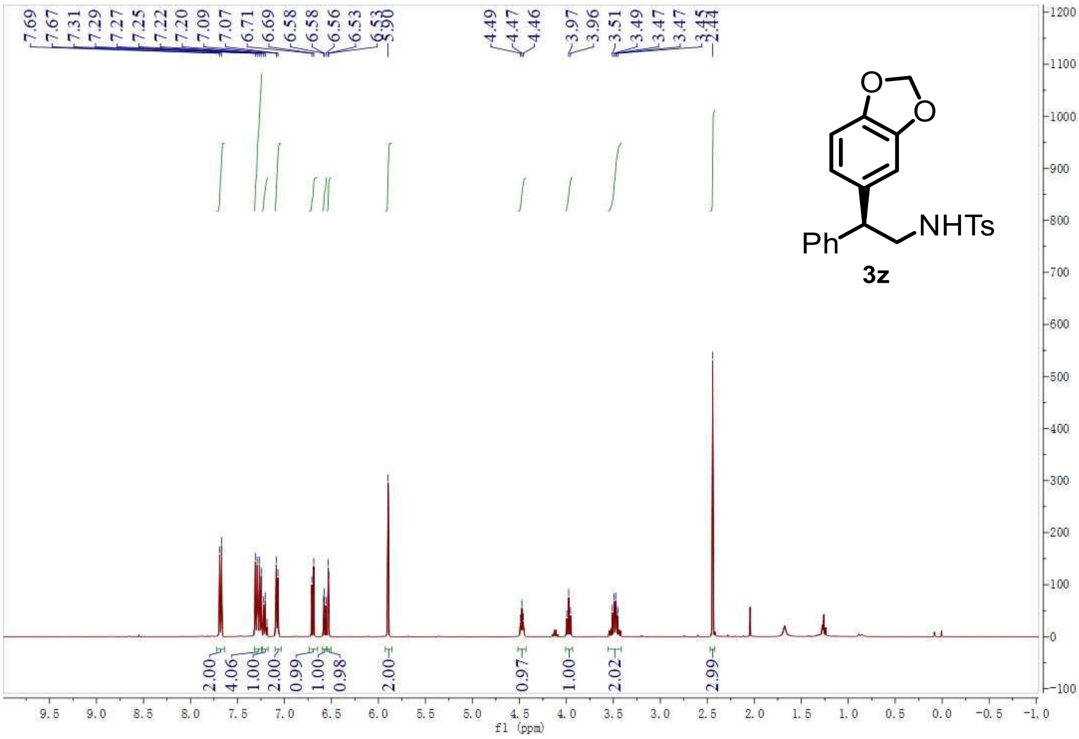

Supplementary Fig. 92. Compound 3z ^{13}C NMR (101 MHz, $CDCl_3$)

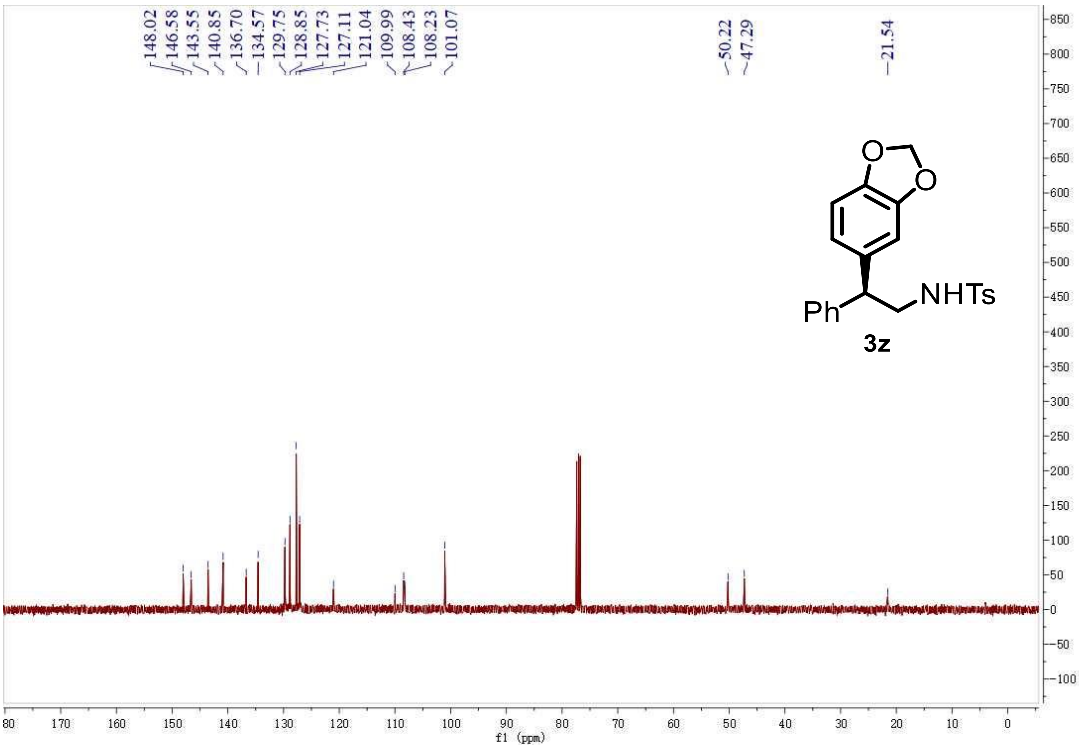

Supplementary Fig. 93. Compound 3aa ^{1}H NMR (400 MHz, $CDCl_3$)

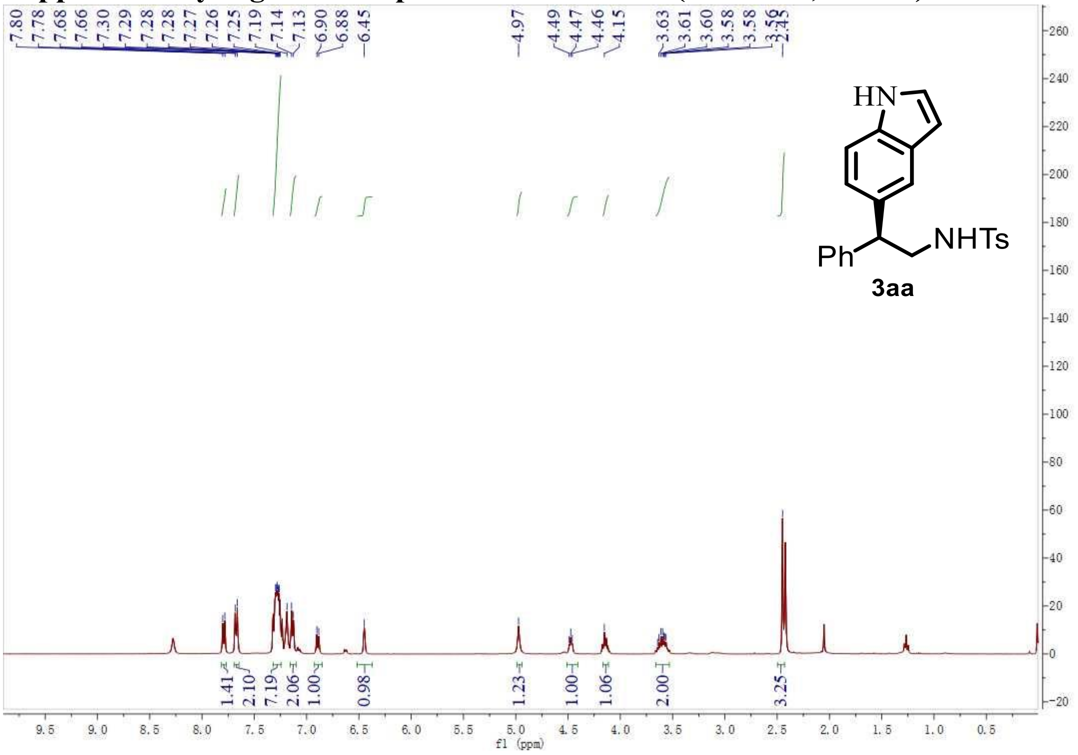

Supplementary Fig. 94. Compound 3aa ^{13}C NMR (101 MHz, $CDCl_3$)

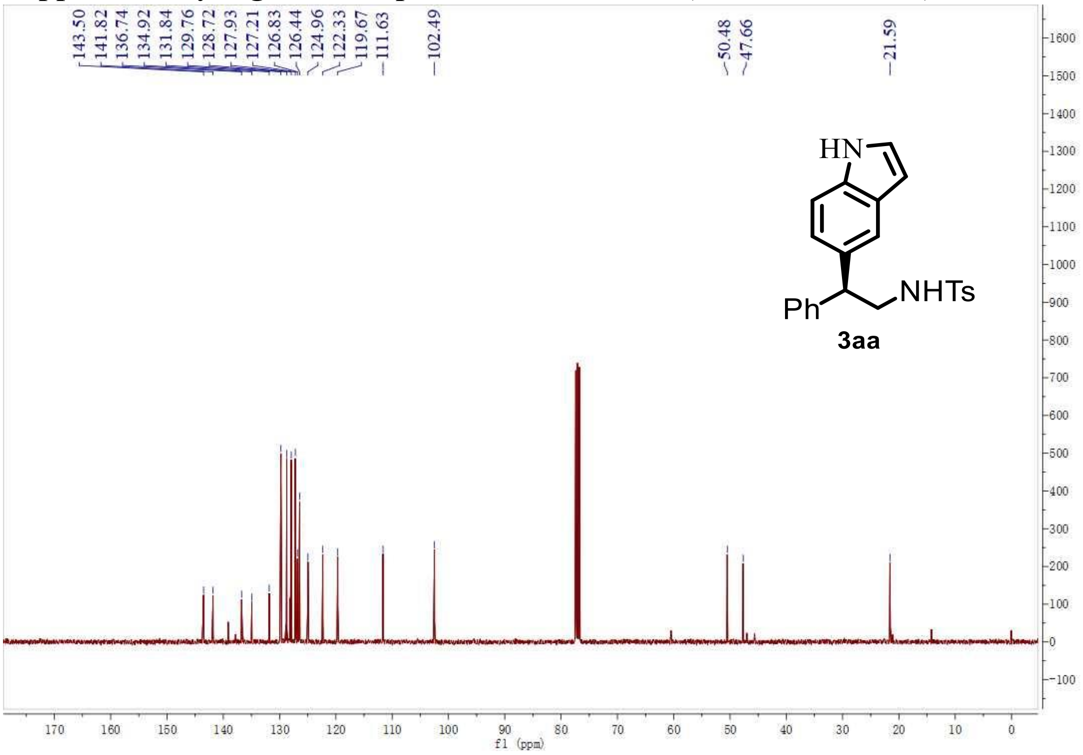

Supplementary Fig. 95. Compound 3ab ^{1}H NMR (400 MHz, $CDCl_3$)

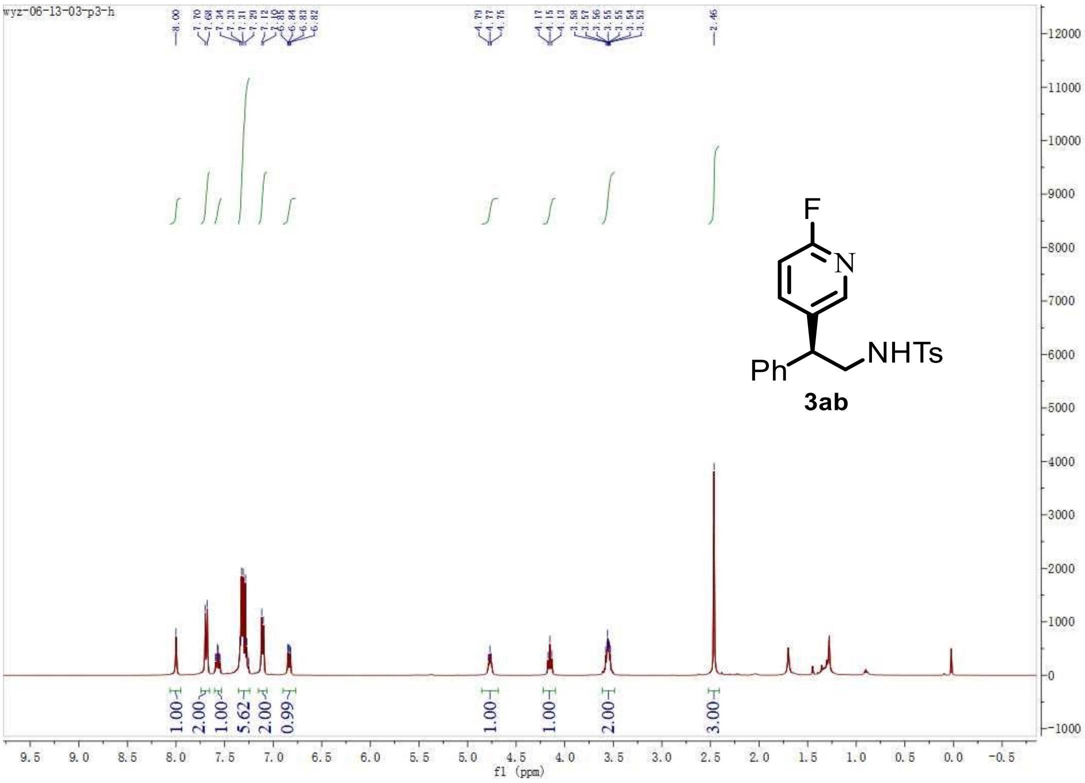

Supplementary Fig. 96. Compound 3ab ^{13}C NMR (101 MHz, $CDCl_3$)

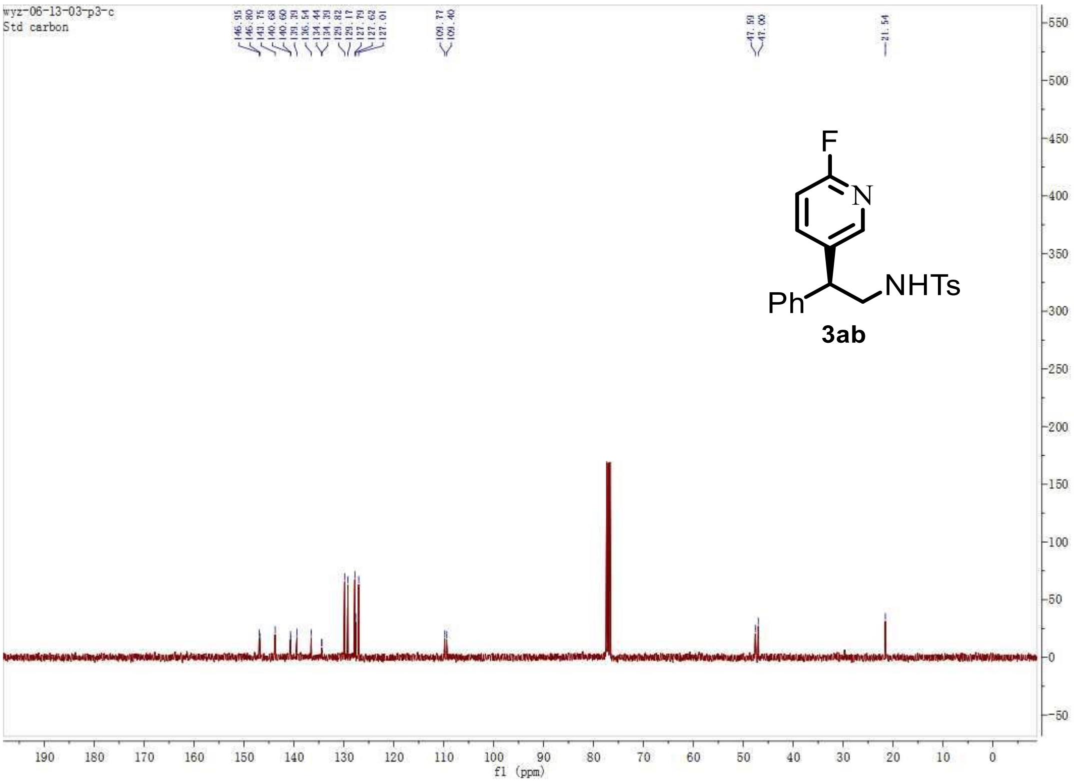

Supplementary Fig. 97. Compound 3ab^{19}F NMR (376 MHz, $CDCl_3$)

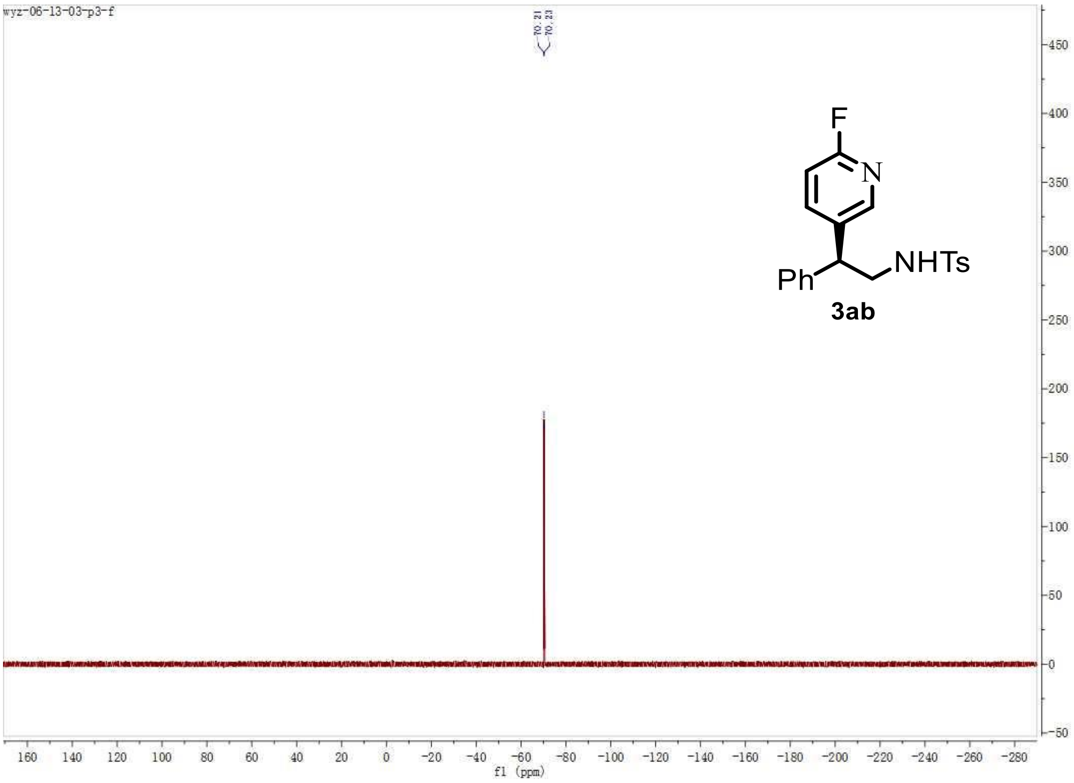

Supplementary Fig. 98. Compound 3ac ^{1}H NMR (400 MHz, $CDCl_3$)

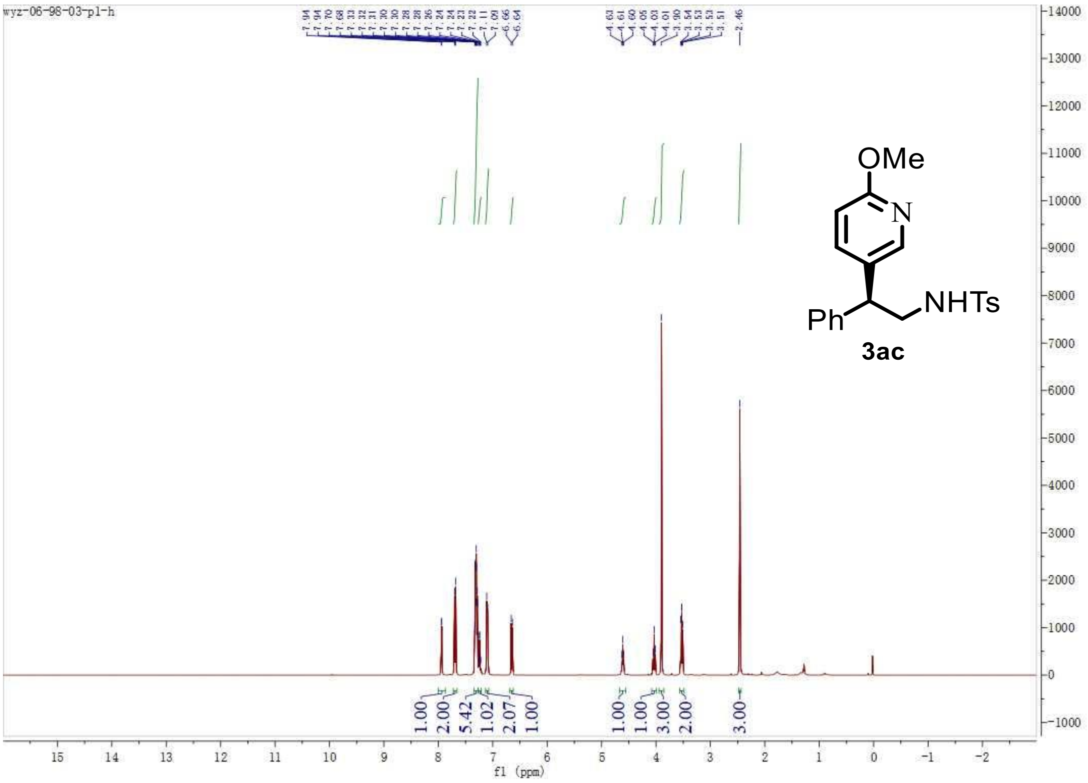

Supplementary Fig. 99. Compound 3ac ^{13}C NMR (101 MHz, $CDCl_3$)

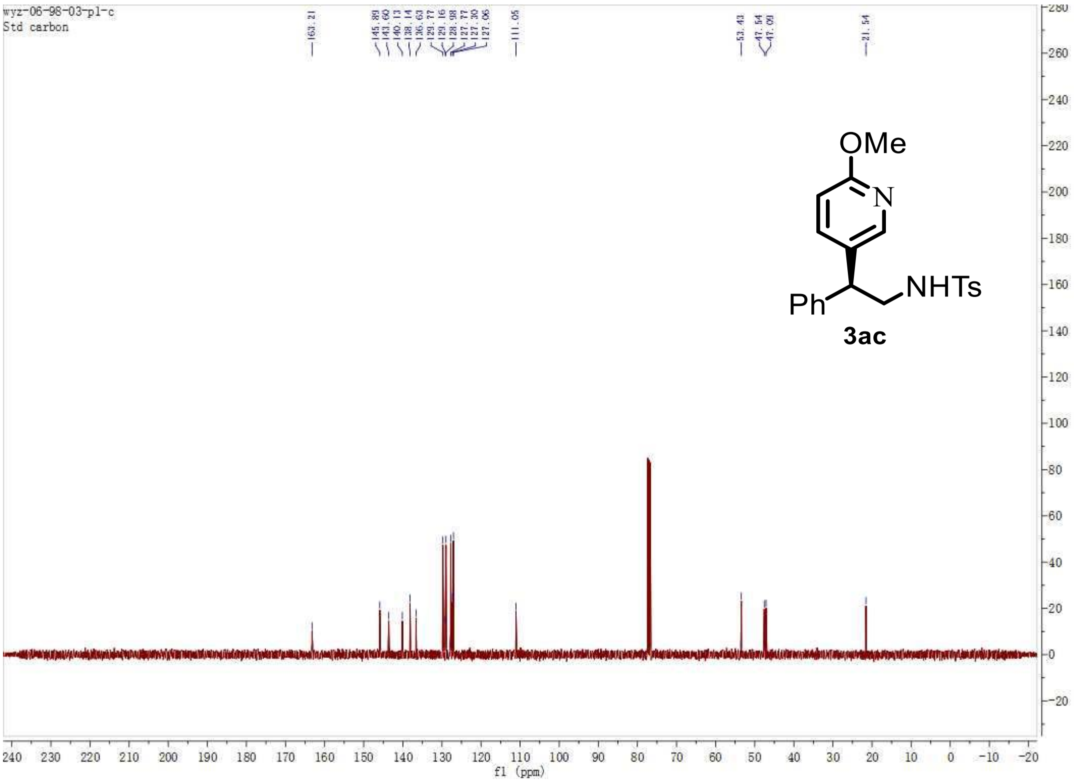

Supplementary Fig. 100. Compound 3ad ^{1}H NMR (400 MHz, $CDCl_3$)

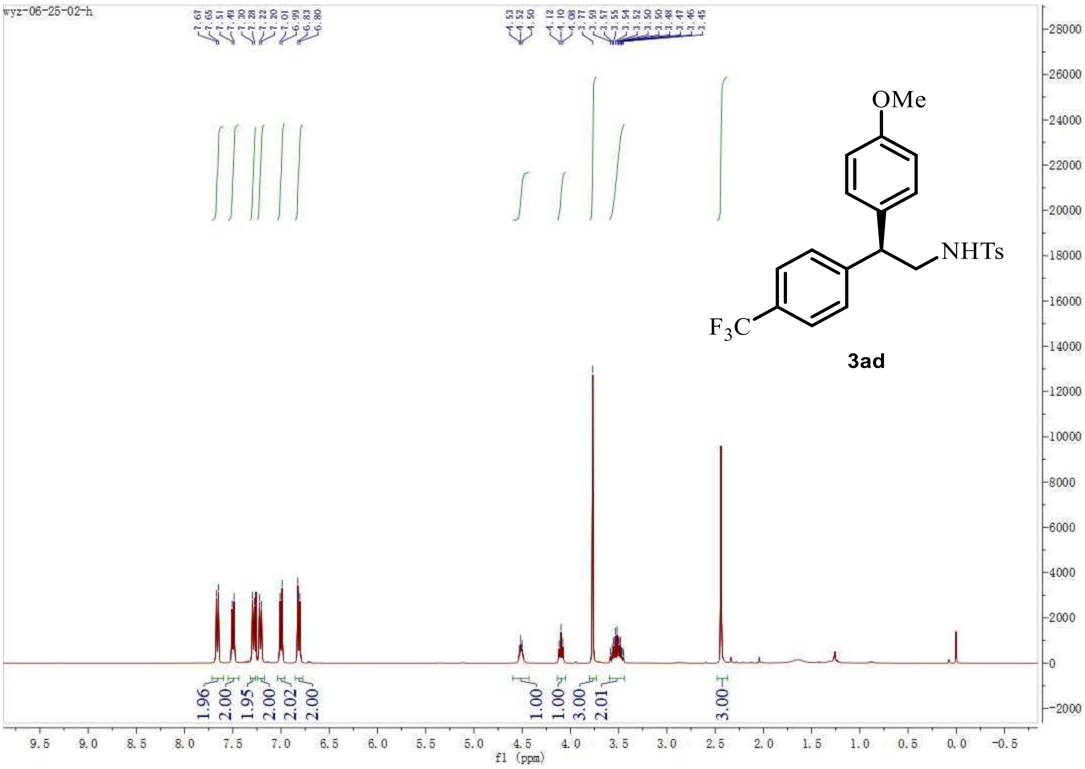

Supplementary Fig. 101. Compound 3ad ^{13}C NMR (101 MHz, $CDCl_3$)

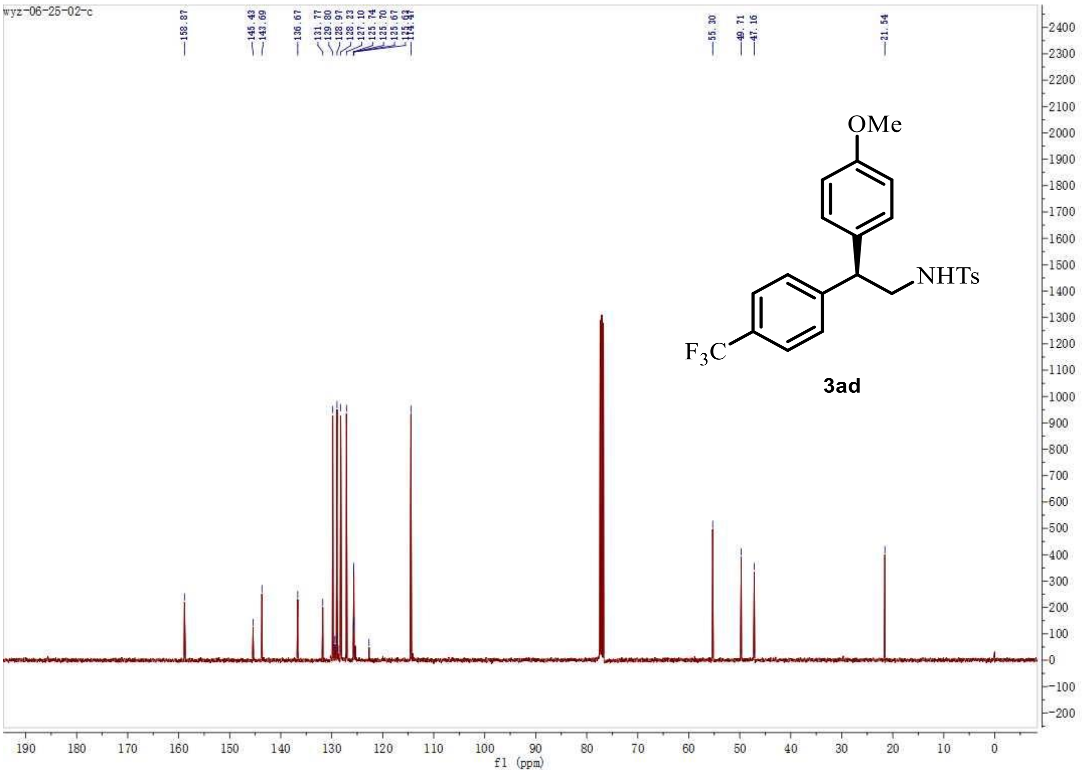

Supplementary Fig. 102. Compound 3ae ^{1}H NMR (400 MHz, $CDCl_3$)

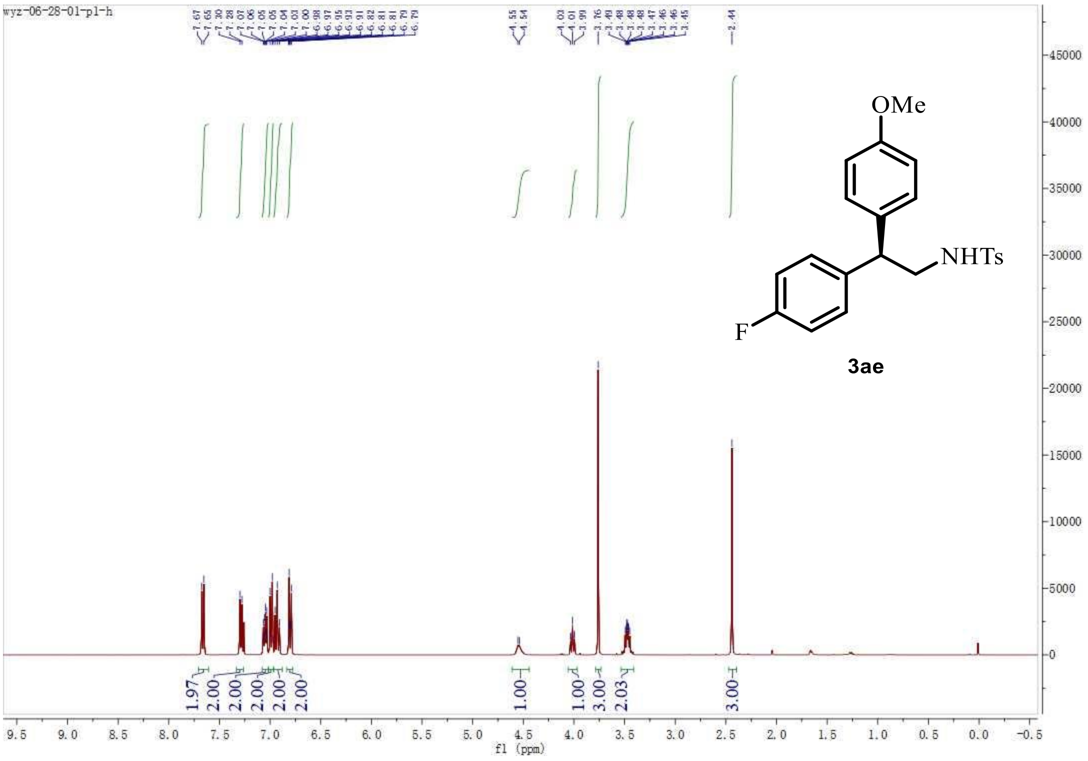

Supplementary Fig. 103. Compound 3ae ^{13}C NMR (101 MHz, $CDCl_3$)

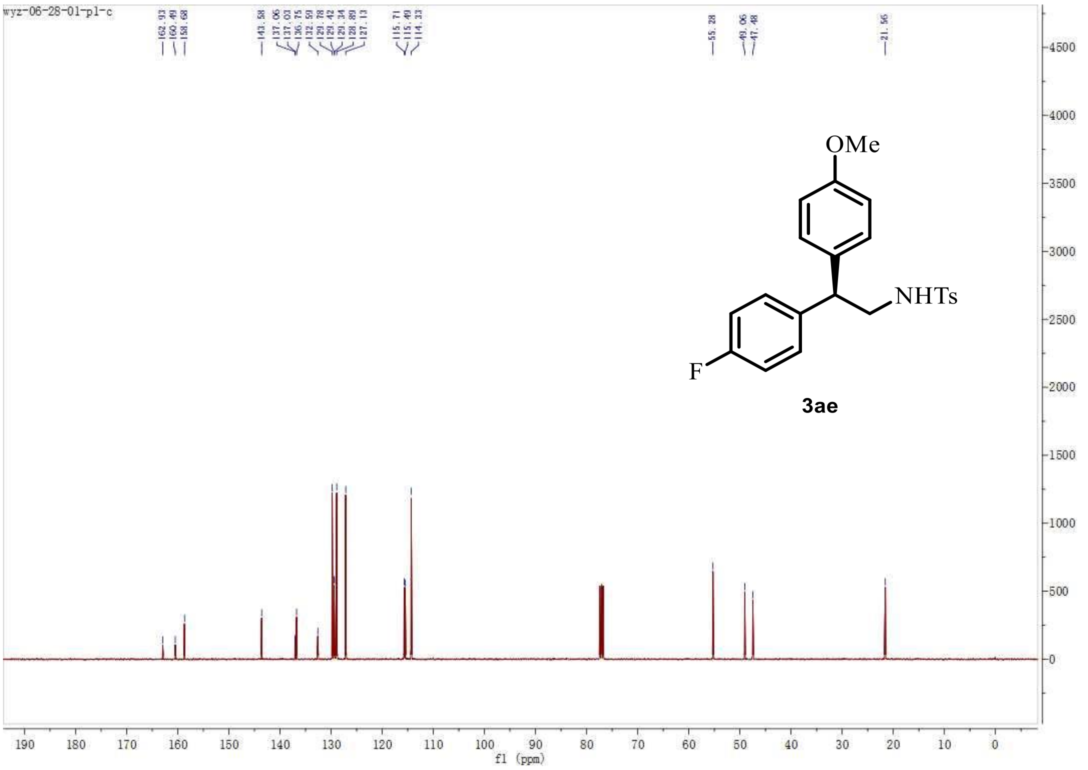

Supplementary Fig. 104. Compound 3af ^{1}H NMR (400 MHz, $CDCl_3$)

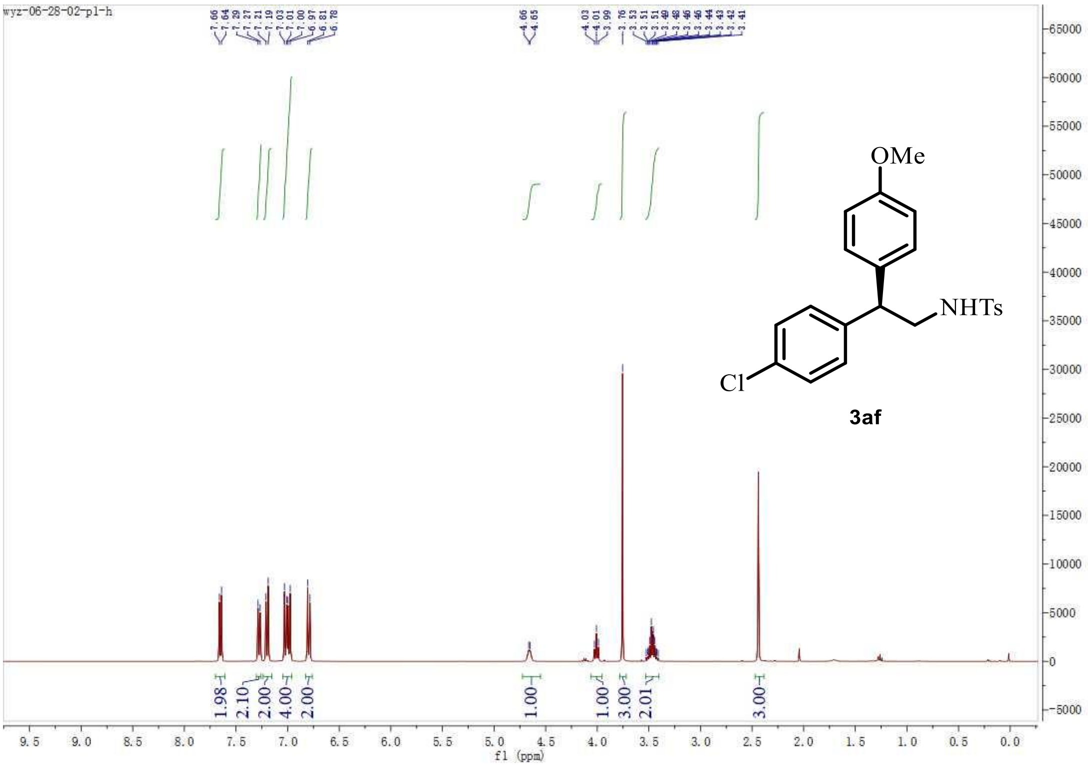

Supplementary Fig. 105. Compound 3af ^{13}C NMR (101 MHz, $CDCl_3$)

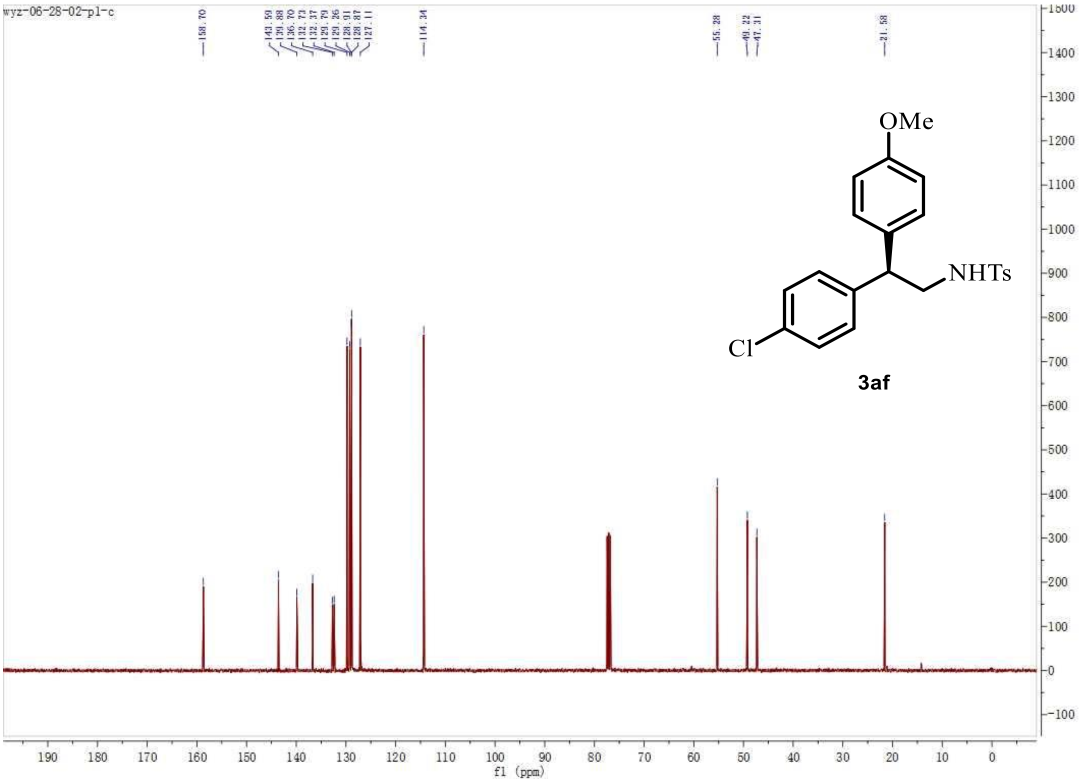

Supplementary Fig. 106. Compound 3ag ^{1}H NMR (400 MHz, $CDCl_3$)

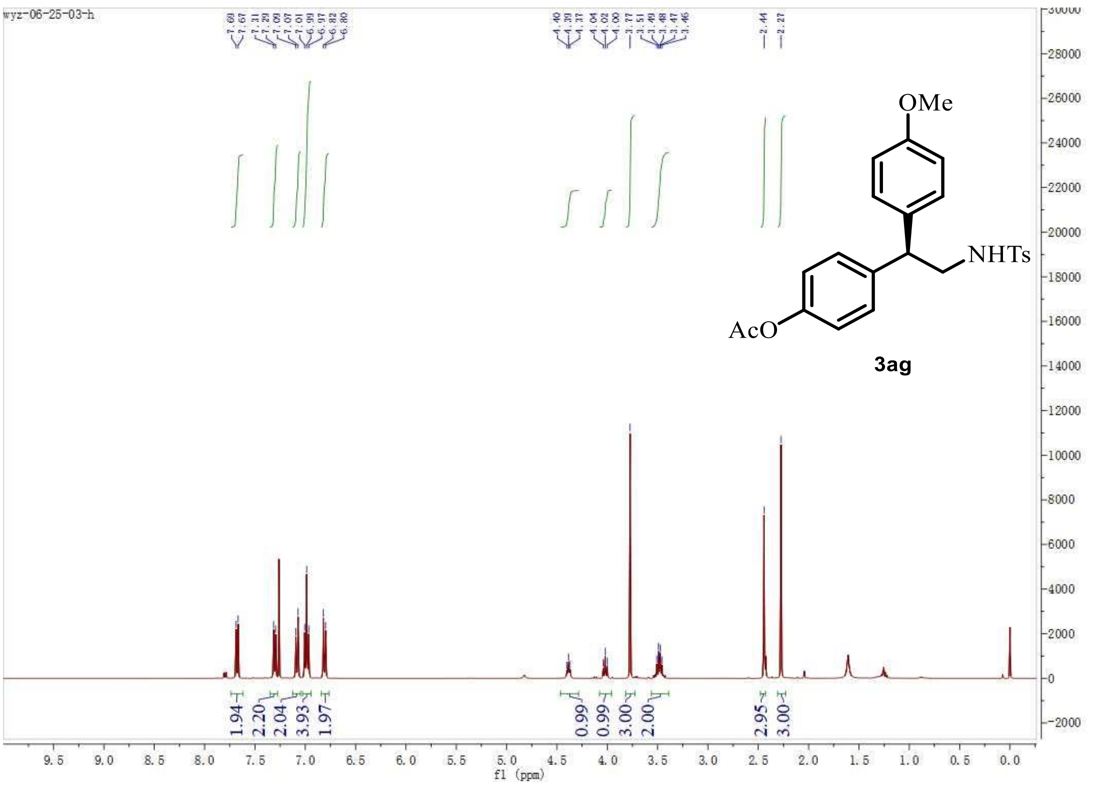

Supplementary Fig. 107. Compound 3ag ^{13}C NMR (101 MHz, $CDCl_3$)

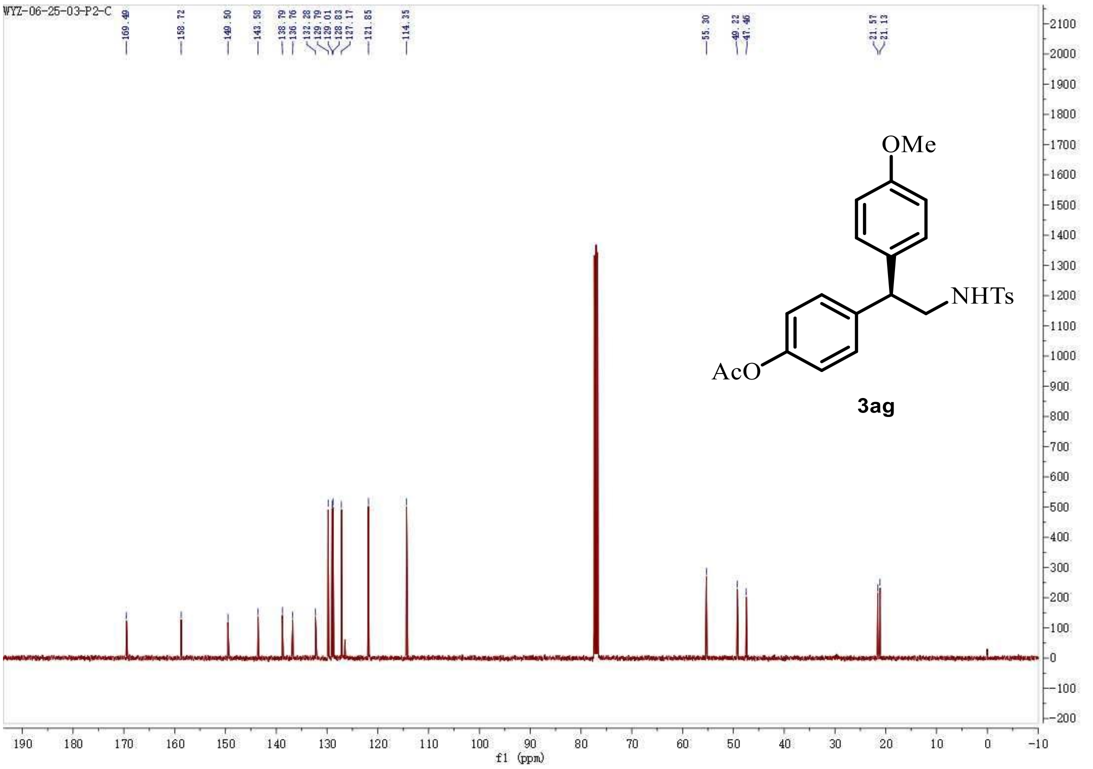

3.4 HPLC Spectra Data

Supplementary Figure 108. HPLC Chromatography of the Racemic N-(2-(4-methoxyphenyl)-2-phenylethyl)-4-methylbenzenesulfonamide (**3a**) (Daicel Chiralpak IC Column, *n*-Hexane: *i*-PrOH = 85:15, flow rate 1.0 mL/min, T = 25 °C, λ = 220 nm)

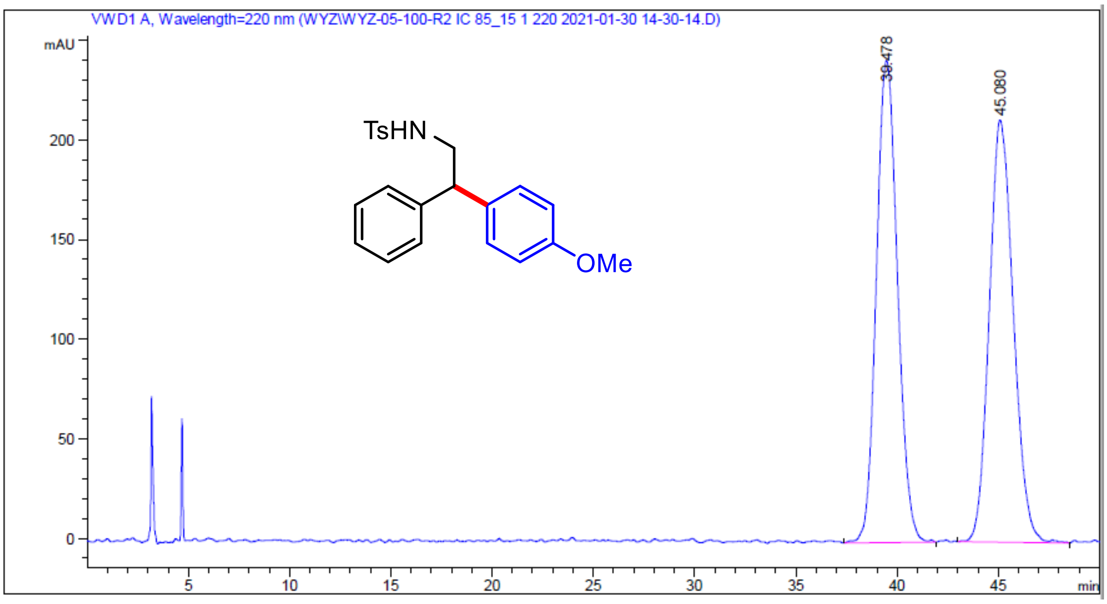

Peak NO	Ret. Time(min)	Area/%
1	39.478	50.1016
2	45.080	49.8984

Supplementary Figure 109. HPLC Chromatography of the (S)-N-(2-(4-methoxyphenyl)-2-phenylethyl)-4-methylbenzenesulfonamide (**3a**) (Daicel Chiralpak IC Column, *n*-Hexane: *i*-PrOH = 85:15, flow rate 1.0 mL/min, T = 25 °C, λ = 220 nm)

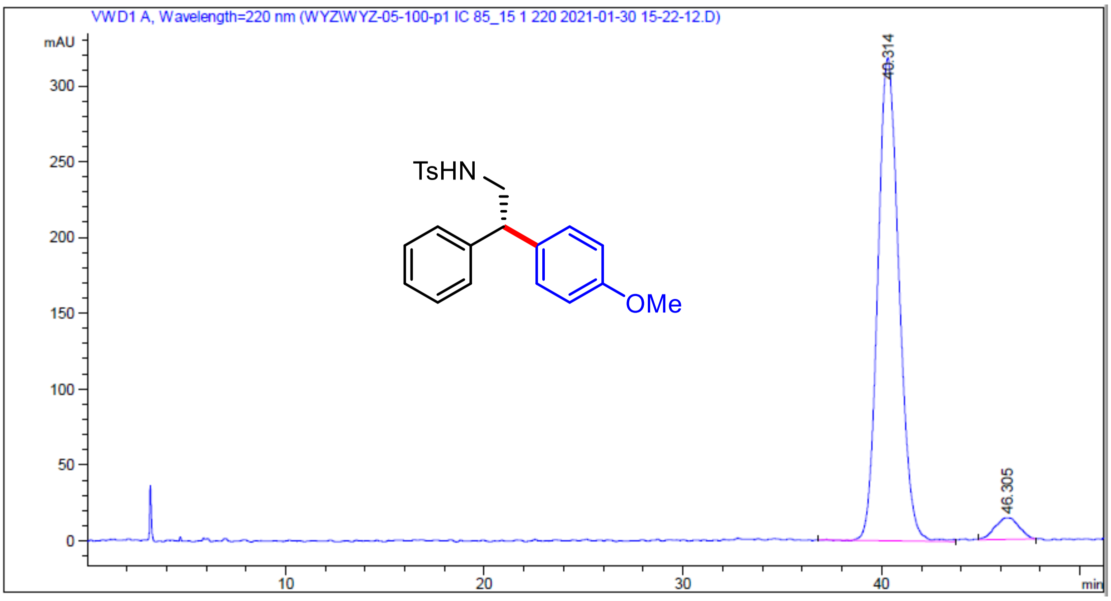

Peak NO	Ret. Time(min)	Area/%
1	40.314	95.1471
2	46.305	4.8529

Supplementary Figure 110. HPLC Chromatography of the Racemic 4-methyl-N-(2-phenyl-2-(p-tolyl)ethyl)benzenesulfonamide (**3b**) (Daicel Chiralpak IC Column, *n*-Hexane: *i*-PrOH = 85:15, flow rate 1.0 mL/min, T = 25 °C, λ = 220 nm)

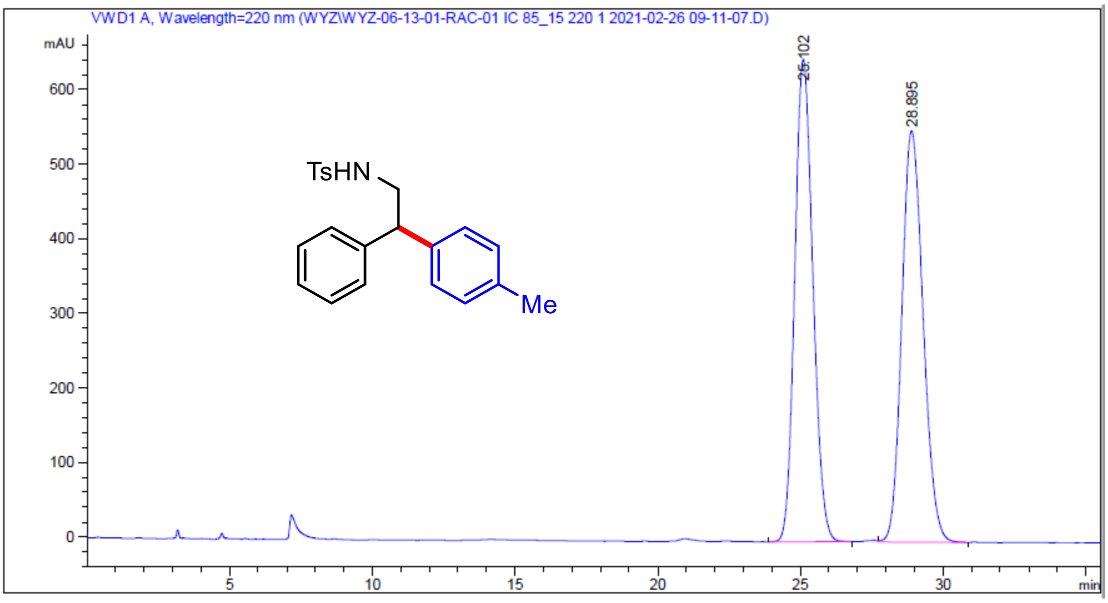

Peak NO	Ret. Time(min)	Area/%
1	25.102	49.9680
2	28.895	50.0320

Supplementary Figure 111. HPLC Chromatography of the (S)-4-methyl-N-(2-phenyl-2-(p-tolyl)ethyl)benzenesulfonamide (**3b**) (Daicel Chiralpak IC Column, *n*-Hexane: *i*-PrOH = 85:15, flow rate 1.0 mL/min, T = 25 °C, λ = 220 nm)

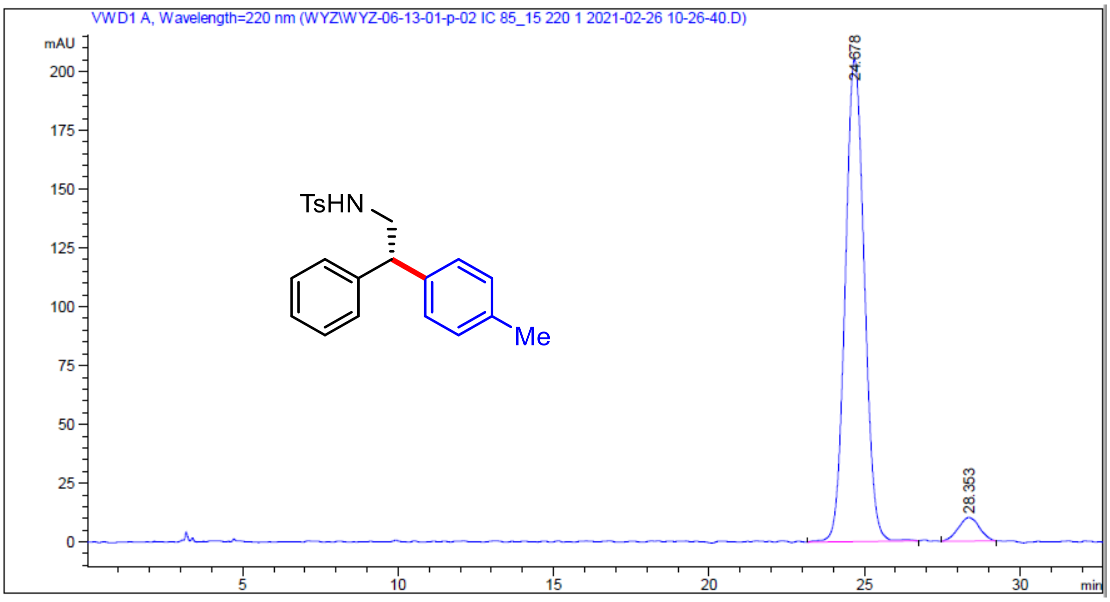

Peak NO	Ret. Time(min)	Area/%
1	24.678	94.8596
2	28.353	5.1404

Supplementary Figure 112. HPLC Chromatography of the Racemic N-(2-(4-isopropylphenyl)-2-phenylethyl)-4-methylbenzenesulfonamide (**3c**) (Daicel Chiralpak OD-H Column, *n*-Hexane: *i*-PrOH = 90:10, flow rate 1.0 mL/min, T = 25 °C, λ = 220 nm)

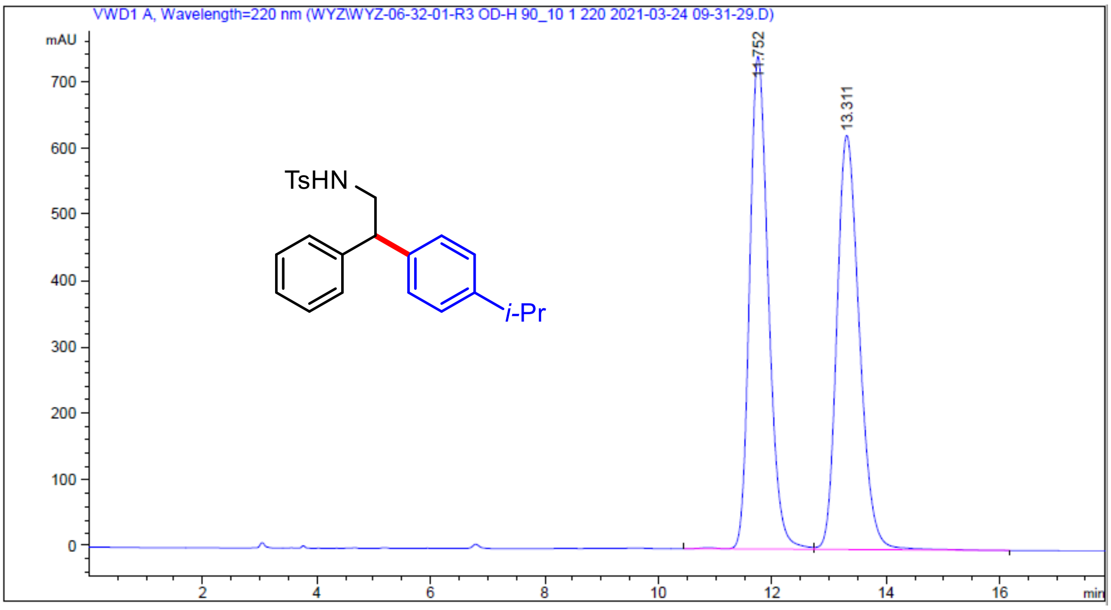

Peak NO	Ret. Time(min)	Area/%
1	11.752	49.8787
2	13.311	50.1213

Supplementary Figure 113. HPLC Chromatography of the (S)-N-(2-(4-isopropylphenyl)-2-phenylethyl)-4-methylbenzenesulfonamide (**3c**) (Daicel Chiralpak OD-H Column, *n*-Hexane: *i*-PrOH = 90:10, flow rate 1.0 mL/min, T = 25 °C, λ = 220 nm)

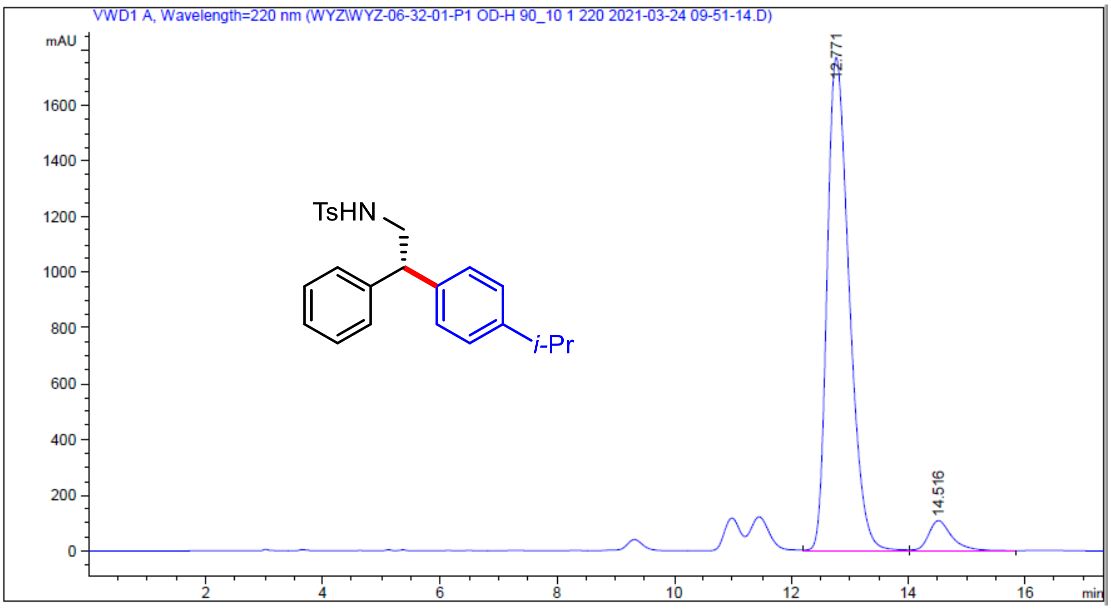

Peak NO	Ret. Time(min)	Area/%
1	12.771	94.0995
2	14.516	5.9005

Supplementary Figure 114. HPLC Chromatography of the Racemic N-(2-(4-(tert-butyl)phenyl)-2-phenylethyl)-4-methylbenzenesulfonamide (**3d**) (Daicel Chiralpak OD-H Column, *n*-Hexane: *i*-PrOH = 90:10, flow rate 1.0 mL/min, T = 25 °C, λ = 220 nm)

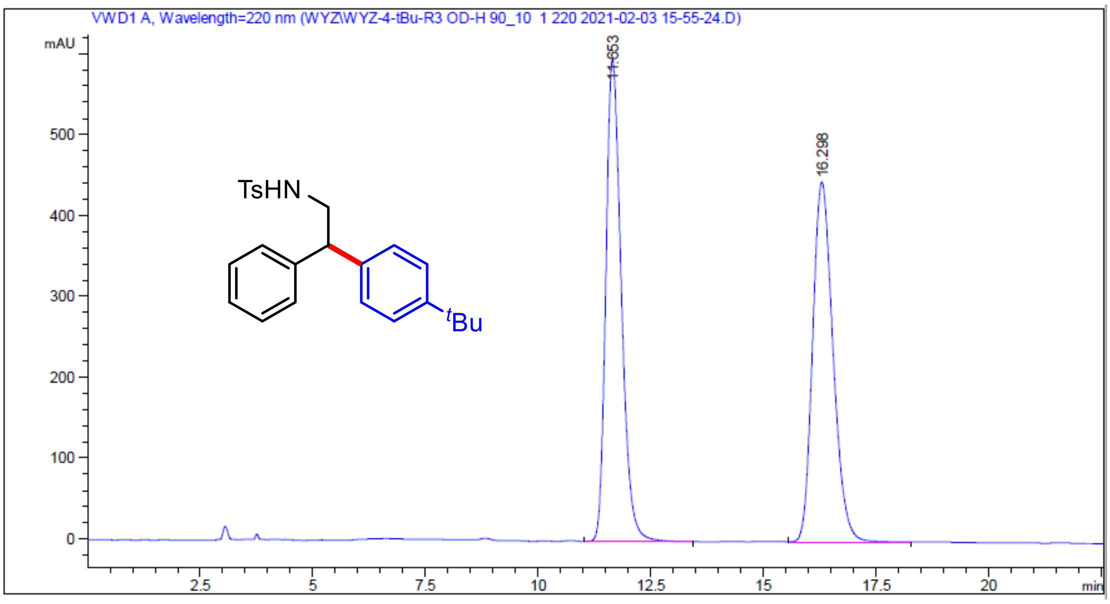

Peak NO	Ret. Time(min)	Area/%
1	11.653	50.4316
2	16.298	49.5684

Supplementary Figure 115. HPLC Chromatography of the (S)-N-(2-(4-(tert-butyl)phenyl)-2-phenylethyl)-4-methylbenzenesulfonamide (**3d**) (Daicel Chiralpak OD-H Column, *n*-Hexane: *i*-PrOH = 90:10, flow rate 1.0 mL/min, T = 25 °C, λ = 220 nm)

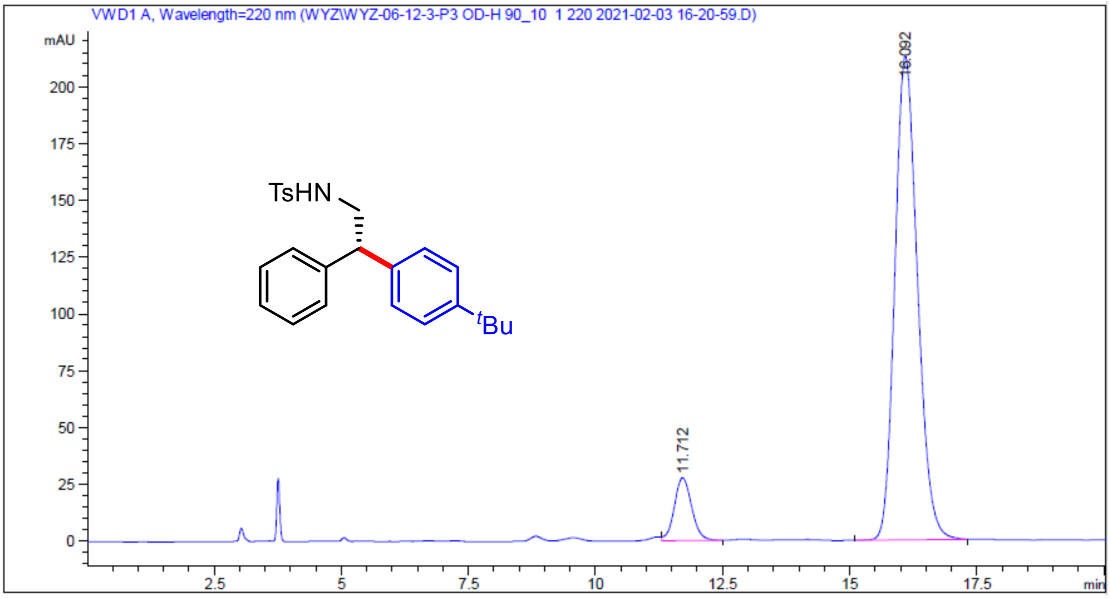

Peak NO	Ret. Time(min)	Area/%
1	11.712	9.0813
2	16.092	90.9187

Supplementary Figure 116. HPLC Chromatography of the Racemic 4-(2-((4-methylphenyl)sulfonamido)-1-phenylethyl)phenyl acetate (**3e**) (Daicel Chiralpak OD-H Column, *n*-Hexane: *i*-PrOH = 85:15, flow rate 1.0 mL/min, T = 25 °C, λ = 220 nm)

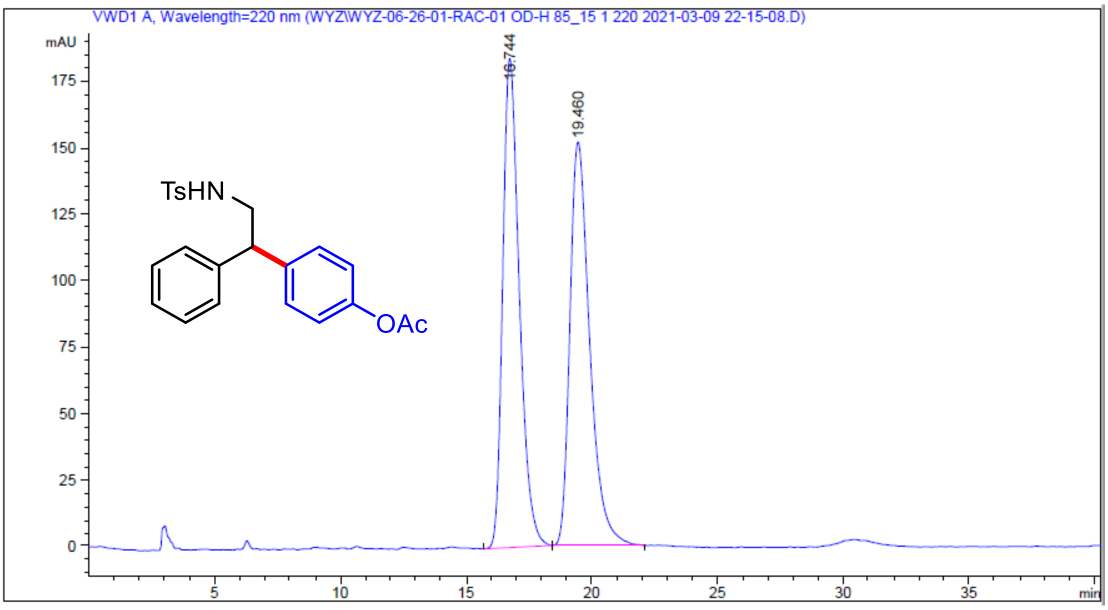

Peak NO	Ret. Time(min)	Area/%
1	16.744	50.3321
2	19.460	49.6679

Supplementary Figure 117. HPLC Chromatography of the (S)-4-(2-((4-methylphenyl)sulfonamido)-1-phenylethyl)phenyl acetate (**3e**) (Daicel Chiralpak OD-H Column, *n*-Hexane: *i*-PrOH = 85:15, flow rate 1.0 mL/min, T = 25 °C, λ = 220 nm)

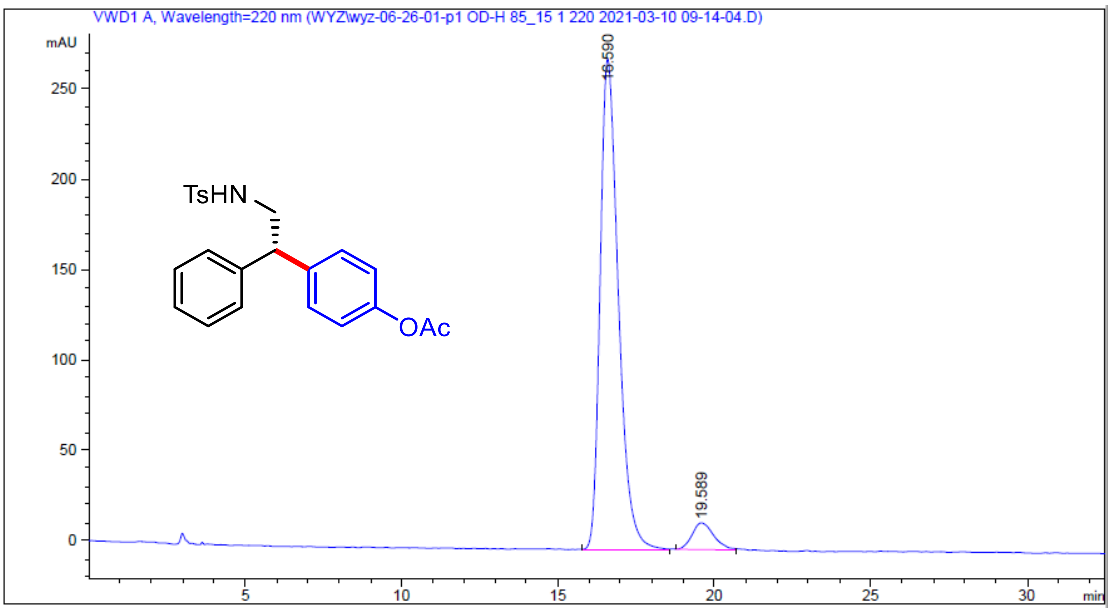

Peak NO	Ret. Time(min)	Area/%

1	16.590	93.8675
2	19.589	6.1325

Supplementary Figure 118. HPLC Chromatography of the Racemic 4-methyl-N-(2-phenyl-2-(4-(trifluoromethoxy)phenyl)ethyl)benzenesulfonamide (**3f**) (Daicel Chiralpak OJ-H Column, *n*-Hexane: *i*-PrOH = 85:15, flow rate 0.7 mL/min, T = 25 °C, λ = 214 nm)

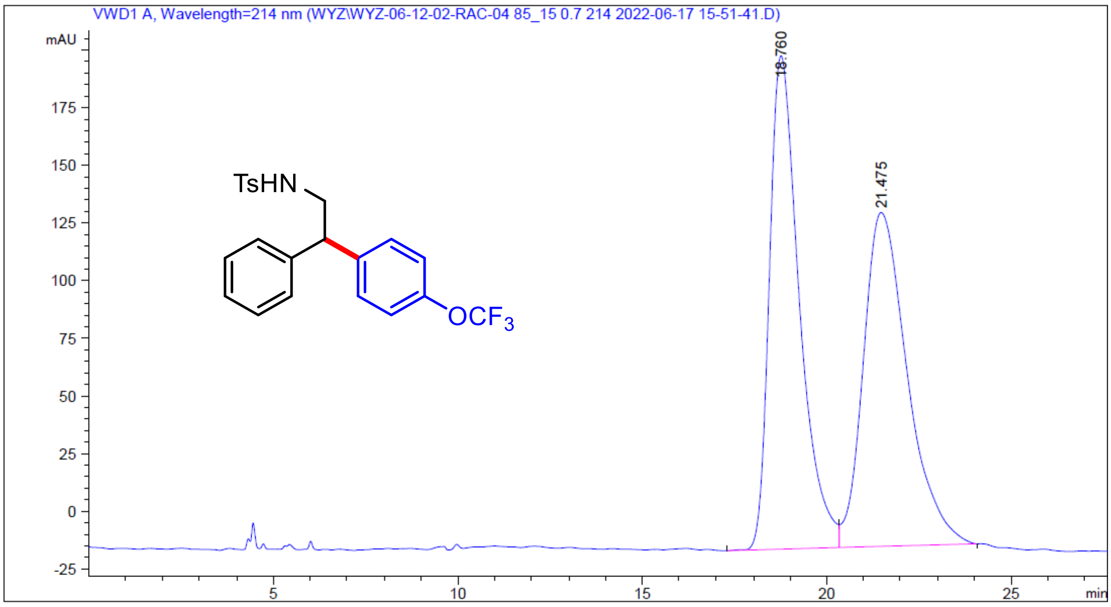

Peak NO	Ret. Time(min)	Area/%
1	18.760	50.0879
2	21.475	49.9121

Supplementary Figure 119. HPLC Chromatography of the (S)-4-methyl-N-(2-phenyl-2-(4-(trifluoromethoxy)phenyl)ethyl)benzenesulfonamide (**3f**) (Daicel Chiralpak OJ-H Column, *n*-Hexane: *i*-PrOH = 85:15, flow rate 0.7 mL/min, T = 25 °C, λ = 214 nm)

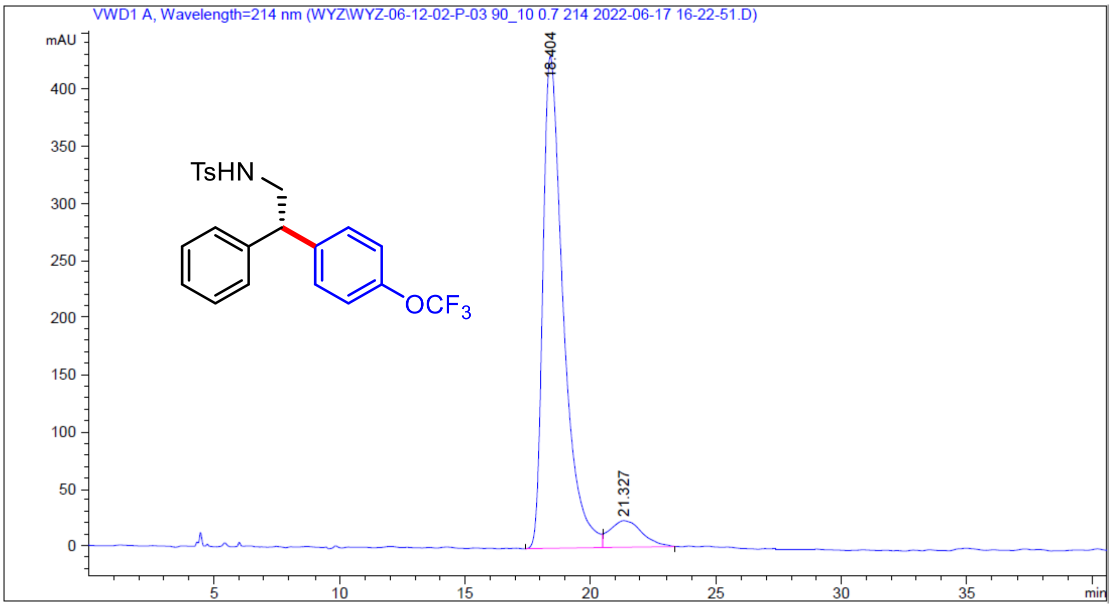

Peak NO	Ret. Time(min)	Area/%
1	18.404	92.0893
2	21.327	7.9107

Supplementary Figure 120. HPLC Chromatography of the Racemic N-(2-([1,1'-biphenyl]-4-yl)-2-phenylethyl)-4-methylbenzenesulfonamide (**3g**) (Daicel Chiralpak OD-H Column, *n*-Hexane: *i*-PrOH = 80:20, flow rate 1.0 mL/min, T = 25 °C, λ = 230 nm)

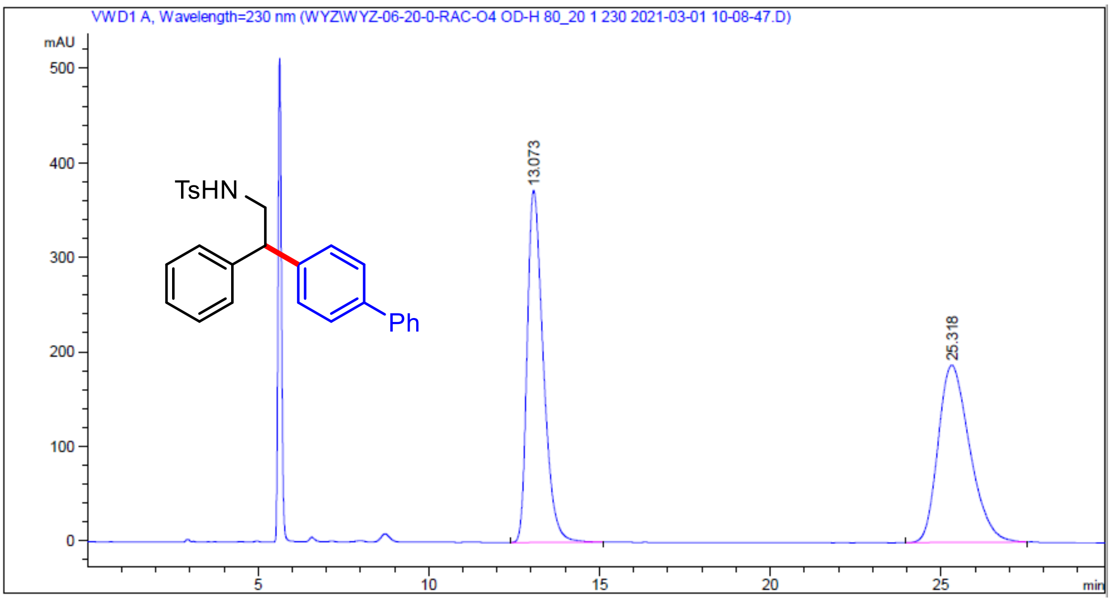

Peak NO	Ret. Time(min)	Area/%
1	13.073	50.1677
2	25.318	49.8323

Supplementary Figure 121. HPLC Chromatography of the (S)-N-(2-([1,1'-biphenyl]-4-yl)-2-phenylethyl)-4-methylbenzenesulfonamide (**3g**) (Daicel Chiralpak OD-H Column, *n*-Hexane: *i*-PrOH = 80:20, flow rate 1.0 mL/min, T = 25 °C, λ = 214 nm)

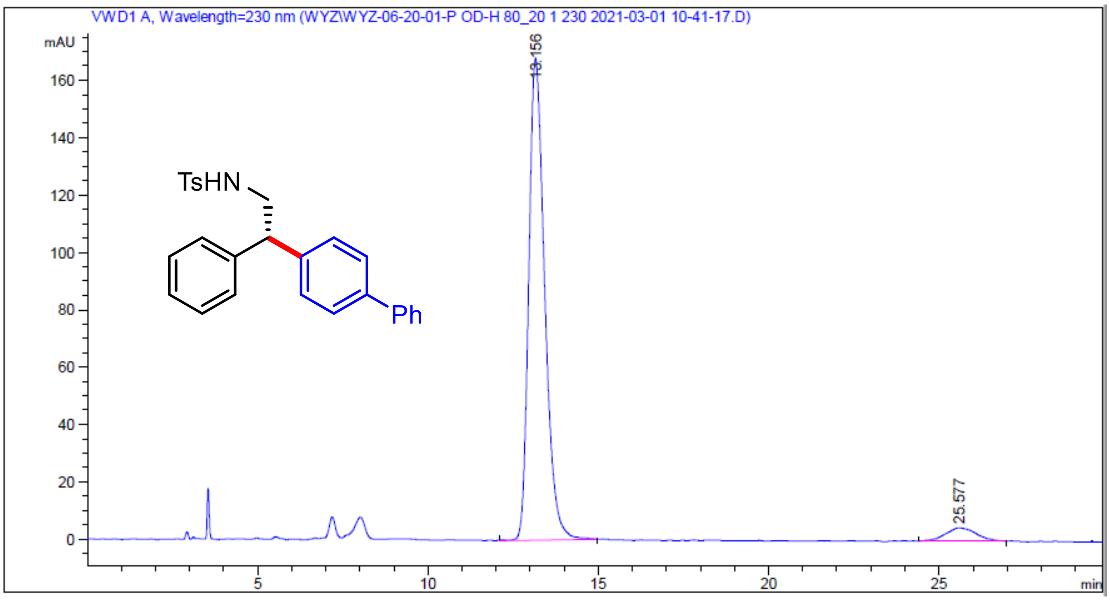

Peak NO	Ret. Time(min)	Area/%
1	13.156	94.8266
2	25.577	5.1734

Supplementary Figure 122. HPLC Chromatography of the Racemic 4-methyl-N-(2-phenyl-2-(4-(trifluoromethyl)phenyl)ethyl)benzenesulfonamide (**3h**) (Daicel Chiralpak OD-H Column, *n*-Hexane: *i*-PrOH = 90:10, flow rate 1.0 mL/min, T = 25 °C, λ = 220 nm)

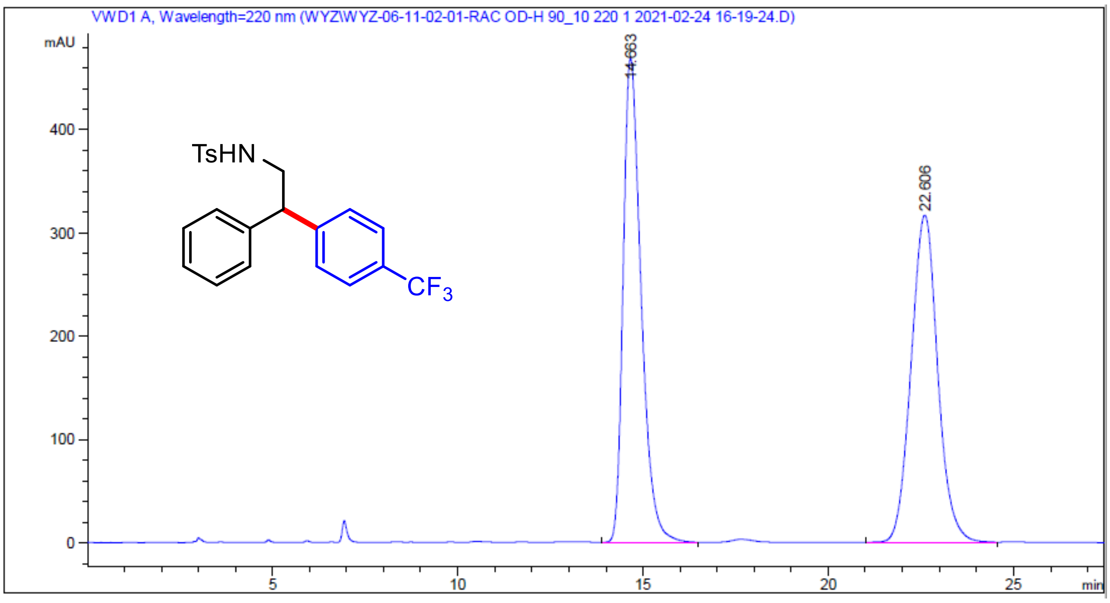

Peak NO	Ret. Time(min)	Area/%
1	14.663	49.8643
2	22.606	50.1357

Supplementary Figure 123. HPLC Chromatography of the (S)-4-methyl-N-(2-phenyl-2-(4-(trifluoromethyl)phenyl)ethyl)benzenesulfonamide (**3h**) (Daicel Chiralpak OD-H Column, *n*-Hexane: *i*-PrOH = 90:10, flow rate 1.0 mL/min, T = 25 °C, λ = 220 nm)

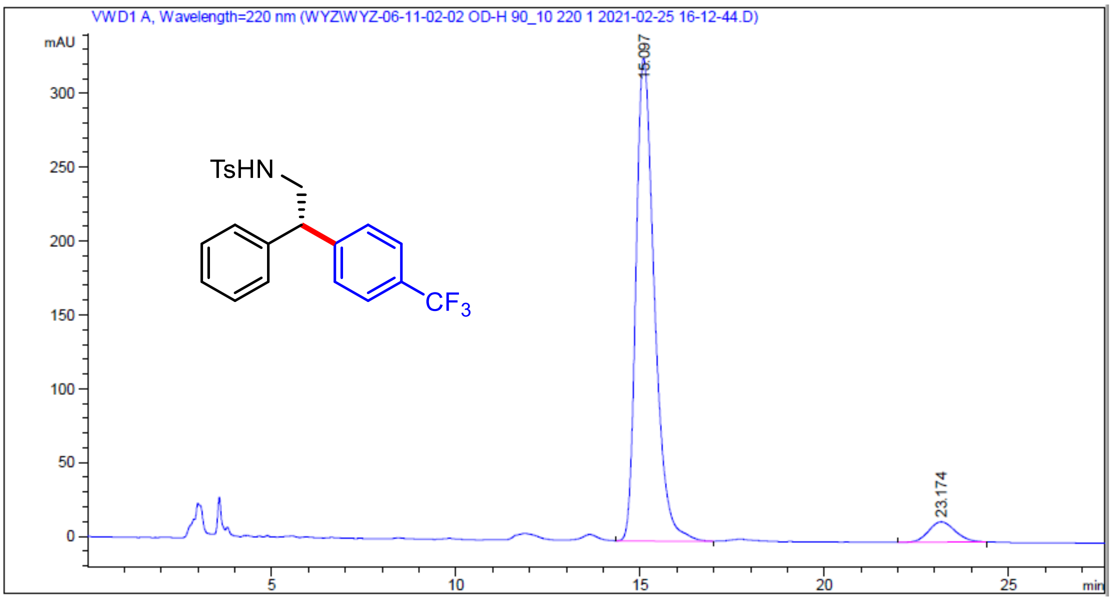

Peak NO	Ret. Time(min)	Area/%
1	15.097	94.1978
2	23.174	5.8022

Supplementary Figure 124. HPLC Chromatography of the Racemic N-(2-(4-fluorophenyl)-2-phenylethyl)-4-methylbenzenesulfonamide (**3i**) (Daicel Chiralpak OD-H Column, *n*-Hexane: *i*-PrOH = 90:10, flow rate 1.0 mL/min, T = 25 °C, λ = 220 nm)

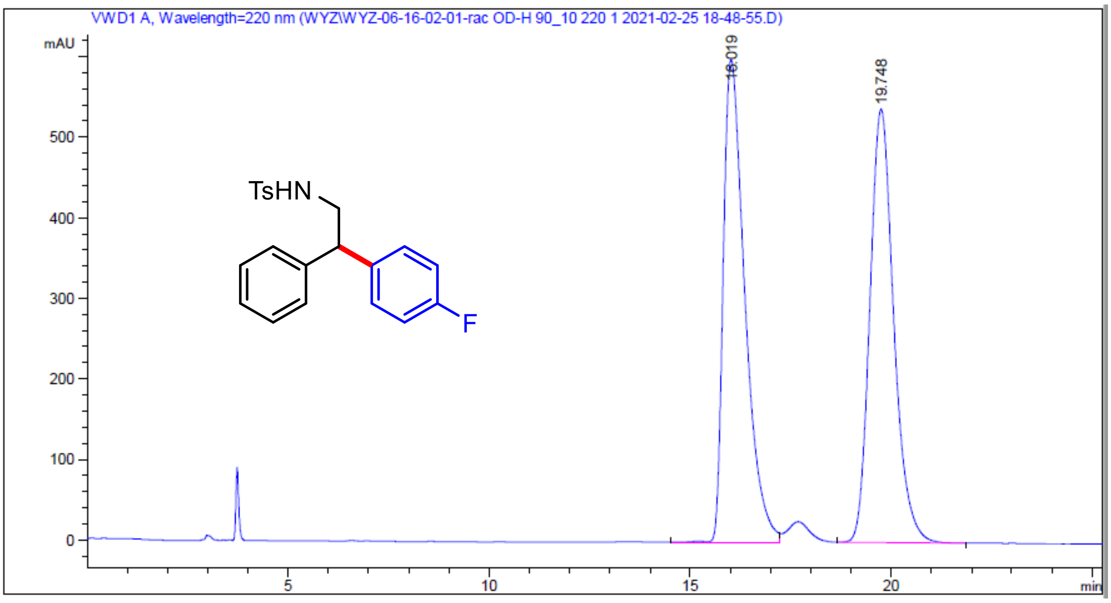

Peak NO	Ret. Time(min)	Area/%
1	16.019	50.1246
2	19.748	49.8754

Supplementary Figure 125. HPLC Chromatography of the (S)-N-(2-(4-fluorophenyl)-2-phenylethyl)-4-methylbenzenesulfonamide (**3i**) (Daicel Chiralpak OD-H Column, *n*-Hexane: *i*-PrOH = 90:10, flow rate 1.0 mL/min, T = 25 °C, λ = 220 nm)

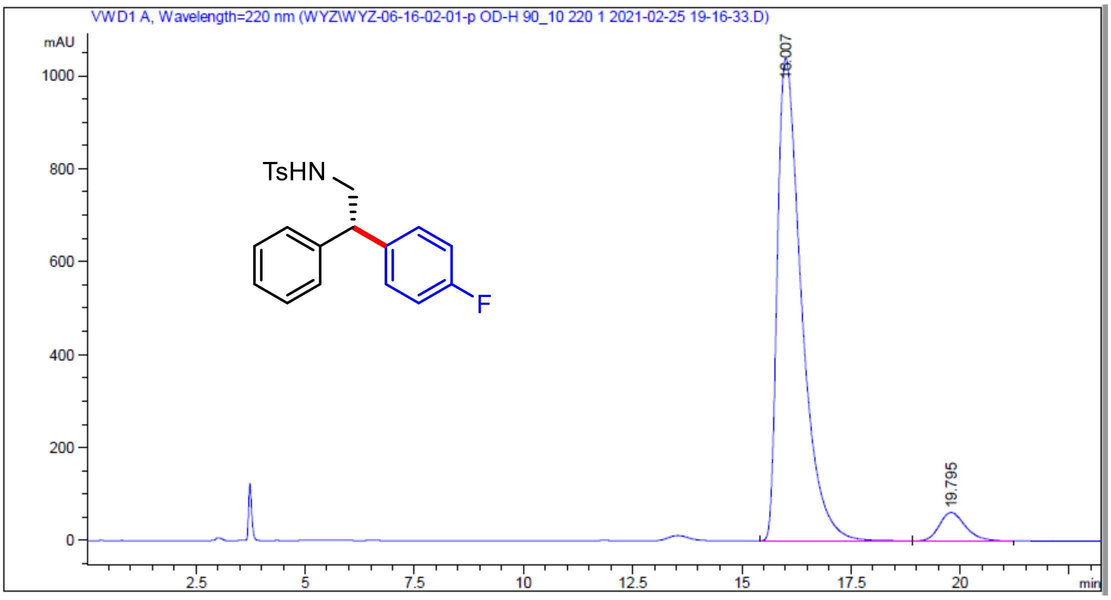

Peak NO	Ret. Time(min)	Area/%
1	15.097	94.0392
2	23.174	5.9608

Supplementary Figure 126. HPLC Chromatography of the Racemic N-(2-(4-chlorophenyl)-2-phenylethyl)-4-methylbenzenesulfonamide (**3j**) (Daicel Chiralpak IC Column, *n*-Hexane: *i*-PrOH = 85:15, flow rate 1.0 mL/min, T = 25 °C, λ = 220 nm)

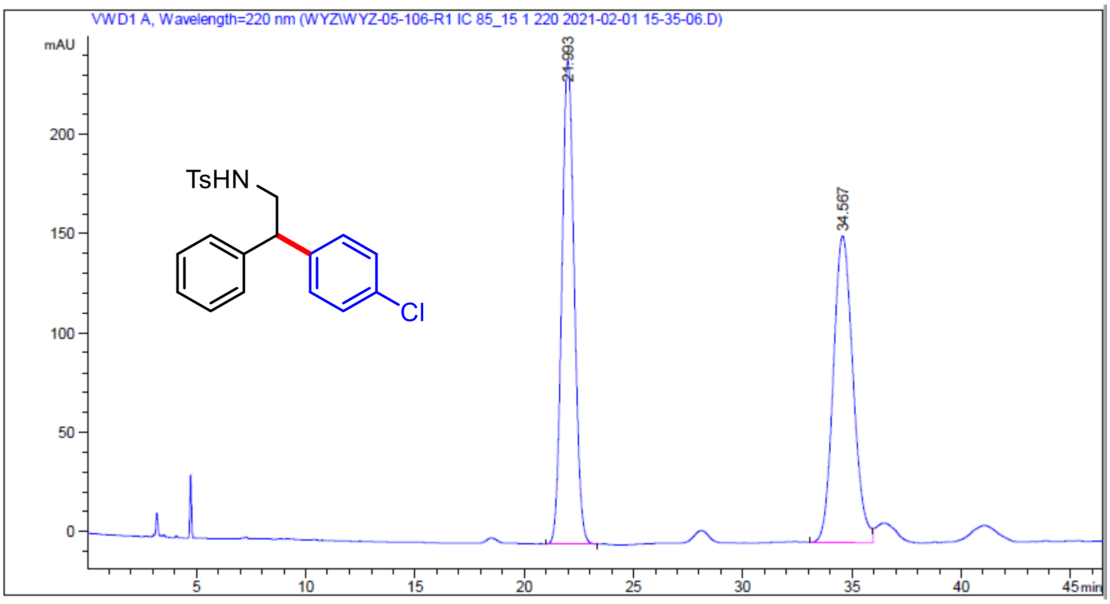

Peak NO	Ret. Time(min)	Area/%
1	21.993	49.6995
2	34.567	50.3005

Supplementary Figure 127. HPLC Chromatography of the (S)-N-(2-(4-chlorophenyl)-2-phenylethyl)-4-methylbenzenesulfonamide (**3j**) (Daicel Chiralpak IC Column, *n*-Hexane: *i*-PrOH = 85:15, flow rate 1.0 mL/min, T = 25 °C, λ = 220 nm)

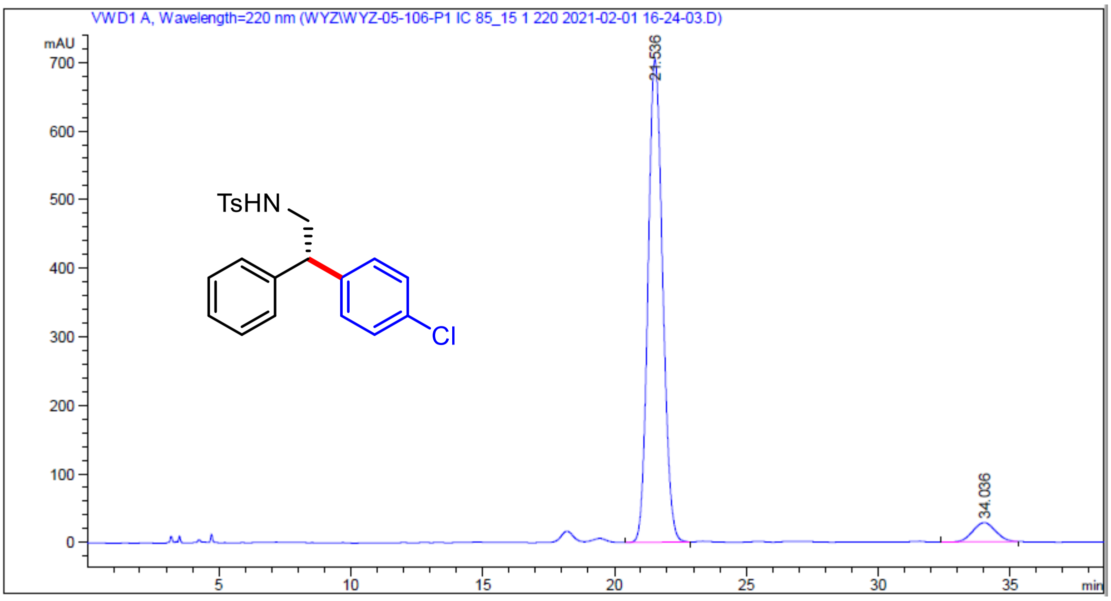

Peak NO	Ret. Time(min)	Area/%
1	21.536	94.1538
2	34.036	5.8462

Supplementary Figure 128. HPLC Chromatography of the Racemic (4-(2-((4-methylphenyl)sulfonamido)-1-phenylethyl)phenyl)carbamate (**3k**) (Daicel Chiralpak ID3 Column, *n*-Hexane: *i*-PrOH = 70:30, flow rate 0.7 mL/min, T = 25 °C, λ = 214 nm)

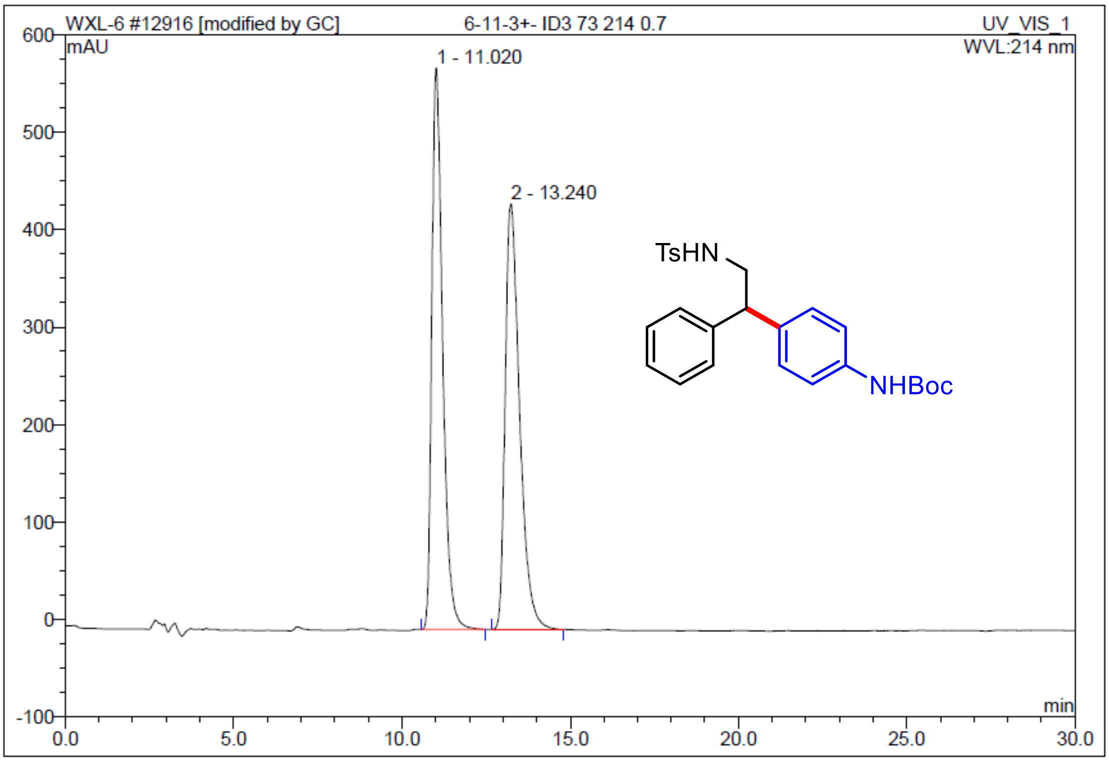

Peak NO	Ret. Time(min)	Area/%

1	11.02	49.80
2	13.24	50.20

Supplementary Figure 129. HPLC Chromatography of the (S)-(4-(2-((4-methylphenyl)sulfonamido)-1-phenylethyl)phenyl)carbamate (**3k**) (Daicel Chiralpak ID3 Column, *n*-Hexane: *i*-PrOH = 70:30, flow rate 0.7 mL/min, T = 25 °C, λ = 214 nm)

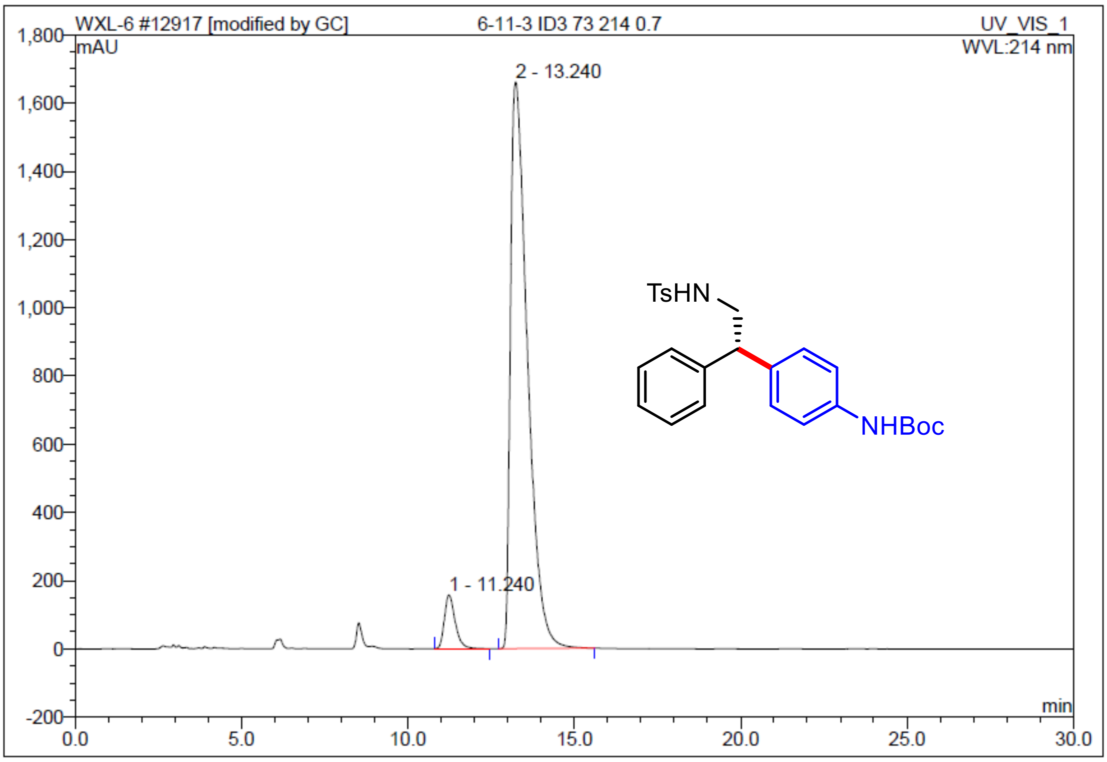

Peak NO	Ret. Time(min)	Area/%
1	11.24	5.67
2	13.24	94.33

Supplementary Figure 130. HPLC Chromatography of the Racemic 4-methyl-N-(2-phenyl-2-(m-tolyl)ethyl)benzenesulfonamide (**3l**) (Daicel Chiralpak AD-H Column, *n*-Hexane: *i*-PrOH = 95:05, flow rate 1.0 mL/min, T = 25 °C, λ = 220 nm)

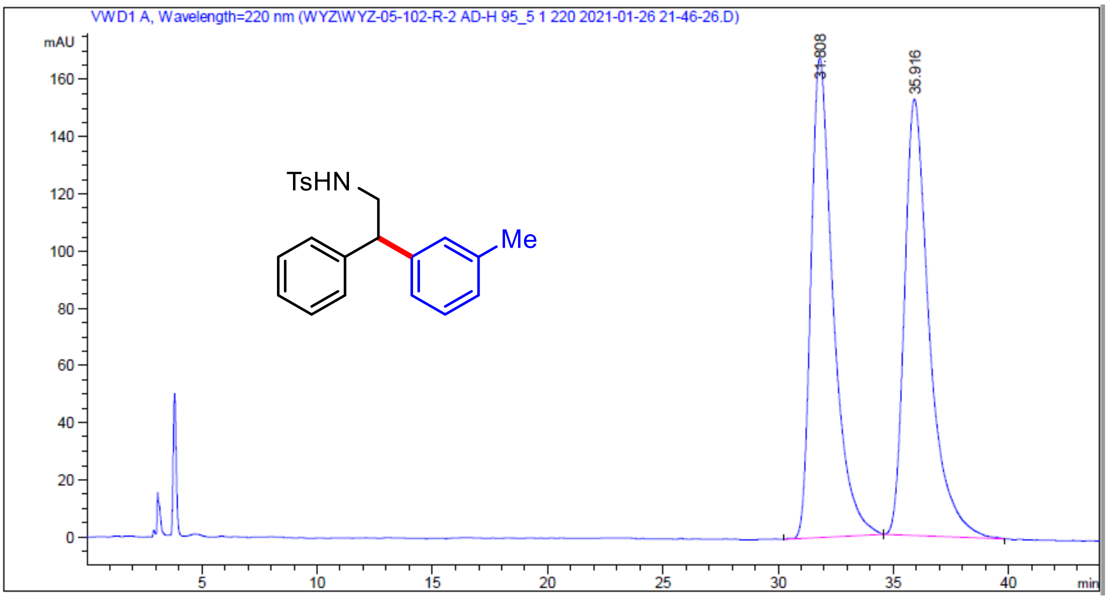

Peak NO	Ret. Time(min)	Area/%
1	31.808	49.5613
2	35.916	50.4387

Supplementary Figure 131. HPLC Chromatography of the (S)-4-methyl-N-(2-phenyl-2-(m-tolyl)ethyl)benzenesulfonamide (**3l**) (Daicel Chiralpak AD-H Column, *n*-Hexane: *i*-PrOH = 95:05, flow rate 1.0 mL/min, T = 25 °C, λ = 220 nm)

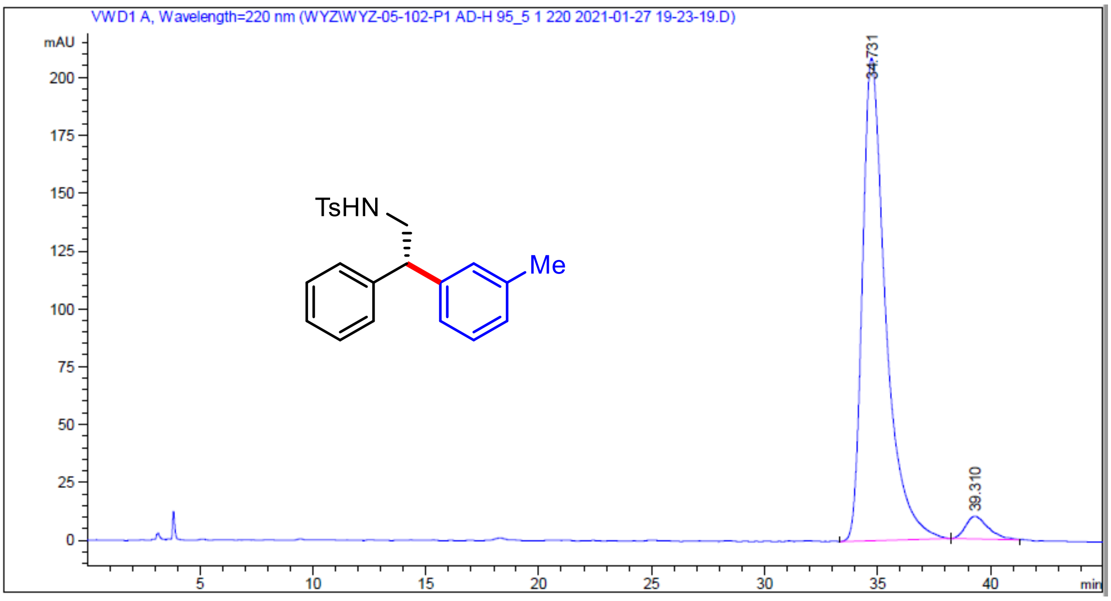

Peak NO	Ret. Time(min)	Area/%
1	34.731	95.6286
2	39.310	4.3714

Supplementary Figure 132. HPLC Chromatography of the Racemic N-(2-([1,1'-biphenyl]-3-yl)-2-phenylethyl)-4-methylbenzenesulfonamide (**3m**) (Daicel Chiralpak OD-H Column, *n*-Hexane: *i*-PrOH = 80:20, flow rate 1.0 mL/min, T = 25 °C, λ = 230 nm)

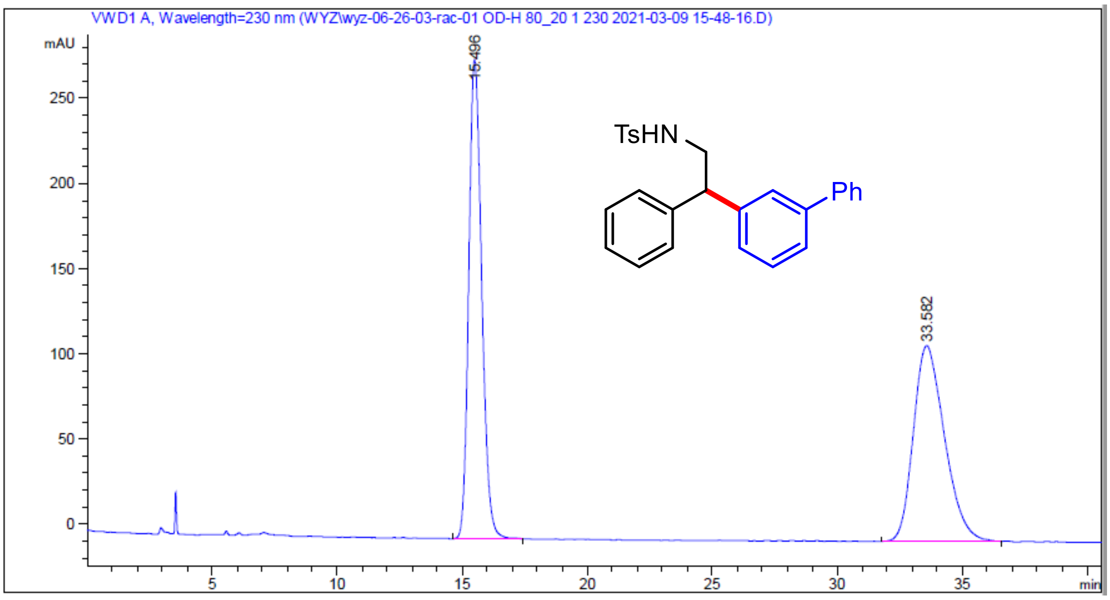

Peak NO	Ret. Time(min)	Area/%
1	15.496	50.3919
2	33.582	49.6081

Supplementary Figure 133. HPLC Chromatography of the (S)-N-(2-([1,1'-biphenyl]-3-yl)-2-phenylethyl)-4-methylbenzenesulfonamide (**3m**) (Daicel Chiralpak OD-H Column, *n*-Hexane: *i*-PrOH = 80:20, flow rate 1.0 mL/min, T = 25 °C, λ = 230 nm)

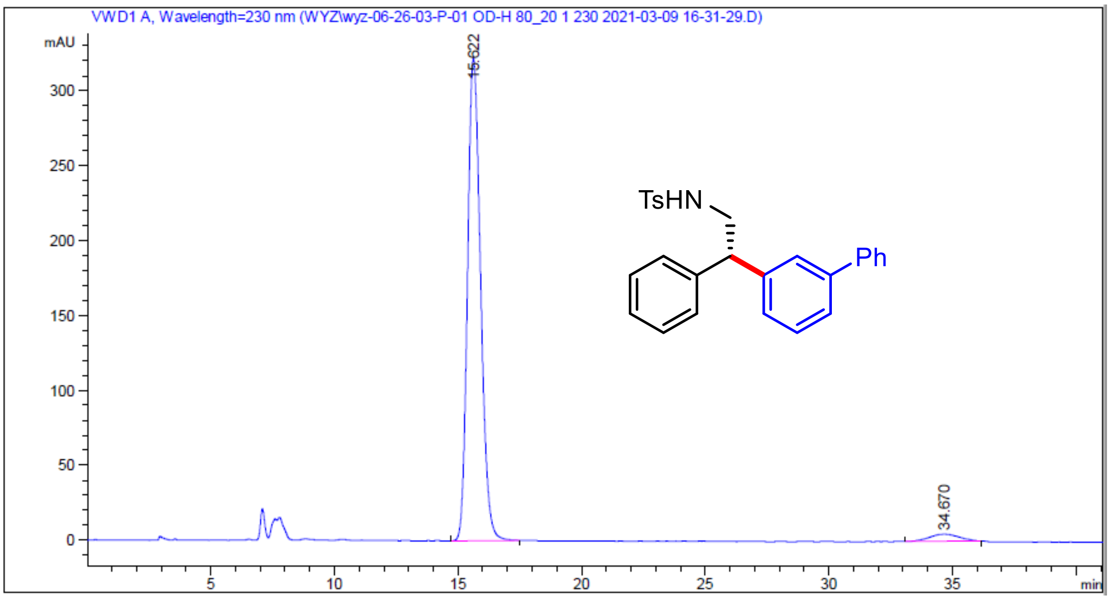

Peak NO	Ret. Time(min)	Area/%
1	15.622	96.7293
2	34.670	3.2707

Supplementary Figure 134. HPLC Chromatography of the Racemic methyl-3-(2-((4-methylphenyl)sulfonamido)-1-phenylethyl)benzoate (**3n**) (Daicel Chiralpak AD-H Column, *n*-Hexane: *i*-PrOH = 80:20, flow rate 1.0 mL/min, T = 25 °C, λ = 220 nm)

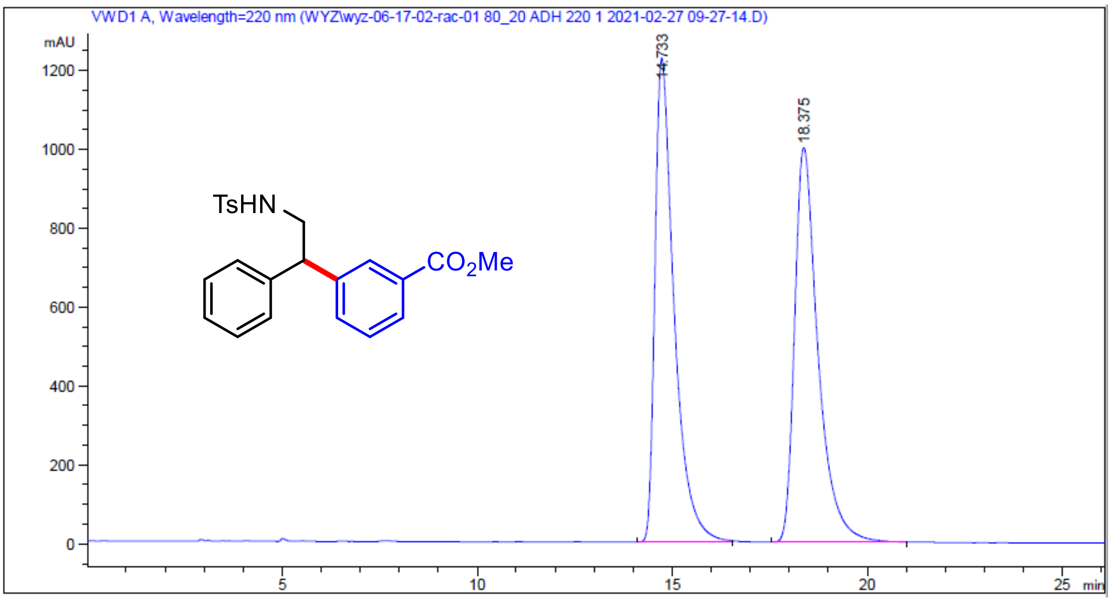

Peak NO	Ret. Time(min)	Area/%
1	14.733	50.1537
2	18.375	49.8463

Supplementary Figure 135. HPLC Chromatography of the methyl (S)-3-(2-((4-methylphenyl)sulfonamido)-1-phenylethyl)benzoate (**3n**) (Daicel Chiralpak AD-H Column, *n*-Hexane: *i*-PrOH = 80:20, flow rate 1.0 mL/min, T = 25 °C, λ = 220 nm)

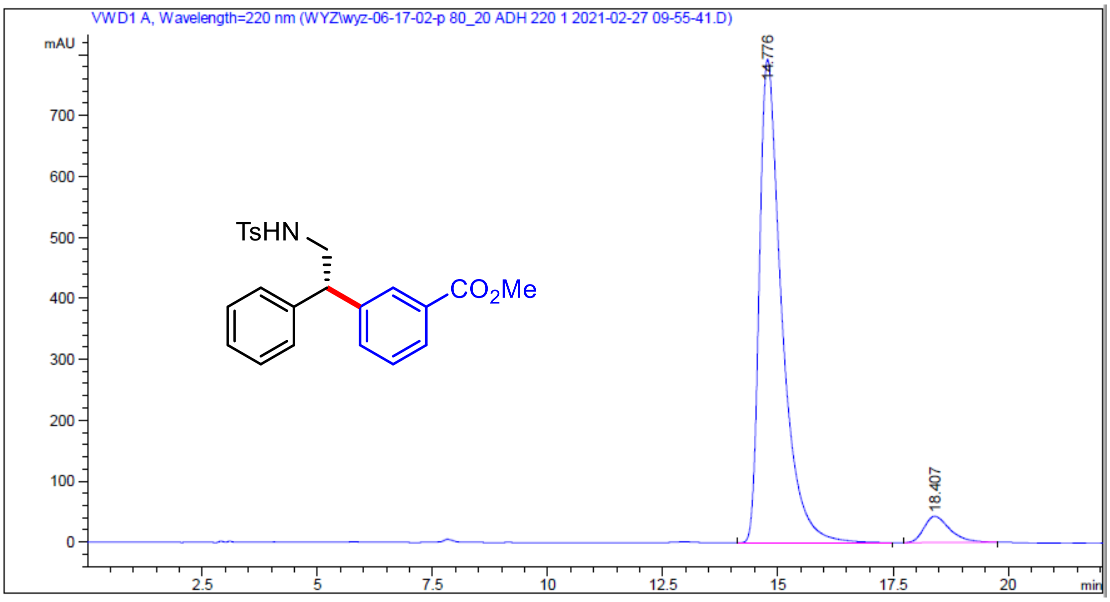

Peak NO	Ret. Time(min)	Area/%
1	14.776	94.0663
2	18.407	5.9337

Supplementary Figure 136. HPLC Chromatography of the Racemic ethyl-3-(2-((4-methylphenyl)sulfonamido)-1-phenylethyl)benzoate (**3o**) (Daicel Chiralpak AD-H Column, *n*-Hexane: *i*-PrOH = 85:15, flow rate 1.0 mL/min, T = 25 °C, λ = 214 nm)

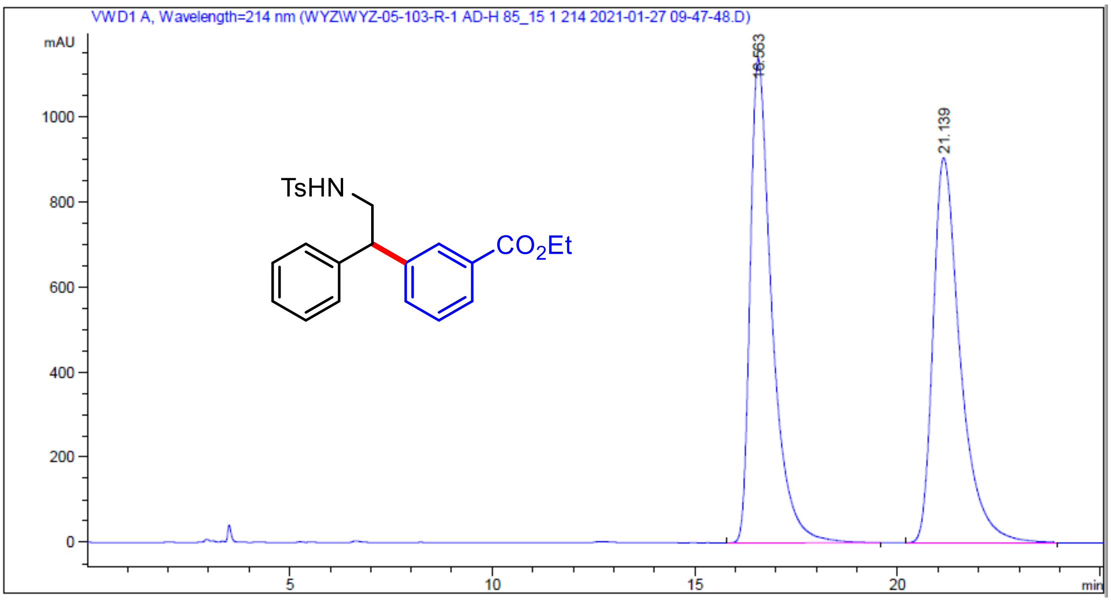

Peak NO	Ret. Time(min)	Area/%
1	16.563	50.1555
2	21.139	49.8445

Supplementary Figure 137. HPLC Chromatography of the ethyl (S)-3-(2-((4-methylphenyl)sulfonamido)-1-phenylethyl)benzoate (**3o**) (Daicel Chiralpak AD-H Column, *n*-Hexane: *i*-PrOH = 85:15, flow rate 1.0 mL/min, T = 25 °C, λ = 214 nm)

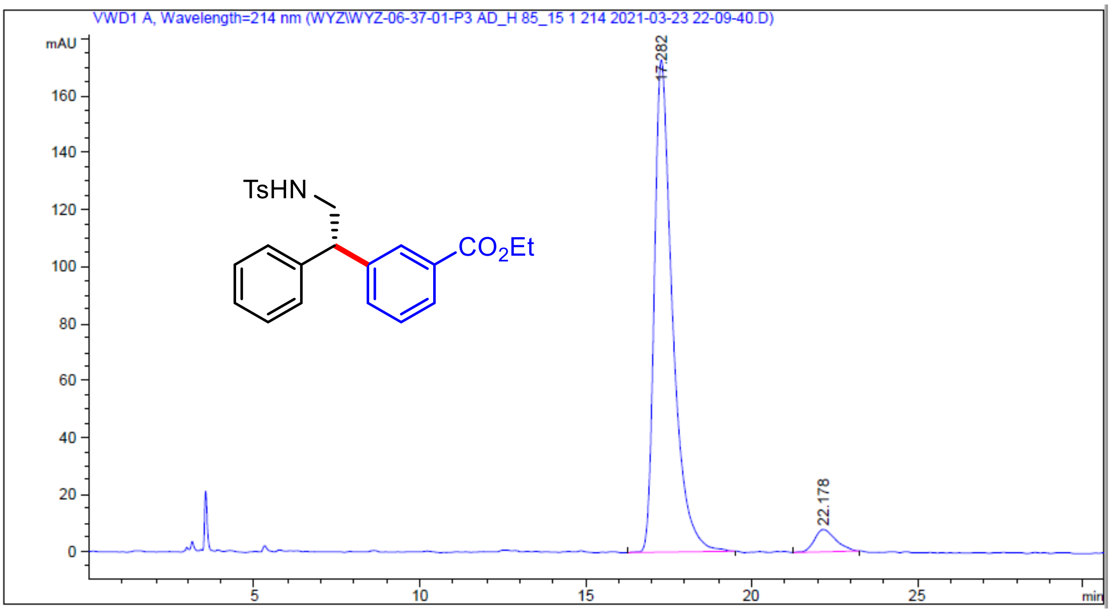

Peak NO	Ret. Time(min)	Area/%
1	17.282	94.9149
2	22.178	5.0851

Supplementary Figure 138. HPLC Chromatography of the Racemic 4-methyl-N-(2-phenyl-2-(3-(trifluoromethyl)phenyl)ethyl)benzenesulfonamide (**3p**) (Daicel Chiralpak AD-H Column, *n*-Hexane: *i*-PrOH = 90:10, flow rate 1.0 mL/min, T = 25 °C, λ = 214 nm)

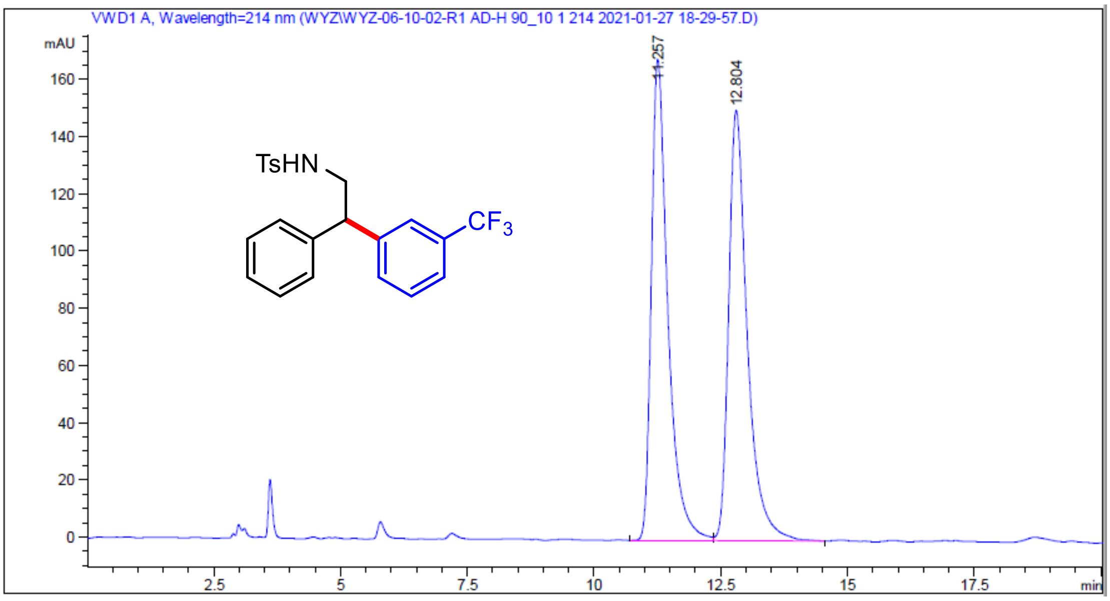

Peak NO	Ret. Time(min)	Area/%
1	11.257	49.8041
2	12.804	50.1959

Supplementary Figure 139. HPLC Chromatography of the (S)-4-methyl-N-(2-phenyl-2-(3-(trifluoromethyl)phenyl)ethyl)benzenesulfonamide (**3p**) (Daicel Chiralpak AD-H Column, *n*-Hexane: *i*-PrOH = 90:10, flow rate 1.0 mL/min, T = 25 °C, λ = 214 nm)

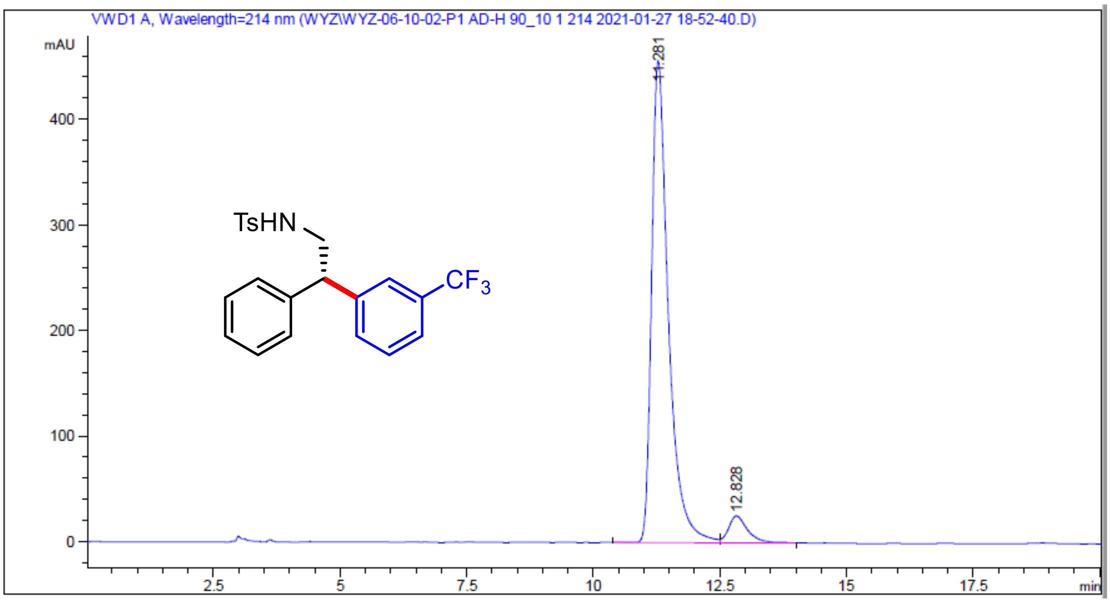

Peak NO	Ret. Time(min)	Area/%
1	11.255	94.8688
2	12.823	5.1312

Supplementary Figure 140. HPLC Chromatography of the Racemic N-(2-(3-fluorophenyl)-2-phenylethyl)-4-methylbenzenesulfonamide (**3q**) (Daicel Chiralpak OD-H Column, *n*-Hexane: *i*-PrOH = 90:10, flow rate 1.0 mL/min, T = 25 °C, λ = 220 nm)

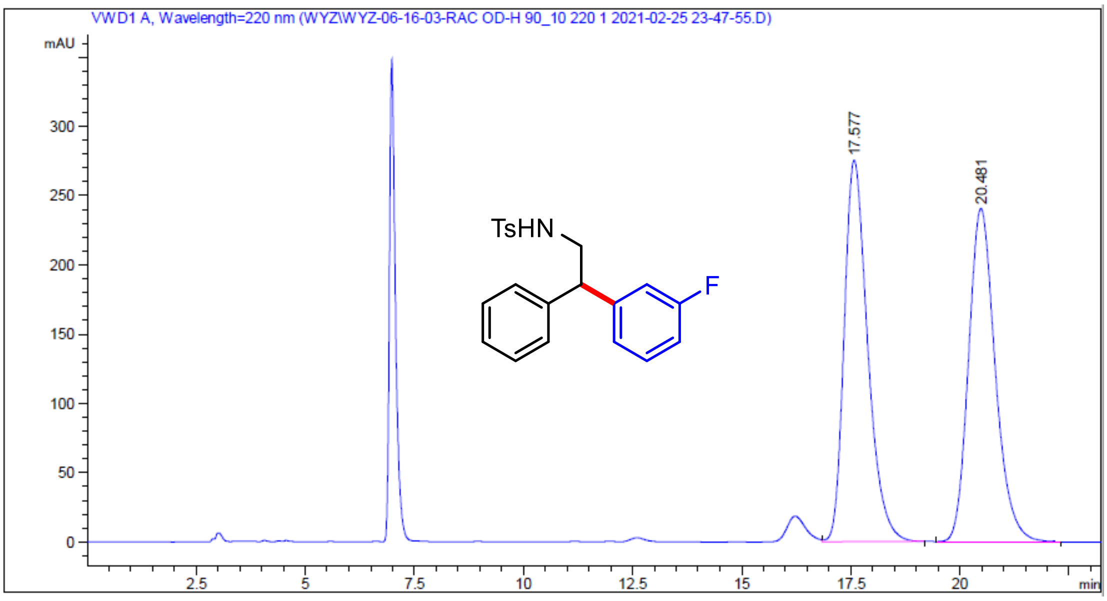

Peak NO	Ret. Time(min)	Area/%
1	17.577	50.1300
2	20.481	49.8700

Supplementary Figure 141. HPLC Chromatography of the (S)-N-(2-(3-fluorophenyl)-2-phenylethyl)-4-methylbenzenesulfonamide (**3q**) (Daicel Chiralpak OD-H Column, *n*-Hexane: *i*-PrOH = 90:10, flow rate 1.0 mL/min, T = 25 °C, λ = 220 nm)

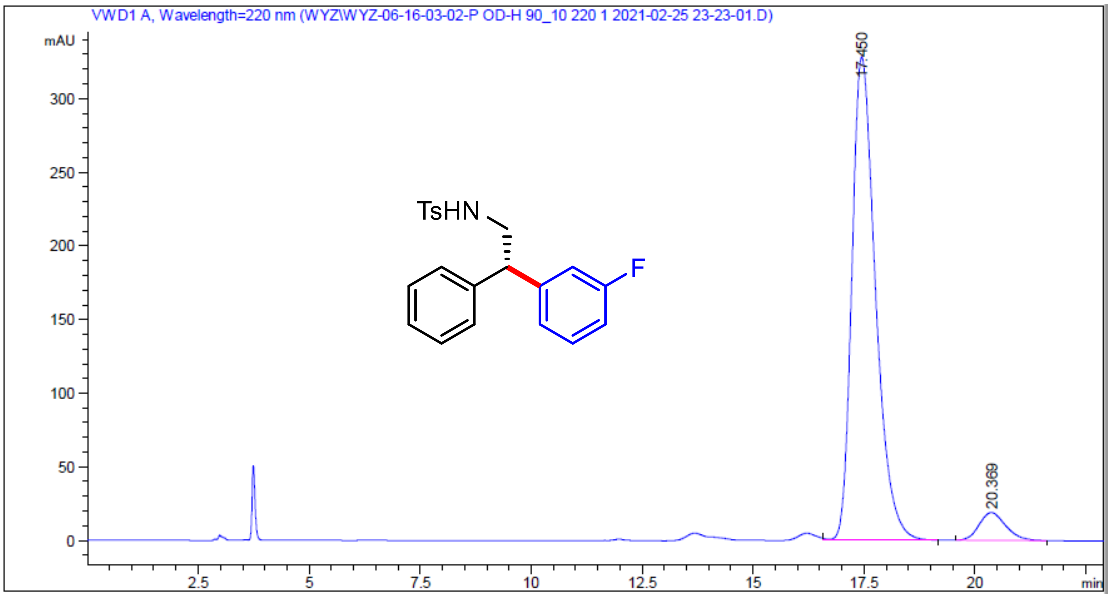

Peak NO	Ret. Time(min)	Area/%
1	17.450	93.8935
2	20.369	6.1065

Supplementary Figure 142. HPLC Chromatography of the Racemic N-(2-(3-chlorophenyl)-2-phenylethyl)-4-methylbenzenesulfonamide (**3r**) (Daicel Chiralpak IC Column, *n*-Hexane: *i*-PrOH = 85:15, flow rate 1.0 mL/min, T = 25 °C, λ = 220 nm)

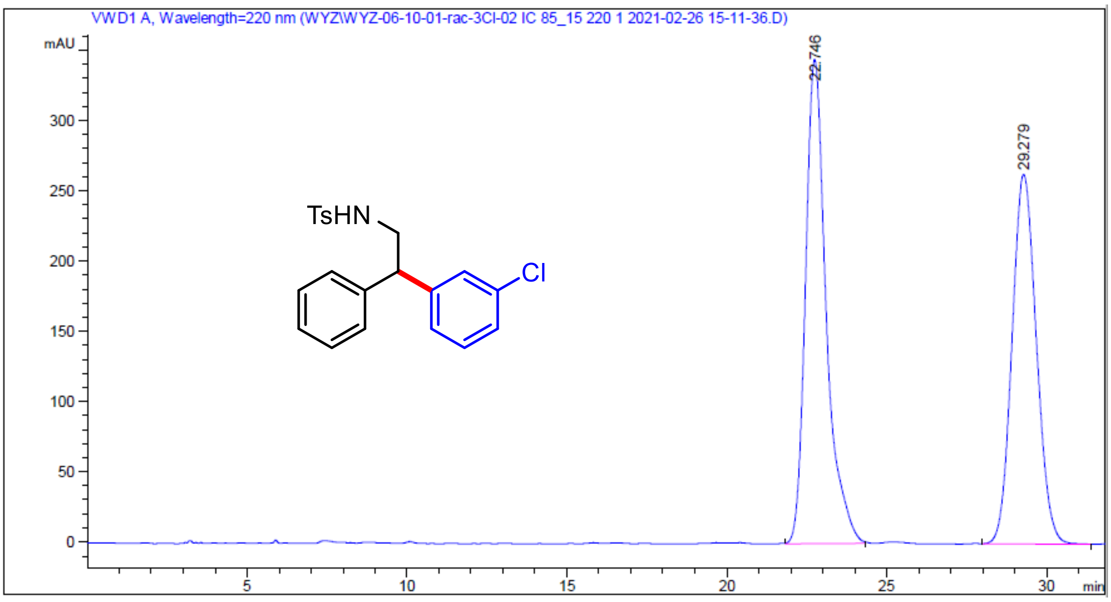

Peak NO	Ret. Time(min)	Area/%
1	22.746	52.7529
2	29.279	47.2471

Supplementary Figure 143. HPLC Chromatography of the (S)-N-(2-(3-chlorophenyl)-2-phenylethyl)-4-methylbenzenesulfonamide (**3r**) (Daicel Chiralpak IC Column, *n*-Hexane: *i*-PrOH = 85:15, flow rate 1.0 mL/min, T = 25 °C, λ = 220 nm)

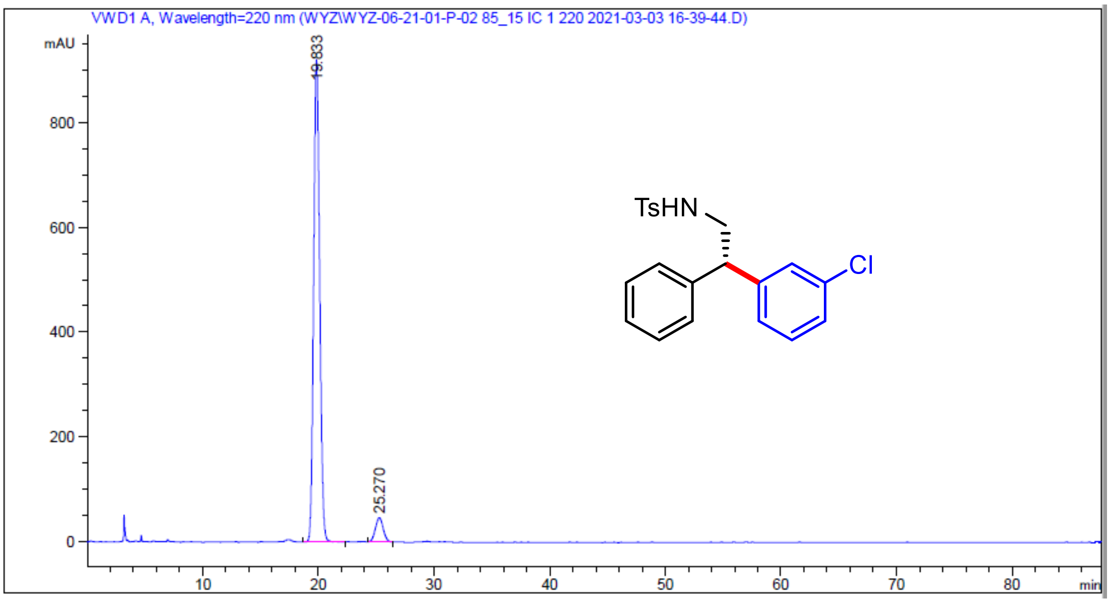

Peak NO	Ret. Time(min)	Area/%

1	19.833	93.8699
2	25.270	6.1301

Supplementary Figure 144. HPLC Chromatography of the Racemic N-(2-(3-methoxyphenyl)-2-phenylethyl)-4-methylbenzenesulfonamide (**3s**) (Daicel Chiralpak IC Column, *n*-Hexane: *i*-PrOH = 85:15, flow rate 1.0 mL/min, T = 25 °C, λ = 220 nm)

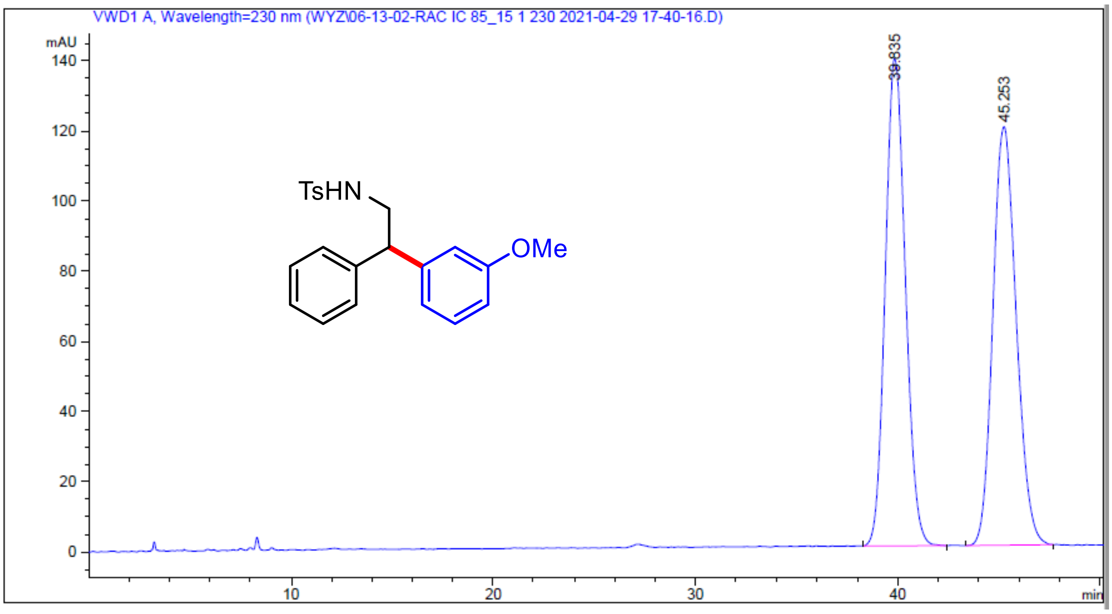

Peak NO	Ret. Time(min)	Area/%
1	39.835	50.0406
2	45.253	49.9594

Supplementary Figure 145. HPLC Chromatography of the (S)-N-(2-(3-methoxyphenyl)-2-phenylethyl)-4-methylbenzenesulfonamide (**3s**) (Daicel Chiralpak IC Column, *n*-Hexane: *i*-PrOH = 85:15, flow rate 1.0 mL/min, T = 25 °C, λ = 220 nm)

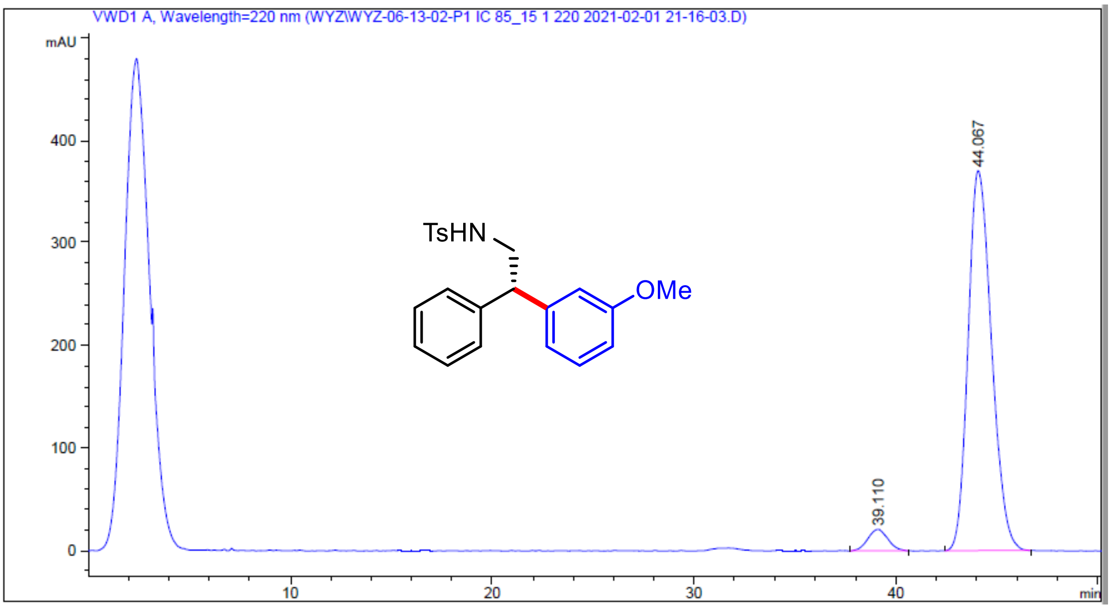

Peak NO	Ret. Time(min)	Area/%
1	39.110	4.5831
2	44.067	95.4169

Supplementary Figure 146. HPLC Chromatography of the Racemic 3-(2-((4-methylphenyl)sulfonamido)-1-phenylethyl)phenyl acetate (**3t**) (Daicel Chiralpak AD-H Column, *n*-Hexane: *i*-PrOH = 85:15, flow rate 1.0 mL/min, T = 25 °C, λ = 220 nm)

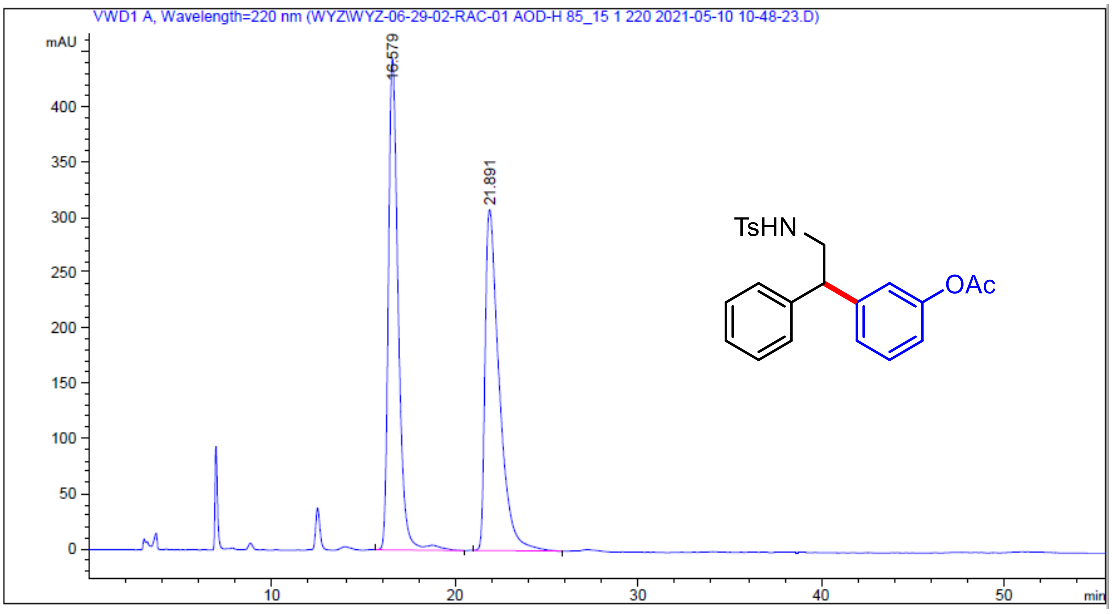

Peak NO	Ret. Time(min)	Area/%
1	16.579	50.1892
2	21.891	49.8108

Supplementary Figure 147. HPLC Chromatography of the (S)-3-(2-((4-methylphenyl)sulfonamido)-1-phenylethyl)phenyl acetate (**3t**) (Daicel Chiralpak AD-H Column, *n*-Hexane: *i*-PrOH = 85:15, flow rate 1.0 mL/min, T = 25 °C, λ = 220 nm)

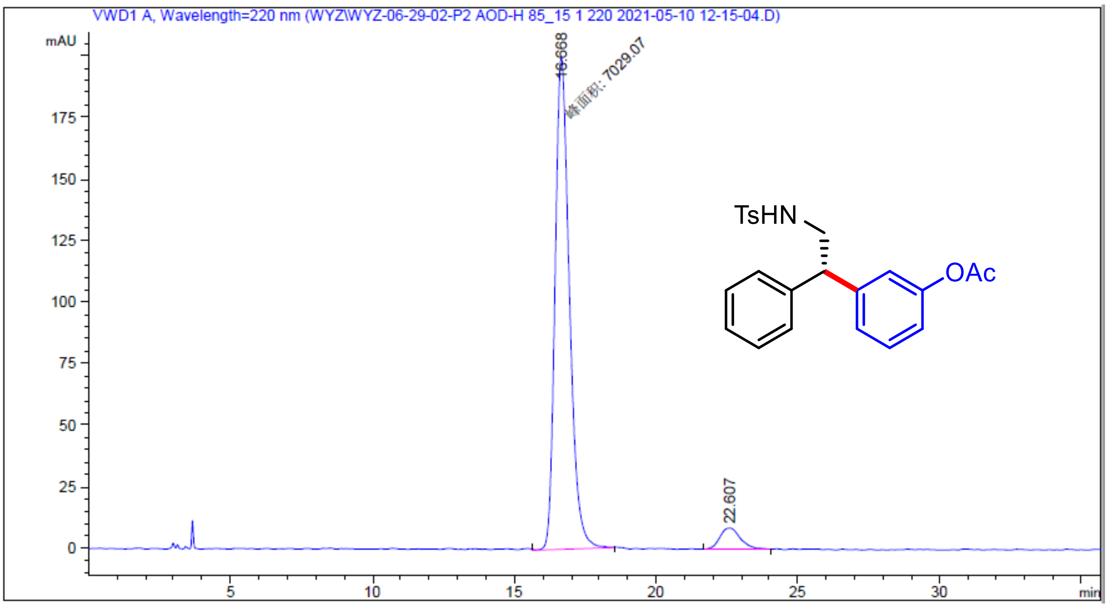

Peak NO	Ret. Time(min)	Area/%
1	16.668	94.2332
2	22.607	5.7668

Supplementary Figure 148. HPLC Chromatography of the Racemic Tert-butyl-(3-(2-((4-methylphenyl)sulfonamido)-1-phenylethyl)phenyl)carbamate (**3u**) (Daicel Chiralpak AD-H Column, *n*-Hexane: *i*-PrOH = 80:20, flow rate 1.0 mL/min, T = 25 °C, λ = 230 nm)

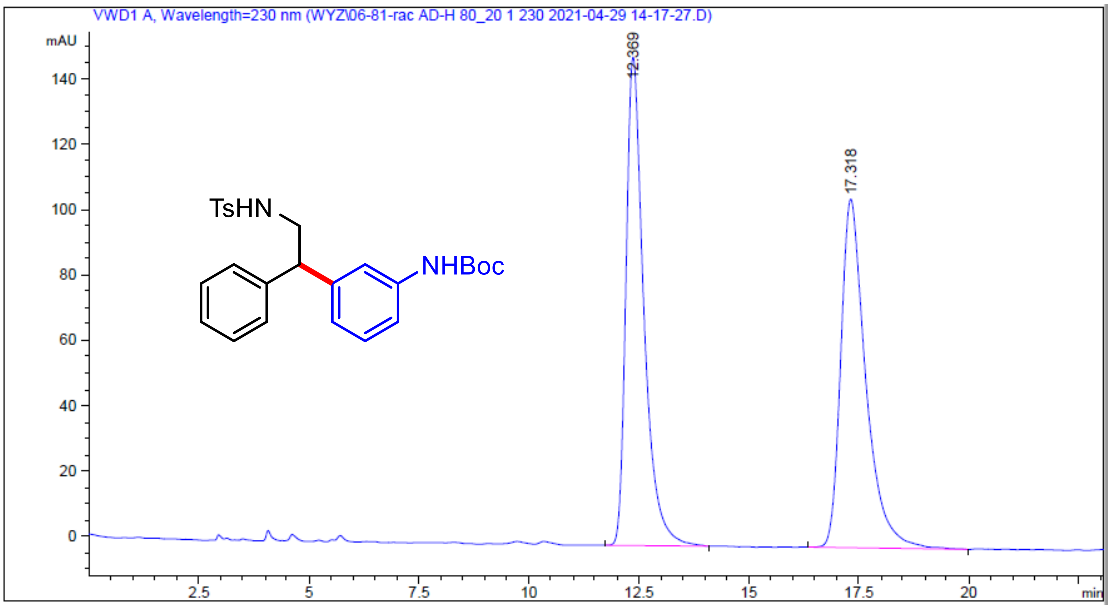

Peak NO	Ret. Time(min)	Area/%
1	12.369	49.9907
2	17.318	50.0093

Supplementary Figure 149. HPLC Chromatography of the Tert-butyl (S)-(3-(2-((4-methylphenyl)sulfonamido)-1-phenylethyl)phenyl)carbamate (**3u**) (Daicel Chiralpak AD-H Column, *n*-Hexane: *i*-PrOH = 80:20, flow rate 1.0 mL/min, T = 25 °C, λ = 230 nm)

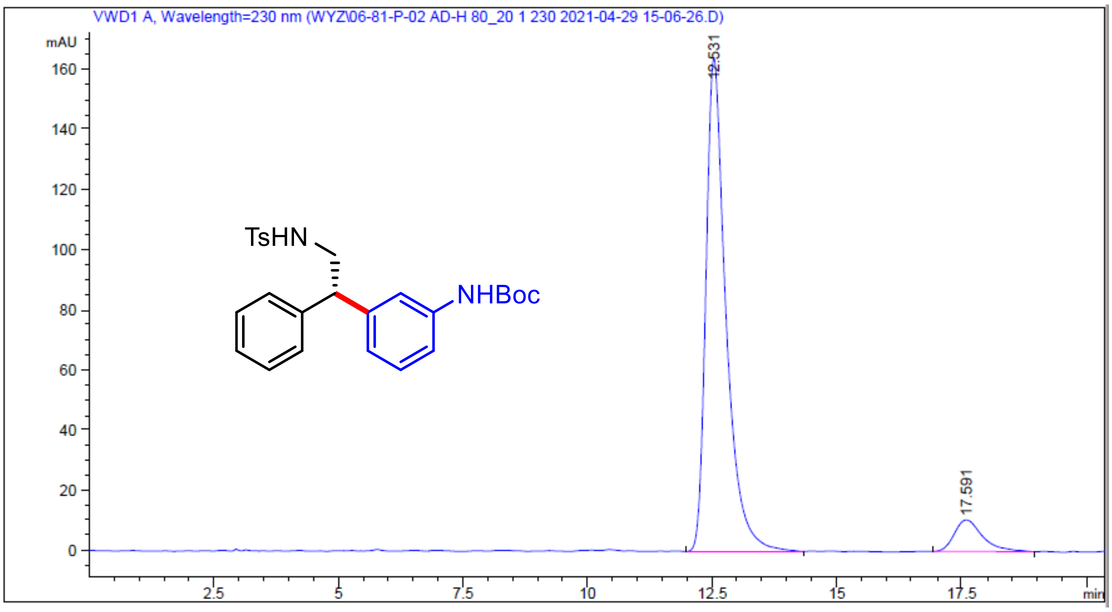

Peak NO	Ret. Time(min)	Area/%
1	12.531	91.7242
2	17.591	8.2758

Supplementary Figure 150. HPLC Chromatography of the Racemic N-(2-(3,4-dimethoxyphenyl)-2-phenylethyl)-4-methylbenzenesulfonamide (**3v**) (Daicel Chiralpak OD-H Column, *n*-Hexane: *i*-PrOH = 85:15, flow rate 1.0 mL/min, T = 25 °C, λ = 220 nm)

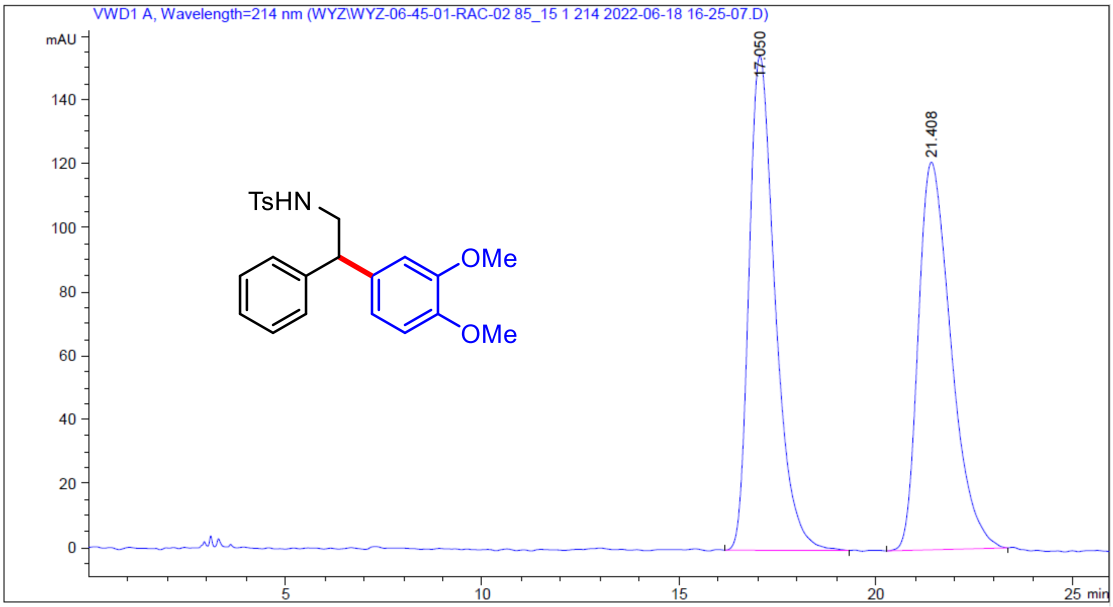

Peak NO	Ret. Time(min)	Area/%
1	17.050	50.4501
2	21.408	49.5499

Supplementary Figure 151. HPLC Chromatography of the (S)-N-(2-(3,4-dimethoxyphenyl)-2-phenylethyl)-4-methylbenzenesulfonamide (**3v**) (Daicel Chiralpak OD-H Column, *n*-Hexane: *i*-PrOH = 85:15, flow rate 1.0 mL/min, T = 25 °C, λ = 220 nm)

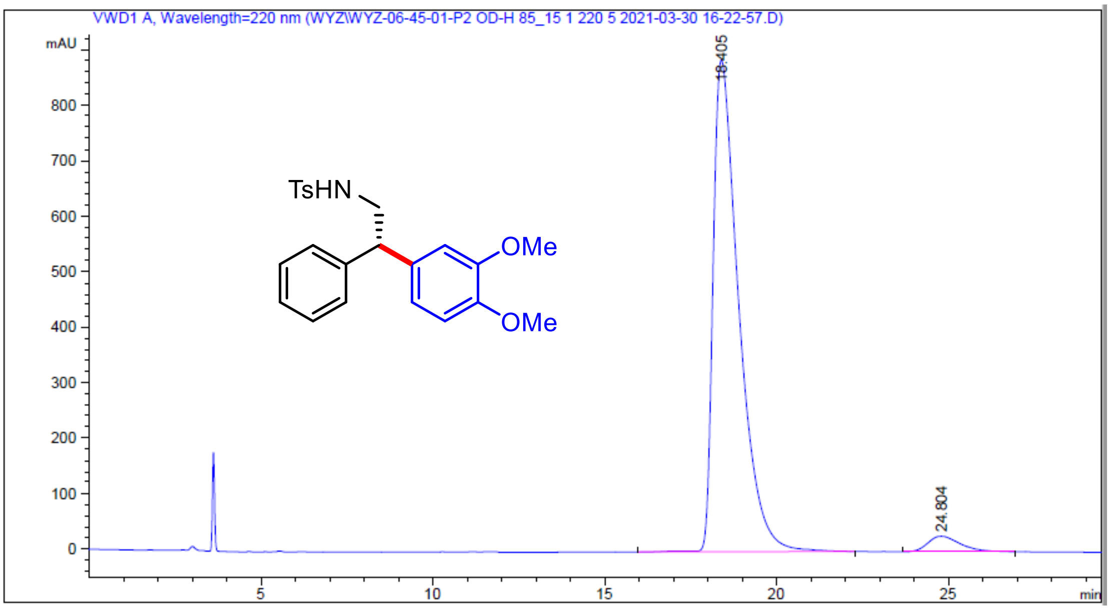

Peak NO	Ret. Time(min)	Area/%
1	18.405	96.1527
2	24.804	3.8473

Supplementary Figure 152. HPLC Chromatography of the Racemic 4-methyl-N-(2-phenyl-2-(3,4,5-trimethoxyphenyl)ethyl)benzenesulfonamide (**3w**) (Daicel Chiralpak OD-H Column, *n*-Hexane: *i*-PrOH = 85:15, flow rate 1.0 mL/min, T = 25 °C, λ = 220 nm)

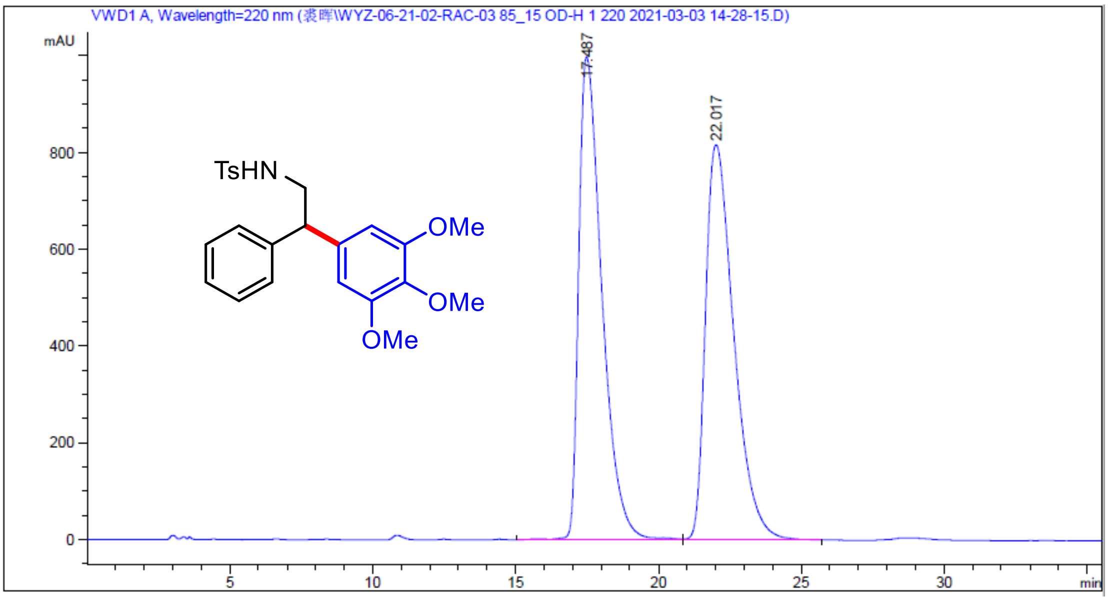

Peak NO	Ret. Time(min)	Area/%
1	17.487	50.0040
2	22.017	49.9960

Supplementary Figure 153. HPLC Chromatography of the (S)-4-methyl-N-(2-phenyl-2-(3,4,5-trimethoxyphenyl)ethyl)benzenesulfonamide (**3w**) (Daicel Chiralpak OD-H Column, *n*-Hexane: *i*-PrOH = 85:15, flow rate 1.0 mL/min, T = 25 °C, λ = 220 nm)

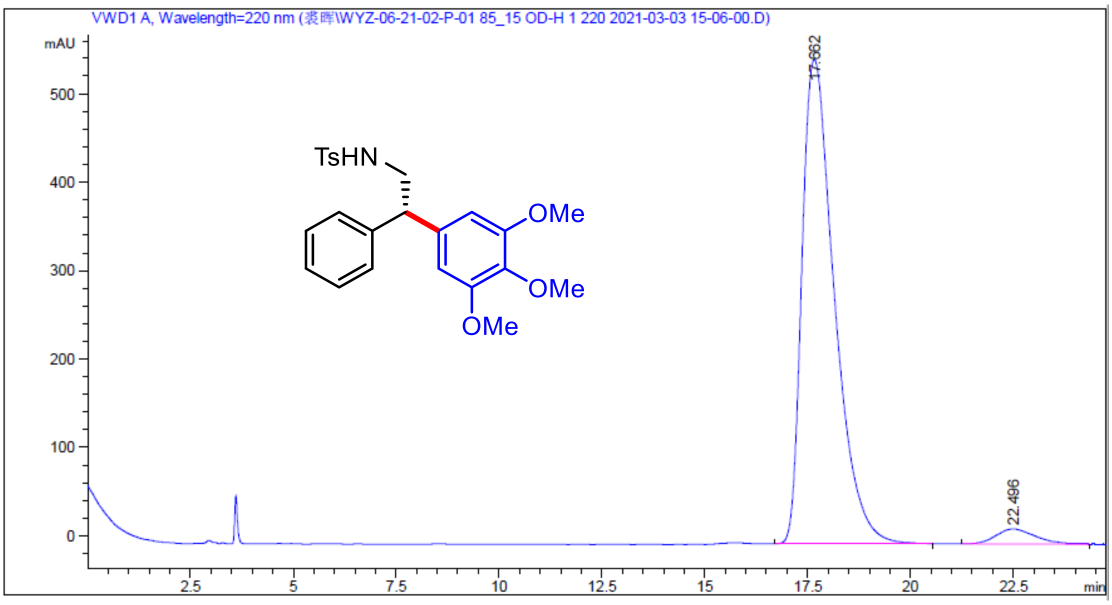

Peak NO	Ret. Time(min)	Area/%
1	17.662	96.4319
2	22.496	3.5681

Supplementary Figure 154. HPLC Chromatography of the Racemic 4-Methyl-N-(2-(naphthalen-2-yl)-2-phenylethyl)benzenesulfonamide (**3x**) (Daicel Chiralpak IC Column, *n*-Hexane: *i*-PrOH = 85:15, flow rate 1.0 mL/min, T = 25 °C, λ = 220 nm)

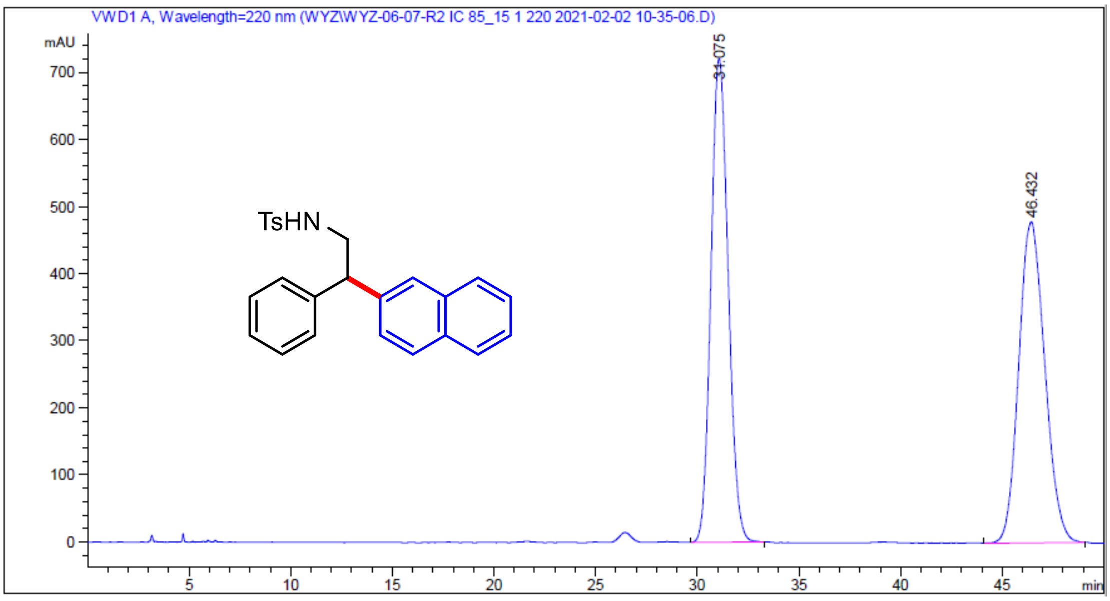

Peak NO	Ret. Time(min)	Area/%
1	31.075	49.9483
2	46.432	50.0517

Supplementary Figure 155. HPLC Chromatography of the (S)-4-Methyl-N-(2-(naphthalen-2-yl)-2-phenylethyl)benzenesulfonamide (**3x**) (Daicel Chiralpak IC Column, *n*-Hexane: *i*-PrOH = 85:15, flow rate 1.0 mL/min, T = 25 °C, λ = 220 nm)

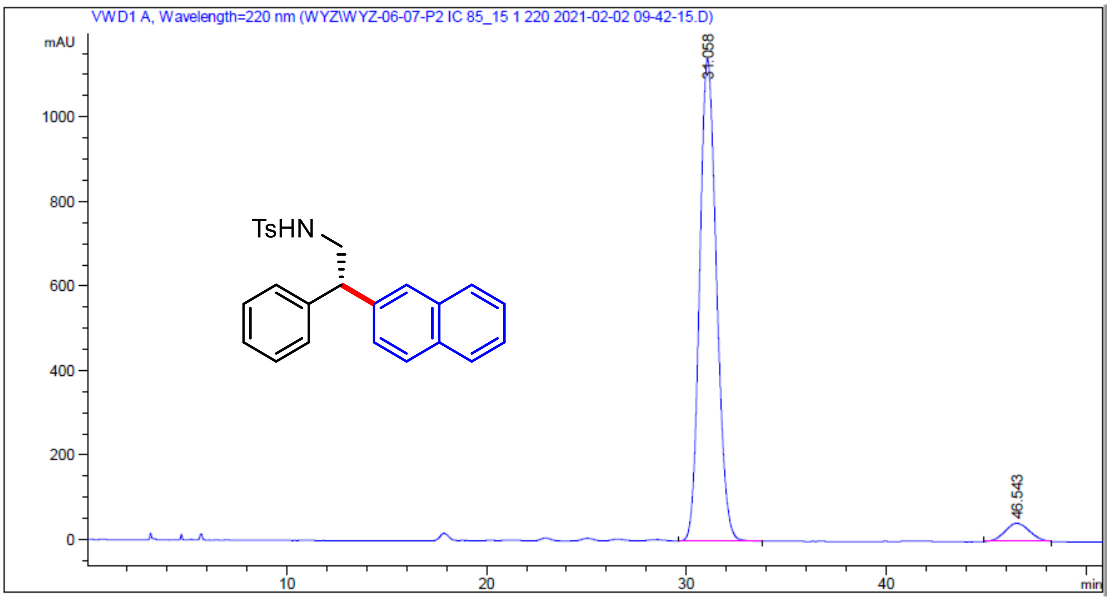

Peak NO	Ret. Time(min)	Area/%
1	31.058	94.9029
2	46.543	5.0971

Supplementary Figure 156. HPLC Chromatography of the Racemic N-(2-(2,3-dihydrobenzofuran-5-yl)-2-phenylethyl)-4-methylbenzenesulfonamide (**3y**) (Daicel Chiralpak IC Column, *n*-Hexane: *i*-PrOH = 80:20, flow rate 1.0 mL/min, T = 25 °C, λ = 230 nm)

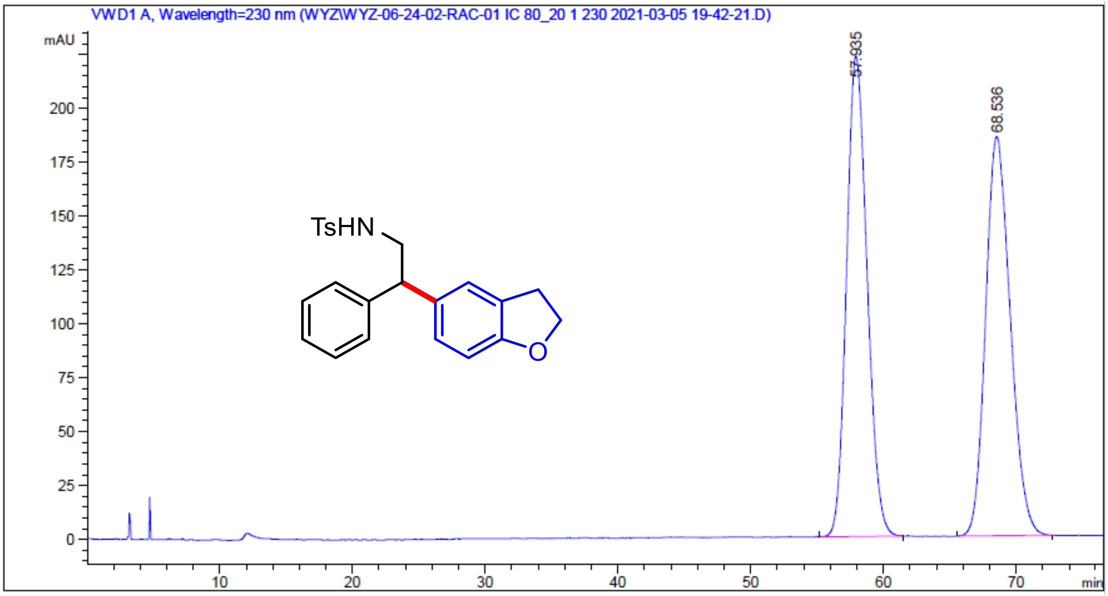

Peak NO	Ret. Time(min)	Area/%
1	57.935	50.0157
2	68.536	49.9843

Supplementary Figure 157. HPLC Chromatography of the (S)-N-(2-(2,3-dihydrobenzofuran-5-yl)-2-phenylethyl)-4-methylbenzenesulfonamide (**3y**) (Daicel Chiralpak IC Column, *n*-Hexane: *i*-PrOH = 80:20, flow rate 1.0 mL/min, T = 25 °C, λ = 230 nm)

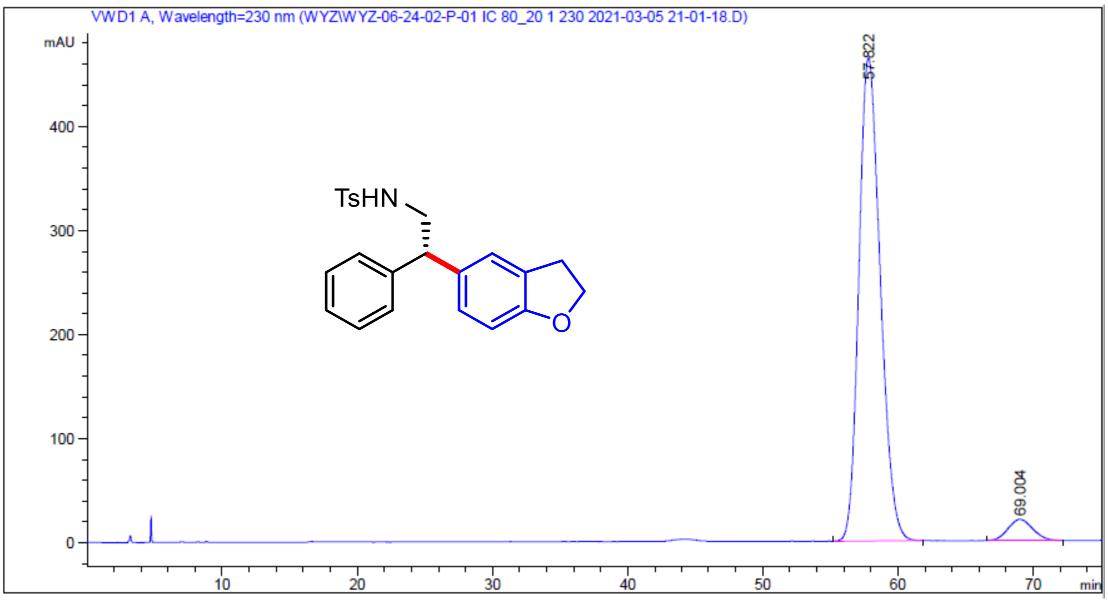

Peak NO	Ret. Time(min)	Area/%
1	57.822	95.1921
2	69.004	4.8079

Supplementary Figure 158. HPLC Chromatography of the Racemic N-(2-(benzo[d][1,3]dioxol-5-yl)-2-phenylethyl)-4-methylbenzenesulfonamide (**3z**) (Daicel Chiralpak IC Column, *n*-Hexane: *i*-PrOH = 85:15, flow rate 1.0 mL/min, T = 25 °C, λ = 220 nm)

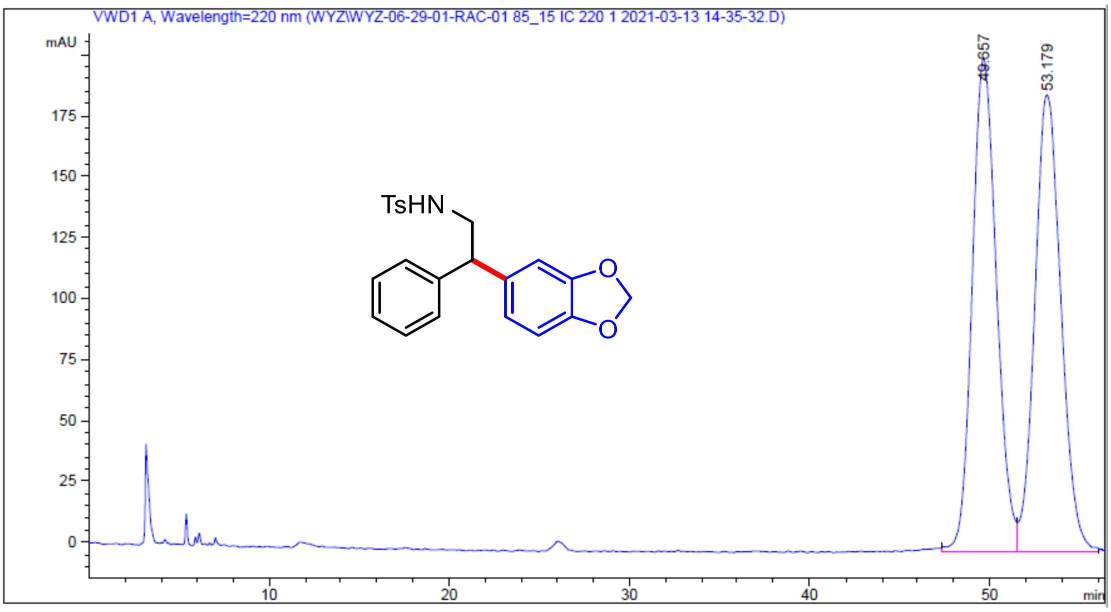

Peak NO	Ret. Time(min)	Area/%
1	49.657	50.0418
2	53.179	49.9582

Supplementary Figure 159. HPLC Chromatography of the (S)-N-(2-(benzo[d][1,3]dioxol-5-yl)-2-phenylethyl)-4-methylbenzenesulfonamide (**3z**) (Daicel Chiralpak IC Column, *n*-Hexane: *i*-PrOH = 85:15, flow rate 1.0 mL/min, T = 25 ℃, λ = 220 nm)

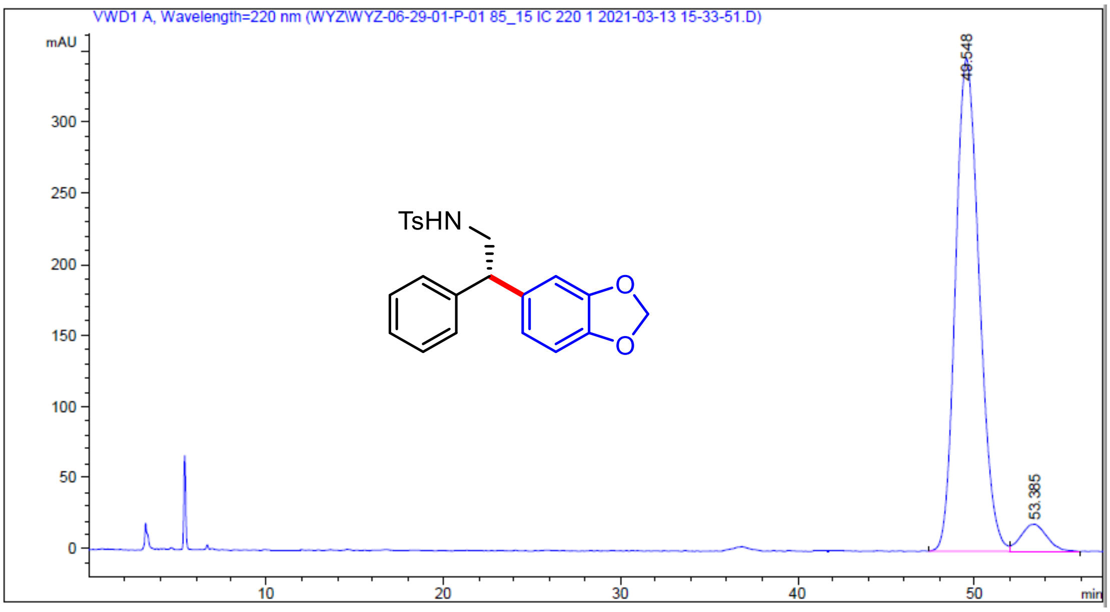

Peak NO	Ret. Time(min)	Area/%
1	49.548	94.3382
2	53.385	5.6618

Supplementary Figure 160. HPLC Chromatography of the Racemic N-(2-(1H-indol-5-yl)-2-phenylethyl)-4-methylbenzenesulfonamide (**3aa**) (Daicel Chiralpak AD-H Column, *n*-Hexane: *i*-PrOH = 85:15, flow rate 1.0 mL/min, T = 25 °C, λ = 220 nm)

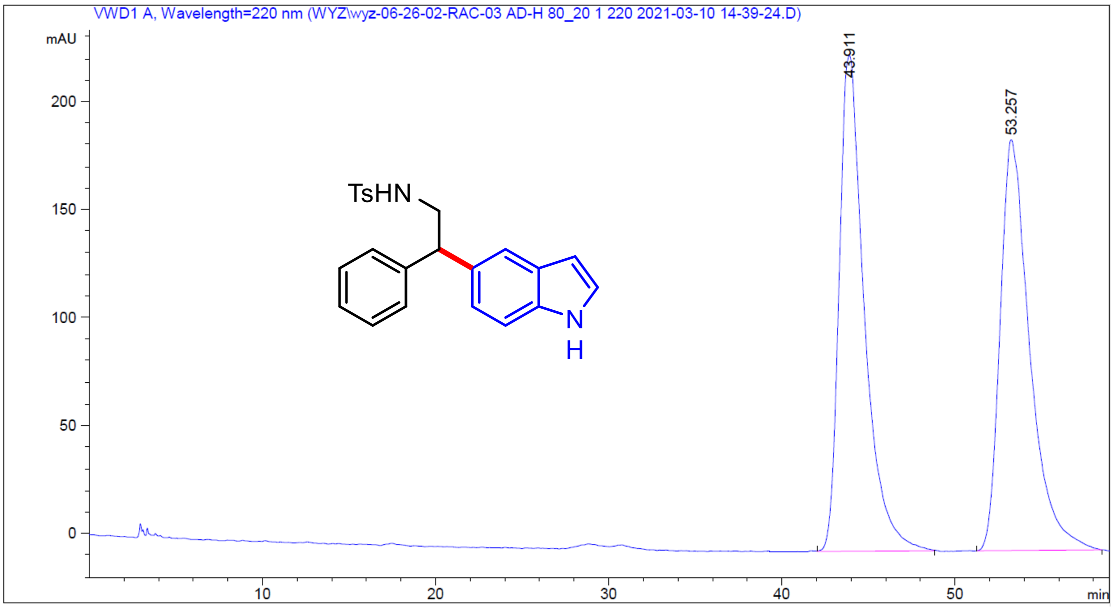

Peak NO	Ret. Time(min)	Area/%
1	43.911	50.6443
2	53.257	49.3557

Supplementary Figure 161. HPLC Chromatography of the (S)-N-(2-(1H-indol-5-yl)-2-phenylethyl)-4-methylbenzenesulfonamide (**3aa**) (Daicel Chiralpak AD-H Column, *n*-Hexane: *i*-PrOH = 85:15, flow rate 1.0 mL/min, T = 25 °C, λ = 220 nm)

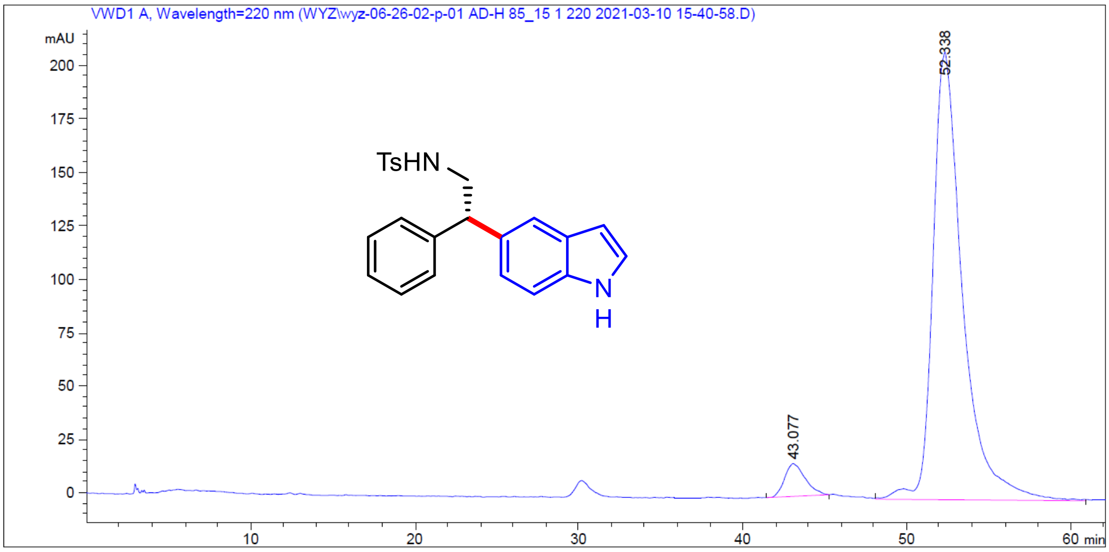

Peak NO	Ret. Time(min)	Area/%
1	43.077	4.8194

2	52.338	95.1806

Supplementary Figure 162. HPLC Chromatography of the Racemic N-(2-(6-fluoropyridin-3-yl)-2-phenylethyl)-4-methylbenzenesulfonamide (**3ab**) (Daicel Chiralpak AD-H Column, *n*-Hexane: *i*-PrOH = 90:10, flow rate 1.0 mL/min, T = 25 °C, λ = 220 nm)

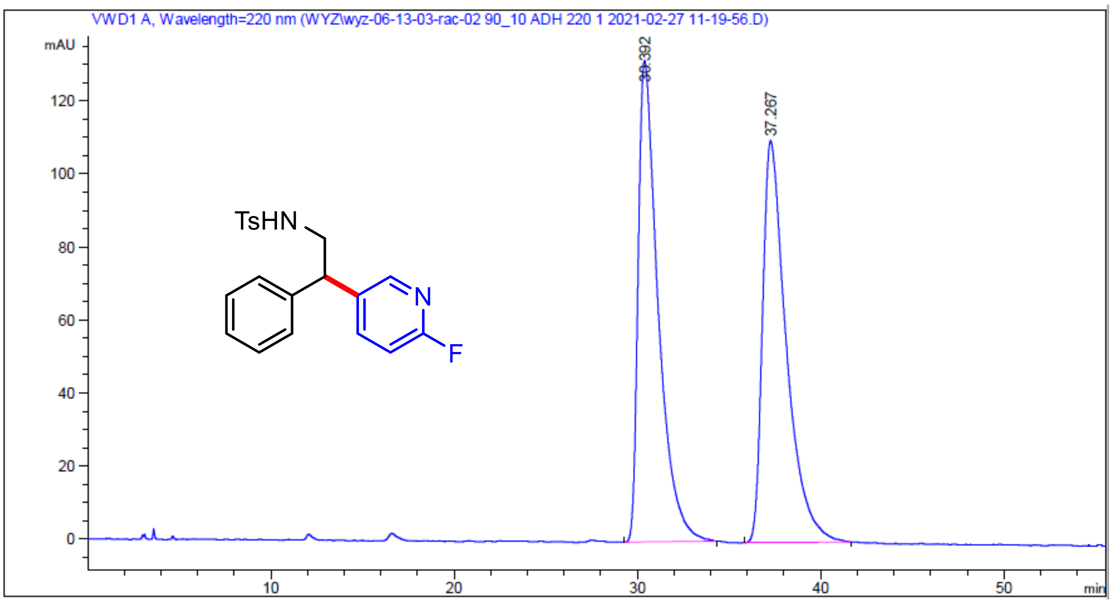

Peak NO	Ret. Time(min)	Area/%
1	30.392	50.1238
2	37.267	49.8762

Supplementary Figure 163. HPLC Chromatography of the (S)-N-(2-(6-fluoropyridin-3-yl)-2-phenylethyl)-4-methylbenzenesulfonamide (**3ab**) (Daicel Chiralpak AD-H Column, *n*-Hexane: *i*-PrOH = 90:10, flow rate 1.0 mL/min, T = 25 °C, λ = 220 nm)

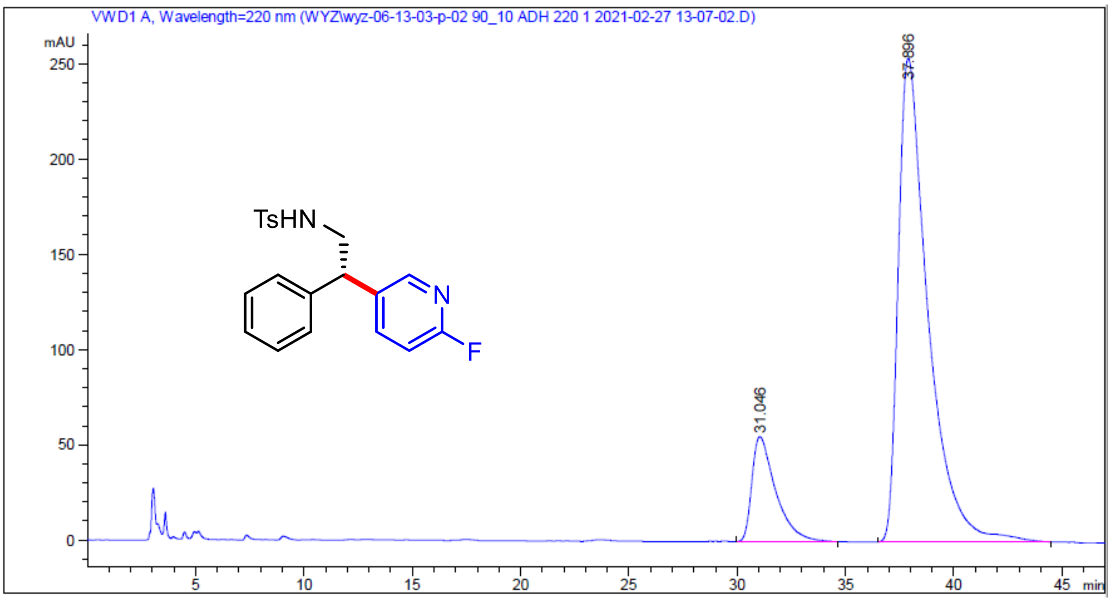

Peak NO	Ret. Time(min)	Area/%
1	31.046	14.7897
2	37.896	85.2103

Supplementary Figure 164. HPLC Chromatography of the Racemic N-(2-(6-methoxypyridin-3-yl)-2-phenylethyl)-4-methylbenzenesulfonamide (**3ac**) (Daicel Chiralpak AD-H Column, *n*-Hexane: *i*-PrOH = 80:20, flow rate 1.0 mL/min, T = 25 °C, λ = 220 nm)

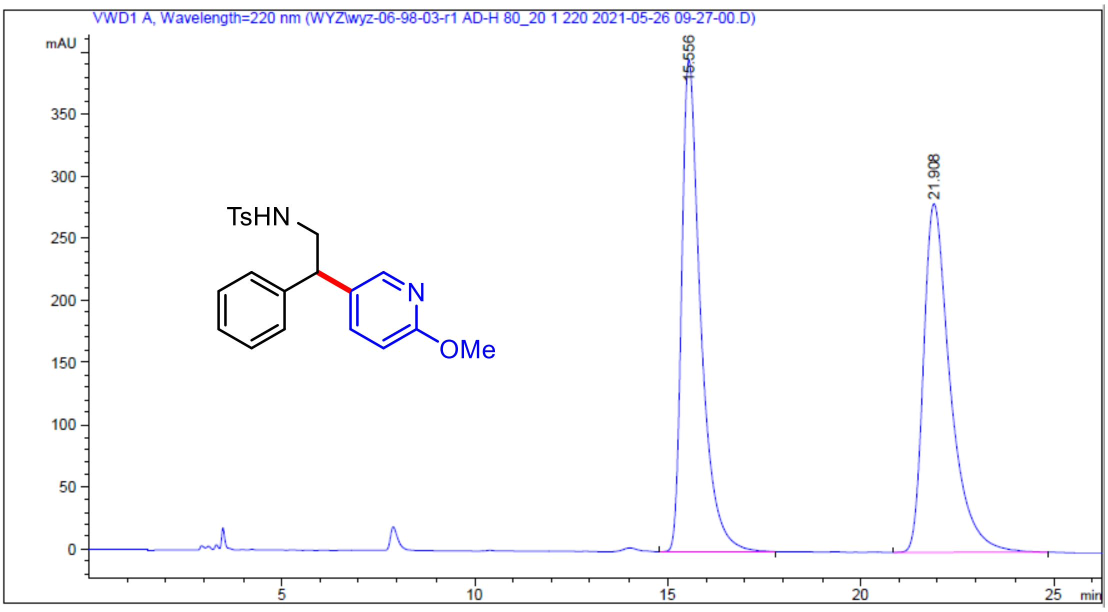

Peak NO	Ret. Time(min)	Area/%
1	15.556	50.0563
2	21.908	49.9437

Supplementary Figure 165. HPLC Chromatography of the (S)-N-(2-(6-methoxypyridin-3-yl)-2-phenylethyl)-4-methylbenzenesulfonamide (**3ac**) (Daicel Chiralpak AD-H Column, *n*-Hexane: *i*-PrOH = 80:20, flow rate 1.0 mL/min, T = 25 °C, λ = 220 nm)

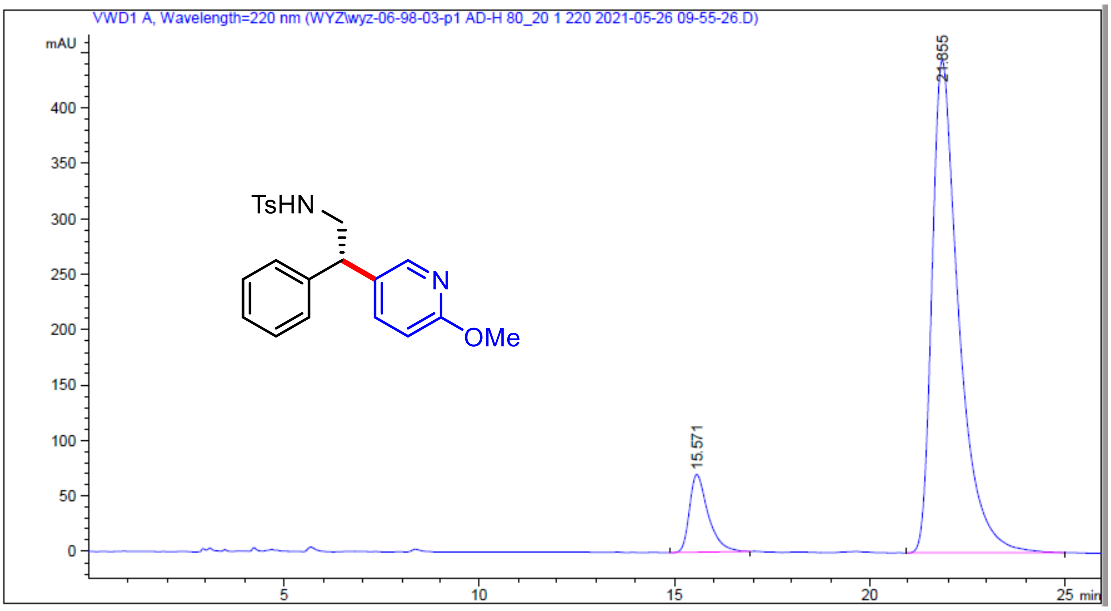

Peak NO	Ret. Time(min)	Area/%
1	15.571	9.8947
2	21.855	90.1053

Supplementary Figure 166. HPLC Chromatography of the Racemic N-(2-(4-methoxyphenyl)-2-(4-(trifluoromethyl)phenyl)ethyl)-4-methylbenzenesulfonamide (**3ad**) (Daicel Chiralpak IC Column, *n*-Hexane: *i*-PrOH = 85:15, flow rate 1.0 mL/min, T = 25 °C, λ = 220 nm)

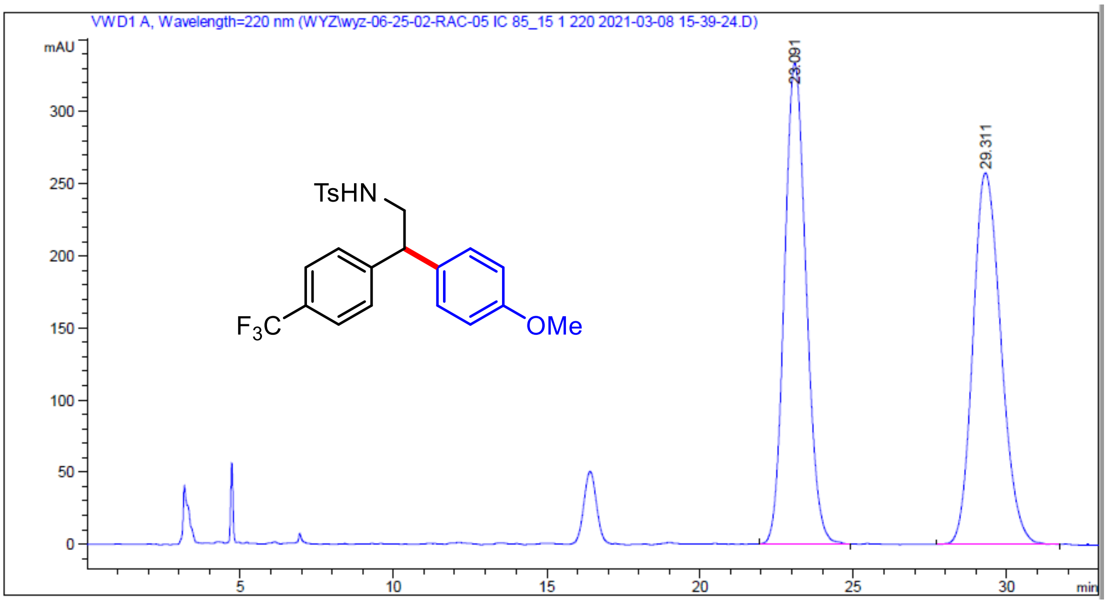

Peak NO	Ret. Time(min)	Area/%
1	23.091	49.9245
2	29.311	50.0755

Supplementary Figure 167. HPLC Chromatography of the (S)-N-(2-(4-methoxyphenyl)-2-(4-(trifluoromethyl)phenyl)ethyl)-4-methylbenzenesulfonamide (**3ad**) (Daicel Chiralpak IC Column, *n*-Hexane: *i*-PrOH = 85:15, flow rate 1.0 mL/min, T = 25 °C, λ = 220 nm)

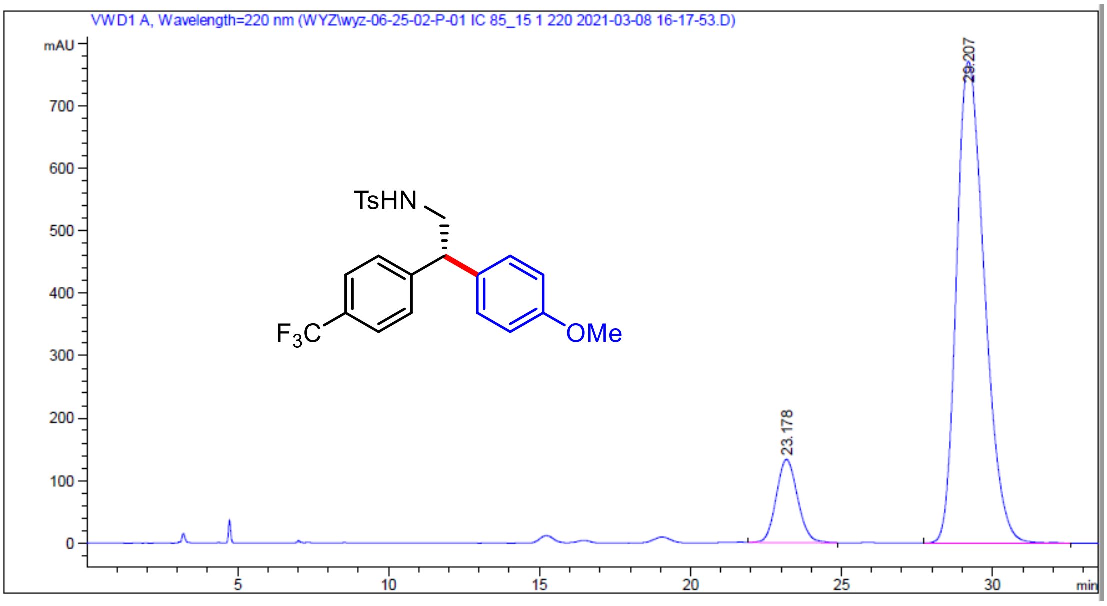

Peak NO	Ret. Time(min)	Area/%
1	23.178	11.6459
2	29.207	88.3541

Supplementary Figure 168. HPLC Chromatography of the Racemic N-(2-(4-fluorophenyl)-2-(4-methoxyphenyl)ethyl)-4-methylbenzenesulfonamide (**3ae**) (Daicel Chiralpak AD-H Column, *n*-Hexane: *i*-PrOH = 85:15, flow rate 1.0 mL/min, T = 25 °C, λ = 220 nm)

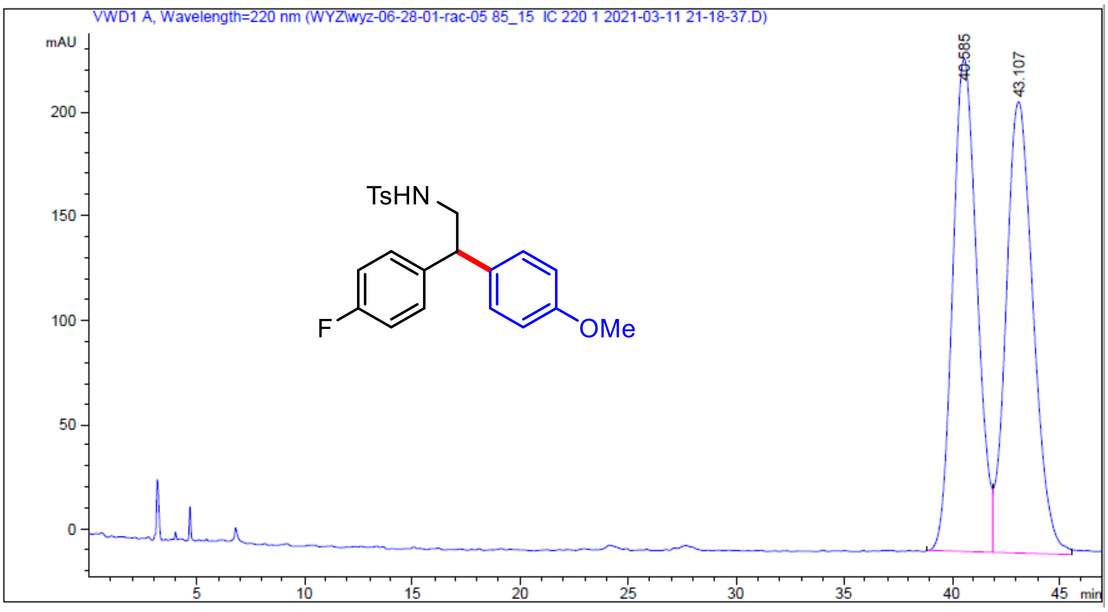

Peak NO	Ret. Time(min)	Area/%
1	40.585	49.2371
2	43.107	50.7629

Supplementary Figure 169. HPLC Chromatography of the (R)-N-(2-(4-fluorophenyl)-2-(4-methoxyphenyl)ethyl)-4-methylbenzenesulfonamide (**3ae**) (Daicel Chiralpak AD-H Column, *n*-Hexane: *i*-PrOH = 85:15, flow rate 1.0 mL/min, T = 25 °C, λ = 220 nm)

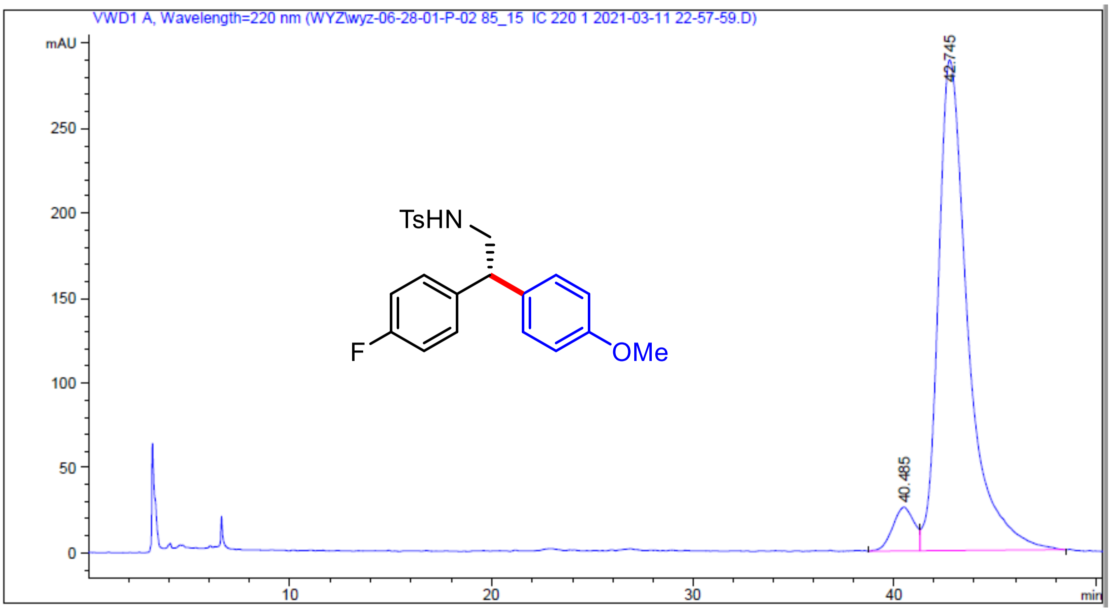

Peak NO	Ret. Time(min)	Area/%
1	40.485	6.0905
2	42.745	93.9095

Supplementary Figure 170. HPLC Chromatography of the Racemic N-(2-(4-chlorophenyl)-2-(4-methoxyphenyl)ethyl)-4-methylbenzenesulfonamide (**3af**) (Daicel Chiralpak IC Column, *n*-Hexane: *i*-PrOH = 85:15, flow rate 1.0 mL/min, T = 25 °C, λ = 220 nm)

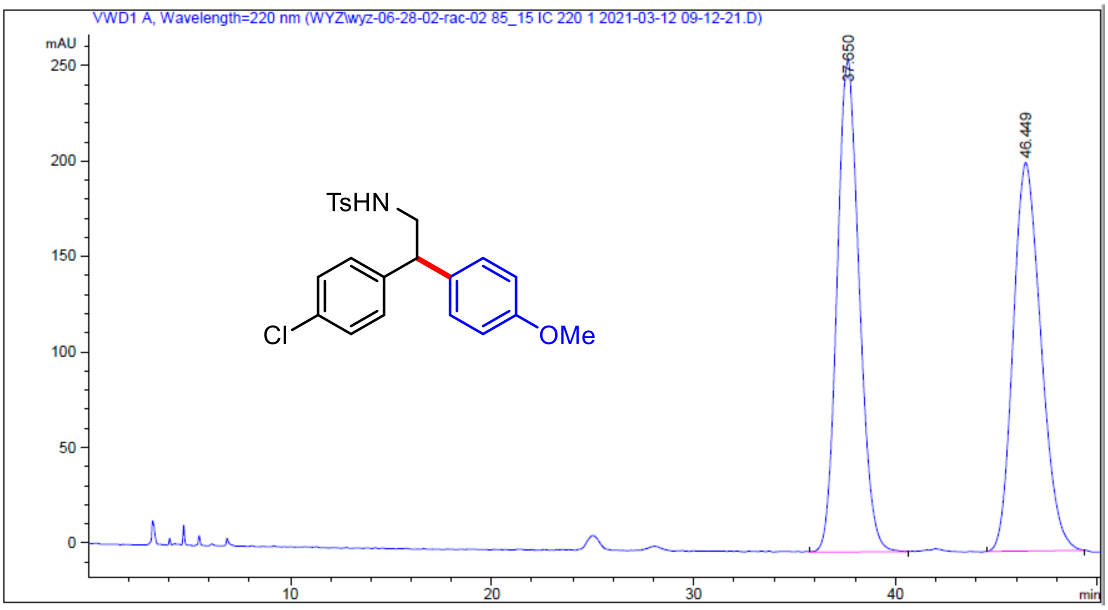

Peak NO	Ret. Time(min)	Area/%
1	37.650	50.2725
2	46.449	49.7275

Supplementary Figure 171. HPLC Chromatography of the (R)-N-(2-(4-chlorophenyl)-2-(4-methoxyphenyl)ethyl)-4-methylbenzenesulfonamide (**3af**) (Daicel Chiralpak IC Column, *n*-Hexane: *i*-PrOH = 85:15, flow rate 1.0 mL/min, T = 25 °C, λ = 220 nm)

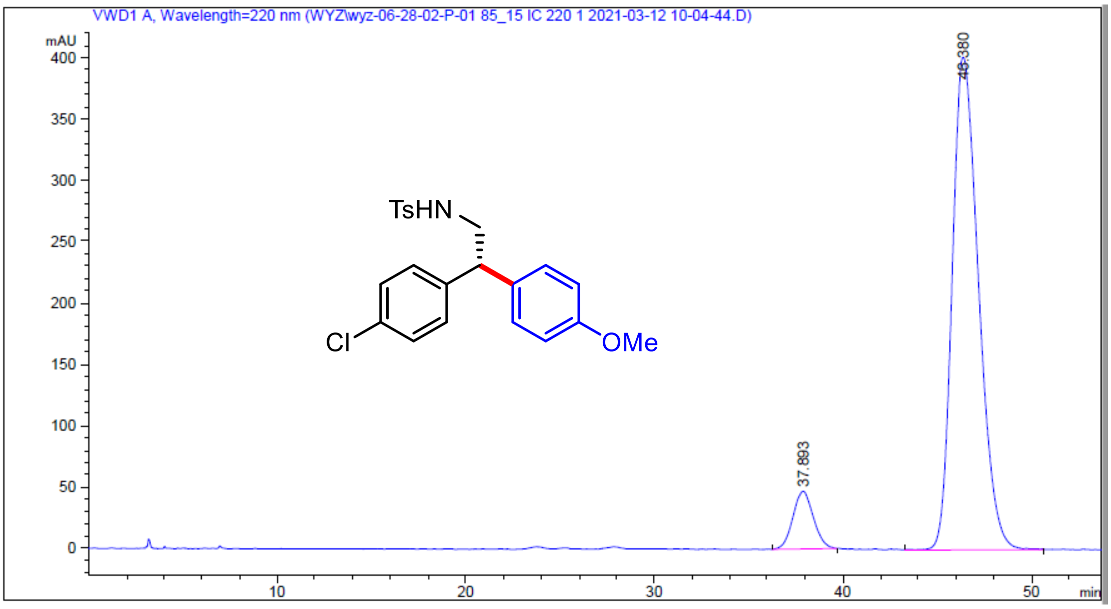

Peak NO	Ret. Time(min)	Area/%
1	37.893	8.3406
2	46.380	91.6594

Supplementary Figure 172. HPLC Chromatography of the Racemic 4-(1-(4-methoxyphenyl)-2-((4-methylphenyl)sulfonamido)ethyl)phenyl acetate (**3ag**) (Daicel Chiralpak IC Column, *n*-Hexane: *i*-PrOH = 85:15, flow rate 1.0 mL/min, T = 25 °C, λ = 220 nm)

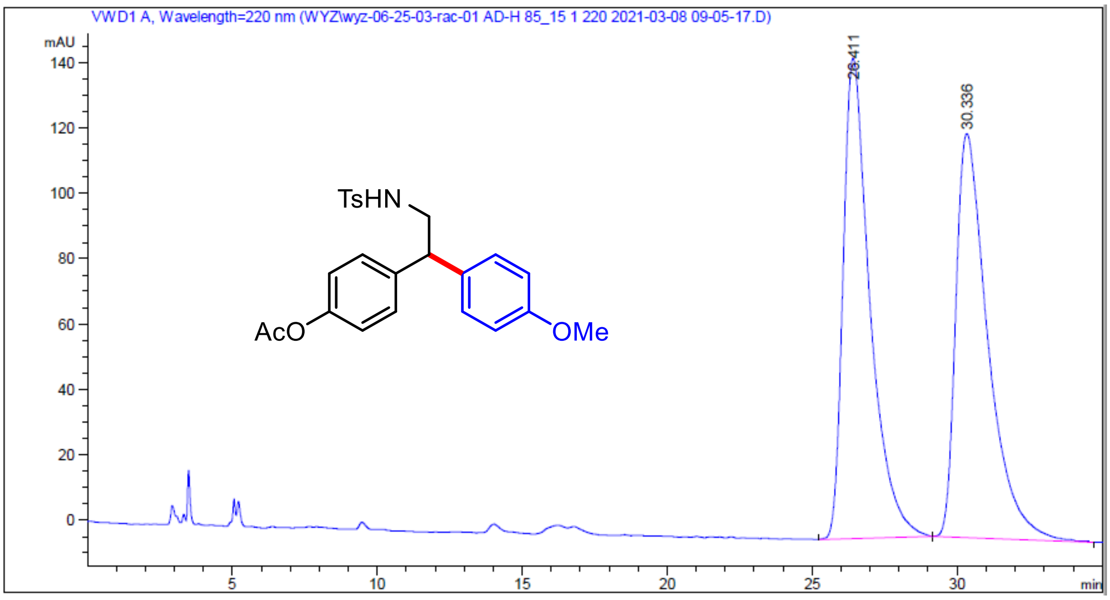

Peak NO	Ret. Time(min)	Area/%
1	26.411	49.8826
2	30.336	50.1174

Supplementary Figure 173. HPLC Chromatography of the (R)-4-(1-(4-methoxyphenyl)-2-((4-methylphenyl)sulfonamido)ethyl)phenyl acetate (**3ag**) (Daicel Chiralpak IC Column, *n*-Hexane: *i*-PrOH = 85:15, flow rate 1.0 mL/min, T = 25 °C, λ = 220 nm)

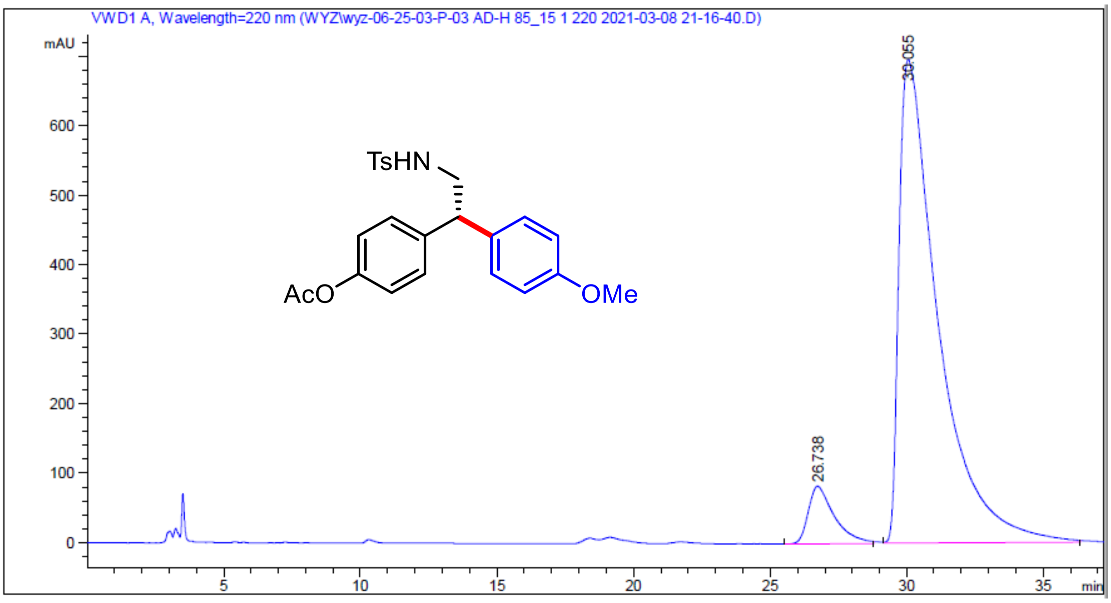

Peak NO	Ret. Time(min)	Area/%
1	26.738	7.2948
2	30.055	92.7052

Supplementary Figure 174. HPLC Chromatography of the Racemic N-(2-(3,4-dimethoxyphenyl)-2-phenylethyl)-N,4-dimethylbenzenesulfonamide (**3va**) (Daicel Chiralpak OD-H Column, *n*-Hexane: *i*-PrOH = 85:15, flow rate 1.0 mL/min, T = 25 °C, λ = 214 nm)

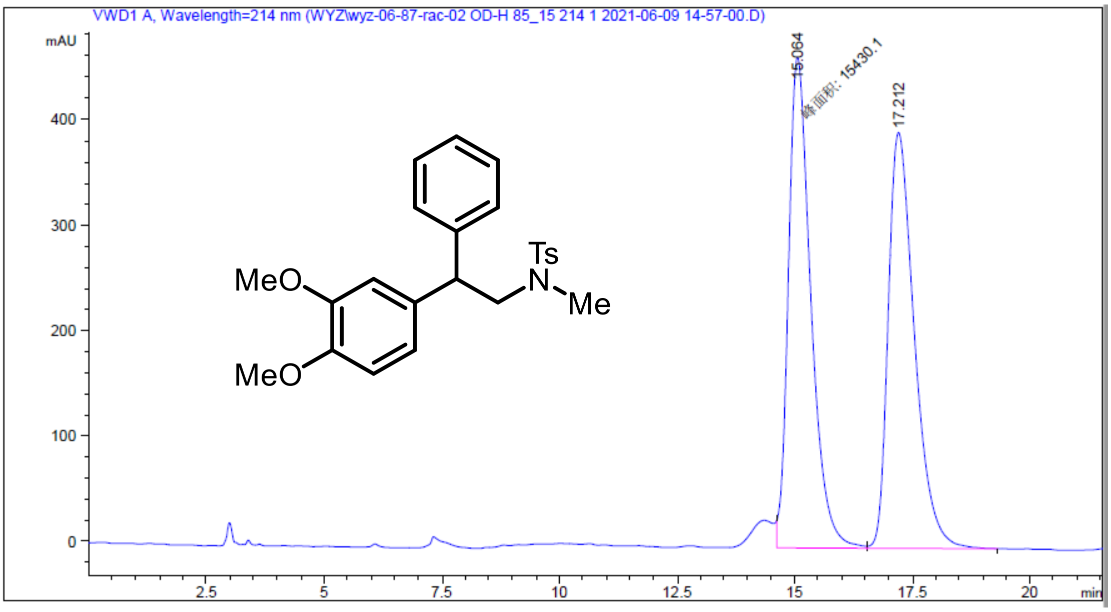

Peak NO	Ret. Time(min)	Area/%
1	15.064	50.3251
2	17.212	49.6749

Supplementary Figure 175. HPLC Chromatography of the (S)-N-(2-(3,4-dimethoxyphenyl)-2-phenylethyl)-N,4-dimethylbenzenesulfonamide (**3va**) (Daicel Chiralpak OD-H Column, *n*-Hexane: *i*-PrOH = 85:15, flow rate 1.0 mL/min, T = 25 °C, λ = 214 nm)

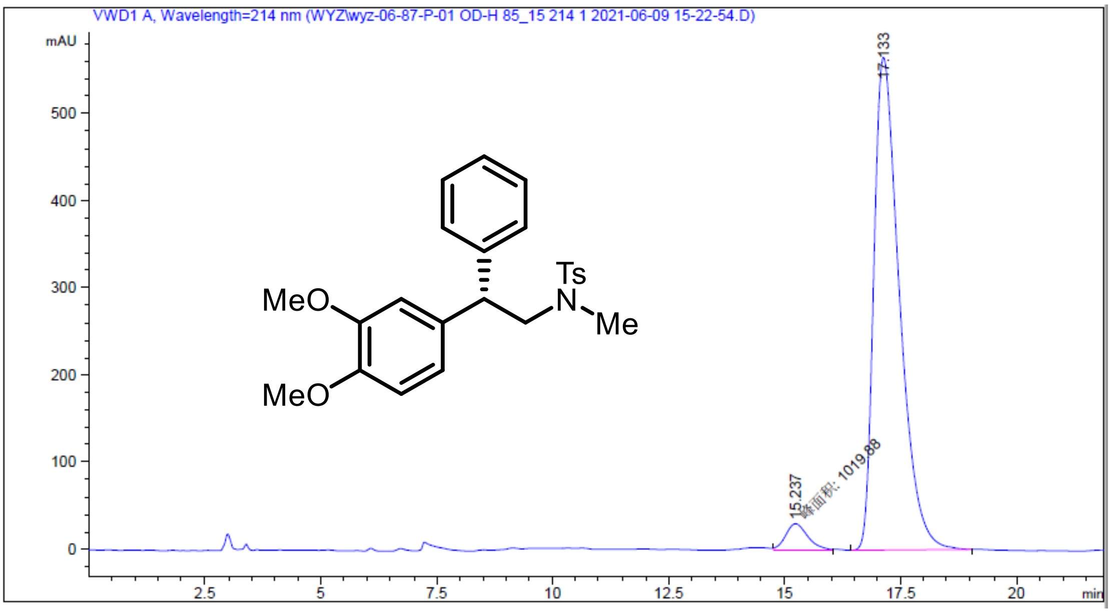

Peak NO	Ret. Time(min)	Area/%
1	15.237	4.4799
2	17.133	95.5201

Supplementary Figure 176. HPLC Chromatography of the Racemic 2-(3,4-dimethoxyphenyl)-N-methyl-2-phenylethan-1-amine (**3vb**) (Daicel Chiralpak IC Column, *n*-Hexane: EtOH : Diethylamine = 80:20:0.05, flow rate 0.8 mL/min, T = 25 °C, λ = 214 nm)

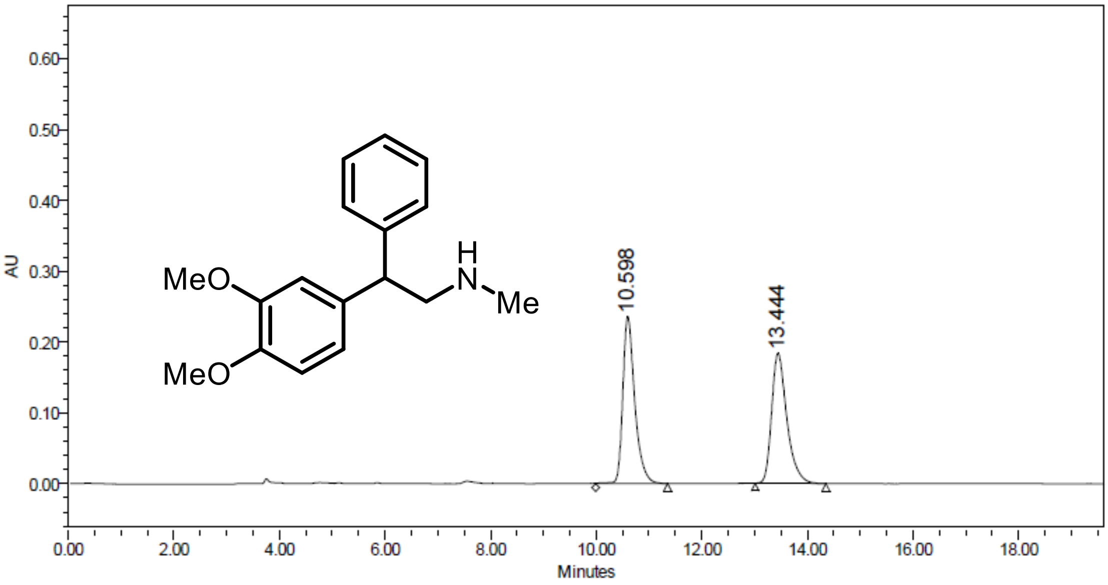

Peak NO	Ret. Time(min)	Area/%
1	10.598	50.34
2	13.444	49.66

Supplementary Figure 177. HPLC Chromatography of the (S)-2-(3,4-dimethoxyphenyl)-N-methyl-2-phenylethan-1-amine (**3vb**) (Daicel Chiralpak IC Column, *n*-Hexane: EtOH : Diethylamine = 80:20:0.05, flow rate 0.8 mL/min, T = 25 °C, λ = 214 nm)

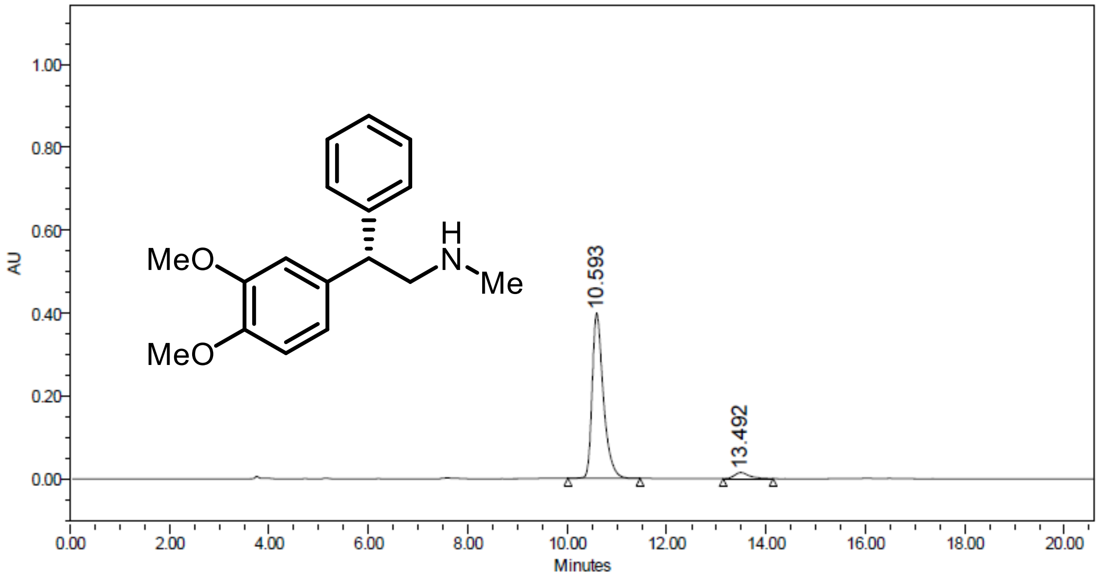

Peak NO	Ret. Time(min)	Area/%
1	10.593	95.39
2	13.492	4.61

Supplementary Figure 178. HPLC Chromatography of the Racemic N-methyl-2-phenyl-2-(3,4,5-trimethoxyphenyl)ethan-1-amine (**3wb**) (Daicel Chiralpak IC Column, *n*-Hexane: EtOH : Diethylamine = 95:5:0.05, flow rate 0.7 mL/min, T = 25 °C, λ = 214 nm)

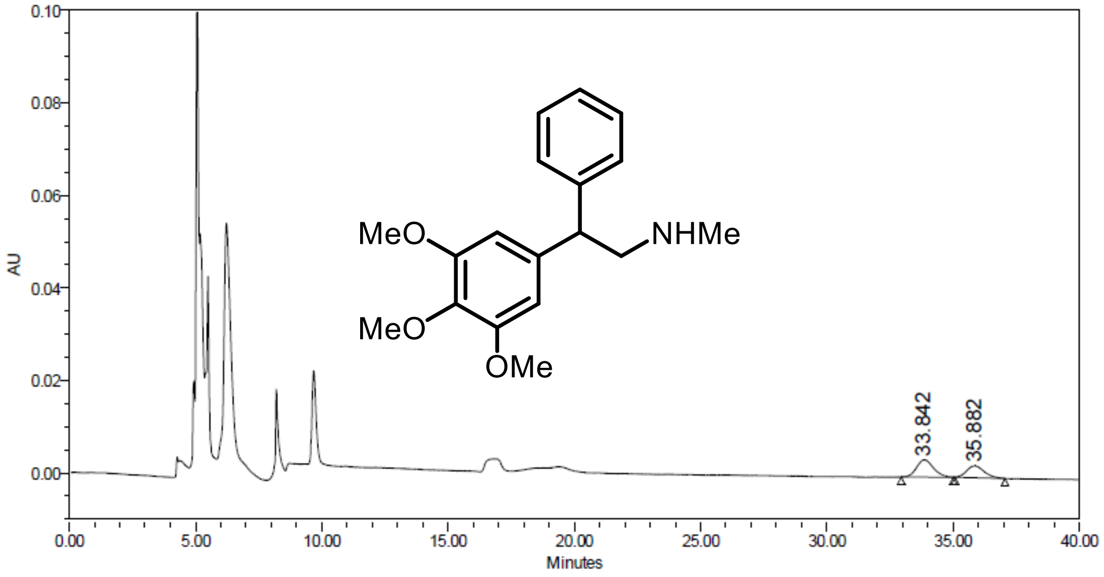

Peak NO	Ret. Time(min)	Area/%
1	33.842	58.55
2	35.882	41.45

Supplementary Figure 179. HPLC Chromatography of the (S)-N-methyl-2-phenyl-2-(3,4,5-trimethoxyphenyl)ethan-1-amine (**3wb**) (Daicel Chiralpak IC Column, *n*-Hexane: EtOH : Diethylamine = 95:5:0.05, flow rate 0.7 mL/min, T = 25 ℃, λ = 214 nm)

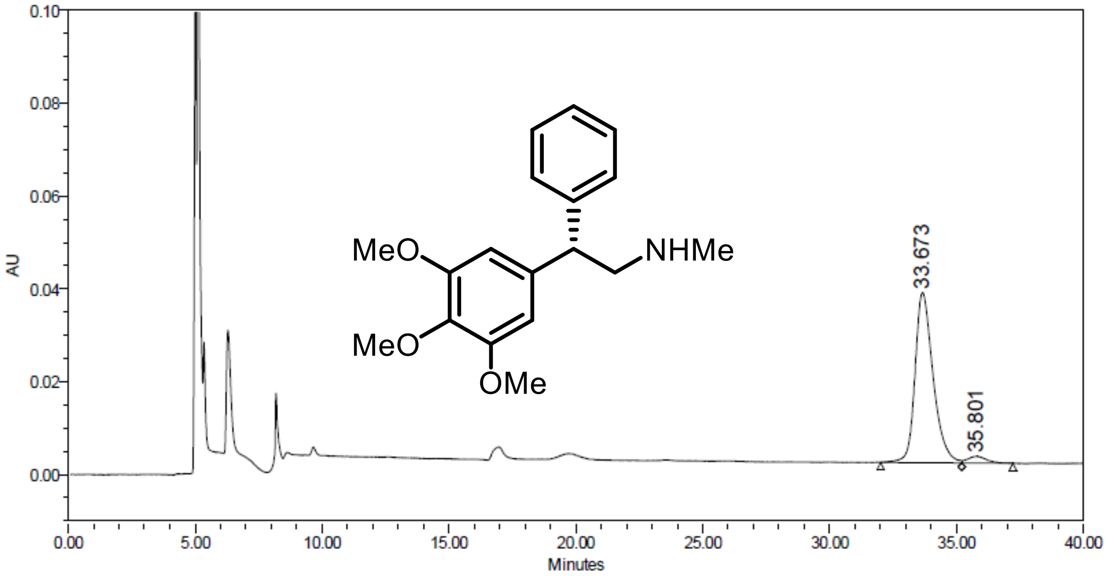

Peak NO	Ret. Time(min)	Area/%
1	33.673	96.18
2	35.801	3.82

4. References

1. X. -Q. Hao, et al. *Tetrahedron*: Asymmetry **26**, 1360 (2015).
2. N.A. Boland, et al. *Org. Biomol. Chem*. **2**, 1995 (2004).
3. Z. Lu, et al. *Nat. Commun*. **10**, 3549 (2019).
4. S.-L. Zhu, et al. *Angew. Chem. Int. Ed*. **59**, 21530 (2020).
5. I. M, et al. *Chem. Pharm. Bull*. **37**, 939 (1989).
6. D. M, et al. *J. Med. Chem.* **29**, 1189 (1986).
