# Supplementary material for: Nickel/biimidazole-catalyzed electrochemical enantioselective reductive cross-coupling of aryl aziridines with aryl iodides
Source: Nat Commun. 2023 Apr 22;14:2322. doi: 10.1038/s41467-023-37965-0 (PMC10122672; doi:10.1038/s41467-023-37965-0)
Supplement: Supplementary file 3 — Description of Additional Supplementary Files [file 41467_2023_37965_MOESM3_ESM.docx]

File Name: Supplementary Data 1

Description: Cartesian coordinates and bond length tables for **3a** and **L7**
