# Supplementary material for: Nickel/biimidazole-catalyzed electrochemical enantioselective reductive cross-coupling of aryl aziridines with aryl iodides
Source: Nat Commun. 2023 Apr 22;14:2322. doi: 10.1038/s41467-023-37965-0 (PMC10122672; doi:10.1038/s41467-023-37965-0)
Supplement: Supplementary file 4 — Supplementary Data 1 [file 41467_2023_37965_MOESM4_ESM.docx]

Table 1. Crystal data and structure refinement for mo_d8v21365_0m.

Identification code mo_d8v21365_0m

Empirical formula C24 H26 N2 O3 S

Formula weight 422.53

Temperature 293(2) K

Wavelength 0.71073 Å

Crystal system Orthorhombic

Space group P 21 21 21

Unit cell dimensions a = 5.7284(2) Å α= 90°.

b = 13.9723(6) Å β= 90°.

c = 28.3107(10) Å γ = 90°.

Volume 2265.96(15) Å3

Z 4

Density (calculated) 1.239 Mg/m3

Absorption coefficient 0.170 mm-1

F(000) 896

Crystal size 0.200 x 0.140 x 0.110 mm3

Theta range for data collection 2.604 to 25.998°.

Index ranges -6<=h<=7, -14<=k<=17, -34<=l<=30

Reflections collected 11455

Independent reflections 4425 [R(int) = 0.0322]

Completeness to theta = 25.242° 99.4 %

Absorption correction Semi-empirical from equivalents

Max. and min. transmission 0.7456 and 0.6774

Refinement method Full-matrix least-squares on F2

Data / restraints / parameters 4425 / 0 / 279

Goodness-of-fit on F2 1.064

Final R indices [I>2sigma(I)] R1 = 0.0402, wR2 = 0.0856

R indices (all data) R1 = 0.0556, wR2 = 0.0945

Absolute structure parameter 0.03(4)

Extinction coefficient 0.019(3)

Largest diff. peak and hole 0.138 and -0.180 e.Å-3

Table 2. Atomic coordinates ( x 104) and equivalent isotropic displacement parameters (Å2x 103)

for mo_d8v21365_0m. U(eq) is defined as one third of the trace of the orthogonalized Uij tensor.

________________________________________________________________________________

x y z U(eq)

________________________________________________________________________________

S(1) 5319(1) 2582(1) 5713(1) 50(1)

O(1) 9519(4) 6946(2) 4105(1) 64(1)

O(2) 4466(4) 2137(2) 5292(1) 68(1)

O(3) 3707(4) 2944(2) 6053(1) 66(1)

N(1) 6917(5) 3460(2) 5539(1) 54(1)

C(1) 8113(6) 4047(2) 5892(1) 51(1)

C(2) 7435(5) 5104(2) 5843(1) 47(1)

C(3) 8393(5) 5653(2) 6264(1) 48(1)

C(4) 7189(6) 5644(2) 6686(1) 66(1)

C(5) 8015(8) 6127(3) 7080(1) 84(1)

C(6) 10078(8) 6613(3) 7055(1) 81(1)

C(7) 11322(7) 6627(3) 6642(1) 74(1)

C(8) 10483(6) 6151(2) 6249(1) 60(1)

C(9) 8045(5) 5542(2) 5371(1) 45(1)

C(10) 6644(5) 6262(2) 5189(1) 50(1)

C(11) 7179(5) 6715(2) 4770(1) 52(1)

C(12) 9135(5) 6455(2) 4518(1) 49(1)

C(13) 10554(6) 5739(2) 4689(1) 57(1)

C(14) 9995(5) 5290(2) 5112(1) 56(1)

C(15) 11580(6) 6736(3) 3848(1) 74(1)

C(16) 7135(5) 1754(2) 6004(1) 45(1)

C(17) 6713(6) 1505(2) 6468(1) 64(1)

C(18) 8142(7) 837(3) 6684(1) 74(1)

C(19) 9988(6) 419(2) 6448(1) 66(1)

C(20) 10388(6) 688(2) 5983(1) 66(1)

C(21) 8970(5) 1349(2) 5764(1) 58(1)

C(22) 11526(8) -318(3) 6689(1) 98(1)

N(2) 5398(10) 3328(4) 7444(2) 144(2)

C(23) 3687(9) 3507(4) 7273(2) 91(1)

C(24) 1516(8) 3706(4) 7046(2) 109(2)

________________________________________________________________________________ Table 3. Bond lengths [Å] and angles [°] for mo_d8v21365_0m.

_____________________________________________________

S(1)-O(3) 1.428(2)

S(1)-O(2) 1.429(2)

S(1)-N(1) 1.608(3)

S(1)-C(16) 1.760(3)

O(1)-C(12) 1.374(3)

O(1)-C(15) 1.417(4)

N(1)-C(1) 1.464(4)

N(1)-H(1) 0.78(3)

C(1)-C(2) 1.533(4)

C(1)-H(1A) 0.9700

C(1)-H(1B) 0.9700

C(2)-C(9) 1.513(4)

C(2)-C(3) 1.519(4)

C(2)-H(2) 0.9800

C(3)-C(4) 1.380(4)

C(3)-C(8) 1.385(4)

C(4)-C(5) 1.386(5)

C(4)-H(4) 0.9300

C(5)-C(6) 1.364(6)

C(5)-H(5) 0.9300

C(6)-C(7) 1.372(5)

C(6)-H(6) 0.9300

C(7)-C(8) 1.382(4)

C(7)-H(7) 0.9300

C(8)-H(8) 0.9300

C(9)-C(14) 1.381(4)

C(9)-C(10) 1.386(4)

C(10)-C(11) 1.379(4)

C(10)-H(10) 0.9300

C(11)-C(12) 1.376(4)

C(11)-H(11) 0.9300

C(12)-C(13) 1.377(4)

C(13)-C(14) 1.390(4)

C(13)-H(13) 0.9300

C(14)-H(14) 0.9300

C(15)-H(15A) 0.9600

C(15)-H(15B) 0.9600

C(15)-H(15C) 0.9600

C(16)-C(21) 1.373(4)

C(16)-C(17) 1.380(4)

C(17)-C(18) 1.384(4)

C(17)-H(17) 0.9300

C(18)-C(19) 1.382(5)

C(18)-H(18) 0.9300

C(19)-C(20) 1.386(4)

C(19)-C(22) 1.517(5)

C(20)-C(21) 1.378(4)

C(20)-H(20) 0.9300

C(21)-H(21) 0.9300

C(22)-H(22A) 0.9600

C(22)-H(22B) 0.9600

C(22)-H(22C) 0.9600

N(2)-C(23) 1.121(6)

C(23)-C(24) 1.428(6)

C(24)-H(24A) 0.9600

C(24)-H(24B) 0.9600

C(24)-H(24C) 0.9600

O(3)-S(1)-O(2) 119.69(14)

O(3)-S(1)-N(1) 107.72(15)

O(2)-S(1)-N(1) 105.80(13)

O(3)-S(1)-C(16) 107.43(13)

O(2)-S(1)-C(16) 107.80(13)

N(1)-S(1)-C(16) 107.92(14)

C(12)-O(1)-C(15) 117.8(3)

C(1)-N(1)-S(1) 119.0(2)

C(1)-N(1)-H(1) 118(3)

S(1)-N(1)-H(1) 112(3)

N(1)-C(1)-C(2) 111.1(2)

N(1)-C(1)-H(1A) 109.4

C(2)-C(1)-H(1A) 109.4

N(1)-C(1)-H(1B) 109.4

C(2)-C(1)-H(1B) 109.4

H(1A)-C(1)-H(1B) 108.0

C(9)-C(2)-C(3) 113.9(2)

C(9)-C(2)-C(1) 114.3(2)

C(3)-C(2)-C(1) 108.9(2)

C(9)-C(2)-H(2) 106.4

C(3)-C(2)-H(2) 106.4

C(1)-C(2)-H(2) 106.4

C(4)-C(3)-C(8) 117.6(3)

C(4)-C(3)-C(2) 119.6(3)

C(8)-C(3)-C(2) 122.8(3)

C(3)-C(4)-C(5) 121.4(3)

C(3)-C(4)-H(4) 119.3

C(5)-C(4)-H(4) 119.3

C(6)-C(5)-C(4) 119.8(4)

C(6)-C(5)-H(5) 120.1

C(4)-C(5)-H(5) 120.1

C(5)-C(6)-C(7) 120.0(4)

C(5)-C(6)-H(6) 120.0

C(7)-C(6)-H(6) 120.0

C(6)-C(7)-C(8) 120.0(3)

C(6)-C(7)-H(7) 120.0

C(8)-C(7)-H(7) 120.0

C(7)-C(8)-C(3) 121.2(3)

C(7)-C(8)-H(8) 119.4

C(3)-C(8)-H(8) 119.4

C(14)-C(9)-C(10) 117.1(3)

C(14)-C(9)-C(2) 123.6(3)

C(10)-C(9)-C(2) 119.3(3)

C(11)-C(10)-C(9) 121.6(3)

C(11)-C(10)-H(10) 119.2

C(9)-C(10)-H(10) 119.2

C(12)-C(11)-C(10) 120.4(3)

C(12)-C(11)-H(11) 119.8

C(10)-C(11)-H(11) 119.8

O(1)-C(12)-C(11) 116.1(3)

O(1)-C(12)-C(13) 124.5(3)

C(11)-C(12)-C(13) 119.4(3)

C(12)-C(13)-C(14) 119.6(3)

C(12)-C(13)-H(13) 120.2

C(14)-C(13)-H(13) 120.2

C(9)-C(14)-C(13) 121.9(3)

C(9)-C(14)-H(14) 119.0

C(13)-C(14)-H(14) 119.0

O(1)-C(15)-H(15A) 109.5

O(1)-C(15)-H(15B) 109.5

H(15A)-C(15)-H(15B) 109.5

O(1)-C(15)-H(15C) 109.5

H(15A)-C(15)-H(15C) 109.5

H(15B)-C(15)-H(15C) 109.5

C(21)-C(16)-C(17) 120.0(3)

C(21)-C(16)-S(1) 119.5(2)

C(17)-C(16)-S(1) 120.5(2)

C(16)-C(17)-C(18) 119.2(3)

C(16)-C(17)-H(17) 120.4

C(18)-C(17)-H(17) 120.4

C(19)-C(18)-C(17) 121.6(3)

C(19)-C(18)-H(18) 119.2

C(17)-C(18)-H(18) 119.2

C(18)-C(19)-C(20) 118.1(3)

C(18)-C(19)-C(22) 120.9(3)

C(20)-C(19)-C(22) 121.0(3)

C(21)-C(20)-C(19) 120.8(3)

C(21)-C(20)-H(20) 119.6

C(19)-C(20)-H(20) 119.6

C(16)-C(21)-C(20) 120.4(3)

C(16)-C(21)-H(21) 119.8

C(20)-C(21)-H(21) 119.8

C(19)-C(22)-H(22A) 109.5

C(19)-C(22)-H(22B) 109.5

H(22A)-C(22)-H(22B) 109.5

C(19)-C(22)-H(22C) 109.5

H(22A)-C(22)-H(22C) 109.5

H(22B)-C(22)-H(22C) 109.5

N(2)-C(23)-C(24) 178.0(7)

C(23)-C(24)-H(24A) 109.5

C(23)-C(24)-H(24B) 109.5

H(24A)-C(24)-H(24B) 109.5

C(23)-C(24)-H(24C) 109.5

H(24A)-C(24)-H(24C) 109.5

H(24B)-C(24)-H(24C) 109.5

_____________________________________________________________

Symmetry transformations used to generate equivalent atoms:

Table 4. Anisotropic displacement parameters (Å2x 103) for mo_d8v21365_0m. The anisotropic

displacement factor exponent takes the form: -2π2[ h2 a*2U11 + ... + 2 h k a* b* U12 ]

______________________________________________________________________________

U11 U22 U33 U23 U13 U12

______________________________________________________________________________

S(1) 52(1) 48(1) 50(1) 1(1) -2(1) -2(1)

O(1) 77(2) 59(1) 56(1) 9(1) 3(1) 3(1)

O(2) 79(1) 67(1) 60(1) -4(1) -19(1) -11(1)

O(3) 59(1) 73(2) 67(1) 3(1) 14(1) 14(1)

N(1) 70(2) 43(2) 47(1) 2(1) 5(1) -4(1)

C(1) 58(2) 41(2) 55(2) 2(1) -6(1) -2(1)

C(2) 43(2) 42(2) 57(2) 3(1) 1(1) 3(1)

C(3) 53(2) 35(2) 56(2) 3(1) 0(2) 4(1)

C(4) 74(2) 57(2) 66(2) 2(2) 10(2) -5(2)

C(5) 110(3) 80(3) 62(2) -7(2) 10(2) 4(3)

C(6) 107(3) 65(2) 71(2) -8(2) -19(2) 0(3)

C(7) 73(2) 68(2) 81(2) 2(2) -16(2) -6(2)

C(8) 56(2) 60(2) 64(2) 1(2) -1(2) 0(2)

C(9) 45(2) 38(2) 53(2) -1(1) -2(1) 2(1)

C(10) 46(2) 49(2) 57(2) -1(1) -2(1) 5(1)

C(11) 52(2) 43(2) 60(2) 3(1) -9(2) 8(1)

C(12) 58(2) 40(2) 50(2) 0(1) -4(1) -4(1)

C(13) 55(2) 50(2) 66(2) -1(2) 12(2) 6(2)

C(14) 56(2) 45(2) 68(2) 9(1) 1(2) 12(2)

C(15) 74(2) 80(3) 69(2) 11(2) 14(2) -7(2)

C(16) 51(2) 39(2) 47(2) 0(1) -1(1) -6(1)

C(17) 79(2) 62(2) 52(2) 1(2) 8(2) 11(2)

C(18) 102(3) 68(2) 50(2) 9(2) -3(2) 15(2)

C(19) 71(2) 51(2) 75(2) 2(2) -13(2) 5(2)

C(20) 60(2) 60(2) 81(2) 2(2) 9(2) 6(2)

C(21) 61(2) 54(2) 58(2) 6(2) 8(2) 2(2)

C(22) 107(3) 80(3) 106(3) 14(2) -27(3) 23(3)

N(2) 118(3) 163(5) 150(4) 7(3) -41(3) 0(4)

C(23) 96(3) 94(3) 82(3) 6(2) -1(3) -1(3)

C(24) 93(3) 141(4) 94(3) 13(3) 9(3) 30(3)

______________________________________________________________________________ Table 5. Hydrogen coordinates ( x 104) and isotropic displacement parameters (Å2x 10 3)

for mo_d8v21365_0m.

________________________________________________________________________________

x y z U(eq)

________________________________________________________________________________

H(1A) 9788 3981 5854 62

H(1B) 7707 3824 6206 62

H(2) 5731 5130 5869 57

H(4) 5794 5307 6707 79

H(5) 7165 6120 7360 101

H(6) 10640 6935 7319 97

H(7) 12730 6957 6625 88

H(8) 11336 6165 5970 72

H(10) 5310 6444 5353 61

H(11) 6214 7198 4657 62

H(13) 11880 5557 4522 68

H(14) 10962 4807 5224 67

H(15A) 11665 7142 3575 112

H(15B) 11554 6079 3750 112

H(15C) 12917 6846 4045 112

H(17) 5485 1783 6633 77

H(18) 7851 665 6996 88

H(20) 11627 419 5818 80

H(21) 9257 1522 5452 69

H(22A) 11163 -944 6569 147

H(22B) 11252 -303 7023 147

H(22C) 13136 -175 6626 147

H(24A) 1295 3268 6788 164

H(24B) 1529 4350 6927 164

H(24C) 265 3634 7268 164

H(1) 7580(60) 3340(20) 5305(11) 67(11)

________________________________________________________________________________ Table 6. Torsion angles [°] for mo_d8v21365_0m.

________________________________________________________________

O(3)-S(1)-N(1)-C(1) 52.7(3)

O(2)-S(1)-N(1)-C(1) -178.2(2)

C(16)-S(1)-N(1)-C(1) -63.0(3)

S(1)-N(1)-C(1)-C(2) -122.9(2)

N(1)-C(1)-C(2)-C(9) -60.6(3)

N(1)-C(1)-C(2)-C(3) 170.7(2)

C(9)-C(2)-C(3)-C(4) 149.5(3)

C(1)-C(2)-C(3)-C(4) -81.6(3)

C(9)-C(2)-C(3)-C(8) -32.2(4)

C(1)-C(2)-C(3)-C(8) 96.7(3)

C(8)-C(3)-C(4)-C(5) 1.0(5)

C(2)-C(3)-C(4)-C(5) 179.3(3)

C(3)-C(4)-C(5)-C(6) -0.9(6)

C(4)-C(5)-C(6)-C(7) 0.4(6)

C(5)-C(6)-C(7)-C(8) 0.1(6)

C(6)-C(7)-C(8)-C(3) -0.1(5)

C(4)-C(3)-C(8)-C(7) -0.4(4)

C(2)-C(3)-C(8)-C(7) -178.7(3)

C(3)-C(2)-C(9)-C(14) 92.1(3)

C(1)-C(2)-C(9)-C(14) -34.0(4)

C(3)-C(2)-C(9)-C(10) -85.4(3)

C(1)-C(2)-C(9)-C(10) 148.5(3)

C(14)-C(9)-C(10)-C(11) -0.6(4)

C(2)-C(9)-C(10)-C(11) 177.1(3)

C(9)-C(10)-C(11)-C(12) 0.4(4)

C(15)-O(1)-C(12)-C(11) 176.7(3)

C(15)-O(1)-C(12)-C(13) -3.3(4)

C(10)-C(11)-C(12)-O(1) 179.9(2)

C(10)-C(11)-C(12)-C(13) -0.1(4)

O(1)-C(12)-C(13)-C(14) 180.0(3)

C(11)-C(12)-C(13)-C(14) -0.1(4)

C(10)-C(9)-C(14)-C(13) 0.4(4)

C(2)-C(9)-C(14)-C(13) -177.1(3)

C(12)-C(13)-C(14)-C(9) -0.1(5)

O(3)-S(1)-C(16)-C(21) -175.1(2)

O(2)-S(1)-C(16)-C(21) 54.7(3)

N(1)-S(1)-C(16)-C(21) -59.2(3)

O(3)-S(1)-C(16)-C(17) 5.5(3)

O(2)-S(1)-C(16)-C(17) -124.8(3)

N(1)-S(1)-C(16)-C(17) 121.4(3)

C(21)-C(16)-C(17)-C(18) -0.8(5)

S(1)-C(16)-C(17)-C(18) 178.6(3)

C(16)-C(17)-C(18)-C(19) 0.5(5)

C(17)-C(18)-C(19)-C(20) 0.1(5)

C(17)-C(18)-C(19)-C(22) -179.5(3)

C(18)-C(19)-C(20)-C(21) -0.4(5)

C(22)-C(19)-C(20)-C(21) 179.2(3)

C(17)-C(16)-C(21)-C(20) 0.5(5)

S(1)-C(16)-C(21)-C(20) -178.9(2)

C(19)-C(20)-C(21)-C(16) 0.1(5)

________________________________________________________________

Symmetry transformations used to generate equivalent atoms:

Table 7. Hydrogen bonds for mo_d8v21365_0m [Å and °].

____________________________________________________________________________

D-H...A d(D-H) d(H...A) d(D...A) <(DHA)

____________________________________________________________________________

N(1)-H(1)...O(2)#1 0.78(3) 2.11(3) 2.892(3) 174(3)

C(24)-H(24A)...O(3) 0.96 2.54 3.257(5) 131.6

____________________________________________________________________________

Symmetry transformations used to generate equivalent atoms:

#1 x+1/2,-y+1/2,-z+1

Table 1. Crystal data and structure refinement for cu_d8v21312_0m.

Identification code cu_d8v21312_0m

Empirical formula C36 H50 N4

Formula weight 538.80

Temperature 293(2) K

Wavelength 1.54178 Å

Crystal system Monoclinic

Space group C 2

Unit cell dimensions a = 23.9420(8) Å α= 90°.

b = 10.6902(3) Å β= 101.0980(10)°.

c = 12.9634(4) Å γ = 90°.

Volume 3255.87(17) Å3

Z 4

Density (calculated) 1.099 Mg/m3

Absorption coefficient 0.487 mm-1

F(000) 1176

Crystal size 0.200 x 0.150 x 0.130 mm3

Theta range for data collection 3.474 to 67.976°.

Index ranges -28<=h<=28, -12<=k<=12, -14<=l<=15

Reflections collected 27936

Independent reflections 5766 [R(int) = 0.0373]

Completeness to theta = 67.679° 97.8 %

Absorption correction Semi-empirical from equivalents

Max. and min. transmission 0.7533 and 0.5503

Refinement method Full-matrix least-squares on F2

Data / restraints / parameters 5766 / 1 / 367

Goodness-of-fit on F2 1.025

Final R indices [I>2sigma(I)] R1 = 0.0532, wR2 = 0.1500

R indices (all data) R1 = 0.0559, wR2 = 0.1550

Absolute structure parameter 0.03(14)

Extinction coefficient n/a

Largest diff. peak and hole 0.218 and -0.133 e.Å-3

Table 2. Atomic coordinates ( x 104) and equivalent isotropic displacement parameters (Å2x 103)

for cu_d8v21312_0m. U(eq) is defined as one third of the trace of the orthogonalized Uij tensor.

________________________________________________________________________________

x y z U(eq)

________________________________________________________________________________

N(1) 4572(1) 6262(2) 977(2) 69(1)

N(2) 4300(1) 5129(2) -506(2) 72(1)

C(1) 4702(1) 5696(2) 101(2) 61(1)

C(2) 4005(1) 5806(3) 1069(3) 76(1)

C(3) 3781(1) 5314(3) -51(2) 69(1)

C(4) 3425(1) 4136(3) -102(2) 71(1)

C(5) 2886(1) 4285(4) 339(3) 90(1)

C(6) 2494(2) 3222(5) -142(4) 105(1)

C(7) 2717(2) 2776(5) -1075(4) 119(2)

C(8) 3204(2) 3632(5) -1197(3) 106(1)

C(9) 4931(1) 6943(2) 1771(2) 60(1)

C(10) 4873(1) 6809(3) 2802(2) 73(1)

C(11) 5228(2) 7498(4) 3560(2) 86(1)

C(12) 5626(2) 8318(4) 3306(2) 82(1)

C(13) 5682(1) 8475(3) 2264(2) 63(1)

C(14) 5329(1) 7777(2) 1514(2) 60(1)

C(15) 6134(1) 9348(3) 1982(2) 77(1)

C(16) 6194(3) 10524(5) 2641(4) 126(2)

C(17) 6710(2) 8658(7) 2206(4) 132(2)

C(18) 6013(2) 9696(6) 820(3) 157(3)

N(3) 4625(1) 4010(2) 6087(2) 69(1)

N(4) 4311(1) 5125(3) 4613(2) 76(1)

C(19) 4714(1) 4501(2) 5156(2) 64(1)

C(20) 4052(1) 4362(4) 6202(3) 88(1)

C(21) 3850(1) 5190(3) 5231(2) 71(1)

C(22) 3272(1) 4877(3) 4575(3) 85(1)

C(23) 2791(1) 5121(4) 5184(3) 94(1)

C(24) 2632(2) 6470(5) 4990(4) 111(1)

C(25) 2848(2) 6888(5) 4057(4) 117(1)

C(26) 3113(2) 5794(5) 3635(3) 104(1)

C(27) 4977(1) 3232(3) 6814(2) 60(1)

C(28) 4923(1) 3271(4) 7861(2) 77(1)

C(29) 5263(2) 2507(5) 8568(2) 97(1)

C(30) 5650(2) 1715(4) 8268(2) 88(1)

C(31) 5711(1) 1641(3) 7223(2) 66(1)

C(32) 5370(1) 2410(3) 6515(2) 61(1)

C(33) 6153(1) 751(3) 6904(2) 80(1)

C(34) 6130(3) -500(5) 7406(6) 150(2)

C(35) 6748(2) 1297(6) 7305(4) 125(2)

C(36) 6087(2) 645(7) 5727(3) 152(3)

________________________________________________________________________________ Table 3. Bond lengths [Å] and angles [°] for cu_d8v21312_0m.

_____________________________________________________

N(1)-C(1) 1.373(3)

N(1)-C(9) 1.410(4)

N(1)-C(2) 1.468(3)

N(2)-C(1) 1.273(4)

N(2)-C(3) 1.487(3)

C(1)-C(1)#1 1.502(4)

C(2)-C(3) 1.541(4)

C(2)-H(2A) 0.9700

C(2)-H(2B) 0.9700

C(3)-C(4) 1.515(4)

C(3)-H(3) 0.9800

C(4)-C(8) 1.515(5)

C(4)-C(5) 1.519(3)

C(4)-H(4) 0.9800

C(5)-C(6) 1.528(5)

C(5)-H(5A) 0.9700

C(5)-H(5B) 0.9700

C(6)-C(7) 1.492(6)

C(6)-H(6A) 0.9700

C(6)-H(6B) 0.9700

C(7)-C(8) 1.515(6)

C(7)-H(7A) 0.9700

C(7)-H(7B) 0.9700

C(8)-H(8A) 0.9700

C(8)-H(8B) 0.9700

C(9)-C(10) 1.378(4)

C(9)-C(14) 1.391(4)

C(10)-C(11) 1.382(5)

C(10)-H(10) 0.9300

C(11)-C(12) 1.382(5)

C(11)-H(11) 0.9300

C(12)-C(13) 1.392(4)

C(12)-H(12) 0.9300

C(13)-C(14) 1.378(4)

C(13)-C(15) 1.526(4)

C(14)-H(14) 0.9300

C(15)-C(16) 1.512(6)

C(15)-C(18) 1.524(4)

C(15)-C(17) 1.541(7)

C(16)-H(16A) 0.9600

C(16)-H(16B) 0.9600

C(16)-H(16C) 0.9600

C(17)-H(17A) 0.9600

C(17)-H(17B) 0.9600

C(17)-H(17C) 0.9600

C(18)-H(18A) 0.9600

C(18)-H(18B) 0.9600

C(18)-H(18C) 0.9600

N(3)-C(19) 1.370(3)

N(3)-C(27) 1.409(4)

N(3)-C(20) 1.456(3)

N(4)-C(19) 1.269(4)

N(4)-C(21) 1.485(3)

C(19)-C(19)#2 1.502(4)

C(20)-C(21) 1.538(4)

C(20)-H(20A) 0.9700

C(20)-H(20B) 0.9700

C(21)-C(22) 1.516(5)

C(21)-H(21) 0.9800

C(22)-C(23) 1.538(4)

C(22)-C(26) 1.553(6)

C(22)-H(22) 0.9800

C(23)-C(24) 1.501(6)

C(23)-H(23A) 0.9700

C(23)-H(23B) 0.9700

C(24)-C(25) 1.472(6)

C(24)-H(24A) 0.9700

C(24)-H(24B) 0.9700

C(25)-C(26) 1.485(7)

C(25)-H(25A) 0.9700

C(25)-H(25B) 0.9700

C(26)-H(26A) 0.9700

C(26)-H(26B) 0.9700

C(27)-C(28) 1.389(4)

C(27)-C(32) 1.395(4)

C(28)-C(29) 1.372(5)

C(28)-H(28) 0.9300

C(29)-C(30) 1.366(6)

C(29)-H(29) 0.9300

C(30)-C(31) 1.392(4)

C(30)-H(30) 0.9300

C(31)-C(32) 1.377(4)

C(31)-C(33) 1.538(4)

C(32)-H(32) 0.9300

C(33)-C(34) 1.493(6)

C(33)-C(36) 1.508(5)

C(33)-C(35) 1.534(6)

C(34)-H(34A) 0.9600

C(34)-H(34B) 0.9600

C(34)-H(34C) 0.9600

C(35)-H(35A) 0.9600

C(35)-H(35B) 0.9600

C(35)-H(35C) 0.9600

C(36)-H(36A) 0.9600

C(36)-H(36B) 0.9600

C(36)-H(36C) 0.9600

C(1)-N(1)-C(9) 128.9(2)

C(1)-N(1)-C(2) 106.3(2)

C(9)-N(1)-C(2) 123.7(2)

C(1)-N(2)-C(3) 106.3(2)

N(2)-C(1)-N(1) 117.2(2)

N(2)-C(1)-C(1)#1 121.6(2)

N(1)-C(1)-C(1)#1 121.2(3)

N(1)-C(2)-C(3) 101.3(2)

N(1)-C(2)-H(2A) 111.5

C(3)-C(2)-H(2A) 111.5

N(1)-C(2)-H(2B) 111.5

C(3)-C(2)-H(2B) 111.5

H(2A)-C(2)-H(2B) 109.3

N(2)-C(3)-C(4) 112.2(2)

N(2)-C(3)-C(2) 104.7(2)

C(4)-C(3)-C(2) 114.6(2)

N(2)-C(3)-H(3) 108.4

C(4)-C(3)-H(3) 108.4

C(2)-C(3)-H(3) 108.4

C(8)-C(4)-C(3) 115.4(3)

C(8)-C(4)-C(5) 102.8(3)

C(3)-C(4)-C(5) 114.1(2)

C(8)-C(4)-H(4) 108.1

C(3)-C(4)-H(4) 108.1

C(5)-C(4)-H(4) 108.1

C(4)-C(5)-C(6) 105.5(3)

C(4)-C(5)-H(5A) 110.6

C(6)-C(5)-H(5A) 110.6

C(4)-C(5)-H(5B) 110.6

C(6)-C(5)-H(5B) 110.6

H(5A)-C(5)-H(5B) 108.8

C(7)-C(6)-C(5) 106.4(3)

C(7)-C(6)-H(6A) 110.5

C(5)-C(6)-H(6A) 110.5

C(7)-C(6)-H(6B) 110.5

C(5)-C(6)-H(6B) 110.5

H(6A)-C(6)-H(6B) 108.6

C(6)-C(7)-C(8) 107.1(3)

C(6)-C(7)-H(7A) 110.3

C(8)-C(7)-H(7A) 110.3

C(6)-C(7)-H(7B) 110.3

C(8)-C(7)-H(7B) 110.3

H(7A)-C(7)-H(7B) 108.6

C(4)-C(8)-C(7) 104.7(3)

C(4)-C(8)-H(8A) 110.8

C(7)-C(8)-H(8A) 110.8

C(4)-C(8)-H(8B) 110.8

C(7)-C(8)-H(8B) 110.8

H(8A)-C(8)-H(8B) 108.9

C(10)-C(9)-C(14) 120.0(3)

C(10)-C(9)-N(1) 119.6(2)

C(14)-C(9)-N(1) 120.4(2)

C(9)-C(10)-C(11) 118.1(3)

C(9)-C(10)-H(10) 120.9

C(11)-C(10)-H(10) 120.9

C(12)-C(11)-C(10) 121.8(3)

C(12)-C(11)-H(11) 119.1

C(10)-C(11)-H(11) 119.1

C(11)-C(12)-C(13) 120.5(3)

C(11)-C(12)-H(12) 119.8

C(13)-C(12)-H(12) 119.8

C(14)-C(13)-C(12) 117.4(3)

C(14)-C(13)-C(15) 121.9(2)

C(12)-C(13)-C(15) 120.7(3)

C(13)-C(14)-C(9) 122.2(2)

C(13)-C(14)-H(14) 118.9

C(9)-C(14)-H(14) 118.9

C(16)-C(15)-C(18) 109.6(4)

C(16)-C(15)-C(13) 111.6(3)

C(18)-C(15)-C(13) 112.3(3)

C(16)-C(15)-C(17) 107.8(4)

C(18)-C(15)-C(17) 107.2(4)

C(13)-C(15)-C(17) 108.2(3)

C(15)-C(16)-H(16A) 109.5

C(15)-C(16)-H(16B) 109.5

H(16A)-C(16)-H(16B) 109.5

C(15)-C(16)-H(16C) 109.5

H(16A)-C(16)-H(16C) 109.5

H(16B)-C(16)-H(16C) 109.5

C(15)-C(17)-H(17A) 109.5

C(15)-C(17)-H(17B) 109.5

H(17A)-C(17)-H(17B) 109.5

C(15)-C(17)-H(17C) 109.5

H(17A)-C(17)-H(17C) 109.5

H(17B)-C(17)-H(17C) 109.5

C(15)-C(18)-H(18A) 109.5

C(15)-C(18)-H(18B) 109.5

H(18A)-C(18)-H(18B) 109.5

C(15)-C(18)-H(18C) 109.5

H(18A)-C(18)-H(18C) 109.5

H(18B)-C(18)-H(18C) 109.5

C(19)-N(3)-C(27) 129.8(2)

C(19)-N(3)-C(20) 107.4(2)

C(27)-N(3)-C(20) 122.6(2)

C(19)-N(4)-C(21) 106.9(2)

N(4)-C(19)-N(3) 117.4(2)

N(4)-C(19)-C(19)#2 119.1(2)

N(3)-C(19)-C(19)#2 122.9(3)

N(3)-C(20)-C(21) 102.7(2)

N(3)-C(20)-H(20A) 111.2

C(21)-C(20)-H(20A) 111.2

N(3)-C(20)-H(20B) 111.2

C(21)-C(20)-H(20B) 111.2

H(20A)-C(20)-H(20B) 109.1

N(4)-C(21)-C(22) 112.3(2)

N(4)-C(21)-C(20) 105.3(2)

C(22)-C(21)-C(20) 116.2(3)

N(4)-C(21)-H(21) 107.5

C(22)-C(21)-H(21) 107.5

C(20)-C(21)-H(21) 107.5

C(21)-C(22)-C(23) 111.5(3)

C(21)-C(22)-C(26) 111.1(3)

C(23)-C(22)-C(26) 101.6(3)

C(21)-C(22)-H(22) 110.8

C(23)-C(22)-H(22) 110.8

C(26)-C(22)-H(22) 110.8

C(24)-C(23)-C(22) 105.5(3)

C(24)-C(23)-H(23A) 110.6

C(22)-C(23)-H(23A) 110.6

C(24)-C(23)-H(23B) 110.6

C(22)-C(23)-H(23B) 110.6

H(23A)-C(23)-H(23B) 108.8

C(25)-C(24)-C(23) 108.2(4)

C(25)-C(24)-H(24A) 110.1

C(23)-C(24)-H(24A) 110.1

C(25)-C(24)-H(24B) 110.1

C(23)-C(24)-H(24B) 110.1

H(24A)-C(24)-H(24B) 108.4

C(24)-C(25)-C(26) 107.8(4)

C(24)-C(25)-H(25A) 110.1

C(26)-C(25)-H(25A) 110.1

C(24)-C(25)-H(25B) 110.1

C(26)-C(25)-H(25B) 110.1

H(25A)-C(25)-H(25B) 108.5

C(25)-C(26)-C(22) 105.4(3)

C(25)-C(26)-H(26A) 110.7

C(22)-C(26)-H(26A) 110.7

C(25)-C(26)-H(26B) 110.7

C(22)-C(26)-H(26B) 110.7

H(26A)-C(26)-H(26B) 108.8

C(28)-C(27)-C(32) 119.0(3)

C(28)-C(27)-N(3) 118.6(3)

C(32)-C(27)-N(3) 122.4(2)

C(29)-C(28)-C(27) 118.7(3)

C(29)-C(28)-H(28) 120.7

C(27)-C(28)-H(28) 120.7

C(30)-C(29)-C(28) 121.8(3)

C(30)-C(29)-H(29) 119.1

C(28)-C(29)-H(29) 119.1

C(29)-C(30)-C(31) 121.1(3)

C(29)-C(30)-H(30) 119.5

C(31)-C(30)-H(30) 119.5

C(32)-C(31)-C(30) 117.0(3)

C(32)-C(31)-C(33) 122.8(2)

C(30)-C(31)-C(33) 120.2(3)

C(31)-C(32)-C(27) 122.4(2)

C(31)-C(32)-H(32) 118.8

C(27)-C(32)-H(32) 118.8

C(34)-C(33)-C(36) 111.6(5)

C(34)-C(33)-C(35) 107.6(4)

C(36)-C(33)-C(35) 106.3(4)

C(34)-C(33)-C(31) 110.6(3)

C(36)-C(33)-C(31) 112.0(3)

C(35)-C(33)-C(31) 108.4(3)

C(33)-C(34)-H(34A) 109.5

C(33)-C(34)-H(34B) 109.5

H(34A)-C(34)-H(34B) 109.5

C(33)-C(34)-H(34C) 109.5

H(34A)-C(34)-H(34C) 109.5

H(34B)-C(34)-H(34C) 109.5

C(33)-C(35)-H(35A) 109.5

C(33)-C(35)-H(35B) 109.5

H(35A)-C(35)-H(35B) 109.5

C(33)-C(35)-H(35C) 109.5

H(35A)-C(35)-H(35C) 109.5

H(35B)-C(35)-H(35C) 109.5

C(33)-C(36)-H(36A) 109.5

C(33)-C(36)-H(36B) 109.5

H(36A)-C(36)-H(36B) 109.5

C(33)-C(36)-H(36C) 109.5

H(36A)-C(36)-H(36C) 109.5

H(36B)-C(36)-H(36C) 109.5

_____________________________________________________________

Symmetry transformations used to generate equivalent atoms:

#1 -x+1,y,-z #2 -x+1,y,-z+1

Table 4. Anisotropic displacement parameters (Å2x 103) for cu_d8v21312_0m. The anisotropic

displacement factor exponent takes the form: -2π2[ h2 a*2U11 + ... + 2 h k a* b* U12 ]

______________________________________________________________________________

U11 U22 U33 U23 U13 U12

______________________________________________________________________________

N(1) 73(1) 69(1) 79(1) -12(1) 49(1) -10(1)

N(2) 76(1) 67(1) 87(1) -7(1) 48(1) -4(1)

C(1) 68(2) 50(1) 77(2) -2(1) 44(1) -2(1)

C(2) 73(2) 75(2) 96(2) -16(2) 54(2) -8(1)

C(3) 70(2) 60(1) 89(2) -4(1) 43(1) -2(1)

C(4) 70(2) 61(1) 91(2) -2(1) 38(1) -1(1)

C(5) 77(2) 92(2) 113(2) -22(2) 51(2) -15(2)

C(6) 85(2) 117(3) 121(3) -19(2) 37(2) -28(2)

C(7) 110(3) 128(4) 129(3) -45(3) 49(3) -34(3)

C(8) 120(3) 103(3) 110(3) -31(2) 57(2) -25(2)

C(9) 66(1) 55(1) 66(1) 4(1) 34(1) 8(1)

C(10) 80(2) 76(2) 75(2) 14(1) 44(1) 7(1)

C(11) 106(2) 103(2) 57(2) 12(2) 38(2) 7(2)

C(12) 94(2) 99(2) 54(1) 1(2) 19(1) 0(2)

C(13) 67(1) 69(1) 54(1) 6(1) 16(1) 6(1)

C(14) 71(1) 63(1) 52(1) 7(1) 26(1) 3(1)

C(15) 82(2) 87(2) 59(1) 2(1) 12(1) -18(2)

C(16) 176(5) 92(3) 115(3) -15(2) 38(3) -37(3)

C(17) 85(2) 157(5) 164(4) 1(4) 46(3) -14(3)

C(18) 175(5) 206(6) 80(2) 47(3) -3(3) -119(5)

N(3) 69(1) 76(1) 74(1) 6(1) 45(1) 8(1)

N(4) 69(1) 82(2) 89(2) 14(1) 45(1) 7(1)

C(19) 66(2) 64(1) 71(2) -3(1) 38(1) 0(1)

C(20) 71(2) 109(2) 99(2) 22(2) 52(2) 20(2)

C(21) 66(1) 75(2) 84(2) 3(1) 40(1) 4(1)

C(22) 73(2) 83(2) 108(2) -16(2) 42(2) -8(1)

C(23) 67(2) 103(3) 123(3) 12(2) 44(2) 0(2)

C(24) 103(3) 111(3) 130(3) 2(3) 55(2) 16(2)

C(25) 119(3) 117(3) 121(3) 26(3) 37(3) 27(3)

C(26) 86(2) 148(4) 80(2) 5(2) 22(2) -2(2)

C(27) 63(1) 66(1) 60(1) -9(1) 29(1) -10(1)

C(28) 78(2) 98(2) 64(2) -14(2) 36(1) -6(2)

C(29) 102(2) 144(3) 53(2) -2(2) 32(2) 2(2)

C(30) 90(2) 118(3) 58(2) 7(2) 18(1) 8(2)

C(31) 70(2) 69(2) 61(1) -7(1) 16(1) -6(1)

C(32) 69(1) 68(1) 52(1) -7(1) 23(1) -1(1)

C(33) 83(2) 78(2) 78(2) -4(2) 16(1) 13(2)

C(34) 176(5) 76(3) 213(6) 16(3) 73(5) 19(3)

C(35) 81(2) 151(4) 147(4) -29(4) 32(2) 6(3)

C(36) 147(4) 203(6) 99(3) -50(3) 9(3) 97(4)

______________________________________________________________________________ Table 5. Hydrogen coordinates ( x 104) and isotropic displacement parameters (Å2x 10 3)

for cu_d8v21312_0m.

________________________________________________________________________________

x y z U(eq)

________________________________________________________________________________

H(2A) 3769 6477 1251 92

H(2B) 4029 5144 1587 92

H(3) 3549 5971 -453 83

H(4) 3658 3484 303 85

H(5A) 2710 5091 144 108

H(5B) 2969 4221 1100 108

H(6A) 2499 2550 362 127

H(6B) 2106 3521 -353 127

H(7A) 2420 2807 -1700 143

H(7B) 2850 1919 -970 143

H(8A) 3499 3173 -1454 127

H(8B) 3071 4308 -1683 127

H(10) 4602 6271 2982 88

H(11) 5198 7408 4261 103

H(12) 5860 8768 3834 98

H(14) 5358 7866 813 72

H(16A) 5841 10978 2508 189

H(16B) 6491 11037 2463 189

H(16C) 6288 10304 3372 189

H(17A) 6810 8467 2942 198

H(17B) 6998 9182 2009 198

H(17C) 6680 7897 1807 198

H(18A) 6021 8956 404 236

H(18B) 6298 10273 683 236

H(18C) 5645 10079 641 236

H(20A) 4058 4825 6847 106

H(20B) 3811 3632 6194 106

H(21) 3833 6053 5478 85

H(22) 3262 4009 4328 102

H(23A) 2468 4583 4930 113

H(23B) 2922 4967 5929 113

H(24A) 2222 6565 4872 133

H(24B) 2799 6970 5595 133

H(25A) 3126 7548 4247 141

H(25B) 2537 7210 3530 141

H(26A) 3450 6046 3376 125

H(26B) 2848 5406 3066 125

H(28) 4661 3804 8078 92

H(29) 5229 2530 9270 117

H(30) 5877 1217 8770 106

H(32) 5402 2380 5812 74

H(34A) 6173 -399 8153 225

H(34B) 6432 -1017 7254 225

H(34C) 5770 -888 7135 225

H(35A) 6777 2098 6984 188

H(35B) 7030 742 7126 188

H(35C) 6809 1393 8054 188

H(36A) 5725 277 5440 227

H(36B) 6386 127 5561 227

H(36C) 6110 1462 5431 227

________________________________________________________________________________ Table 6. Torsion angles [°] for cu_d8v21312_0m.

________________________________________________________________

C(3)-N(2)-C(1)-N(1) 1.9(3)

C(3)-N(2)-C(1)-C(1)#1 179.0(2)

C(9)-N(1)-C(1)-N(2) 179.9(3)

C(2)-N(1)-C(1)-N(2) 11.7(3)

C(9)-N(1)-C(1)-C(1)#1 2.8(4)

C(2)-N(1)-C(1)-C(1)#1 -165.4(2)

C(1)-N(1)-C(2)-C(3) -18.7(3)

C(9)-N(1)-C(2)-C(3) 172.3(3)

C(1)-N(2)-C(3)-C(4) -138.8(3)

C(1)-N(2)-C(3)-C(2) -14.0(3)

N(1)-C(2)-C(3)-N(2) 19.7(3)

N(1)-C(2)-C(3)-C(4) 142.9(2)

N(2)-C(3)-C(4)-C(8) -60.4(4)

C(2)-C(3)-C(4)-C(8) -179.6(3)

N(2)-C(3)-C(4)-C(5) -179.1(3)

C(2)-C(3)-C(4)-C(5) 61.7(4)

C(8)-C(4)-C(5)-C(6) 33.6(4)

C(3)-C(4)-C(5)-C(6) 159.3(3)

C(4)-C(5)-C(6)-C(7) -18.3(5)

C(5)-C(6)-C(7)-C(8) -4.5(6)

C(3)-C(4)-C(8)-C(7) -161.2(3)

C(5)-C(4)-C(8)-C(7) -36.4(4)

C(6)-C(7)-C(8)-C(4) 25.7(6)

C(1)-N(1)-C(9)-C(10) -141.2(3)

C(2)-N(1)-C(9)-C(10) 25.1(4)

C(1)-N(1)-C(9)-C(14) 41.0(4)

C(2)-N(1)-C(9)-C(14) -152.7(3)

C(14)-C(9)-C(10)-C(11) -1.6(4)

N(1)-C(9)-C(10)-C(11) -179.4(3)

C(9)-C(10)-C(11)-C(12) 1.1(5)

C(10)-C(11)-C(12)-C(13) 0.1(6)

C(11)-C(12)-C(13)-C(14) -0.6(5)

C(11)-C(12)-C(13)-C(15) -178.1(3)

C(12)-C(13)-C(14)-C(9) 0.0(4)

C(15)-C(13)-C(14)-C(9) 177.4(3)

C(10)-C(9)-C(14)-C(13) 1.2(4)

N(1)-C(9)-C(14)-C(13) 178.9(2)

C(14)-C(13)-C(15)-C(16) 143.4(3)

C(12)-C(13)-C(15)-C(16) -39.2(5)

C(14)-C(13)-C(15)-C(18) 20.0(5)

C(12)-C(13)-C(15)-C(18) -162.6(4)

C(14)-C(13)-C(15)-C(17) -98.1(4)

C(12)-C(13)-C(15)-C(17) 79.3(4)

C(21)-N(4)-C(19)-N(3) 3.3(4)

C(21)-N(4)-C(19)-C(19)#2 -167.4(2)

C(27)-N(3)-C(19)-N(4) 176.1(3)

C(20)-N(3)-C(19)-N(4) 0.2(4)

C(27)-N(3)-C(19)-C(19)#2 -13.6(4)

C(20)-N(3)-C(19)-C(19)#2 170.5(2)

C(19)-N(3)-C(20)-C(21) -3.4(4)

C(27)-N(3)-C(20)-C(21) -179.7(3)

C(19)-N(4)-C(21)-C(22) -132.6(3)

C(19)-N(4)-C(21)-C(20) -5.2(4)

N(3)-C(20)-C(21)-N(4) 5.1(4)

N(3)-C(20)-C(21)-C(22) 130.1(3)

N(4)-C(21)-C(22)-C(23) -172.3(3)

C(20)-C(21)-C(22)-C(23) 66.3(4)

N(4)-C(21)-C(22)-C(26) -59.7(4)

C(20)-C(21)-C(22)-C(26) 178.9(3)

C(21)-C(22)-C(23)-C(24) 87.3(4)

C(26)-C(22)-C(23)-C(24) -31.1(4)

C(22)-C(23)-C(24)-C(25) 18.5(5)

C(23)-C(24)-C(25)-C(26) 2.8(6)

C(24)-C(25)-C(26)-C(22) -22.8(5)

C(21)-C(22)-C(26)-C(25) -85.8(4)

C(23)-C(22)-C(26)-C(25) 33.0(4)

C(19)-N(3)-C(27)-C(28) 153.4(3)

C(20)-N(3)-C(27)-C(28) -31.3(4)

C(19)-N(3)-C(27)-C(32) -27.8(4)

C(20)-N(3)-C(27)-C(32) 147.5(3)

C(32)-C(27)-C(28)-C(29) 0.7(5)

N(3)-C(27)-C(28)-C(29) 179.6(3)

C(27)-C(28)-C(29)-C(30) -0.1(6)

C(28)-C(29)-C(30)-C(31) -0.7(6)

C(29)-C(30)-C(31)-C(32) 0.8(5)

C(29)-C(30)-C(31)-C(33) 179.5(4)

C(30)-C(31)-C(32)-C(27) -0.1(4)

C(33)-C(31)-C(32)-C(27) -178.7(3)

C(28)-C(27)-C(32)-C(31) -0.7(4)

N(3)-C(27)-C(32)-C(31) -179.4(2)

C(32)-C(31)-C(33)-C(34) -136.2(4)

C(30)-C(31)-C(33)-C(34) 45.2(5)

C(32)-C(31)-C(33)-C(36) -10.9(5)

C(30)-C(31)-C(33)-C(36) 170.5(4)

C(32)-C(31)-C(33)-C(35) 106.0(4)

C(30)-C(31)-C(33)-C(35) -72.6(5)

________________________________________________________________

Symmetry transformations used to generate equivalent atoms:

#1 -x+1,y,-z #2 -x+1,y,-z+1

Table 7. Hydrogen bonds for cu_d8v21312_0m [Å and °].

____________________________________________________________________________

D-H...A d(D-H) d(H...A) d(D...A) <(DHA)

____________________________________________________________________________
